# Development of the First Small-Molecule Inhibitor Targeting Oncostatin M for Treatment of Breast Cancer

**DOI:** 10.1021/acs.jmedchem.4c03233

**Published:** 2025-08-01

**Authors:** Cody L. Wolf, Andrea Feci, Joseph P. Tuccinardi, Grace H. Coughlin, Kelsey A. Holdaway, Thaaer Muhammed, Clyde Pruett, Darren Lighter, Cooper McGrath, Terrell Engmann, Maria Pou-Torres, Brittany Rushing, Luke Woodbury, Sierra E. Haile, Hannah Scott, Ken Tawara, Simion Dinca, Dong Xu, Matthew D. King, Lisa Rose Warner, Cheryl L. Jorcyk, Don L. Warner

**Affiliations:** † Department of Biomolecular Sciences, 1791 Boise State University, Boise, Idaho 83725, United States; ‡ Department of Biological Sciences, 1791 Boise State University, Boise, Idaho 83725, United States; § Department of Chemistry and Biochemistry, 1791 Boise State University, Boise, Idaho 83725, United States; ∥ Biomedical Research Institute, 1791 Boise State University, Boise, Idaho 83725, United States; ⊥ Biomedical and Pharmaceutical Sciences, 15470 Idaho State University, Meridian, Idaho 83642, United States

## Abstract

Oncostatin M (OSM) is a proinflammatory cytokine implicated in inflammatory diseases and multiple cancers, especially breast cancer. To date, no federally approved anti-OSM therapeutics exist. We computationally screened ∼1.65 million compounds to identify small-molecule inhibitors (SMIs) of the OSM, and candidates were validated in human breast cancer models. We identified a tetrasubstituted furan (**SMI-10**) that inhibited OSM signaling, and optimization generated **SMI-10B** (*K*_D_ = 12.9 μM) and **SMI-10B13** (*K*_D_ = 6.6 μM). SMI-10B13 strongly inhibited OSM-mediated STAT3 phosphorylation in T47D and MCF-7 cell lines (IC_50_= 136 and 164 nM, respectively). Fluorescence quenching, NMR, and surface plasmon resonance assays were used to characterize SMI/OSM interactions and identify a number of analogs with low-micromolar affinity for OSM. In a human breast cancer mouse model, **SMI-10B13** reduced tumor growth (*p* < 0.001). Kaplan–Meier analysis showed improved survival in **SMI-10B13**-treated mice (*p* = 0.04), highlighting its potential as the first anti-OSM therapeutic to inhibit breast cancer progression and extend survival.

## Graphical abstract

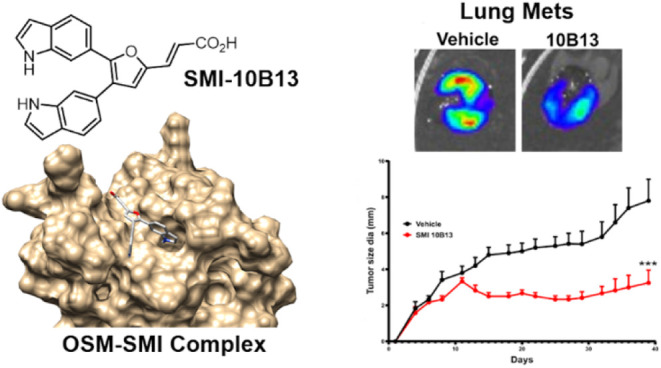

## Introduction

While medical advancements in detection and treatment have drastically increased the survivability rate for breast cancer patients, it is estimated that over 42,000 women will die in the United States during 2024 alone.^1^ Research efforts to identify novel targets and develop therapeutics to treat patients within various subtypes are ongoing and needed to improve patient survival outcomes. Over the past few years, chronic inflammation and the inflammatory cytokines that drive it have been associated with a worse prognosis in breast cancer patients and have been investigated as potential targets for intervention.

The interleukin-6 (IL-6) family of inflammatory cytokines consists of IL-6, IL-11, IL-27, leukemia inhibitory factor (LIF), ciliary neurotrophic factor (CNTF), cardiotrophin 1 (CT-1), cardiotrophin-like cytokine factor 1 (CLCLF1), and oncostatin M (OSM). IL-6, LIF, and OSM, in particular, have been investigated as potential therapeutic targets in cancer.^2,3^ The physiological role of OSM, pleiotropic in nature, is similar to other IL-6 family members and is necessary for a variety of inflammatory processes, as well as stem cell differentiation, bone remodeling, liver regeneration, wound healing, and hematopoiesis.^4–8^ While early studies evaluated the role of IL-6 in cancer,^9–11^ OSM has recently been identified as another significant contributor to cancer development and metastasis.^9–11^ Increased expression of OSM has been correlated with the recurrence of tumors and decreased survival in breast cancer patients.^12^ Previous work from our lab and others has demonstrated that OSM is responsible for breast tumor cell detachment, invasion, migration, and metastasis to distant sites such as the bone, brain, liver, and lung.^13,14^ Further, OSM promotes the expression of tumorigenic proteins such as proteases, including cathepsins and matrix metalloproteinases (MMPs), proangiogenic factors such as vascular endothelial growth factor (VEGF) and hypoxia-inducible factor 1α (HIF1α), as well as IL-6.^12,15–17^ OSM is also a powerful modulator for the breast tumor microenvironment. Evidence has shown it remains bioactive in the extracellular matrix (ECM) for extended periods of time, promotes extracellular matrix remodeling in breast cancer through lysyl oxidase-like 2 (LOXL2) upregulation, and recruits neutrophils to cancer cells for release of additional OSM.^18–20^ *In vivo*, OSM has been shown to increase circulating tumor cell (CTC) counts, as well as breast cancer metastasis to bone and lung.^13,14^

OSM, like all other members of the IL-6 family, is a four α-helical bundle cytokine.^21^ OSM activates its signaling pathways by interacting with two separate receptor complexes: the OSM receptor (OSMR), which consists of glycoprotein 130 (gp130) and OSMRβ, and, to a lesser degree, the LIF receptor complex consisting of gp130/LIFRβ.^21–25^ Through the main complex for OSM signaling, OSM binds first to gp130 and then recruits OSMRβ, activating several signaling cascades, including the Janus kinase/STAT3 (JAK/STAT3), PI3K/Protein Kinase B (PI3K/AKT), MAPK/ERK (see Figure 1A), as well as the Jun N-terminal kinase (JNK) pathway.^26–28^ Due to a gene duplication event, OSM is genetically and structurally similar to LIF^29^ and, as a result, can also interact with LIFRβ with a lower affinity than with OSMRβ.^22–25,30,31^ The physiological relevance of OSM binding to the LIFR is currently unknown.^32,33^ After the publication of the first molecular structure of OSM in 2000 by Deller et al., two distinct sites responsible for receptor complex binding were discovered.^34^ The two receptor-binding sites are named site II, responsible for OSM binding with gp130, and site III, responsible for OSM binding with OSMRβ and LIFRβ (Figure 1B).^24,34^ While the crystal structure of OSMRβ has yet to be fully elucidated, computational models of OSM-OSMRβ (based on LIF-LIFRβ structure) and molecular substitution and alanine-scanning experiments comparing OSM and LIF revealed several amino acids responsible for the OSM-OSMRβ interaction: mainly Tyr-34, Gln-38, Gly-39, and Leu-45 in OSM’s AB loop, and Pro-153, Phe-160, Gln-161, and Lys-163 in helix D (Figure 1B).^35,36^

**Figure 1:**
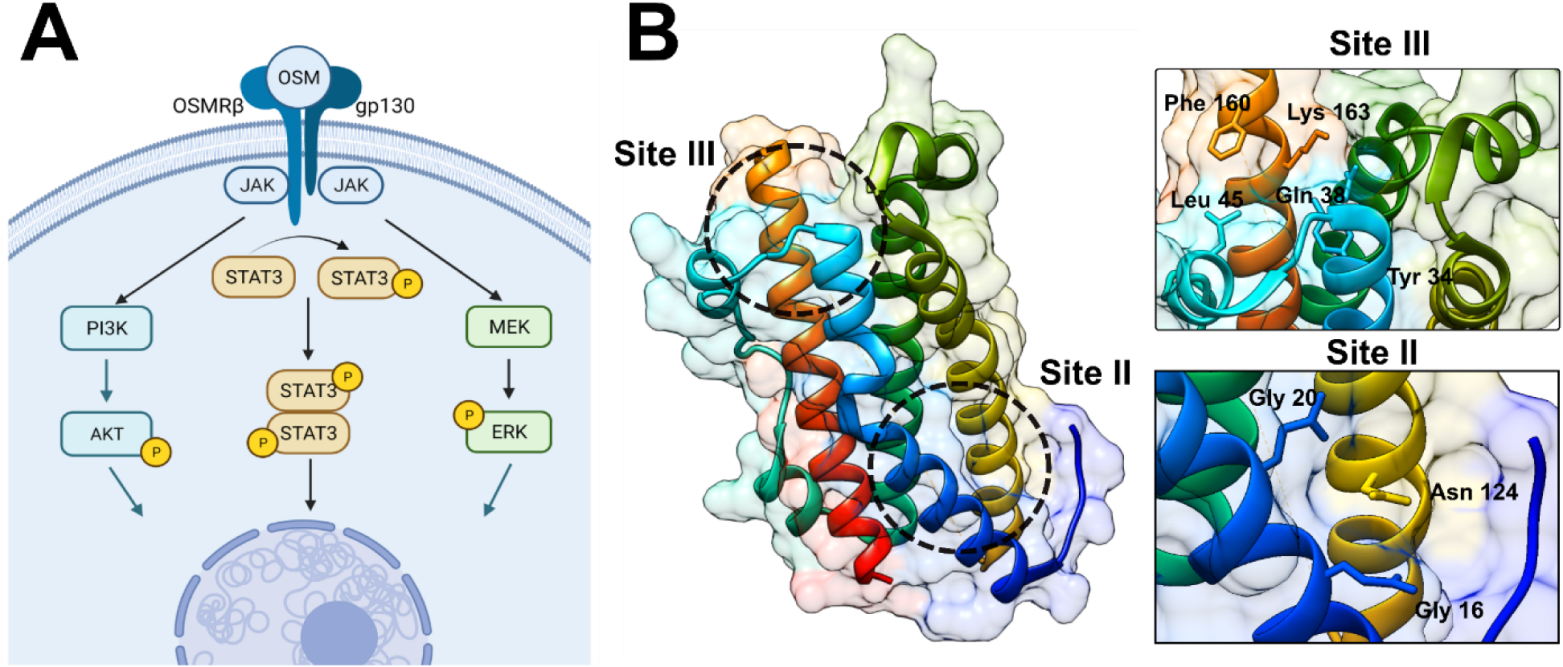
OSM structure and signaling. (A) In order to form a complete signaling complex, OSM first binds to gp130, which then allows for the recruitment of LIFRβ or OSMRβ. Afterward, several signaling cascades are activated—mainly the JAK/STAT3, PI3K/AKT, MAPK/ERK, and JNK pathways (not shown). (B) OSM is a four α-helical bundle protein with two distinct binding pockets for ligand–receptor interaction. Site II is responsible for gp130 binding and Site III is utilized for interaction with OSMRβ.

Our research has extensively implicated OSM in breast tumor progression, and our lab and others have identified it as a potential therapeutic target for breast cancer, as well as rheumatoid arthritis, systemic scleroderma, inflammatory bowel disease, and a variety of cancers outside of breast cancer, such as prostate, ovarian, and gastric.^37–43^ While it is clear that improved treatments are necessary for these inflammatory diseases, therapeutic intervention with the OSM has remained elusive. While no clinical therapeutics targeting OSM signaling have received FDA approval, three clinical trials for monoclonal antibodies targeting OSM or OSMRβ have been initiated for the treatment of rheumatoid arthritis, prurigo nodularis, or systemic sclerosis.^37,38,44,45^ Monoclonal antibodies, while effective, can be difficult and expensive to develop, are more challenging to administer to patients, and may create more severe side effects than alternative drug strategies.^46,47^ For these reasons, small molecules are often preferred as drug candidates.

Here, we report the first-time synthesis of novel small molecule inhibitors (SMIs) designed to prevent the interaction of the OSM-gp130/OSMRβ for the inhibition of the activation of the signaling of the OSM in breast cancer progression. An initial high-throughput virtual screen identified **SMI-10** as a promising compound that targets OSM and inhibits its receptor binding. Using a diversity-oriented synthetic approach guided by computational modeling, we generated first- and second-generation compounds with unique motifs at the 2-, 4-, and 5-positions around the five-membered furan core. To quantify the inhibition of OSM-induced signaling, fluorescence quenching assays, enzyme-linked immunosorbent assays (ELISAs), and immunoblot analyses were performed. We previously described the synthesis of an **SMI-10** analog, so-called **SMI-10B**, that binds to OSM’s site III pocket with a dissociation constant (*K*_D_) below 13 μM.^48^ This initial report was independently supported by Du et al., who computationally analyzed the binding of **SMI-10B** to OSM.^49^

This work also describes the discovery, synthesis, binding properties, and *in vitro* inhibitory activity of additional SMI-10 analogs. Furthermore, we report that, upon evaluation using an *in vivo* ER+ human breast cancer model, the analog **SMI-10B13** reduces tumor burden and metastasis. The results suggest that these novel SMIs may provide a basis for new therapeutic interventions for patients with breast cancer and other inflammatory diseases.

## Results and Discussion

### High-Throughput Virtual Docking Screen and Hit Validation

Without a high-resolution structure of the OSM-gp130/OSMRβ complex and without any known small molecules with activity against the OSM, our initial efforts to discover a lead compound for inhibition of the OSM focused on a *de novo* computational screening approach. We began by using the AutoLigand program to scan the protein surface to identify potential ligand-binding sites, which identified binding site III as the most promising binding pocket based on total volume and energy-to-volume ratios. Furthermore, previous literature reports showed that mutations of amino acid residues in binding site III drastically reduced OSM/OSMRβ-induced JAK/STAT3 signaling, suggesting that this binding site is where OSM recruits the receptor.^34,35^

Targeting site III, we conducted a high-throughput virtual screen (HTVS) of ∼1.65 million compounds in the National Cancer Institute (NCI) open and the ZINC databases using AutoDock 4.2 ^50,51^ to identify potential inhibitors of OSM (Figure 2A,B). This screen identified several initial hits with predicted binding constants <10of <10 and/or binding free energies more negative than −5.0 kcal/mol. The top hits from the virtual screen were tested experimentally for inhibition of OSM-induced phosphorylation of STAT3 via ELISA in the MDA-MB-231 human breast cancer cell line (Figure 2C). The top three compounds identified via ELISA, hereafter designated as **SMI-8**, **SMI-10**, and **SMI-11,** were tested for the inhibition of the-mediated signaling foracrossl pathways via immunoblot analysis in MDA-MB-231 (Figure 2D) and T47D (Figure 2E) cells. Of the three compounds, **SMI-8** was the most successful analog as it displayed almost complete inhibition of all OSM signaling cascades (Figure 2D,E). However, issues associated with its synthesis and predicted poor drug-like properties (i.e., reactive functional groups) deprioritized its optimization, although it remains under investigation. Additionally, while **SMI-11** performed well in MDA-MB-231 breast cancer cells, it proved to be much less effective in other cell lines. Taking each of these considerations together, we thus focused our efforts on developing analogs of **SMI-10** (Figure 2F) to improve binding within site III and optimize for inhibition of OSM signaling.

**Figure 2:**
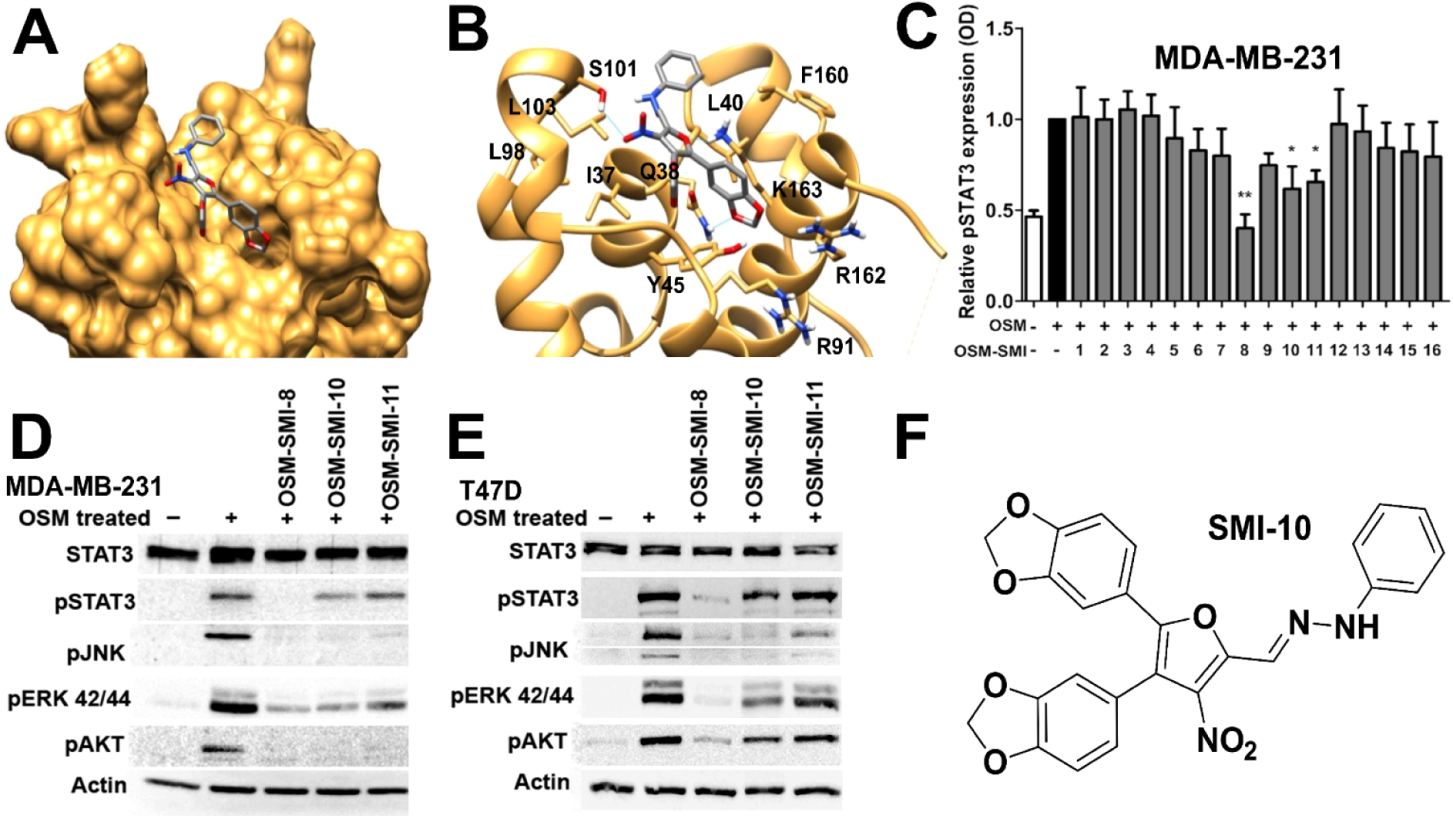
High-throughput virtual screening identifies three novel compounds capable of interacting with site III of OSM. (A) Virtual screening analysis of over 300,000 compounds identified 16 top molecules to interact with crucial amino within Site III of OSM (**SMI-10** displayed in (A). (B) Autodock Vina-computationally predicted pose of parent **SMI-10** docked to the crystal structure of OSM (PDB: 1EVS), with literature amino acids that have a role in OSMRβ binding shown. (C) MDA-MB-231 human breast cancer cells were treated with OSM (10 ng/mL) and/or SMI (10 μM) for 30 min, analyzed via pSTAT3 ELISA. **SMI-8**, **SMI-10**, and **SMI-11** were identified as effective inhibitors of OSM. (D) MDA-MB-231 and (E) T47D human breast cancer cell lines were treated with **SMI-8**, **SMI-10**, and **SMI-11** (10 μM) and OSM (10 ng/mL) for 30 min, and immunoblots were performed for pSTAT3, pAKT, pJNK, pERK, and β-actin. (F) Structure of parent SMI-10. Data are presented as mean ± SD relative to + OSM treatment, analyzed by one-way ANOVA with Tukey’s posttest *p < 0.05, **p < 0.01.

### Rational Design and Synthesis of **SMI-10** Analogs

Structurally, SMI-10 contains a 2,3,4,5-tetrasubstituted furan core appended with 4,5-bis­(benzo­[d]­[1,3]­dioxol-5-yl) substituents, a nitro group at the 3-position, and an *N*- phenylhydrazone group at the 2-position. Computational docking studies suggested that potentially important interactions include hydrogen bonding between the furan oxygen and positively charged Lys-163, hydrogen bonding and cation/pi interactions between the 5-benzodioxyl group and two positively charged Arg-91/Arg-162 residues, and hydrophobic interactions involving the phenylhydrazone and 4-benzodioxyl substituents. Despite the promising activity of **SMI-10** against OSM *in vitro*, the solvolytic instability of the phenylhydrazone group and the likely toxicity conferred by the aryl nitro group raised concerns about its potential as a lead compound.^52^ However, based on the computationally predicted binding pose, it was hypothesized that the nitro group was solvent-exposed and could be removed with minimal impact on activity, and that the phenylhydrazone could be synthetically replaced with a less labile group that would retain the important hydrogen bonding interactions while circumventing issues associated with toxicity and instability. With these considerations in mind, we set out to design and synthesize analogs of compound **SMI-10** that retained the 4,5-bis­(benzo­[d]­[1,3]­dioxol-5-yl)­furan scaffold with varying groups at the 2-position of the furan core.

With no previously reported synthesis of compound **SMI-10** or closely related compounds, we first developed a synthetic scheme to access such analogs (Scheme 1). It was envisioned that installation of the benzodioxole groups could be accomplished using benzodioxyl-5-boronic acid in a one-pot Suzuki-Miyaura diarylation of 4,5-dibromo-2-furfural or 4,5-dibromo-2-furoic carboxylic acid derivatives. Using the aldehyde or carboxylate as a handle, diversification at this position easily facilitated the formation of a small library of 2-substituted analogs, as elaborated on in Scheme 2.

**Scheme 1:**
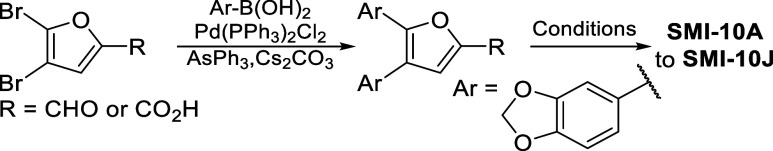
General Synthetic Route to **SMIs-10A–10J**

**Scheme 2:**
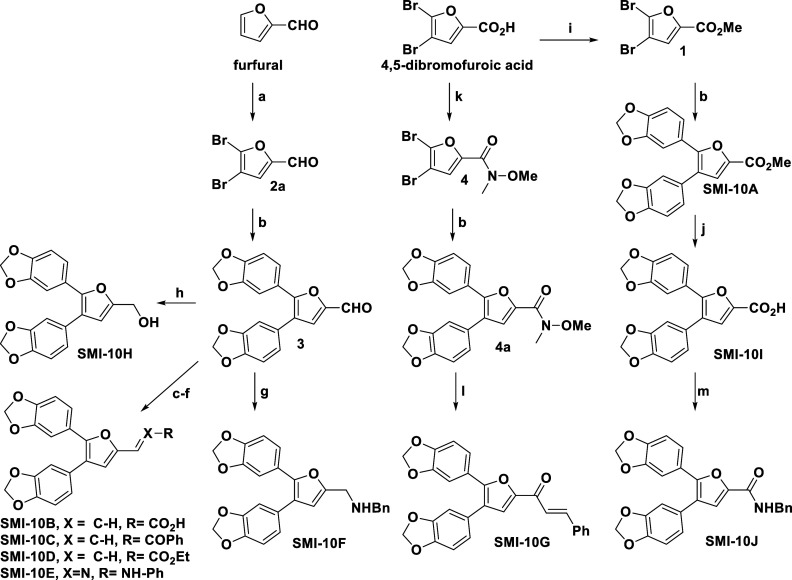
Full Synthesis of **SMI** **10A–J** Analogs^a^

a Reagents and conditions: (**a**) AlCl_3_, Br_2_, CH_2_Cl_2_, 0 °C to rt. (**b**) 3,4-Methylenedioxyphenylboronic acid, Pd­(PPh_3_)_2_Cl_2_, AsPh_3_, Cs_2_CO_3_, DMF, 90 °C. (**c**) *X* = C–H, *R* = CO_2_H, malonic acid, pyridine, piperidine, 120 °C. (**d**) X = C–H, *R* = COPh, diethyl­(2-oxo-2-phenylethyl)­phosphonate, NaH, THF, 0 °C to rt. (**e**) *X* = C–H, *R* = CO_2_Et, triethylphosphonoacetate, NaH, EtOH, 0 °C to rt. (**f**) *X* = N, *R* = N-Ph, phenylhydrazine, Sc­(OTf)_3_, CHCl_3_, rt. (**g**) BnNH_2_, benzene, 85 °C, then NaBH_3_CN, TFA, MeOH, 0 °C to rt. (**h**) NaBH_4_, MeOH, 0 °C to rt. (**i**) MeOH, H_2_SO_4_, 80 °C. (**j**) LiOH, THF, H_2_O, 70 °C. (**k**) CH_2_Cl_2_, N,O-dimethylhydroxylamineHydrochloride, Et_3_N, 1,3-dicyclohexylcarbodiimide, dimethylaminopyridine, rt. (**l**) Styryl magnesium bromide, THF, then NH_4_Cl (aq.). (**m**) CH_2_Cl_2_, DMF, SOCl_2_, 50 °C; then Et_2_O, BnNH_2_, Et_3_N, rt.

As shown in Scheme 2, the synthesis of the first-generation **SMI-10** analogs (**SMI-10A–J**) was developed from two key starting materials: furfural (**SMI-10B–F** and **10H**) and a brominated furanoic acid derivative (**SMI-10A**, **10G**, **10I**, and **10J**). From the furfural-derived route, bromination (a) led to the furfural dibromide intermediate **2a**. Suzuki coupling (b) with 3,4-methylenedioxyphenylboronic acid under Pd-catalyzed conditions provided the key triaryl scaffold **3**. Knoevenagel condensation (c–e) with either malonic acid or phosphonate derivatives under basic conditions gave the propenoic acid analogs **SMI-10B–D**. Further diversification was achieved via condensation with phenylhydrazine (f) or benzylhydrazine (g) to yield the phenylhydrazone analog **SMI-10E** or the benzylamine analog **SMI-10F**, respectively. Reduction (h) of the carbonyl functionality of **3** with sodium borohydride led to alcohol **SMI-10H**. From the dibrominated-furanoic acid route, we performed esterification (i) to obtain **1**, followed by Suzuki coupling (b) to obtain disubstituted methyl ester **SMI-10A**. Saponification (j) of **SMI-10A** led to the synthesis of **SMI-10I**. Two-step amidation (**m**) of **SMI-10I** led to benzylamine **SMI-10J**. Finally, to obtain **SMI-10G**, we converted the furanoic acid into the Weinreb amide **4** via carbodiimide-mediated coupling (k), followed by Suzuki coupling (b) to obtain **4a** and Grignard addition (l) to obtain the chalcone analog **SMI-10G**.

### Biochemical and *In Vitro* Evaluation of Compounds **SMI-10A**–**10J**

With a small library of compounds in hand, we sought to test their binding affinity and inhibitory activity against the OSM *in vitro*. Table 1 presents *K*_D_ values obtained from fluorescence quenching assays conducted for each SMI, as previously described by Mass et al.^48^ Using this technique, compounds **SMI-10B** and **SMI-10F** were shown to have the lowest dissociation constants at 12.9 ± 1.5 and 16.8 ± 4.1 μM, respectively.

**Table 1:**
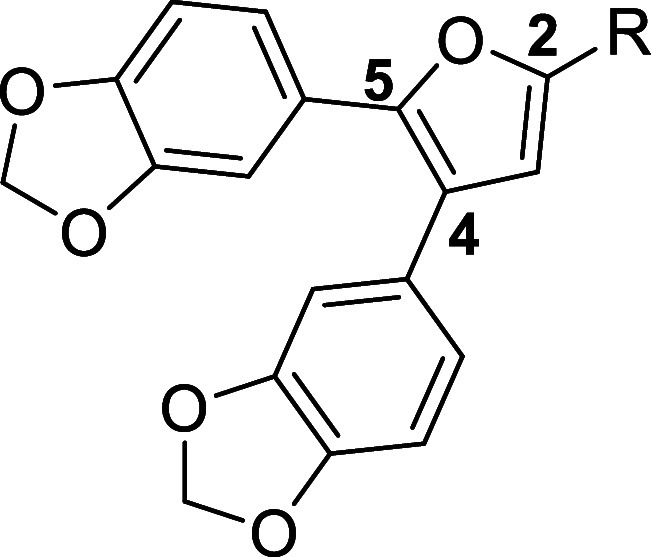

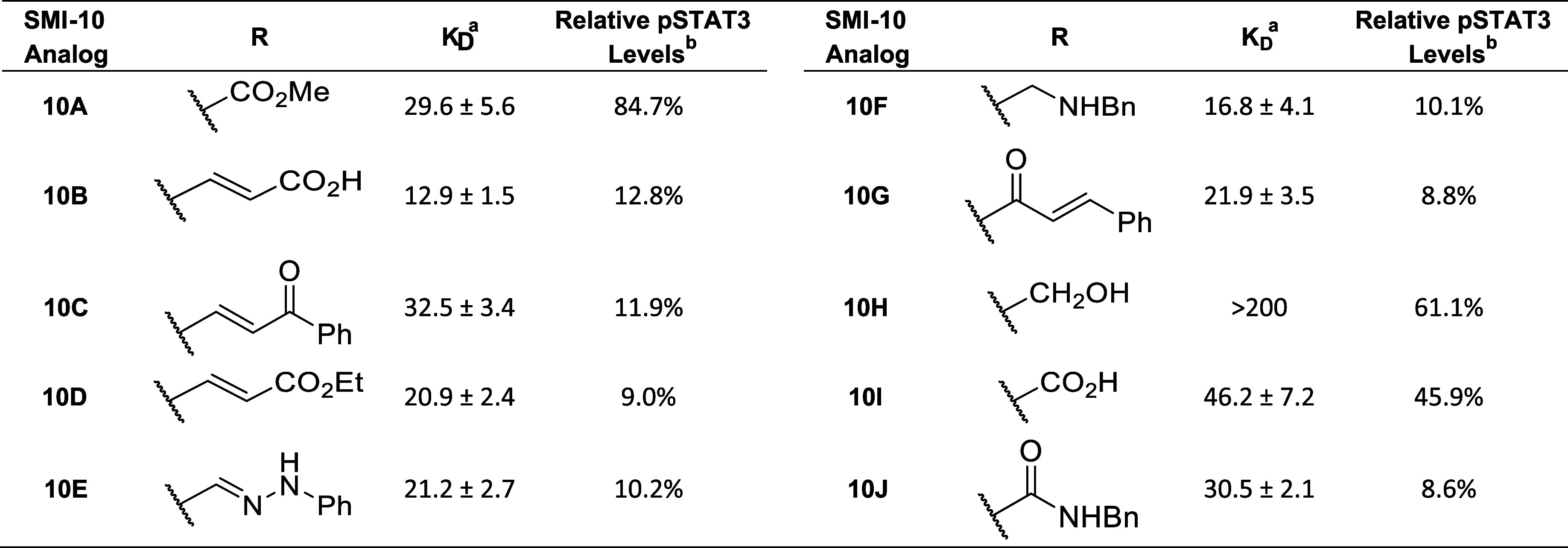
Dissociation Constants and Relative PSTAT3 Levels of 2,4,5-Trisubstituted Furan Analogs **SMI-10A–10J** ^a^ ^,^ ^b^

a Average of three independent replicates; errors are reported at 95% confidence level, and values are reported in μM.

b Relative to + OSM, baseline expression levels of pSTAT3 were determined by subtracting the no-treatment blank in T47D human breast cancer cell lines, as determined by ELISA.

To test the effect of SMIs *in vitro*, each SMI was incubated with OSM for a period of 1 h at 37 °C, and T47D and MDA-MB-231 human breast cancer cell lines were subsequently treated with OSM (10 ng/mL) and/or **SMI-10** (10 μM) analogs for 30 min and measured for pSTAT3 levels via ELISA. The results of these assays are presented graphically in Figure 3 and, for T47D cells, in Table 1 as a percentage of reduction from +OSM alone.

**Figure 3:**
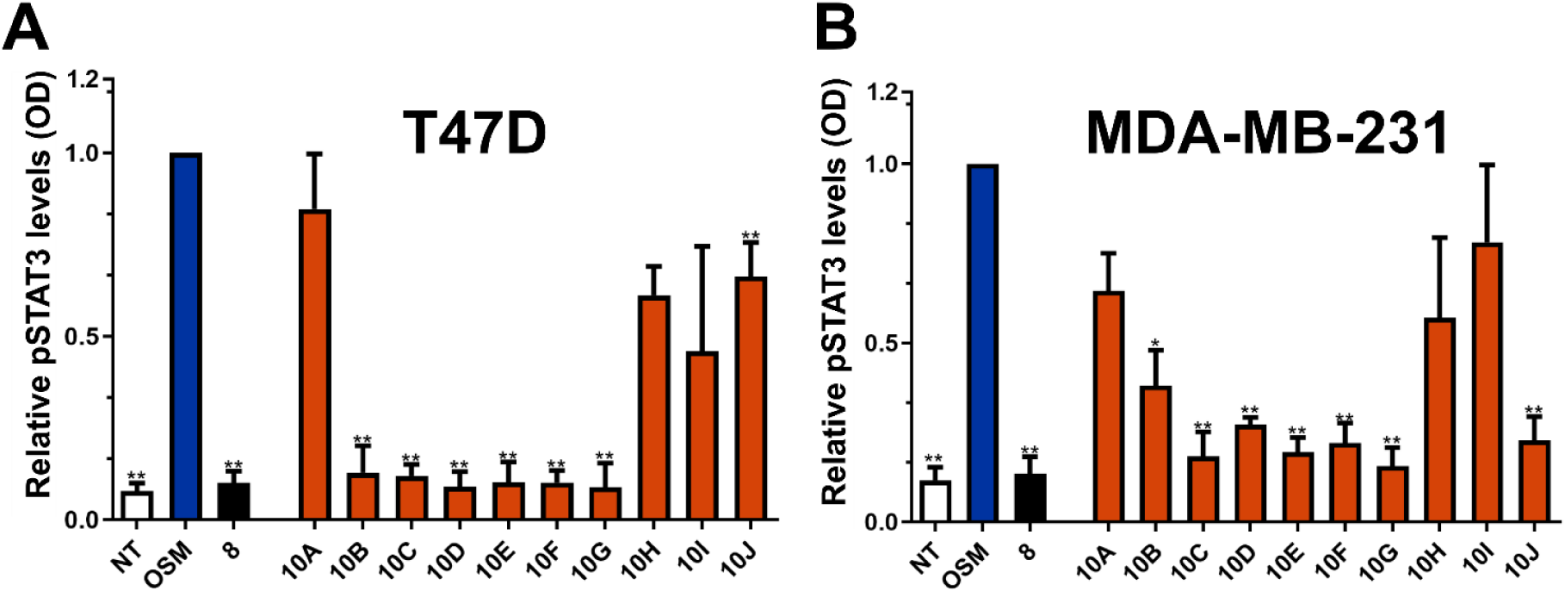
First-generation SMI-10 analogs inhibit OSM-induced pSTAT3 via ELISA. (A) Human T47D and (B) MDA-MB-231 cells were treated with **SMI-10A-J** analogs (10 μM) and OSM (10 ng/mL) for 30 min, and cell lysates were analyzed via ELISA. *In vitro* analysis identified several compounds capable of inhibiting OSM-induced pSTAT3 via ELISA, including **10B**, **10C**,**10D**, **10E**, **10F**, and **10G**. **SMI-8** is used as a positive control. Data are expressed as mean ± SD and assessed relative to + OSM treatment by one-way ANOVA with Tukey’s posttest *p < 0.05, **p < 0.01.

The inhibition of OSM-mediated signaling was also examined via immunoblot analysis. T47D cells treated with OSM ± SMI were evaluated for inhibition of OSM activation of the JAK/STAT3, PI3K/AKT, MAPK/ERK, and JNK signaling cascades, as determined by levels of pSTAT3, pAKT, pERK, and pJNK, respectively. From the immunoblot analysis, **SMI-10B**, **10C**, **10D**, **10E**, **10F**, **10G**, and **10J** all displayed significant reductions, near nontreated levels, in multiple OSM-mediated signaling pathways, particularly pSTAT3, pAKT, and pERK. (Figure S1).

The combined fluorescence quenching, ELISA, and immunoblot analysis of the initial **SMI-10** analogs provided insight for structural optimization to increase the level of binding of the OSM and create a more effective drug. First, since none of the new analogs contain a 3-nitro group, the data indicate that this moiety is nonessential for inhibition, as was suggested by preliminary docking studies. Next, only **SMI-10E** contains 2-phenylhydrazone present in the parent **SMI-10**. Since this functional group was found to be labile and prone to degradation, it was gratifying to note that good activity could be achieved using different substitution patterns. The data demonstrate that shorter substituents at the 2-position (**10A**, **10H**, and **10I**) consistently had lower binding affinity and activity against the OSM, as determined by fluorescence quenching, ELISA, and immunoblot analysis, while chain lengthening at both positions (**10C**, **10D**, **10E**, **10F**, and **10G**) appeared to increase activity *in vitro* and improve binding affinity.

While a longer/bulkier group at the 2-position showed dramatically improved binding of the SMI to the OSM, the identity of the group at this position did not show appreciable differences by fluorescence quenching or by *in vitro* analysis, as **SMI-10C**, **10D**, **10E**, **10F**, and **10G** returned similar values. However, fluorescence quenching experiments identified 2-propenoic acid derivative **SMI-10B** as the most potent compound in the series. This analog also showed good activity against the OSM *in vitro*, had increased aqueous solubility compared to other analogs, and could be rapidly prepared using a short synthetic sequence. As a result of these positive characteristics, **SMI-10B** was selected for further investigation and structural optimization.

### SAR-Guided Optimization and Synthesis of **SMI-10B** Analogs

To improve binding to OSM and enhance biological activity, additional structural optimization of **SMI-10B** was undertaken (see Table 2 for **SMI-10B** analogs and Scheme 3 for the synthetic scheme). After observing the positive impact of the propenoic acid chain at the furan 2-position, this moiety was generally maintained in the next iteration of structural optimization, although the effect of a saturated side chain was also examined. Additional analogs were prepared to determine the importance of the oxygen heteroatom in the furan core, so nitrogen and sulfur (e.g., pyrrole and thiophene) variants were prepared. Finally, the impact of the aryl groups at the 4- and 5-positions was also evaluated. As demonstrated in Scheme 3, the synthesis of the second-generation analogs **SMI-10B1** to **SMI-10B18** was accomplished from commercially available 3,5-dibromofurfural, brominated thiophene, or brominated pyrrole carbaldehydes. Suzuki–Miyaura cross-coupling was used to install aryl substituents on the furfural core. Reaction with a single arylboronic acid (a) yielded symmetrically diarylated products, while sequential coupling with two different arylboronic acids (b) produced unsymmetrical biaryl intermediates. The coupled products were then subjected to base-catalyzed Doebner–Knoevenagel condensation with malonic acid (**c**) to afford the corresponding propenoic acid derivatives (**SMI-10B1–B10 and B13**). Suzuki coupling on monobrominated furfural (d) generated analogs **SMI-10B11** and **10B12**. To access a second subset of analogs (**SMI-10B14–B18**) with a saturated propanoic acid chain at the furan 2-position, we introduced an ethyl propanoate chain via a Horner–Wadsworth–Emmons olefination of dibrominated furfural using triethyl phosphonoacetate (e) to generate an enoate that was then reduced via a tosylhydrazone protocol (f) to generate the ethyl propanoate **5**. The brominated furan subsequently underwent symmetric bis-Suzuki-Miyaura coupling (a) or monocoupling (g) to yield monobrominated **6**, followed by purification and a second coupling sequence to generate the asymmetric ethyl propanoate. Subsequent saponification (**i**) generated propanoic acids **SMI-10B14** to **SMI-10B18**.

**Scheme 3:**
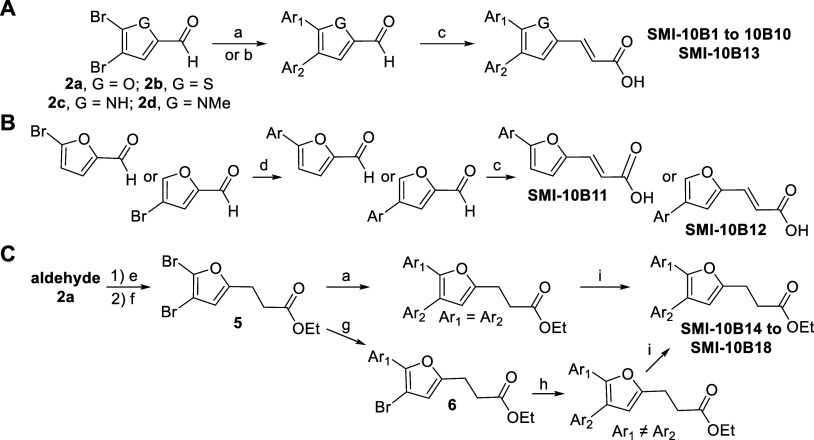
Synthesis of **S** **MI 10B** Analogs[Fn sch3-fn2]

a Reagents and Conditions: (a) Ar_1_ = Ar_2_: Arylboronic acid (2.2 equiv), Na_2_CO_3_ (5.0 equiv), Pd­(PPh_3_)_4_ (0.1 equiv), 90–110 °C (b) Ar_1_ ≠ Ar_2_: Ar_1_-Boronic acid (1.05 equiv), Na_2_CO_3_ (5.0 equiv), Pd­(PPh_3_)_4_ (0.1 equiv), 90–110 °C until Disappearance of the Starting Material by TLC then Ar_2_-Boronic Acid (2.0 equiv), 90 °C, 12 h. (c) Malonic Acid (10 equiv), Piperidine (cat.), Pyridine, 120 °C. (d) Arylboronic acid (2.2 equiv), Cs_2_CO_3_ (6 equiv), Pd­(PPh_3_)_2_Cl_2_ (16 mol %), AsPh_3_ (20 mol %), 90 °C. (e) triethyl Phosphonoacetate (2.2 equiv), Et_3_N (2.3 equiv) LiBr (2.4 equiv), 0 °C, 30 min. (f) TsNHNH_2_ (8 equiv), NaOAc (9 equiv), H_2_O, 100 °C, 11 h. (g) Ar_1_ ≠ Ar_2_: *3,4-(methylenedioxy)­phenylboronic acid* (1 equiv), Na_2_CO_3_ (5.0 equiv), Pd­(PPh_3_)_4_ (0.1 equiv), 110 °C, 12 h. (h) Ar_2_-Boronic acid (2.0 equiv), Pd­(PPh_3_)_4_ (0.1 equiv), 110 °C, until Disappearance of the Starting Material by TLC. (i) EtOH, 6 M NaOH (10 equiv), 90 °C, 30 min.

**Table 2:**
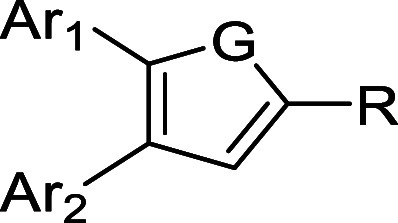

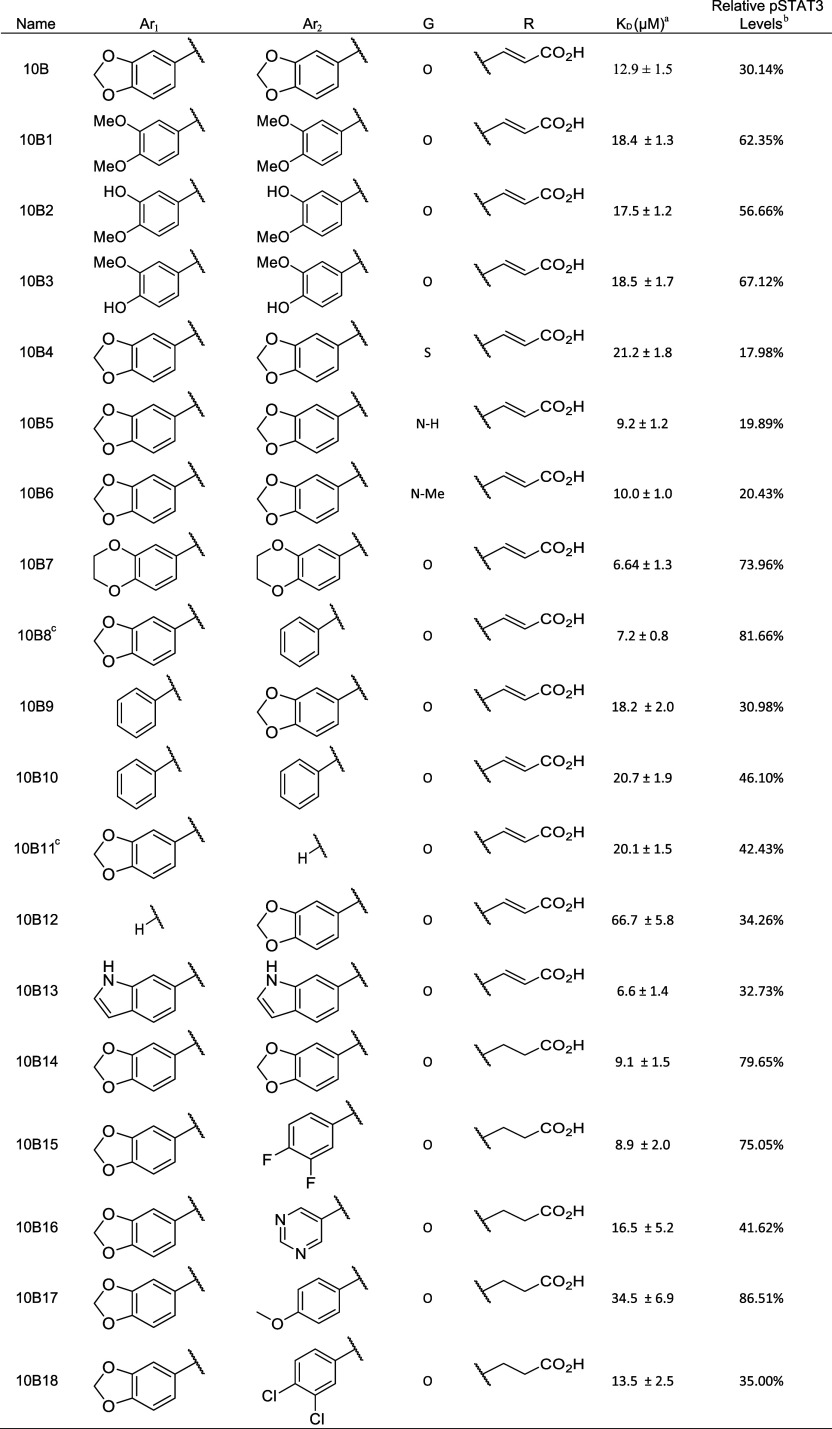
OSM-SMI Dissociation Constants and Relative pSTAT3 Levels in T47D Cells Post-Treatment with OSM and SMI^a^ ^,^ ^b^ ^,^ ^c^

a Average of three independent replicates, errors are reported at 95% confidence level.

b Relative to + OSM, for baseline levels of pSTAT3 by subtraction of no treatment blank in T47D human breast cancer cell lines.

c Due to nomenclature consistency issues, SMI-10B8 shown here was originally reported by our partners in Mass et al.^48^ as SMI-10B11, while SMI-10B11 shown here refers to a newly presented and different compound.

### Binding Affinities and *In Vitro* Efficacy of **SMI-10B** Analogs

The synthesized analogs **SMI-10B1** to **SMI-10B18** were tested for binding affinity to the OSM by fluorescence quenching assays and their ability to inhibit downstream cell signaling of the OSM using ELISA and immunoblot assays. Analysis of the binding affinities via fluorescence quenching (reported in Table 2) suggested that the introduction of diverse heteroatoms into the furan core, such as N–H and N–Me (**SMI-10B5** and **SMI-10B6**, *K*_D_ = 9.2 and 10.0 μM, respectively), resulted in increased activity relative to larger atoms like sulfur (S) or smaller atoms like oxygen (O). This difference can also be attributed to the collective influence of other properties: ionizability, the hard–soft acid–base (HSAB) character, ionization potential, and electronegativity. Nitrogen-containing groups (N–H and N–Me) generally have lower ionization potentials, making them more reactive, and less negative electronegativities compared to sulfur and oxygen, leading to stronger interaction capabilities for the N-containing ring. Additionally, the nitrogen in these groups is considered a “hard” base, which better facilitates electronic interactions such as hydrogen bonding with amino acids such as K163, compared to the thiophene, whose sulfur is a “soft” base, and the furan, which is a hard–soft borderline case. Looking at the 5-position, the large benzodioxyl group appeared to be essential for activity (binding affinity of **SMI-10B** > **SMI-10B9** > **SMI-10B12**), while introduction of a smaller aryl group in the 4-position (**SMI-10B8**, *K*_D_ = 7.2 μM) further increased binding affinity. A more flexible and larger 1,4-benzodioxane (**SMI-10B7,** *K*_D_ = 6.6 μM) or a more compact and hydrophobic indole (**SMI-10B13,** *K*_D_ = 6.6 μM), which remains an excellent electron donor, both performed better than the parent **SMI-10B** analog (*K*_D_ = 12.9 μM). In addition, a less rigid propanoic acid substituent at the 2-position (**SMI-10B14**, *K*_D_ = 9.1 μM) further improved the binding affinity compared to that of **SMI-10B**.

As before, we sought to directly test the ability of the **SMI-10B** analogs to block OSM signaling *in vitro*. SMIs were preincubated with OSM as described previously, and T47D human breast cancer cells were treated with OSM (10 ng/mL) ± SMI for 30 min and evaluated for pSTAT3 levels via ELISA (Figure 4). The most promising candidates from the ELISA data were **SMIs-10B4–B6**, **B9–B13**, **B16**, and **B18**; however, no significant trend was identified that correlated with the fluorescence quenching data regarding the specific roles of 4- and 5-position substituents.

**Figure 4:**
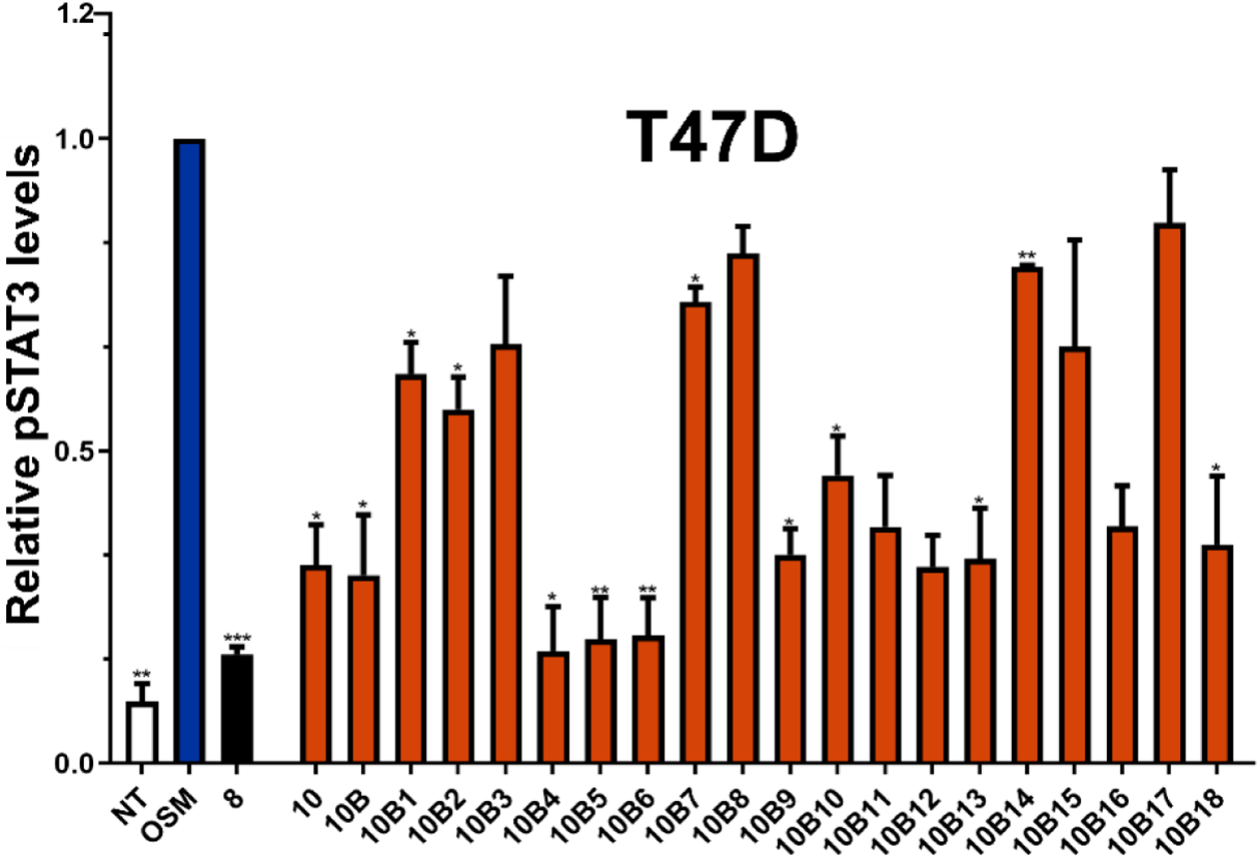
**10B** analogs with increased inhibition of OSM-mediated pSTAT3 levels. Human T47D breast cancer cells were treated with OSM (10 ng/mL) and **SMI-10B** analogs (10 μM) for 30 min, and cell lysates were evaluated for pSTAT3 induction via ELISA. Analysis of **10B** analogs suggests that further modification of **10B** creates more effective inhibitors of OSM, specifically **SMI-10B13**. Data are expressed as mean ± SD and assessed relative to + OSM treatment by one-way ANOVA with Tukey’s posttest *p < 0.05, **p < 0.01.

To further elucidate the role of functional groups in **SMI-10B** analogs, selected compounds were evaluated via immunoblot analysis (Figure S2). We selected **SMI-10B8** through **SMI-10B13** to maximize the diversity from parent **SMI-10B**, while **SMI-10B14** was tested to probe the role of alkyl chain saturation. T47D cells were treated with OSM and/or SMIs and evaluated for all OSM-mediated signaling cascades. Compared to **SMI-10B**, several compounds were better at inhibiting pSTAT3, pAKT, pJNK, and pERK signaling, especially **10B8**, **10B12**, **10B13**, and **10B14**. Due to **SMI-10B13’s** low *K*_D_ (Table 2), promising ELISA results (Figure 4), and top-performing ability at inhibiting multiple OSM-mediated signaling pathways (Figure S2), it was selected for further experiments.

### Binding Comparisons of **SMI-10B** and **SMI-10B13** by NMR Titrations of ^15^N OSM Reveals Conserved Specificity for Site III

To better understand how **SMI-10B** and **SMI-10B13** interact with OSM, we conducted a chemical shift perturbation (CSP) NMR assay where ^15^N-isotope-labeled OSM^1^-187 protein was titrated individually with each of the SMIs, and spectra were collected upon SMI addition. The spectra from a titration of ^15^N OSM with **SMI-10B** (Figure 5A,B) from new preparations of both protein and small molecule are identical to the spectra reported in Mass et al.,^48^ demonstrating reproducibility in the workflow.

**Figure 5:**
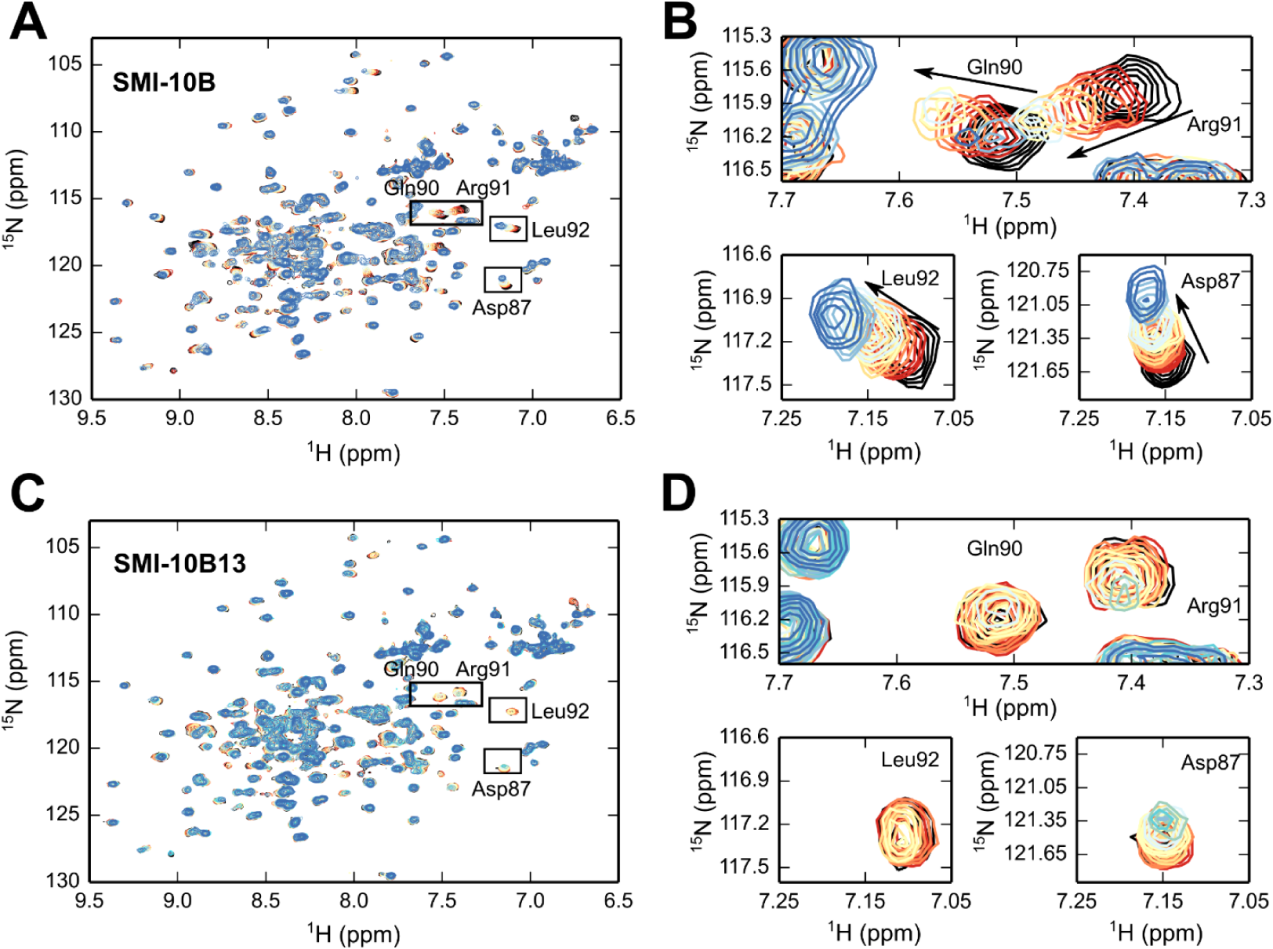
SMI-10B and SMI-10B13 NMR CSP assays with ^15^N OSM. (A) An overlay of ^1^H, ^15^N HSQC NMR spectra of 100 μM ^15^N-labeled OSM with cross peaks color-ramped from red to blue with increasing **SMI-10B** concentration, from 20 μM (red) to a final concentration of 260 μM (blue). The no-SMI control is shown in black. (B) Zoomed regions of panel A for Asp87, Gln90, Arg91, and Leu92. (C) An overlay of ^1^H, ^15^N HSQC NMR spectra of 100 μM ^15^N-labeled OSM with cross peaks color-ramped from red to blue with increasing **SMI-10B13** concentration, from 0.5 μM (red) to a final concentration of 60 μM (blue). Note that spectra of ^15^N-labeled OSM titrated with up to 200 μM **SMI-10B13** were collected but showed very little difference compared to 60 μM SMI and were therefore not included in this overlay. The no-SMI control is shown in black. (D) Zoomed regions of panel C for Asp87, Gln90, Arg91, and Leu92.

Several peaks shifted upon the addition of **SMI-10B** (Figure 5A,B), although only Asp87, Gln90, Arg91, and Leu92 have been assigned by comparison with the spectra of a glycosylated version of OSM for which peak assignments are available.^53^ These residues are located at or near the OSM binding site III and show clear shifts in the fast exchange regime, collected at 600 MHz field strength. Interestingly, when titrated with **SMI-10B13** (which has a higher observed affinity for OSM compared to **SMI-10B,** as measured by fluorescence quenching), the ^15^N OSM shows chemical shift perturbations of the same residues as **SMI-10B**, with the exception that most peaks are broadened at increasing SMI concentrations, at times to the point of disappearing (Figure 5C,D). This suggests a condition of intermediate exchange, which is often coincident with a slower off rate and, subsequently, a lower *K*_D_.^54^ While peak broadening can be due to conformational dynamics or exchange, the more likely cause, in this case, is that **SMI-10B13** is binding to the same site as **SMI-10B** with a higher affinity, consistent with the fluorescence quenching experiments.

### Lead Inhibitor **SMI-10B13** Potently Suppresses OSM-Mediated JAK/STAT3 Signaling Activation

**SMI-10B13** exhibited a similar amino acid shift pattern in CSP NMR experiments as the parent compound **SMI-10B** and the sister analog **SMI-10B8**, as reported here and by Mass et al.,^48^ confirming the conserved specificity of the analog family for site III of OSM. Among all tested analogs, **SMI-10B13** had the lowest dissociation constant (*K*_D_ = 6.6 ± 1.4 μM) by fluorescence quenching and reduced relative pSTAT3 expression to 32.7% compared to OSM treatment alone—ranking it fourth among the STAT3-suppressing compounds tested here. Based on this profile, **SMI-10B13** was selected as the lead compound for additional binding studies and *in vitro* and *in vivo* biological evaluation. To further explore the potency of **SMI-10B13**, an IC_50_ curve was generated for inhibition of STAT3 phosphorylation in T47D and MCF-7 cells, with an IC_50_ of 136 and 164 nM, respectively (Figure S3). ADMET profiling of **SMI-10B13** revealed favorable GI absorption, moderate lipophilicity, and no PAINS or Lipinski violations, although solubility and CYP inhibition remain concerns that should be addressed in future structural optimization (Table S1).

### Surface Plasmon Resonance Assays Reveal Possible Additional Allosteric Inhibition of Site II Receptor Recruitment by **SMI-10B13**

SPR assays were used to investigate the ability of **SMI-10B13** to disrupt the binding of the OSM receptor. We began our SPR assays by replicating the gp130-to-immobilized OSM binding kinetic experiment published by Chollangi et al.^31^ to evaluate the reliability of our setup. In this experiment, increasing concentrations of gp130 were injected, and the association and dissociation phases were monitored. We obtained a *K*_D_ value of 20.5 nM, which is comparable to the 22.69 nM dissociation constant previously reported.^31^ Next, since OSMRβ requires the formation of the gp130-OSM complex before binding to OSM, as previously demonstrated by Chollangi et al.,^31^ we initially attempted a surface competition binding assay to assess OSMRβ binding to a preformed, immobilized gp130-OSM complex. However, the signal generated was insufficient for meaningful results, likely due to the suboptimal availability of accessible binding sites for OSMRβ, although maximum feasible concentrations of both immobilized and injected gp130 were reached. As a result, we shifted our focus to assess whether **SMI-10B13** could influence the recruitment of gp130 to OSM in a surface competition binding assay. In this setup, gp130 and the SMI compete for immobilized OSM (fluorescence quenching was used to verify that **SMI-10B13** does not bind to gp130; data not shown). We observed that **SMI-10B13** almost completely inhibited the ability of gp130 to bind to immobilized OSM at a concentration of 100 μM, with an estimated IC_50_ between 30 and 100 μM (Figure S4). While this effect could be justified by site II competitive binding at high SMI concentrations, the NMR CSP experiments are indicative of SMI binding at site III (Figure 5). Therefore, the inhibition may involve an allosteric disruption of the OSM-gp130 interaction, consistent with the weak potency expected for small-molecule inhibitors targeting a high-affinity protein–protein interaction^55^ (*K*_D_ = 20.5 nM for gp130-OSM), as such compounds often require higher micromolar concentrations to exert measurable effects on protein–protein nanomolar-affinity interactions. Importantly, gp130 is proposed to dock at site II on OSM,^56^ whereas the NMR CSP experiments suggest that **SMI-10B13** binds at site III (Figure 5). Together, these data seem to point to an additional allosteric mode of inhibition by **SMI-10B13** that prevents the OSM from recruiting receptor subunit gp130 at high drug concentrations. However, consistent with our NMR mechanistic data, we believe the observed biological activity of SMI-10B13 is likely on-target and primarily due to the disruption of OSMRβ recruitment at site III, plausibly after the OSM-gp130 complex has already formed.

### **SMI-10B13** Suppresses Tumor Growth in an ER+ Breast Cancer Xenograft Model

To further examine the efficacy of **SMI-10B13** as a legitimate candidate for drug development, we elected to use an *in vivo* xenograft mouse model to evaluate **SMI-10B13’s** ability to inhibit breast cancer progression. It has been well established that OSM is associated with worse prognosis in breast cancer patients, particularly in ER+ patients.^12^ Due to this finding, we generated an ER+ orthotopic breast cancer model using human ER+ MCF-7-luc cells with constitutive OSM overexpression. This ER+ model successfully produces viable tumors for the evaluation of tumor growth and metastasis.

To evaluate **SMI-10B13** on OSM inhibition *in vivo*, athymic nu/nu mice were randomized and given equal dose of either vehicle or **SMI-10B13** (50 mg/kg) 12 h before tumor cell injection. Mice were then injected with 2 × 10^6^ cells in the fourth mammary fat pad and monitored for tumor growth over 40 days, receiving either vehicle or **SMI-10B13** treatment 3x weekly and evaluated for tumor growth until the end point of the experiment (Figure 6A). It is worth noting that none of the mice injected with SMI exhibited any signs of drug-related toxicity throughout the course of the experiment.

**Figure 6:**
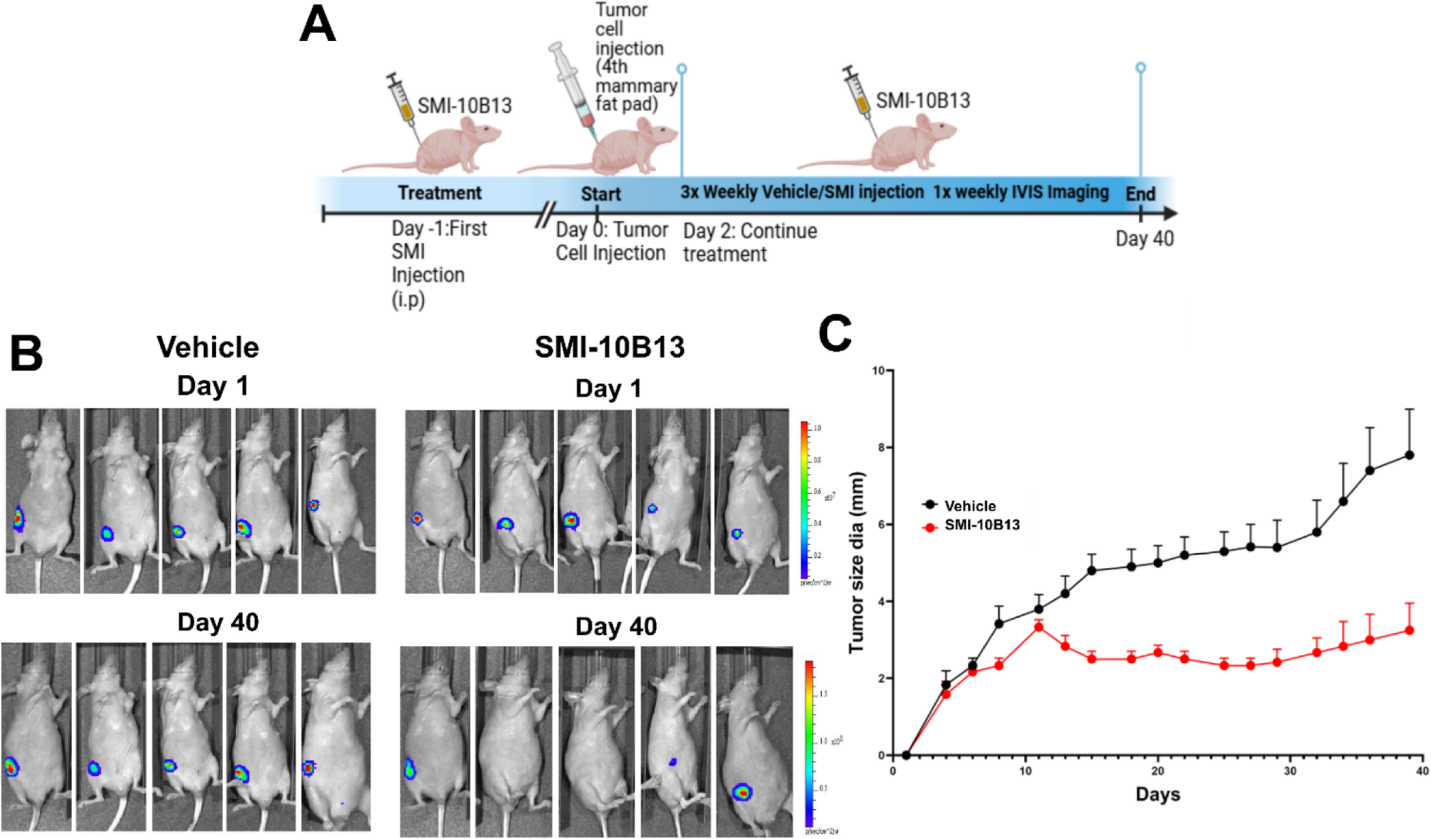
SMI-10B13 inhibits tumor growth *in vivo.* (A) Athymic nu/nu mice were injected with **SMI-10B13** or vehicle control (*n* = 6) 12 h before injection of MCF-7-luc-OSM-overexpressing cells. Afterward, mice were injected three times weekly for a period of 40 days with either vehicle or **SMI-10B13**. (B) Once weekly, mice were evaluated for tumor growth via bioluminescence imaging. Mice treated with **SMI-10B13** displayed a significant reduction in bioluminescent detection compared to vehicle control. (C) Tumor size diameter (mm) was also measured 3x weekly. At the end point of experiment, mice given **SMI-10B13** compared to vehicle control displayed significant reduction in tumor mm size, suggesting **SMI-10B13** reduces tumor growth *in vivo*. Data are expressed as mean + SD and assessed relative to + OSM treatment by unpaired *t*-test, ***p < 0.001.

Mice that received **SMI-10B13** showed a significant reduction (*p*-value <0.001) in tumor growth in comparison to vehicle control (Figure 6B,C). This evidence suggests that **SMI-10B13** is not only effective at inhibiting OSM-induced signaling cascades but also prevents tumor growth, indicating that this therapeutic may be beneficial for patients with high levels of OSM. Interestingly, **SMI-10B** and **SMI-10B13** display minimal to no cytotoxicity at high concentrations via an MTS assay (Figure S5). This, in addition to the lack of drug-related distress in treated mice, suggests that the reduction in tumor growth is likely due to specific inhibition of the OSM signaling rather than general toxicity.

To evaluate the effectiveness of **SMI-10B13** against survivability and metastasis, we performed a related but separate experiment. As before, mice were treated with either vehicle or **SMI-10B13** 12 h prior to tumor cell injection and monitored for tumor growth while receiving treatment 3x weekly (Figure 7A). Mice were sacrificed after losing no more than 20% of their total body weight or displayed significant pain or discomfort until the end point of the experiment. Over this period, mice treated with vehicle had a larger tumor volume during the first 20 days and were frequently sacrificed earlier than mice treated with **SMI-10B13** (Figure 7B). After 50 days, all mice were sacrificed, and metastases were evaluated with *ex vivo* imaging. From this, we discovered that there was an increase in lung metastases fluorescent intensity in untreated versus treated mice (9.33 × 10^5^ intensity and 41.9 × 10^5^ intensity, respectively; Figure 7C,D). Although the reduction in intensity was not statistically significant, an obvious trend exists between vehicle-treated and **SMI-10B13**-treated animals, suggesting **SMI-10B13**, in addition to reducing tumor burden, may also prevent metastasis for breast cancer patients and increase survival (Figure 7B,D). Thus, for the first time, we have shown that an anti-OSM small molecule inhibitor may inhibit breast cancer progression and metastasis. While our lead compound, **SMI-10B13**, shows promise for further development, structural optimization, and pharmacokinetic/pharmacodynamic validation, an anti-OSM therapeutic may lead to increased survival in breast cancer patients with high OSM expression.

**Figure 7:**
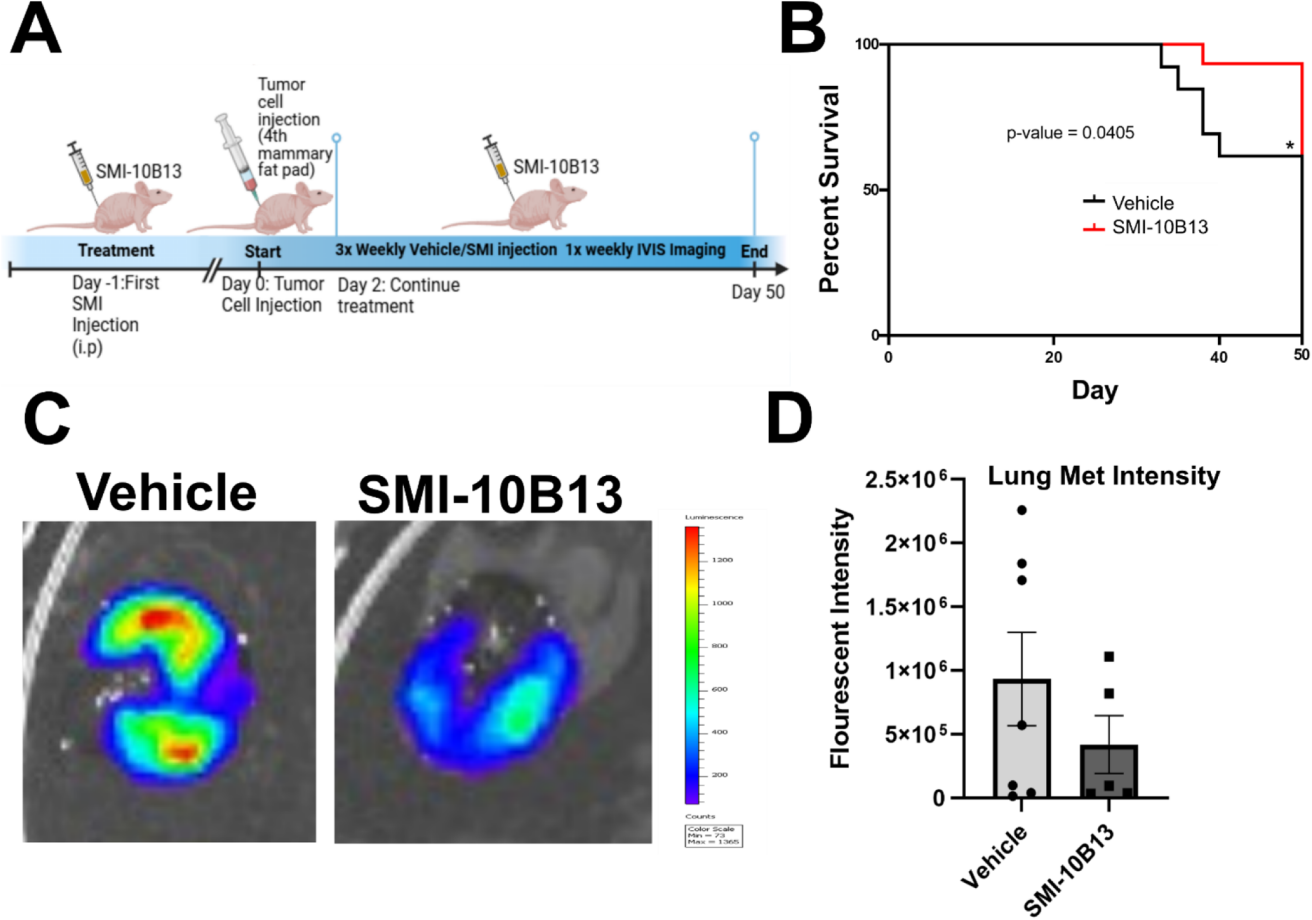
SMI-10B13 inhibits metastasis *in vivo.* (A) 12 h prior to cell injection, athymic nu/nu mice were injected with **SMI-10B13** (*n* = 13) or vehicle control (*n* = 15). The following day, mice were injected with 2 × 10^6^ MCF-7-luc-OSM-overexpressing breast tumor cells in the 4th mammary fat pad and subsequently injected 3x weekly with treatment or vehicle for a period of 50 days. Tumor growth was monitored via caliper measurement, and once weekly, mice were imaged for bioluminescent tumor detection via IVIS imaging. (B) To assess survivability, mice were monitored for weight and tumor size. Mice that reached a clinical end point (weight loss greater than 20% or tumor size greater than 2 cm) were immediately sacrificed, and *ex vivo* imaging was performed for detection of metastasis. After 50 days, all mice were sacrificed. At the end point of the experiment, mice given **SMI-10B13** compared to vehicle control displayed prolonged survival and overall smaller tumors than those treated with vehicle. (C,D) *Ex vivo* imaging revealed that mice treated with **SMI-10B13** displayed a trend toward significance in decreasing lung metastasis intensity compared to mice treated with vehicle control.

## Conclusion

We have identified a novel anti-OSM therapeutic that binds to and inhibits the OSM both *in vitro* and *in vivo*. Identification of our lead compound was accomplished via a high-throughput virtual screening using the crystal structure of OSM followed by biological screening for inhibition of OSM signaling pathways via ELISA and immunoblot analysis. Synthetic optimization and subsequent biological evaluation led to the identification of a small molecule, **SMI-10B13**, that has a binding affinity value (*K*_D_) as low as 7 μM and demonstrated IC_50_ values of 136 and 164 nM in suppressing OSM-mediated STAT3 phosphorylation in T47D and MCF-7 human breast cancer cell lines, respectively. Furthermore, **SMI-10B13** was found to significantly inhibit OSM-mediated tumor growth and increase survival, with a trend toward significance in decreasing metastasis that needs to be validated in future studies. **SMI-10B13** is predicted to have limitations in its pharmacokinetics and safety profiles, which include solubility challenges and the potential inhibition of cytochrome P450 enzymes. Nevertheless, this study represents a proof-of-concept demonstration that direct pharmacological inhibition of OSM with SMIs is feasible and pharmacologically relevant. Further structural optimization of **SMI-10B13’s** scaffold is expected to produce an even more efficacious and drug-like inhibitor of OSM that can be used clinically to treat patients with OSM-related diseases, including those with breast cancer.

## Experimental Section

### High-Throughput Virtual Screening

To identify potential SMI candidates, a *de novo* computational screening approach was employed. First, using the human OSM crystal structure (PDB ID: 1EVS), the AutoLigand program was used to scan the protein surface to search for clefts and pockets to serve as potential SMI binding sites.^57^ The calculations, using ligand shape matching and 3-dimensional templates, revealed three regions on the OSM surface with favorable energy-to-volume ratios and, thus, the potential to serve as SMI binding sites. A structural alignment of OSM and the LIF-LIFR complex (PDB ID: 2Q7N) indicated that one of these sites corresponded to the so-called OSM site III, which is responsible for OSM-OSMRβ interactions. Next, structures deposited into the National Cancer Institute Diversity Set III and ZINC databases were queried against site III using the OpenEye Scientific program.^58^ The AutoDock Vina and AutoDock 4.2 programs were used to predict SMI-OSM site III binding constants and free energies,^50,51^ and all compounds with predicted binding constants of less than 10 μM and/or binding free energies more negative than −5.0 kcal/mol were prioritized for further experiments.

### General Methods for Synthesis, Characterization, and Purity Determination

All commercially available chemicals were purchased from Sigma-Aldrich, TCI, CombiBlocks, or Thermo Fisher Scientific and used without further purification. Reactions were conducted in oven-dried glassware, unless H_2_O was present as a solvent or reactant. Commercially obtained anhydrous solvents were stored over 3Å Linde-type molecular sieves (ca. 20% v/v). THF was regularly distilled from Na/benzophenone. Reactions were run under an inert N_2_ atmosphere unless indicated otherwise. Reaction progress was monitored by thin-layer chromatography (TLC) (glass-backed, 250 μm silica gel) in the same eluent as reported for corresponding column chromatography purification, or a 3:1 EtOAc and hexanes mixture for reactions producing a carboxylic acid. UV light, KMnO_4_ plus heat, 2,4-dinitrophenylhydrazine (DNPH), or ninhydrin-based stains were used for visualization. All instances of flash chromatography purification used 60 Å, 230–400 mesh silica gel. ^1^H and ^13^C NMR spectra were recorded at 298 K (25 °C) on a Bruker Avance III 600 MHz equipped with a 5 mm TCI cryoprobe with *z*-axis gradients or on a Bruker Avance III 300 MHz instrument, as specified below in each spectrum. Compounds were dissolved in deuterated chloroform (CDCl_3_) or deuterated DMSO (DMSO-d6) solvents stored over 3Å Linde-type molecular sieves. The residual CHCl_3_ signal at δ­(H) = 7.26 ppm and the central peak of the CDCl_3_ triplet appearing at δ­(C) = 77.16 ppm were used to reference the spectra obtained in CDCl_3_. The central peak of the DMSO-d_5_ signal pentet at δ­(H) = 2.50 ppm and the central signal of the septet at δ­(C) = 39.52 ppm were used for referencing spectra obtained in DMSO-d_6_. All spectra were processed on MestreNova 14.2.0 with multiplicity denoted as s = singlet; d = doublet; t = triplet; q = quartet; sept = septet; m = multiplet. HRMS spectra were recorded on a Bruker Daltonics maXis Quadrupole Time-of-Flight (Q-ToF) Mass Spectrometer using positive ionization mode, unless otherwise specified.

All compounds are >95% pure by HPLC analysis. An Agilent 1100 series HPLC instrument equipped with a Phenomenex Synergy Fusion reverse-phase C18 column with polar end-capping (4 μm Hydro-RP 80 Å, 250 × 4.60 mm) and a UV/vis detector was used. The UV detection was set to either 254 or 280 nm. Samples were dissolved in DMSO or THF (∼1 mg/mL), and the DMSO peak (see DMSO blank trace) was excluded from the integration when present, or it was subtracted from the trace using the blank trace function. The purity percentage of each compound was obtained from the % area of the UV trace at the scanning wavelength. The elution solvent consisted of 95% acetonitrile and 5% H_2_O (1% formic acid/H_2_O solution). The flow rate was 1.000 mL/min, the column temperature was 32 °C, and the injection volume was 5.0 μL unless specified otherwise. For compounds **SMI-10F** and **SMI-10C**, the elution solvent was 95% isopropanol and 5% H_2_O (with 0.001 M NaN_3_), with a flow rate of 1.430 mL/min, a column temperature of 55 °C, and an injection volume of 3.0 μL. For compounds **SMI-10B** and **SMI-10B1** through **SMI-10B12**, the elution solvent 70% CH_3_CN and 30% H_2_O (1% HCO_2_H/H_2_O solution), with a flow rate of 1.500 mL/min, a column temperature of 27 °C, and a 3.0 μL injection volume was used.

### General Synthetic Procedures

#### General Procedure for Suzuki–Miyaura Coupling of 4,5-Dibromoheteroaryl-2-carbaldehydes

To a degassed solution of 5:1 v/v DMF/H_2_O, 4,5-dibromoheteroaryl-2-carbaldehyde (1.0 equiv), arylboronic acid (2.2 equiv), Na_2_CO_3_ (5.0 equiv), and Pd­(PPh_3_)_4_ (0.1 equiv) were added and heated to 90 °C under an N_2_ atmosphere. Reaction progress was monitored by TLC (hexanes/EtOAc elution), and upon disappearance of starting material and monocoupled intermediate, the reaction mixture was cooled to RT and diluted in 50 mL of EtOAc. The mixture was washed with 3 × 50 mL sat. NaHCO_3_ and 1 × 50 mL brine, dried over MgSO_4_, filtered, and concentrated under reduced pressure to afford a brown crude residue. The residue was purified by trituration in hexanes/EtOAc when necessary, and a fully coupled aldehyde intermediate was obtained.

#### General Procedure for Doebner–Knoevenagel Reaction

A solution of aldehyde intermediate (1.0 equiv), malonic acid (10 equiv), and a catalytic amount of piperidine in pyridine was heated to reflux (120 °C) under an N_2_ atmosphere. The reaction was maintained under reflux until the evolution of CO_2_ gas ceased (ca. 4–7 h), after which the mixture was cooled to 0 °C and poured into ice-cold 6 M HCl. The precipitate was collected by filtration and washed with 3 × 50 mL of DI H_2_O to afford the α,β-unsaturated acid. As necessary, analogs were purified by flash column chromatography (silica, hexanes/EtOAc elution), and concentration afforded the propenoic acid

### Synthesis of **SMI-10** Analogs (Scheme 2, **SMI-10A**–**10J**)

#### Methyl 4,5-Dibromofuran-2-carboxylate (**1**)

4,5-Dibromo-2-furoic acid (300.0 mg, 1.11 mmol) was dissolved in 2.8 mL CH_3_OH. To the resulting solution, concentrated H_2_SO_4_ (63 μL, 1.22 mmol, 1.1 equiv) was added dropwise, and the mixture was allowed to reflux at 80 °C under an N_2_ atmosphere for 42 h. The solution was concentrated under reduced pressure to remove excess CH_3_OH and quenched with 15 mL of saturated NaHCO_3_ solution until a basic pH was obtained. The aqueous phase was extracted with 2 × 20 mL of EtOAc. The organic layers were combined, washed with 20 mL of DI H_2_O, dried with MgSO_4_, filtered, and concentrated to yield a white powder. The resulting product was carried forward without further purification (226.7 mg, 72% yield).

#### Methyl 4,5-Bis­(benzo­[d]­[1,3]­dioxol-5-yl)­furan-2-carboxylate (**SMI-10A**)

Compound **1** (0.5000 g, 1.76 mmol), 3,4-methylenedioxyphenylboronic acid (642.9 mg, 3.87 mmol, 2.2 equiv), Cs_2_CO_3_ (3.441 g, 10.6 mmol, 6.0 equiv), AsPh_3_ (107.8 mg, 0.352 mmol, 0.2 equiv), and (Ph_3_P)_2_PdCl_2_ (197.7 mg, 0.28 mmol, 0.16 equiv) were combined in a flask that had been evacuated and refilled with N_2_ (5 cycles). The resulting mixture was dissolved in 12 mL of dry, distilled DMF and refluxed at 90 °C under an argon atmosphere for 113 h. The reaction was concentrated under reduced pressure to remove excess DMF. The resulting residue was dissolved in 50 mL of EtOAc and washed with 3 × 50 mL saturated NaHCO_3_ solution. The aqueous layers were extracted with 3 × 30 mL of EtOAc. The organic layers were combined, dried with MgSO_4_, filtered, and concentrated to yield a yellow/brown oil. The crude product was applied to a 6 in. (6 cm diameter) silica column and eluted with 3:1 hexanes/EtOAc to yield 331.3 mg of pure product (51% yield). ^1^H NMR (600 MHz, CDCl_3_ with 0.05% v/v TMS) δ 7.22 (s, 1H), 7.14 (dd, *J* = 8.2, 1.7 Hz, 1H), 7.05 (d, *J* = 1.8 Hz, 1H), 6.84 – 6.81 (m, 3H), 6.77 (d, *J* = 8.2 Hz, 1H), 6.00 (s, 2H), 5.97 (s, 2H), 3.91 (s, 3H). ^13^C NMR (151 MHz, CDCl_3_ with 0.05% v/v TMS) δ: 159.36, 152.06, 148.30, 148.12, 147.87, 147.40, 142.31, 126.72, 123.92, 123.11, 122.40, 121.85, 121.74, 109.26, 108.90, 108.67, 107.57, 101.45, 101.37, 52.05. HRMS *m*/*z*: [2 M + Na]^+^ Calcd for C_20_H_14_O_7_ 755.1371; Found 755.1376.

#### 4,5-Dibromofuran-2-carbaldehyde (**2a**)

Synthesis adapted from the procedure reported by Chiarello et al.,^59^ with modifications to the purification process. To a round-bottom flask under N_2_, AlCl_3_ (4 equiv) was added together with anhydrous MeCl_2_ (9 volumes relative to furfural). Furfural was then added via cannula dropwise over the course of 30 min. Br_2_ (1.5 volumes relative to furfural) was then added dropwise through an addition funnel over the course of 30 min. After 1 h, the ice bath was removed, and the reaction was stirred at room temperature overnight. The reaction mixture was quenched with a saturated solution of Na_2_S_2_O_3_ and extracted three times in Et_2_O. The organic layer was dried with MgSO_4_, filtered, and concentrated to yield a viscous brown liquid. The crude product was applied to a 7 in. silica column and eluted with 6:1 hexanes/EtOAc. The resulting product was a brown, thick liquid (5–34% yield) that was used immediately to minimize decomposition.

#### 4,5-Bis­(benzo­[d]­[1,3]­dioxol-5-yl)­furan-2-carbaldehyde (**3**)

Aldehyde **2a** (0.990 g, 3.90 mmol) was dissolved in 26 mL of dry, distilled DMF, into which 3,4-methylenedioxyphenyl boronic acid (1.424 g, 8.58 mmol, 2.2 equiv), Cs_2_CO_3_ (7.624 g, 23.4 mmol, 6 equiv), AsPh_3_ (0.239 g, 0.78 mmol, 0.2 equiv), and (Ph_3_P)_2_PdCl_2_ (0.382 g, 0.54 mmol, 0.14 equiv) were added under N_2_ atmosphere. The resulting brown solution was allowed to reflux at 90 °C for 23 h and then concentrated under reduced pressure to remove the excess DMF. The remaining residue was dissolved in 100 mL of EtOAc and washed with 3 × 50 mL of saturated NaHCO_3_ solution. The organic layer was dried with MgSO_4_, filtered, and concentrated to yield a brown solid. The crude product was applied to a 7 in. (5 cm diameter) silica column and eluted with 3:1 hexanes:EtOAc. Fractions containing the pure product, as determined by TLC, were combined and concentrated to yield 1.003 g of an orange solid (76% yield).

#### (E)-3-(4,5-Bis­(benzo­[d]­[1,3]­dioxol-5-yl)­furan-2-yl)­acrylic acid (**SMI-10B**)

Synthesized according to the general Doebner–Knoevenagel procedure with aldehyde intermediate **Compound 3**. Orange solid, 78% yield. ^1^H NMR (600 MHz, DMSO) δ 12.43 (s, 1H), 7.38 (d, *J* = 15.7 Hz, 1H), 7.06 (d, *J* = 9.8 Hz, 3H), 6.95 (dd, *J* = 15.4, 8.0 Hz, 2H), 6.91 (s, 1H), 6.85 (d, *J* = 8.0 Hz, 1H), 6.32 (d, *J* = 15.7 Hz, 1H), 6.06 (s, 2H), and 6.06 (s, 2H). ^13^C NMR (151 MHz, DMSO) δ: 167.45, 149.39, 148.54, 147.63, 147.54, 146.86, 130.18, 126.41, 123.61, 123.51, 121.99, 120.70, 119.36, 116.37 (m, 2C), 108.78, 108.74, 108.71, 106.35, 101.46, and 101.25. HRMS *m*/*z*: [M + H]^+^ Calcd for C_21_H_14_O_7_ 379.0812; Found 379.0815.

#### (E)-3-(4,5-Bis­(benzo­[d]­[1,3]­dioxol-5-yl)­furan-2-yl-1-phenylprop-2-en-1-one (**SMI-10C**)

Sodium hydride (6.7 mg, 0.28 mmol, 1.3 equiv), dissolved in 1 mL of tetrahydrofuran, was added dropwise to diethyl (2-oxo-2-phenylethyl)­phosphonate (67 μL, 0.31 mmol, 1.4 equiv), cooled to 0 °C and stirred for 10 min. Compound **3** (74 mg, 0.22 mmol) was then added and allowed to stir at 0 °C for 30 min. The ice bath was removed, and the mixture was stirred at rt for 1.5 h. The resulting solution was diluted with 10 mL of Et_2_O and washed with 15 mL of saturated NaHCO_3_ solution and 15 mL of brine. The organic layer was dried with MgSO_4_, filtered, and concentrated to yield an orange solid. The crude product was applied to a 6 in. (2 cm diameter) silica column and eluted with 3:1 hexanes:EtOAc to yield 72.0 mg of pure yellow solid (75% yield). ^1^H NMR (600 MHz, CDCl_3_ with 0.05% v/v TMS) δ 8.08–8.04 (m, 2H), 7.63–7.56 (m, 2H), 7.55–7.47 (m, 3H), 7.15 (dd, *J* = 8.2, 1.8 Hz, 1H), 7.09 (d, *J* = 1.8 Hz, 1H), 6.91–6.81 (m, 3H), 6.80 (d, *J* = 8.7 Hz, 2H), 6.01 (s, 2H), and 6.00 (s, 2H). ^13^C NMR (151 MHz, CDCl_3_ with 0.05% v/v TMS) δ: 189.86, 150.87, 149.77, 148.10, 147.90, 147.35, 138.50–138.38 (m, 2C), 132.88, 130.42, 128.77, 128.58, 127.01, 124.47, 124.32, 122.36, 121.37, 120.92, 118.87, 109.26, 108.91, 108.75, 107.23, 101.47, and 101.37. HRMS *m*/*z*: [M + H]^+^ Calcd for C_27_H_18_O_6_ 439.1176; Found 439.1171.

#### Ethyl (E)-3-(4,5-Bis­(benzo­[d]­[1,3]­dioxol-5-yl)­furan-2-yl)­acrylate (**SMI-10D**)

Sodium hydride (15 mg, 0.63 mmol, 1.4 equiv) dissolved in 5 mL of dry ethanol was allowed to stir at 0 °C for 10 min. Triethyl phosphonoacetate (122 μL, 0.61 mmol, 1.4 equiv) and compound **3** (148 mg, 0.44 mmol) were added to the solution and allowed to stir at rt for 167 h until the disappearance of starting material, as seen by TLC. The reaction was quenched with the dropwise addition of 3 mL saturated ammonium chloride solution until a precipitate formed. The resulting reaction mixture was diluted with 15 mL of H_2_O and extracted with 3x 10 mL of CH_2_Cl_2_. The organic layers were combined, dried with MgSO_4_, filtered, and concentrated to yield a dark yellow oil. The crude product was applied to a 6 in. (4 cm diameter) silica column and eluted with 5:1 hexanes/Et_2_O to yield 71.9 mg of pure yellow oil (40% yield). ^1^H NMR (600 MHz, CDCl_3_ with 0.05% v/v TMS) δ 7.42 (d, *J* = 15.6 Hz, 1H), 7.10 (dd, *J* = 8.2, 1.7 Hz, 1H), 7.02 (d, *J* = 1.7 Hz, 1H), 6.86 −6.80 (m, 3H), 6.76 (d, *J* = 8.2 Hz, 1H), 6.65 (s, 1H), 6.38 (d, *J* = 15.6 Hz, 1H), 6.00 (s, 2H), 5.97 (s, 2H), 4.26 (q, *J* = 7.2 Hz, 2H), and 1.34 (t, *J* = 7.1 Hz, 3H). ^13^C NMR (151 MHz, CDCl_3_ with 0.05% v/v TMS) δ: 167.28, 150.42, 149.00, 148.09, 147.94, 147.87, 147.30, 130.67, 127.19, 124.41, 123.91, 122.35, 121.11, 119.17, 115.67, 109.28, 108.88, 108.68, 107.09, 101.41, 101.34, 60.60, and 14.49. HRMS *m*/*z*: [M + H]^+^ Calcd for C_23_H_18_O_7_ 407.1125; Found 407.1120.

#### (E)-1-((4,5-Bis­(benzo­[d]­[1,3]­dioxol-5-yl)­furan-2-yl)­methylene)-2-phenylhydrazine (**SMI-10E**)

Compound **3** (73.2 mg, 0.22 mmol) was dissolved in 5 mL of dry chloroform along with 4 Å molecular sieves. To the resulting solution, scandium­(III) triflate (1.1 mg, 0.0022 mmol, 0.01 equiv) was added and allowed to stir at rt. After 5 min, PhNHNH_2_ (43 μL, 0.44 mmol, 2 equiv) was added and allowed to stir for 15 h. The resulting solution was filtered through Celite, washed with 5 mL of CHCl_3_, and concentrated to yield a red oil. The crude product was applied to a 6-in. (2 cm diameter) silica column eluted with 6:1 hexanes/EtOAc to yield 51.7 mg of pure product (55% yield). ^1^H NMR (600 MHz, CDCl_3_ with 0.05% v/v TMS) δ 7.63 (s, 1H), 7.57 (s, 1H), 7.34–7.22 (m, 2H), 7.11 (m, 3H), 7.06 (d, *J* = 1.7 Hz, 1H), 6.94–6.85 (m, 3H), 6.88–6.79 (m, 1H), 6.77 (d, *J* = 8.2 Hz, 1H), 6.65 (s, 1H), 6.00 (s, 2H), and 5.97 (s, 2H). ^13^C NMR (151 MHz, CDCl_3_ with 0.05% v/v TMS) δ: 148.83, 148.54, 147.96, 147.72, 147.72, 147.36, 147.05, 144.38, 129.43, 127.72, 127.38, 124.93, 123.27, 122.29, 120.75, 120.42, 113.18, 112.92, 109.30, 108.78, 108.60, 107.04, and 101.25. HRMS *m*/*z*: [M + H]^+^ Calcd for C_25_H_18_N_2_O_5_ O7 427.1288; Found 427.129.

#### *N*-Benzyl-1-(4,5-bis­(benzo­[d]­[1,3]­dioxol-5-yl)­furan-2-yl)­methanamine (**SMI-10F**)

Compound **3** (60 mg, 0.18 mmol) was dissolved in 2 mL of benzene. To this solution, freshly distilled benzylamine (22 μL, 0.20 mmol, 1.1 equiv) was added and allowed to reflux at 85 °C under a N_2_ atmosphere for 4 h. The reaction was then concentrated under reduced pressure to remove excess benzene, and the resulting residue was dissolved in 1 mL MeOH. NaBH_4_ (17 mg, 0.27 mmol, 1.5 equiv) and CF_3_CO_2_H (15 μL, 0.20 mmol, 1.1 equiv) were added to the reaction and allowed to stir at 0 °C. After 30 min, the reaction was allowed to warm to rt and stirred for 1 h. The reaction mixture was then concentrated under reduced pressure to remove excess MeOH, and the resulting residue was then dissolved in 20 mL of EtOAc. The solution was washed with 15 mL of 1 M NaOH and then 15 mL of brine. The organic layer was dried with MgSO_4_, filtered, and concentrated to yield a red-orange oil. The crude product was applied to a 6 in. (4 cm diameter) silica column and eluted with 1:1 hexanes/EtOAc to yield 64.2 mg of pure yellow oil (83% yield). ^1^H NMR (600 MHz, CDCl_3_ with 0.05% v/v TMS) δ 7.37–7.29 (m, 4H), 7.27–7.21 (m, 1H), 7.02 (d, *J* = 8.2 Hz, 1H), 6.98 (s, 1H), 6.86 – 6.82 (m, 2H), 6.78 (d, *J* = 7.9 Hz, 1H), 6.72 (d, *J* = 8.2 Hz, 1H), 6.26 (s, 1H), 5.93 (s, 2H), 5.90 (s, 2H), 3.85 (s, 2H), 3.81 (s, 2H), and 1.76 (s, 1H). ^13^C NMR (151 MHz, CDCl_3_ with 0.05% v/v TMS) δ: 128.47, 123.88, 123.67, 123.39, 123.02, 122.77, 116.04, 104.56, 104.40, 104.24, 103.18, 101.43, 98.11, 97.66, 96.44, 87.47, 85.20, 84.70, 84.51, 83.03, 77.16, 29.07, and 21.56. HRMS *m*/*z*: [M + H]^+^ Calcd for C_26_H_21_NO_5_ 428.1492; Found 428.1491.

#### 4,5-Dibromo-*N*-methoxy-*N*-methylfuran-2-carboxamide (**4**)

4,5-Dibromo-2-furoic acid (996 mg, 3.69 mmol) was dissolved in 25 mL CH_2_Cl_2_, into which were added N,O-dimethylhydroxylamine hydrochloride (414 mg, 4.24 mmol, 1.15 equiv), NEt_3_ (0.56 mL, 4.06 mmol, 1.1 equiv), 1,3-dicyclohexylcarbodiimide (761 mg, 3.69 mmol, 1 equiv), and 4-dimethylaminopyridine (235 mg, 1.92 mmol, 0.5 equiv). The reaction mixture was allowed to stir for 24 h. The solid precipitate that formed was collected through gravity filtration, washed with CH_2_Cl_2_, and concentrated under reduced pressure. The resulting residue was dissolved in 30 mL of EtOAc and washed with 25 mL of brine, 25 mL of saturated NaHCO_3_ solution, and 25 mL of H_2_O. The organic layer was dried with Na_2_SO_4_, filtered, and concentrated to yield a white solid. The crude product was applied to a 6-in. (4.5 cm diameter) silica column and eluted with a gradient of 3:1 hexanes/EtOAc (1200 mL), followed by 1:1 hexanes/EtOAc (600 mL), to yield 663 mg of pure product (57% yield).

#### 4,5-Bis­(benzo­[d]­[1,3]­dioxol-5-yl)-*N*-methoxy-*N*-methylfuran-2-carboxamide (**4a**)

Compound **4** (313 mg, 1.0 mmol) was dissolved in 7.6 mL of dry, distilled DMF. To the resulting solution were added 3,4-methylenedioxyphenylboronic acid (365 mg, 2.2 mmol, 2.2 equiv), Cs_2_CO_3_ (1.95 g, 6 mmol, 6 equiv), AsPh_3_ (61 mg, 0.2 mmol, 0.2 equiv), and bis­(triphenylphosphine)­palladium­(II) dichloride (112 mg, 0.16 mmol, 0.16 equiv), and the mixture was allowed to reflux at 90 °C under a N_2_ atmosphere for 44 h. The reaction mixture was concentrated under reduced pressure to remove excess DMF. The resulting residue was dissolved in 100 mL of EtOAc and washed three times with 50 mL of 25% NaHCO_3_ solution. The organic layer was dried with MgSO_4_, filtered, and concentrated to yield a brown solid. The crude product was applied to an 8 in. (4 cm diameter) silica column and eluted with a gradient of 1:1 hexanes/EtOAc (600 mL), followed by 3:1 hexanes/EtOAc (1500 mL), to yield 297.8 mg of pure yellow solid product (75% yield).

#### (E)-1-(4,5-Bis­(benzo­[d]­[1,3]­dioxol-5-yl)­furan-2-yl)-3-phenylprop-2-en-1-one (**SMI-10G**)

β-Bromostyrene (0.97 mL, 7.59 mmol, 50 equiv)—dried via passage through a 1.5-in. (1 cm diameter) column containing Na_2_SO_4_ and neutral alumina—was dissolved in 7 mL of dry THF, and the reaction flask was purged with N_2_. The resulting solution was transferred via cannula to a second flask, also purged with N_2_, containing 3 crystals of iodine and magnesium (199 mg, 8.19 mmol, 55 equiv) that had been crushed with a mortar and pestle. The empty, original flask was rinsed with 3 mL of dry THF and added to the flask containing magnesium and iodine. The resulting solution was refluxed at 50 °C under a N_2_ atmosphere for 3 h to synthesize the Grignard reagent. Compound **4a** (60 mg, 0.15 mmol) was dissolved in 3 mL of the newly synthesized Grignard reagent and allowed to stir at rt under a N_2_ atmosphere for 1.5 h. The reaction was quenched with 10 mL of saturated NH_4_Cl solution. The reaction mixture was diluted with 5 mL of H_2_O and extracted with 2x 10 mL of Et_2_O. The organic layers were combined and washed with 10 mL of saturated NaHCO_3_ solution, 10 mL of 1 M hydrochloric acid, and 10 mL of brine. The organic layers were then dried with MgSO_4_, filtered, and concentrated to yield a bright yellow oil. The crude product was applied to a 6-in. (5 cm diameter) silica column and eluted with 3:1 hexanes/EtOAc to yield 45.4 mg of pure yellow oil (69% yield). ^1^H NMR (600 MHz, CDCl_3_ with 0.05% v/v TMS) δ 7.90 (d, *J* = 15.7 Hz, 1H), 7.71–7.65 (m, 2H), 7.49 (d, *J* = 15.8 Hz, 1H), 7.47–7.39 (m, 3H), 7.38 (s, 1H), 7.21 (dd, *J* = 8.1, 1.7 Hz, 1H), 7.12 (d, *J* = 1.8 Hz, 1H), 6.89 (dd, *J* = 7.7, 1.8 Hz, 1H), 6.85 (d, *J* = 8.0 Hz, 2H), 6.81 (d, *J* = 8.2 Hz, 1H), 6.01 (s, 2H), and 6.00 (s, 2H). ^13^C NMR (151 MHz, CDCl_3_ with 0.05% v/v TMS) δ: 177.58, 152.48, 151.50, 148.56, 148.17, 147.95, 147.52, 143.78, 135.00, 130.69, 129.09, 128.69, 126.67, 124.19, 123.88, 122.48, 121.98, 121.68, 121.37, 109.30, 108.95, 108.77, 107.58, 101.54, and 101.41. HRMS *m*/*z*: [M + H]^+^ Calcd for C_27_H_18_O_6_ 439.1176; Found 439.1194.

#### (4,5-Bis­(benzo­[d]­[1,3]­dioxol-5-yl)­furan-2-yl)­methanol (**SMI-10H**)

Compound **3** (250 mg, 0.74 mmol) was dissolved in 20 mL of MeOH and cooled to 0 °C in an ice bath. NaBH_4_ (33.8 mg, 0.89 mmol, 1.2 equiv) was added portion-wise over 15 min. The resulting solution was then allowed to stir at rt for 1 h. The solution was again cooled to 0 °C, and additional NaBH_4_ (33.8 mg, 0.89 mmol, 1.2 equiv) was added portion-wise over 15 min. After the resulting solution was stirred at rt for another 1 h, the solution was cooled to 0 °C, and a final equivalent of NaBH_4_ (33.8 mg, 0.89 mmol, 1.2 equiv) was added portion-wise over 15 min. The resulting solution was stirred at rt for another 69 h, at which time 1 mL of H_2_O was added and stirred for 30 min. The resulting solution was concentrated under reduced pressure to remove excess MeOH and diluted with 30 mL of 10% 1 M HCl in H_2_O. The reaction mixture was then extracted with 3x 25 mL of EtOAc, and the organic layers were combined, dried with MgSO_4_, filtered, and concentrated to yield a brown oil. The crude product was applied to a 6-in. (4 cm diameter) silica column and eluted with 1:1 hexanes/EtOAc to yield 248.0 mg of pure yellow oil (99% yield). ^1^H NMR (600 MHz, CDCl_3_ with 0.05% v/v TMS) δ 7.04 (dd, *J* = 8.2, 1.8 Hz, 1H), 6.98 (d, *J* = 1.8 Hz, 1H), 6.86–6.80 (m, 2H), 6.80 (d, *J* = 7.9 Hz, 1H), 6.75 (d, *J* = 8.2 Hz, 1H), 6.38 (s, 1H), 5.98 (s, 2H), 5.95 (s, 2H), 4.65 (s, 2H), and 1.90 (s, 1H). ^13^C NMR (151 MHz, CDCl_3_ with 0.05% v/v TMS) δ: 152.36, 148.23, 147.93, 147.72, 147.27, 146.91, 127.97, 125.15, 122.19, 121.81, 120.66, 112.29, 109.22, 108.75, 108.56, 107.13, 101.22, 101.20, and 57.72. HRMS *m*/*z*: [M + Na]^+^ Calcd for C_19_H_14_O_6_ 361.0683; Found 361.0683.

#### 4,5-Bis­(benzo­[d]­[1,3]­dioxol-5-yl)­furan-2-carboxylic Acid (**SMI-10I**)

**SMI-10A** (180 mg, 0.49 mmol) was dissolved in 4.6 mL of THF and 1.4 mL H_2_O into which LiOH (117 mg, 4.9 mmol, 10 equiv) was added and allowed to reflux at 70 °C for 3 h. The reaction mixture was diluted with 5 mL of H_2_O and quenched with 6 mL of 1 M hydrochloric acid solution until an acidic pH was obtained. The resulting solution was extracted with 3x 15 mL of EtOAc. The organic layers were combined, washed with 2x 20 mL of H_2_O, dried with MgSO_4_, filtered, and concentrated to yield 138.8 mg of a pale-yellow solid that was used without further purification (80% yield). ^1^H NMR (600 MHz, DMSO) δ 13.17 (s, 1H), 7.36 (d, *J* = 1.0 Hz, 1H), 7.03 (dd, *J* = 8.2, 1.7 Hz, 1H), 7.00–6.93 (m, 4H), 6.86 (dd, *J* = 8.1, 1.7 Hz, 1H), 6.07 (s, 2H), and 6.06 (s, 2H). ^13^C NMR (151 MHz, DMSO) δ: 159.30, 150.69, 147.98, 147.64, 147.56, 146.92, 142.87, 125.94, 123.34, 122.76, 122.10, 121.28, 120.98, 108.85, 108.81, 108.77, 106.69, 101.60, and 101.27. HRMS *m*/*z*: [M + H]^+^ Calcd for C_19_H_12_O_7_ 353.0656; Found 353.0653.

#### 4,5-Bis­(benzo­[d]­[1,3]­dioxol-5-yl)-*N*-benzylfuran-2-carboxamide (**SMI-10J**)

Compound **SMI-10I** (50 mg, 0.14 mmol) was dissolved in 1 mL CH_2_Cl_2_ and 5 drops of DMF, into which SOCl_2_ (21 μL, 0.28 mmol, 2 equiv) was added, and the mixture was allowed to reflux at 60 °C for 4 h. The resulting solution was concentrated under reduced pressure to remove the excess CH_2_Cl_2_. The resulting residue was dissolved in 1 mL Et_2_O. To the reaction mixture were added benzylamine (31 μL, 0.29 mmol, 2.1 equiv) and NEt_3_ (39 μL, 0.28 mmol, 2 equiv) and the mixture was allowed to stir at rt under a N_2_ atmosphere for 2 h. The solution was diluted with 10 mL of H_2_O and extracted with 3x 10 mL of Et_2_O. The organic layers were combined, dried with MgSO_4_, filtered, and concentrated. The crude product was applied to a 7-in. (2 cm diameter) silica column and eluted with 3:2 hexanes/EtOAc to yield 48.2 mg of pure product (78% yield). ^1^H NMR (600 MHz, CDCl_3_) δ 7.41–7.34 (m, 4H), 7.30 (ddt, *J* = 8.6, 5.9, 2.1 Hz, 1H), 7.22 (s, 1H), 7.03 (dd, *J* = 8.2, 1.7 Hz, 1H), 6.96 (d, *J* = 1.7 Hz, 1H), 6.90–6.78 (m, 3H), 6.75 (d, *J* = 8.2 Hz, 1H), 6.67 (t, *J* = 6.0 Hz, 1H), 5.99 (s, 2H), 5.96 (s, 2H), and 4.66 (d, *J* = 6.0 Hz, 2H). ^13^C NMR (151 MHz, CDCl_3_) δ: 167.36, 149.64, 148.04, 147.97, 147.78, 147.31, 146.96, 134.32, 131.83, 128.78, 127.87, 127.17, 125.13, 122.20, 121.97, 120.64, 112.22, 109.21, 108.77, 108.61, 107.12, 101.25, 101.22, and 37.29. HRMS *m*/*z*: [M + H]^+^ Calcd for C_26_H_19_NO_6_ 442.1285; Found 442.1291.

### Synthesis of **SMI-10B** Analogs (Scheme 3A and Scheme 3B, **SMI-10B1**–**B13**)

#### (E)-3-(4,5-Bis­(3,4-dimethoxyphenyl)­furan-2-yl)­acrylic Acid (**SMI-10B1**)

The intermediate 4,5-bis­(3,4-dimethoxyphenyl)­furan-2-carbaldehyde was prepared according to the general Suzuki-Miyaura coupling procedure using 4,5-dibromofuran-2-carbaldehyde (**2a**) and 3,4-dimethoxyphenylboronic acid. The product was via flash column chromatography using a 2:1 hexanes/EtOAc elution (Rf = 0.24). Concentration of the appropriate fractions afforded a fluffy yellow solid (61% yield).

The target compound **SMI-10B1** was synthesized according to the general Doebner–Knoevenagel procedure using the aldehyde intermediate in 66% yield. ^1^H NMR (600 MHz, CDCl_3_) δ 7.39 (s, 0H), 7.15–7.09 (m, 3H), 7.00 (d, *J* = 8.2 Hz, 1H), 6.97 (dd, *J* = 5.3, 3.3 Hz, 2H), 6.94 (dd, *J* = 8.2, 2.0 Hz, 1H), 6.32 (d, *J* = 15.7 Hz, 1H), 3.77 (d, *J* = 5.5 Hz, 6H), 3.69 (s, 3H), and 3.63 (s, 3H). ^13^C NMR (151 MHz, DMSO) δ: 149.64, 149.23, 148.80, 148.53, 148.47, 148.40, 130.25, 125.25, 123.54, 122.41, 120.78, 119.32, 119.19, 116.03, 112.16, 112.06, 111.81, 111.81, 109.65, 55.55, 55.51, 55.48, and 55.27. HRMS *m*/*z*: [M—H]^−^ Calcd for C_23_H_22_O_7_ 409.1282; Found 409.1301.

#### (E)-3-(4,5-Bis­(3-hydroxy-4-methoxyphenyl)­furan-2-yl)­acrylic Acid (**SMI-10B2**)

The intermediate *4,5-bis­(3-hydroxy-4-methoxyphenyl)­furan-2-carbaldehyde* was synthesized according to the general Suzuki–Miyaura procedure, with modifications. A protected acetylated derivative of the boronic acid, *3-acetoxy-4-methoxy-phenylboronic acid pinacol ester*, was used in the reaction. The reaction mixture was heated at reflux in 1,4-dioxane for 40 h, after which the mixture was cooled to room temperature and subjected to standard aqueous workup. Flash column chromatography using a 1:1 hexanes/EtOAc elution (Rf = 0.20) afforded 104.1 mg of a mixture of deacetylated and acetylated products. Following a procedure originally reported by Narender et al.,^60^ the mixture (99 mg, ca. 0.29 mmol) was taken up in EtOH (3 mL) and H_2_O (0.3 mL), and NaOAc (237.8 mg, 2.9 mmol) was added. The mixture was stirred and heated to reflux overnight, after which the mixture was diluted with 5 mL of H_2_O and extracted 2× 10 mL of EtOAc. The organic layers were combined, dried over anhydrous Na_2_SO_4_, filtered, and concentrated to afford an orange crude residue. The residue was recrystallized from CH_2_Cl_2_/hexanes to afford 83 mg of a crystalline orange solid (13% yield, two steps).

The target **SMI-10B2** was synthesized according to the general Doebner–Knoevenagel reaction procedure for the aldehyde intermediate, with modifications. The acidified reaction mixture was extracted with 2× 20 mL of EtOAc, and the organic layers were combined, dried over anhydrous Na_2_SO_4_, filtered, and concentrated to afford an orange crude residue. The crude residue was subjected to flash column chromatography (7% MeOH in EtOAc, Rf = 0.32), and the appropriate fractions were collected, concentrated under reduced pressure, and recrystallized from EtOAc/hexanes to afford a yellow, crystalline solid (65% yield). ^1^H NMR (300 MHz, DMSO) δ 12.35 (s, 1H), 9.14 (d, *J* = 21.4 Hz, 2H), 7.39 (d, *J* = 15.7 Hz, 1H), 7.05–6.87 (m, 5H), 6.81–6.72 (m, 2H), 6.23 (d, *J* = 15.7 Hz, 1H), 3.79 (s, 3H), and 3.77 (s, 3H). ^13^C NMR (151 MHz, DMSO) δ 167.57, 149.95, 148.11, 147.66, 147.45, 147.32, 146.17, 130.47, 123.81, 123.33, 121.17, 121.08, 119.68, 119.54, 115.74, 115.66, 115.43, 112.50, 110.15, 55.58, and 55.36. HRMS *m*/*z*: [M + H]^+^ Calcd for C_21_H_18_O_7_ 383.1125; Found 383.1132.

#### (E)-3-(4,5-Bis­(4-hydroxy-3-methoxyphenyl)­furan-2-yl)­acrylic Acid (**SMI-10B3**)

The intermediate *4,5-bis­(4-hydroxy-3-methoxyphenyl)­furan-2-carbaldehyde* was *s*ynthesized according to the general Suzuki–Miyaura procedure with modifications. A protected derivative of the boronic acid, *4-acetoxy-3-methoxy-phenylboronic acid pinacol ester*, was used in the reaction. The reaction mixture was heated at reflux in 1,4-dioxane for 21 h, after which the mixture was cooled to room temperature and subjected to standard aqueous workup. Flash column chromatography using a 1:1 hexanes/EtOAc elution (Rf = 0.33) and concentration of the appropriate fractions afforded a fluffy yellow-orange solid (64% yield).

The target **SMI-10B3** was synthesized according to the general Doebner–Knoevenagel reaction procedure on the aldehyde intermediate with modifications. The acidified reaction mixture was extracted 2× 20 mL of EtOAc, and the organic layers were combined, dried over anhydrous Na_2_SO_4_, filtered, and concentrated to afford an orange crude residue. The crude residue was subjected to flash column chromatography (7% MeOH in EtOAc, Rf = 0.32), and the appropriate fractions were collected, concentrated under reduced pressure, and recrystallized from EtOAc/hexanes to afford a yellow crystalline solid (65% yield). ^1^H NMR (600 MHz, DMSO) δ 12.43 (s, 1H), 9.45 (s, 1H), 9.18 (s, 1H), 7.37 (d, *J* = 15.7 Hz, 1H), 7.08 (d, *J* = 7.7 Hz, 2H), 7.06–7.01 (m, 1H), 6.93 (s, 1H), 6.81 (s, 2H), 6.77 (d, *J* = 8.4 Hz, 1H), 6.28 (d, *J* = 15.7 Hz, 1H), 3.70 (s, 3H), and 3.64 (s, 3H). ^13^C NMR (151 MHz, DMSO) δ: 167.63, 149.87, 148.17, 147.65, 147.44, 147.31, 146.17, 130.23, 123.82, 123.30, 121.19, 121.08, 119.67–119.36 (m, 2C), 115.83–115.60 (m, 3C), 112.50, 110.14, 55.58, and 55.36. HRMS *m*/*z*: [M—H]^−^ Calcd for C_21_H_18_O_7_ 381.0988; Found 381.0969.

#### (E)-3-(4,5-Bis­(benzo­[d]­[1,3]­dioxol-5-yl)­thiophen-2-yl)­acrylic Acid (**SMI-10B4**)

The intermediate *4,5-bis­(benzo­[d]­[1,3]­dioxol-5-yl)­thiophene-2-carbaldehyde* was synthesized according to a modified version of the general Suzuki-Miyaura procedure on commercially obtained *4,5-dibromo-thiophene-2-carbaldehyde* (**2b**), where Cs_2_CO_3_ (6 equiv) is used as the base and Pd­(PPh_3_)_2_Cl_2_ (16 mol %)/AsPh_3_ (20 mol %) as the catalyst system. The black oil obtained from the standard aqueous workup was purified by flash column chromatography (3:1 hexanes/EtOAc, Rf = 0.25) to afford a crystalline yellow solid (93% yield).

The target compound **SMI-10B4** was synthesized according to the general Doebner–Knoevenagel reaction procedure, yielding a yellow solid (71% yield). ^1^H NMR (300 MHz, DMSO) δ 7.67 (d, *J* = 15.7 Hz, 1H), 7.54 (s, 1H), 6.91 (t, *J* = 8.1 Hz, 2H), 6.86 – 6.64 (m, 4H), 6.19 (d, *J* = 15.7 Hz, 1H), 6.05 (s, 2H), and 6.03 (s, 2H). ^13^C NMR (151 MHz, DMSO) δ: 167.37, 147.52, 147.46, 147.36, 146.60, 139.72, 138.04, 136.61, 136.08, 134.67, 128.99, 126.82, 122.92, 122.40, 118.13, 108.99, 108.93, 108.78, 108.54, 101.50, and 101.18. HRMS *m*/*z*: [M—H]^−^ Calcd for C_21_H_14_O_6_S 393.0427; Found 393.0414.

#### (E)-3-(4,5-Bis­(benzo­[d]­[1,3]­dioxol-5-yl)-1H-pyrrol-2-yl)­acrylic Acid (**SMI-10B5**)

The dibrominated aldehyde *4,5-dibromo-1H-pyrrole-2-carbaldehyde* (**2c**) was prepared according to the procedure reported by Zhang et al.,^61^ with a modification of the purification. To a round-bottom flask was added 20 mL of anhydrous THF together with commercially obtained pyrrole-2-carboxaldehyde (0.970 g, 10.0 mmol), and the flask was cooled to −78 °C in an acetone/liquid nitrogen bath. The solution was stirred for 15 min, after which the acetone bath was removed, and NBS (3.685 g, 20.5 mmol, 2.05 equiv) was added to the reaction vessel together with an additional 5 mL of THF. After 22 h at room temperature, Na_2_SO_3_ (2.583 g, 20.5 mmol) was added in one portion, and the reaction was stirred for another 30 min. The reaction mixture was then passed through a small layer of diatomaceous earth, and the filtrate was concentrated under reduced pressure to yield a pale-yellow crude residue. The residue was applied to a 6 in. silica flash chromatography column (4 cm) and eluted through with a gradient of hexanes/EtOAc from 20:1 to 5:1. A fluffy white solid was obtained (1.593 g, 63% yield).

The intermediate *4,5-bis­(benzo­[d]­[1,3]­dioxol-5-yl)-1H-pyrrole-2-carbaldehyde* was synthesized according to the general Suzuki–Miyaura procedure on aldehyde (**2b**) with a modified reaction solvent of 6:1 1,2-dimethoxyethane/H_2_O. The crude brown residue obtained is purified by repeated recrystallization from CH_2_Cl_2_ to afford a white solid (87% yield, two steps). The crude residue can also be purified by flash column chromatography (2:1 hexanes/EtOAc, Rf = 0.26), but its solubility in most common solvent systems was found to be exceptionally poor, and the crystallization method afforded the product of greater purity.

The ethyl ester of **SMI-10B5**, *ethyl (E)-3-(4,5-bis­(benzo­[d]­[1,3]­dioxol-5-yl)-1H-pyrrol-2-yl)­acrylate*, was prepared via addition of NaH (60% dispersion in mineral oil, 174 mg, 4.4 mmol, 2.7 equiv), freshly distilled THF (10 mL), and triethylphosphonoacetate (0.960 mL, 4.84 mmol, 3.0 equiv) to a flame-dried round-bottom flask purged with argon at 0 °C. The external ice bath was removed, and the mixture was allowed to stir and warm to rt over the course of 30 min. A solution of the aldehyde intermediate (540.7 mg, 1.61 mmol) in freshly distilled THF (5 mL) was transferred via cannula, and the resulting yellow solution was allowed to stir overnight. The reaction mixture is diluted in H_2_O (10 mL), extracted 2 × 30 mL with EtOAc, washed 1 × 25 mL with brine, dried (Na_2_SO_4_), and concentrated to afford a crude yellow oil. The oil obtained is purified by flash column chromatography (2:1 hexanes/EtOAc, Rf = 0.34), and concentration of the appropriate fractions afforded 420 mg of yellow solid (64% yield).

The target compound **SMI-10B5** was prepared by adding 6 M NaOH (1 mL) to a solution of the ethyl ester intermediate (119.3 mg, 0.294 mmol) in EtOH (3 mL) and heating to 85 °C for one h, after which the mixture was cooled to room temperature and acidified to pH 2 with 6 M HCl. The resulting yellow-brown precipitate is collected by filtration, washed with copious H_2_O, and dried via filtration to afford 90 mg of yellow-brown solid (81% yield). ^1^H NMR (600 MHz, DMSO) δ 11.99 (s, 1H), 11.50 (d, *J* = 2.6 Hz, 1H), 7.37 (d, *J* = 15.8 Hz, 1H), 6.93 (d, *J* = 8.1 Hz, 1H), 6.89 (d, *J* = 1.7 Hz, 1H), 6.89 – 6.81 (m, 2H), 6.74 (d, *J* = 1.7 Hz, 1H), 6.70 (dd, *J* = 8.0, 1.7 Hz, 1H), 6.67 (d, *J* = 2.6 Hz, 1H), 6.29 (d, *J* = 15.8 Hz, 1H), 6.04 (s, 2H), and 5.99 (s, 2H). ^13^C NMR (151 MHz, DMSO) δ 168.38, 147.25, 146.65, 145.59, 133.77, 132.05, 129.77, 127.96, 125.90, 122.90, 121.90, 121.40, 115.87, 112.29, 108.53 (d, *J* = 3.7 Hz), 108.44, 108.28, 101.22, and 100.86. HRMS *m*/*z*: [2 M—H]^−^ Calcd for C_21_H_15_NO_6_ 753.1715; Found 753.1719.

#### (E)-3-(4,5-Bis­(benzo­[d]­[1,3]­dioxol-5-yl)-1-methyl-1*H*-pyrrol-2-yl)­acrylic Acid (**SMI-10B6**)

The dibrominated aldehyde *4,5-dibromo-1-methyl-1H-pyrrole-2-carbaldehyde* was prepared according to the procedure reported by He et al.,^62^ with modifications. To a round-bottom flask was added commercially obtained *N*-methyl-2-pyrrolecarboxaldehyde (479.4 mg, 4.39 mmol) together with 8 mL of THF. The flask was cooled to −78 °C in an acetone/liquid nitrogen bath, and NBS (1.564 g, 8.29 mmol, 2.0 equiv) was added in one portion. After 15 h at room temperature, Na_2_SO_3_ (2000 g, 15.9 mmol) was added in one portion, and the reaction was stirred for another hour. The reaction mixture was then passed through a small layer of diatomaceous earth, and the filtrate was concentrated under reduced pressure to yield a pale-yellow crude residue. The residue was applied to an 8 in. silica flash chromatography column (3.5 cm) and eluted with a 4:1 hexanes/EtOAc mixture. A white, flaky solid was obtained (1.037 g, 88% yield).

#### 4,5-Bis­(benzo­[d]­[1,3]­dioxol-5-yl)-1-methyl-1*H*-pyrrole-2-carbaldehyde

The intermediate *4,5-bis­(benzo­[d]­[1,3]­dioxol-5-yl)-1-methyl-1H-pyrrole-2-carbaldehyde* was synthesized according to a modified version of the general Suzuki–Miyaura procedure. The product was purified via flash column chromatography using a 2:1 hexanes/EtOAc elution (Rf = 0.24). Concentration of the appropriate fractions afforded an off-white solid with 30% yield.

The target **SMI-10B6** was synthesized according to the general Doebner–Knoevenagel reaction procedure on the aldehyde intermediate, with modifications. The acidified reaction mixture was extracted with 2 × 25 mL of EtOAc, washed with 1x 20 mL of brine, dried over Na_2_SO_4_, filtered, and concentrated to afford a crude orange paste. The crude residue was purified by repeated recrystallization from EtOAc to afford an off-white solid (80% yield). ^1^H NMR (600 MHz, DMSO) δ 12.10 (s, 1H), 7.54 (d, *J* = 15.6 Hz, 1H), 7.04 (s, 1H), 6.99 (d, *J* = 8.0 Hz, 1H), 6.86 (d, *J* = 1.6 Hz, 1H), 6.77 (d, *J* = 8.1 Hz, 1H), 6.73 (dd, *J* = 7.9, 1.7 Hz, 1H), 6.63 (d, *J* = 1.7 Hz, 1H), 6.60 (dd, *J* = 8.1, 1.8 Hz, 1H), 6.24 (d, *J* = 15.6 Hz, 1H), 6.09 (s, 2H), 5.94 (s, 2H), and 3.46 (s, 3H). ^13^C NMR (151 MHz, DMSO) δ: 168.18, 147.47, 147.34, 147.10, 145.22, 134.23, 132.31, 129.42, 128.71, 125.06, 124.82, 123.16, 120.57, 113.64, 110.93, 110.76, 108.70, 108.25, 107.74, 101.41, 100.73, and 31.59. HRMS *m*/*z*: [M—H]^−^ Calcd for C_22_H_17_NO_6_ 390.0986; Found 390.1000.

#### (E)-3-(4,5-Bis­(2,3-dihydrobenzo­[b]­[1,4]­dioxin-6-yl)­furan-2-yl)­acrylic Acid (**SMI-10B7**)

The aldehyde intermediate *4,5-bis­(2,3-dihydrobenzo­[b]­[1,4]­dioxin-6-yl)­furan-2-carbaldehyde* was synthesized according to the general Suzuki–Miyaura procedure. Flash column chromatography performed in 2:1 hexanes/EtOAc (Rf = 0.27) gave a yellow solid (85%).

The target **SMI-10B7** was synthesized according to the general Doebner–Knoevenagel reaction procedure. Yellow solid (70% yield). ^1^H NMR (300 MHz, DMSO) δ 12.43 (s, 1H), 7.37 (d, *J* = 15.7 Hz, 1H), 7.08–6.99 (m, 3H), 6.94–6.76 (m, 4H), 6.28 (d, *J* = 15.7 Hz, 1H), and 4.31 – 4.19 (m, 8H). ^13^C NMR (75 MHz, DMSO) δ: 167.42, 149.18, 148.52, 143.97, 143.53, 143.36, 143.06, 130.32, 125.78, 123.24, 123.00, 121.36, 119.57, 119.41, 117.67–117.29 (m, 2C), 116.86, 116.04, 114.72, and 64.48–63.97 (m, 4C). HRMS *m*/*z*: [M—H]^−^ Calcd for C_23_H_18_O_7_ 405.0969; Found 405.0985.

#### (E)-3-(5-(Benzo­[d]­[1,3]­dioxol-5-yl)-4-phenylfuran-2-yl)­acrylic Acid (**SMI-10B8**)

The unsymmetrically coupled aldehyde *5-(benzo­[d]­[1,3]­dioxol-5-yl)-4-phenylfuran-2-carbaldehyde was s*ynthesized according to the general Suzuki–Miyaura procedure, with modifications. *3,4-(Methylenedioxy)­phenylboronic acid* (1.05 equiv) was added in one portion, and the reaction was monitored by TLC until disappearance of the dibrominated starting material (3 h), upon which PhB­(OH)_2_ (2.0 equiv) was added and the reaction was stirred at 90 °C overnight. The reaction mixture was cooled to room temperature and subjected to a standard aqueous workup. Flash column chromatography of the obtained crude oil (2:1 hexanes/EtOAc, Rf = 0.40) affords an inseparable mixture of the ca. 12:1 desired product/impurity (68% yield, two steps).

The target **SMI-10B8** was synthesized according to the general Doebner–Knoevenagel reaction procedure for the aldehyde intermediate, with modifications. The filtered solid obtained after acidification was recrystallized from CH_2_Cl_2_ to afford a crystalline brown solid (39% yield). ^1^H NMR (300 MHz, DMSO) δ 12.41 (s, 1H), 7.50–7.31 (m, 6H), 7.13 (s, 1H), 7.09–6.96 (m, 2H), 6.92 (d, *J* = 8.1 Hz, 1H), 6.34 (d, *J* = 15.7 Hz, 1H), and 6.05 (s, 2H). ^13^C NMR (75 MHz, DMSO) δ: 167.42, 149.61, 148.76, 147.70, 147.55, 132.85, 130.24, 128.93, 128.38, 127.77, 123.73, 123.57, 120.71, 119.22, 116.35, 108.74, 106.35, and 101.47. HRMS *m*/*z*: [M + H]^+^ Calcd for C_20_H_14_O_5_ 333.0758; Found 333.0769.

#### (E)-3-(4-(Benzo­[d]­[1,3]­dioxol-5-yl)-5-phenylfuran-2-yl)­acrylic Acid (**SMI-10B9**)

The unsymmetrically coupled aldehyde *4-(benzo­[d]­[1,3]­dioxol-5-yl)-5-phenylfuran-2-carbaldehyde* was synthesized according to the general Suzuki–Miyaura procedure, with modifications. PhB­(OH)_2_ (1.05 equiv) was added in one portion, and the reaction was monitored by TLC until disappearance of the dibrominated starting material (4 h), upon which *3,4-(methylenedioxy)­phenylboronic acid* (2.0 equiv) was added, and the reaction was stirred at 90 °C overnight. The reaction mixture was cooled to room temperature and subjected to the standard aqueous workup. Flash column chromatography of the obtained crude oil (3:1 hexanes/EtOAc, Rf = 0.42) afforded a fluffy yellow solid (44% yield in two steps).

The target compound **SMI-10B9** was synthesized according to the general Doebner–Knoevenagel reaction procedure on the aldehyde intermediate to afford a yellow solid (54% yield). ^1^H NMR (600 MHz, DMSO) δ 12.46 (s, 1H), 7.58–7.52 (m, 2H), 7.44–7.31 (m, 4H), 7.12 (s, 1H), 6.97 (d, *J* = 8.0 Hz, 1H), 6.92 (d, *J* = 1.7 Hz, 1H), 6.86 (dd, *J* = 7.9, 1.7 Hz, 1H), 6.33 (d, *J* = 15.7 Hz, 1H), and 6.06 (s, 2H). ^13^C NMR (151 MHz, DMSO) δ: 167.39, 149.45, 149.05, 147.67, 146.96, 130.34, 129.71, 128.84, 128.64, 126.33, 126.18, 124.57, 122.01, 119.38, 116.64, 108.82, 108.71, and 101.29. HRMS *m*/*z*: [M—H]^−^ Calcd for C_20_H_14_O_5_ 333.0758; Found 333.0766.

#### (E)-3-(4,5-Diphenylfuran-2-yl)­acrylic Acid (**SMI-10B10**)

The aldehyde intermediate *4,5-diphenylfuran-2-carbaldehyde* was synthesized according to the general Suzuki–Miyaura procedure. The crude residue thus obtained was purified by flash column chromatography (5:1 hexanes/EtOAc). A yellow-orange oil was obtained, 73% yield.

The target **SMI-10B10** was synthesized according to the general Doebner–Knoevenagel reaction procedure on the aldehyde intermediate to afford a yellow solid (83% yield). ^1^H NMR (300 MHz, DMSO) δ 12.45 (s, 1H), 7.58–7.48 (m, 2H), 7.50–7.29 (m, 9H), 7.17 (s, 1H), 6.35 (d, *J* = 15.8 Hz, 1H). ^13^C NMR (75 MHz, DMSO) δ: 167.33, 149.63, 149.27, 132.75, 130.29, 129.67, 128.93, 128.80, 128.71, 128.35, 127.86, 126.16, 124.76, 119.15, and 116.73. HRMS *m*/*z*: [M—H]^−^ Calcd for C_19_H_14_O_3_ 289.0859; Found 289.0865.

#### (E)-3-(5-(Benzo­[d]­[1,3]­dioxol-5-yl)­furan-2-yl)­acrylic Acid (**SMI-10B11**)

The aldehyde intermediate *5-(benzo­[d]­[1,3]­dioxol-5-yl)­furan-2-carbaldehyde was s*ynthesized according to a modified version of the general Suzuki–Miyaura procedure on commercially obtained *5-bromo-2-furaldehyde,* where Cs_2_CO_3_ (6 equiv) is used as the base and Pd­(PPh_3_)_2_Cl_2_ (16 mol %)/AsPh_3_ (20 mol %) as the catalyst system. An orange solid was obtained (66% yield).

The target **SMI-10B11** was synthesized according to the general Doebner–Knoevenagel reaction procedure on the aldehyde intermediate to afford a yellow solid (89% yield). ^1^H NMR (600 MHz, DMSO) δ 12.33 (s, 1H), 7.42 (d, *J* = 1.7 Hz, 1H), 7.39–7.34 (m, 2H), 7.03–6.96 (m, 3H), 6.30 (d, *J* = 15.7 Hz, 1H), and 6.08 (s, 2H). ^13^C NMR (151 MHz, DMSO) δ: 167.62, 155.23, 149.43, 148.00, 147.68, 130.47, 123.72, 118.47, 118.20, 115.36, 108.90, 107.69, 104.68, and 101.47. HRMS *m*/*z*: [M—H]^−^ Calcd for C_14_H_10_O_5_ 257.0445; Found 257.0455.

#### (E)-3-(4-(Benzo­[d]­[1,3]­dioxol-5-yl)­furan-2-yl)­acrylic Acid (**SMI-10B12**)

The aldehyde intermediate *4-(benzo­[d]­[1,3]­dioxol-5-yl)­furan-2-carbaldehyde* was prepared according to a modified version of the general Suzuki–Miyaura procedure on commercially obtained *4-bromo-2-furaldehyde*, where Cs_2_CO_3_ (6 equiv) is used as the base and Pd­(PPh_3_)_2_Cl_2_ (16 mol %)/AsPh_3_ (20 mol %) as the catalyst system. A yellow-orange oil was obtained in 55% yield.

The target **SMI-10B12** was synthesized according to the general Doebner–Knoevenagel reaction procedure on the aldehyde intermediate to afford a brown solid (66% yield). ^1^H NMR (300 MHz, DMSO) δ 12.44 (s, 1H), 8.26 (s, 1H), 7.44–7.31 (m, 2H), 7.24 (d, *J* = 1.7 Hz, 1H), 7.11 (dd, *J* = 8.0, 1.8 Hz, 1H), 6.95 (d, *J* = 8.1 Hz, 1H), 6.20 (d, *J* = 15.8 Hz, 1H), and 6.04 (s, 2H). ^13^C NMR (75 MHz, DMSO) δ: 167.26, 151.01, 147.87, 146.64, 141.32, 130.67, 128.16, 125.01, 119.05, 116.58, 113.54, 108.69, 106.07, and 101.11. HRMS *m*/*z*: [M—H]^−^ Calcd for C_14_H_10_O_5_ 257.0445; Found 257.0451.

#### (E)-3-(4,5-di­(1*H*-Indol-6-yl)­furan-2-yl)­acrylic Acid (**SMI-10B13**)

The aldehyde *4,5-di­(1H-indol-6-yl)­furan-2-carbaldehyde* was prepared according to the general Suzuki–Miyaura procedure with a modified solvent system of 5:1 THF/H_2_O and *indole-6-boronic acid pinacol ester*. The reaction was stirred at reflux for 30 h, cooled to room temperature, and subjected to the standard aqueous workup. The brown residue obtained was purified by flash column chromatography (1:1 hexanes/EtOAc, Rf = 0.27), and the concentration of the appropriate fractions afforded a tan crystalline solid (77% yield).

The target **SMI-10B13** was synthesized according to the general Doebner–Knoevenagel reaction procedure for the aldehyde intermediate, with modifications. The acidified reaction mixture was extracted 2 × 25 mL of EtOAc, washed 1x 20 mL of brine, dried over Na_2_SO_4_, filtered, and concentrated to afford a brown oil. The crude oil was purified by flash column chromatography (1:9 hexanes/EtOAc, Rf = 0.20), concentrated, and triturated from hexanes/EtOAc to afford a yellow-orange crystalline solid (66% yield). Of note, **SMI-10B13** can also be successfully synthesized through the Horner-Wadsworth-Emmons reaction of ***4,5-di­(1H-indol-6-yl)­furan-2-carbaldehyde*** as described in the synthesis of compound **5** below (86% yield), followed by the saponification of the ethyl ester, as described in the synthesis of **SMI-10B14** below (90% yield). ^1^H NMR (300 MHz, DMSO) δ 11.18 (d, *J* = 11.4 Hz, 2H), 7.64 (s, 1H), 7.56 (d, *J* = 8.1 Hz, 1H), 7.54–7.42 (m, 2H), 7.40–7.29 (m, 3H), 7.25 (d, *J* = 7.6 Hz, 1H), 7.11–7.01 (m, 2H), 6.48–6.37 (m, 2H), and 6.33 (d, *J* = 15.7 Hz, 1H). ^13^C NMR (75 MHz, DMSO) δ: 168.52, 150.43, 149.00, 136.15, 135.69, 128.57 (m, 2C), 127.79, 127.05, 126.05, 125.93, 124.36, 123.07, 120.33, 120.18, 119.74, 118.73 (m, 2C), 117.66, 111.07, 109.45, 101.31, and 101.11. HRMS *m*/*z*: [M—H]^−^ Calcd for C_23_H_16_N_2_O_3_ 367.1077; Found 367.1072.

### Synthesis of **SMI-10B** Analogs (Scheme 3C, **SMI-10B14**–**B18**)

#### Ethyl 3-(4,5-dibromofuran-2-yl)­propanoate (**5**)

A 2-neck, flame-dried 100 mL round-bottom flask was purged with argon and cooled in an ice bath. LiBr (976.6 mg, 11.25 mmol, 2.41 equiv) was added to the flask in one portion, followed by 15 mL of THF. The flask was purged again, and *triethyl phosphonoacetate* (2.04 mL, 10.27 mmol, 2.20 equiv) was added in one portion, followed by Et_3_N (1.50 mL, 10.74 mmol, 2.30 equiv) dropwise. Compound **2** (1.1852 g, 4.668 mmol), dissolved in 5 mL of THF, was added via cannula dropwise under inert gas. The reaction was monitored by 6:1 hexanes/EtOAc TLC and stirred for 30 min at 0 °C. A 5 mL of sat. aqueous solution of NH_4_Cl was added and concentrated under reduced pressure. The reaction mixture was transferred to a separatory funnel and redissolved with 25 mL of EtOAc and 25 mL of DI H_2_O. The phases were separated, and the aqueous phase was extracted with 3x 20 mL of EtOAc. The combined organic phases were washed with 2x 15 mL of 1 M NaOH, 1x 15 mL of H_2_O, and 1x 15 mL of brine. The organic layer was dried with MgSO_4_, filtered, and concentrated under reduced pressure. The resulting brown oil was purified on a 6 in. (7 cm diameter) silica column using 6:1 hex:EtOAc as the eluent, collecting 25 mL fractions.. Fractions 19–35 were combined and concentrated under reduced pressure to yield *ethyl (E)-3-(4,5-dibromofuran-2-yl)­acrylate* as a white, crystalline solid (1.285 g, 85% yield).

To a 2-neck, 100 mL round-bottom flask purged with N_2_, the enoate from above (1.6506 g, 5.10 mmol) was added and allowed to dissolve in 20 mL of THF. To the solution, TsNHNH_2_ (7590.5 mg, 40.76 mmol, 8 equiv) was added in one portion, followed by sodium acetate (3761.4 mg, 45.854 mmol, 9 equiv) and 20 mL DI H_2_O. The reaction was refluxed at 100 °C for 11 h, after which the mixture was concentrated under reduced pressure and redissolved in 75 mL of EtOAc. The mixture was extracted twice with 75 mL portions of EtOAc and then washed with 3x 100 mL of DI H_2_O and 1x 50 mL of brine. The organic layer was dried in MgSO_4_, filtered, and concentrated under reduced pressure until a yellow solid was obtained. The crude mixture was purified on a 6-in. silica (5 cm) column using a 7:1 hex/EtOAc elution. The product was found in 10 mL fractions 18–25, which were concentrated and yielded a white solid (876.1 mg, 53% yield).

#### Ethyl 3-(5-(benzo­[d]­[1,3]­dioxol-5-yl)-4-bromofuran-2-yl)­propanoate (**6**)

To a 100 mL round-bottom flask under N_2_, compound **6** (3125.2 mg, 9.59 mmol, 1.5 equiv) was added together with 20 mL of toluene and 10 mL of EtOH. To the solution, *3,4-(methylenedioxy)­phenylboronic acid* (1061 mg, 6.39 mmol, 1 equiv) and Pd­(PPh_3_)_4_ (178 mg, 0.154 mmol, 0.024 equiv) were added in one portion. 20 mL of 2N Na_2_CO_3_ (aq) were added dropwise. The mixture was refluxed at 110 °C overnight, after which 50 mL DI H_2_O were added and extracted with 3x 50 mL of EtOAc. The organic layer was washed with 50 mL of brine, dried in MgSO_4_ and concentrated under reduced pressure until a yellow solid was obtained. The crude mixture was purified on a 6-in. silica gel (7 cm) column using a 5:1 hexanes/EtOAc elution. The product was found in 25 mL fractions 55–75, which were concentrated and yielded an orange solid (845.1 mg, 34% yield).

### General Procedure for Suzuki–Miyaura Coupling of Compound **6**

Monocoupled intermediate **6** (1 equiv) was added in one portion to a solution of 2:1:2 v/v/v PhMe/EtOH/2N Na_2_CO_3_ (5 mL per 50 mg of starting material), followed by boronic acid (2.0 equiv) and Pd­(PPh_3_)_4_ (0.05 equiv). The reaction was stirred and refluxed at 110 °C under a N_2_ atmosphere. Reaction progress is monitored by TLC (hexanes/EtOAc elution), and upon disappearance of starting material, the reaction mixture is cooled to RT, and diluted in 25 mL DI H_2_O. The mixture was extracted with 3 × 25 mL EtOAc, washed with 1 × 25 mL brine, dried over MgSO_4_, filtered, and concentrated under reduced pressure to afford a brown crude residue. The residue is purified by flash column chromatography (silica, hexanes/EtOAc elution as specified below), and concentration of the appropriate fractions affords the dicoupled furan-ethyl-propenone.

### General Saponification Procedure

25 mg portion of starting material is dissolved in 0.5 mL of EtOH and heated to 90 °C with reflux. Aqueous 6 M NaOH (10 equiv of NaOH) was added dropwise, and the reaction was stirred for 1.5 h. The reaction mixture was then concentrated under reduced pressure, suspended in about 1 mL of 6 M HCl, filtered, and the solid dried under vacuum.

#### 3-(4,5-Bis­(benzo­[d]­[1,3]­dioxol-5-yl)­furan-2-yl)­propanoic Acid (**SMI-10B14**)

Compound **5** (163.2 mg, 0.501 mmol, 1 equiv) was loaded in a 10 mL round-bottom flask and dissolved in 2 mL toluene and 1 mL EtOH. The flask was purged with N_2_ gas and *3,4-(methylenedioxy)­phenylboronic acid* (207.64 mg, 1.252 mmol, 2.5 equiv) was added in one portion, followed by Pd­(PPh_3_)_4_ (28.95 mg, 0.0251 mmol, 0.05 equiv) in one portion. 2 mL of 2N Na_2_CO_3_ (aq) were added dropwise, and the reaction was stirred and refluxed at 110 °C overnight. The reaction mixture was transferred to a separatory funnel, and 50 mL DI H_2_O were added. The mixture was extracted with 3 × 50 mL of EtOAc, and the organic layer was washed with 50 mL brine, dried with MgSO_4_, filtered, and concentrated under reduced pressure until a yellow solid was obtained. The mixture was purified in a 3 cm diameter column loaded with 6 in. of silica using 500 mL 6:1 hexane/EtOAc solvent. A total of 100 5-mL fractions were collected, and the product was found in fractions starting at number 34. These fractions were concentrated and yielded a yellow solid (184.2 mg, 91% yield). This product was then dissolved in 3 mL of EtOH, and 6 M NaOH (aq) (0.76 mL, 4.56 mmol, 10 equiv) was added dropwise. The mixture was refluxed at 90 °C for 30 min. The mixture was then concentrated under reduced pressure and acidified by suspending it in approximately 1 mL 6 M HCl. The precipitate was then filtered, and a gray solid was obtained (109.2 mg, 63% yield, 57% yield overall for the combined two steps). ^1^H NMR (300 MHz, CDCl_3_ with 0.05% v/v TMS) δ 7.00 (dd, *J* = 8.1, 1.7 Hz, 1H), 6.95 (d, *J* = 1.7 Hz, 1H), 6.89–6.75 (m, 3H), 6.74 (d, *J* = 8.1 Hz, 1H), 6.17–6.10 (m, 1H), 5.97 (s, 2H), 5.94 (s, 2H), 3.03 (t, *J* = 7.5 Hz, 2H), and 2.79 (t, *J* = 7.5 Hz, 2H). ^13^C NMR (75 MHz, CDCl_3_ with 0.05% v/v TMS) δ: 177.61, 152.29, 147.90, 147.70, 146.99 (m, 2C), 146.80, 128.29, 125.48, 122.15, 121.76, 120.29, 110.13, 109.24, 108.72, 108.54, 106.92, 101.17 (m, 2C), 32.34, and 23.34. HRMS *m*/*z*: [M + H]^+^ Calcd for C_21_H_16_O_7_ 381.0969; Found 381.0977.

#### 3-(5-(Benzo­[d]­[1,3]­dioxol-5-yl)-4-(3,4-difluorophenyl)­furan-2-yl)­propanoic Acid (**SMI-10B15**)

Synthesis was carried out according to general procedure for Suzuki–Miyaura coupling of **6** and saponification of the corresponding dicoupled ester. Flash column chromatography elution solvent 6:1 hexanes/EtOAc was used for purification of the ester intermediate. The process yielded a yellow solid with a 78% yield. ^1^H NMR (600 MHz, CDCl_3_ with 0.05% v/v TMS) δ 10.68 (s, 1H), 7.19–7.13 (m, 1H), 7.13–7.03 (m, 2H), 6.94 (dd, *J* = 8.1, 1.7 Hz, 1H), 6.90 (d, *J* = 1.7 Hz, 1H), 6.75 (d, *J* = 8.1 Hz, 1H), 6.16 (t, *J* = 0.9 Hz, 1H), 5.96 (s, 2H), 3.04 (t, *J* = 7.4 Hz, 1H), and 2.79 (t, *J* = 7.5 Hz, 2H). ^13^C NMR (151 MHz, CDCl_3_ with 0.05% v/v TMS) δ 177.38, 152.77, 151.28 (d, *J* = 12.9 Hz, 0.5C), 150.41 (d, *J* = 12.6 Hz, 0.5C), 149.64 (d, *J* = 12.6 Hz, 0.5C), 148.77 (d, *J* = 12.5 Hz, 0.5C), 147.86, 147.67, 147.39, 131.55–131.40 (m, 1C), 124.86, 124.74 (dd, *J* = 6.2, 3.4 Hz, 1C), 120.57, 120.08 (m, 2C), 117.56 (t, *J* = 16.8 Hz, 1C), 109.61, 108.66, 107.03, 101.32, 32.22, and 23.27. HRMS *m*/*z*: [M + H]^+^ Calcd for C_20_H_14_F_2_O_5_ 373.0882; Found 373.0893.

#### 3-(5-(Benzo­[d]­[1,3]­dioxol-5-yl)-4-(pyrimidin-5-yl)­furan-2-yl)­propanoic Acid (**SMI-10B16**)

Synthesis according to the general procedure for Suzuki-Miyaura coupling of **6** with modifications and saponification of the corresponding dicoupled ester with modifications. Flash column chromatography was used for purification of the ester intermediate, employing a gradient elution solvent system ranging from 4:1 to 1:2 hexanes/EtOAc. The process yielded a yellow solid with an 83% yield. ^1^H NMR (600 MHz, DMSO) δ 9.11 (s, 1H), 8.78 (s, 2H), 6.96–6.92 (m, 2H), 6.90 (dd, *J* = 8.1, 1.7 Hz, 1H), 6.57 (t, *J* = 1.0 Hz, 1H), 6.06 (s, 2H), 2.92 (t, *J* = 7.9 Hz, 1H), and 2.64 (t, *J* = 7.4 Hz, 2H). ^13^C NMR (75 MHz, DMSO) δ: 173.33, 156.69, 155.61, 154.42, 147.83 – 147.59 (m), 147.43, 128.05, 123.84, 120.50, 114.89, 108.86, 108.55, 106.55, 101.43, and 22.96. HRMS *m*/*z*: [M—H]^−^ Calcd for C_18_H_14_N_2_O_5_ 337.0819; Found 337.0820.

#### 3-(5-(Benzo­[d]­[1,3]­dioxol-5-yl)-4-(4-methoxyphenyl)­furan-2-yl)­propanoic acid (**SMI-10B17**)

Synthesis according to the general procedure for Suzuki–Miyaura coupling of **6** with modifications and saponification of the corresponding dicoupled ester. Flash column chromatography elution solvent 5:1 hexanes/EtOAc was used for purification of the ester intermediate. Obtained a yellow solid, 58% yield. ^1^H NMR (600 MHz, CDCl_3_ with 0.05% v/v TMS) δ 7.18–7.13 (m, 2H), 6.90 (dd, *J* = 8.1, 1.7 Hz, 2H), 6.87 (d, *J* = 1.8 Hz, 1H), 6.79–6.71 (m, 2H), 6.60 (d, *J* = 8.1 Hz, 1H), 6.01 (s, 1H), 5.81 (s, 2H), 3.74 (s, 3H), 2.93 (t, *J* = 7.8 Hz, 2H), and 2.67 (t, *J* = 7.8 Hz, 2H). ^13^C NMR (151 MHz, CDCl_3_ with 0.05% v/v TMS) δ: 178.90, 158.69, 152.94, 147.55, 146.70, 146.54, 129.75, 126.88, 125.78, 121.66, 120.04, 114.11, 109.75, 108.41, 106.74, 101.03, 55.30, 33.50, and 23.86. HRMS *m*/*z*: [M + H]^+^ Calcd for C_21_H_18_O_6_ 367.1176; Found 367.1185.

#### 3-(5-(Benzo­[d]­[1,3]­dioxol-5-yl)-4-(3,4-dichlorophenyl)­furan-2-yl)­propanoic Acid (**SMI-10B18**)

Synthesis was carried out according to general procedure for the Suzuki-Miyaura coupling of **6** and saponification of the corresponding dicoupled ester. Flash column chromatography elution solvent 4:1 hexanes/EtOAc was used for purification of the ester intermediate. The process yielded a yellow, crystalline solid (72% yield). ^1^H NMR (600 MHz, CDCl_3_) δ 10.82 (s, 1H), 7.46 (d, *J* = 2.1 Hz, 1H), 7.38 (d, *J* = 8.3 Hz, 1H), 7.19 (dd, *J* = 8.3, 2.0 Hz, 1H), 6.94 (dd, *J* = 8.2, 1.7 Hz, 1H), 6.92 (d, *J* = 1.7 Hz, 1H), 6.75 (d, *J* = 8.1 Hz, 1H), 6.17 (s, 1H), 5.97 (s, 2H), 3.04 (t, *J* = 7.5 Hz, 2H), and 2.79 (t, *J* = 7.5 Hz, 2H). ^13^C NMR (151 MHz, CDCl_3_ with 0.05% v/v TMS) δ: 177.55, 152.94, 147.95, 147.91, 147.49, 134.61, 132.80, 131.00, 130.69, 130.30, 127.97, 124.77, 120.65, 119.72, 109.42, 108.68, 107.05, 101.35, 32.24, and 23.26. HRMS *m*/*z*: [M + H]^+^ Calcd for C_20_H_14_Cl_2_O_5_ 405.0291; Found 405.0296.

### Fluorescence Quenching Assays

To perform fluorescence quenching assays, 2 mL of 1 μM OSM solution in polymethylmethacrylate cuvettes was prepared by dilution of a concentrated stock solution of recombinant OSM with buffer solution (100 mM sodium chloride, 50 mM sodium phosphate, 50 mM freshly added DTT, pH = 6.6).^48^ Cuvettes were titrated with 5 μL increments of a 1 mM small molecule stock solution in DMSO, with a cuvette titrated with an equivalent volume of DMSO as a control. A total of 21 titration points were collected, ensuring the total concentration of DMSO did not exceed 5% (v/v). In cases where little to no significant quenching was observed, the titration was supplemented with 2.5 μL increments of a 10 mM small molecule stock solution to increase the concentration delivered. The fluorescence intensity measurements were obtained on a Cary Eclipse spectrofluorometer equipped with a Xenon arc lamp. Fluorescence intensities were measured by exciting at 280 nm (4 nm slit width) and recording the emission from 345 to 355 nm with a photomultiplier tube voltage of 700 V, unless otherwise noted. Each titration point was normalized to OSM’s native fluorescence intensity, corrected by adding the decrease in fluorescence intensity caused by dilution with DMSO, and the average value of the three replicates for each point was plotted against concentration of small molecule delivered and fit to a modified Stern–Volmer function as described by Charlier and Plapp.^54^ Binding affinity (*K*_D_) values were obtained as the standard error from the fitting routine. All curve-fitting analysis was performed in GraphPad Prism 8.0.

### ^1^H,^15^N HSQC NMR Experiments

Protein was prepared and chemical shift perturbation (CSP) experiments were conducted as previously described by Mass et al.^48^ For SMI inhibitor titration studies, in a 5 mm NMR tube, 600 μL of a 100 μM sample of ^15^N OSM in NMR buffer (50 mM sodium phosphate (pH 6.6), 100 mM sodium chloride, and 5% D_2_O) was titrated with a 20 mM stock solution of SMI in d_6_-dimethyl sulfoxide. The SMI stock solutions were added in 0.75–4 μL increments to a final concentration of 260 μM (**SMI-10B**) or 200 μM (**SMI-10B13**) and final volumes of 636 μL (**SMI-10B**) and 614.25 μL (**SMI-10B13**). Titration points for **SMI-10B** were: 0, 20, 40, 60, 80, 100, 140, 180, 220, and 260 μM. Titration points for **SMI-10B13** were: 0, 0.5, 1.0, 6.0, 10, 20, 30, 40, 50, 60, and 200 μM.

### Surface Plasmon Resonance (SPR)

SPR experiments were performed on a Reichert SR7000 instrument that was fed by a Surveyor LC autosampler and pump. All experiments were conducted at 25 °C. In all experiments, OSM (∼24 kDa; as previously described)^48^ was immobilized to the surface of a PEF-10% carboxyl mixed self-assembled monolayer gold sensor chip (Reichert part #13206061), and the experiments were completed within 3 days. The immobilization phase was conducted using sterile-filtered, fresh 10 mM acetate buffer, pH 5, at a flow rate of 15 μL/min. The chip was first washed with 100 μL of 2 M NaCl in DI water and 100 μL of 10 mM NaOH in DI water. The chip was activated with a 100 μL injection of 10 mg/mL *N*-hydroxysuccinimide (NHS) and 40 mg/mL 1-ethyl-3-(3-dimethylaminopropyl)­carbodiimide (EDC) mixed in 1 mL of nanopure water immediately before injecting. Then, a 200 μg/mL solution of OSM (100–300 μL) in acetate buffer was diluted and injected multiple times with 100 μL injections to achieve a response of about 600–1000 RU. The remaining reactive NHS groups were then deactivated with a 100 μL injection of 1 M ethanolamine hydrochloride in DI H_2_O. All kinetics and surface competition binding assays were performed in sterile-filtered, fresh PBS buffer with 0.005% Tween-20, and run at a flow rate of 30 μL/min with 90 μL injections. At 30 min after each injection, the surface was washed with one or two injections of 10 mM NaOH. Buffer-only and buffer-plus-DMSO injection blanks were run as controls and subtracted during data analysis. Each injection was run in triplicate, and the average curve was generated. The real-time changes in refractive index units of the association and dissociation phases were monitored. The raw data were processed in Excel and graphed using GraphPad Prism 8.0. Protein used: gp130 (R&D Systems, cat. #671-GP) and OSMRβ (R&D Systems, cat. # 4389-OR).

### Cell Culture Maintenance

Human breast cancer cell lines T47D, MDA-MB-231, and MCF-7 were obtained from the American Type Culture Collection (ATCC; Manassas, VA). All cell lines were maintained in RPMI-1640 media (Genesee Scientific; San Diego, CA) supplemented with either 10% fetal bovine serum or 10% synthetic Fetal Clone III serum (Thermo Fisher; Waltham, MA) and 1% penicillin/streptomycin (Genesee Scientific). All cells and experimental incubations were sustained at 37 °C, 5% carbon dioxide, and 100% humidity in a sterile tissue culture incubator.

### Enzyme-Linked Immunosorbent Assays (ELISA)

For phospho-STAT3 (pSTAT3) ELISAs, T47D MDA-MB-231, and MCF-7 cells were seeded in 12- or 24-well plates at 70–75% confluency and allowed to adhere overnight, after which cells were serum-starved with serum-free RPMI-1640 for 4 h. SMIs (10 μM in DMSO or serially diluted to specified concentrations from a 100 mM stock in DMSO) and human recombinant OSM (10 ng/mL; PeproTech; #300–10T-10UG) were incubated in serum-free RPMI-1640 medium for 1 h. After incubation, the **SMI-10** analogs or SMI-10B13 and OSM were added to the serum-starved cells for 30 min. Immediately afterward, the cells were lysed using 1x PathScan Sandwich ELISA Lysis Buffer (Cell Signaling Technology; #7018S), collected, and stored at −20 °C. Lysates were analyzed for pSTAT3 levels using either the PathScan Phospho-STAT3 (Tyr705) Sandwich ELISA Antibody Pair kit (Cell Signaling Technology; #7146) or Human/Mouse Phospho-STAT3 (Y705) DuoSet IC ELISA (R&D Systems; DYC4607B), according to manufacture’s protocol. pSTAT3 expression was measured with absorbance at 450 nm and quantified by comparison relative to OSM-induced pSTAT3 levels. To calculate an IC50 for SMI-10B13, a standard curve was generated as described in the DuoSet IC ELISA datasheet to determine the concentration of pSTAT3.

### Immunoblot Assays

For immunoblot analysis, T47D breast cancer cells were treated and collected in the same manner as described above for the ELISA preparation. After lysate collection, samples were run on a 10% SDS-PAGE gel and transferred to a nitrocellulose membrane via semidry transfer. Blots were rinsed with DI H_2_O and allowed to completely dry at room temperature before blocking with LiCor Intercept PBS Blocking Buffer (LiCor, Lincoln, NE; Cat# 927–90001) for 1 h. After blocking, primary antibodies (1:1000) suspended in blocking buffer supplemented with 0.1% Tween were added, shaken at room temperature for 1 h, and stored at 4 °C overnight. Membranes were then washed 6 times for 5 minutes each with 1X PBS supplemented with 0.5% Tween while shaking at room temperature, and secondary antibodies (1:15,000) were applied and incubated for 1 h. Afterward, membranes were washed as previously described above and imaged at 700 nm using the LiCor Odyssey CLx Imaging System. Quantification of immunoblots was performed using the LiCor Image Studio software, normalizing to β-Actin, and calculating relative fold change compared to nontreated samples. Antibodies used included phospho-STAT3 (Y705) (Cell Signaling Technology, Danvers, MA; cat. no. 9145), phospho-AKT (S473) (Cell Signaling Technology, Danvers, MA; cat. no. 4060), phospho-JNK (T183/Y185) (Cell Signaling Technology, Danvers, MA; cat. no. 4668), phospho-ERK (T202/Y204) (Cell Signaling Technology, Danvers, MA; cat. no. 9101), β-Actin (Cell Signaling Technology; cat. no. 3700), and donkey antirabbit IRDye 800CW (LiCor, Lincoln, NE; Cat# 925–32213).

### MTS Assay

Cell viability was assessed using the CellTiter 96 AQueous One Solution Cell Proliferation Assay (Promega, cat. no. G3580), following the manufacturer’s instructions. Briefly, T47D and MCF-7 human breast cancer cells were seeded in 96-well plates at a density of 10,000 cells/well in 100 μL of complete growth medium and allowed to adhere overnight. Cells were then treated with a serial dilution (100 μM to 0.1 nM) of SMI-10B, SMI-10B13, Temozolomide (positive control), or vehicle (DMSO) for 24 h. Following treatment, 20 μL of MTS reagent was added directly to each well, and plates were incubated at 37 °C in a humidified incubator with 5% CO_2_ and imaged after 1 h of incubation. Absorbance was measured at 490 nm by using a microplate reader. Cell viability was expressed as a percentage relative to the vehicle-treated controls, and experiments were performed in triplicate.

### ER+ MCF-7-luc-OE-OSM Cell Line Development

For full-length human OSM (flOSM) vector transduction, the luminal A invasive ductal carcinoma cell line MCF-7-Luc was used. This cell line, purchased from PerkinElmer, had already been transformed to express the bioluminescent, enzymatic firefly luciferase protein by the incorporation of a firefly luciferase expression vector with G418 antibiotic resistance. Antibiotic resistance was critical in determining the plasmid for flOSM cDNA integration, ensuring that the resistances were different and did not overlap, thereby preventing unintended colony selection. The MCF-7-Luc cells were transformed to constitutively overexpress flOSM (MCF-7-Luc-flOSM) or empty vector control (MCF-7-Luc-EVctrl) using a CMV promoter-containing pLenti6.3/TO/V5-DEST plasmid found in the ViraPower HiPerform T-Rex Gateway Vector Kit (Thermo Fisher), according to the accompanying protocol. The plasmids contained either the full-length human OSM (flOSM) cDNA sequence, inserted according to protocol in the manual or were left empty (EVctrl) to control for the effects of transduction in MCF-7-Luc cells by lentiviral particles.

A series of volumes (20–100 μL) for each lentiviral plasmid construct, flOSM and EVctrl, was added to the wells of a tissue culture-treated 96-well plate (Genesee Scientific) plated with 15,000 or 30,000 cells in triplicate for each vector and volume combination, resulting in a total volume of 200 μL in combination with RPMI-1640 complete media. Consequently, MCF-7-Luc cells were exposed to lentiviral particles at ratios of 1:10 up to 1:2 for 24 or 48 h. Cells were washed with 1X PBS, and 200 μL of complete RPMI-1640 media containing 5 μg/mL blasticidin was added to select for MCF-7-Luc cells that expressed flOSM or EVctrl media. Selection media was replaced every 3 to 4 days until stable colonies were isolated and subcloned. These colonies were maintained by intermittently adding 2.5 μg/mL blasticidin to the media. For luciferase expression maintenance in parental MCF-7-Luc, MCF-7-Luc-flOSM, and MCF-7-Luc-EVctrl colonies, 250 μg/mL G418 was intermittently added to the media. Selected colonies were established, followed by characterizing the morphology and protein expression of each colony to find suitable cells for *in vivo* studies. RPMI-1640 media was slowly replaced with DMEM media due to excessive detachment observed in the MCF-7-Luc-flOSM colonies using RPMI-1640 media at confluency that DMEM prevented.

The flOSM and EVctrl pLenti6.3/TO/V5-DEST plasmids were transfected into HEK293FT cells (Thermo Fisher) with the use of ViraPower Packaging Mix (Thermo Fisher) and Lipofectamine 2000 (Thermo Fisher), following the protocols provided in ViraPower HiPerform Lentiviral Expression Systems (Thermo Fisher). All biosafety level 2 (BSL-2) guidelines and protocols were adhered to, including the use of a Class 2 tissue culture hood. After 24 h of incubation, the above transfection media was removed and replaced with fresh media after 1x PBS rinse. Conditioned media containing lentiviral particles that were formed and released by the cells was collected 72 h later. The resulting lentiviral media solution was carefully handled, and cellular debris was eliminated by centrifuging the conditioned media at 300 g for 5 min and sterile filtering it using a PVDF 0.45 μm luer lock syringe filter (Millipore Sigma). Following conditioned media cleanup, the lentiviral solution was titrated following ViraPower manufacturer protocol. Once the solution was titrated, the lentiviral conditioned media was safely and sterilely aliquoted into cryovials with screw-on caps that had O-rings for an airtight seal (Genesee Scientific) and stored at −80 °C.

### *In Vivo* Assays

Seven- to eight-week-old athymic nu/nu mice were purchased from Jackson Laboratory and quarantined for 6 days. Twelve hours before tumor cell injection, the mice were grouped and injected intraperitoneally (i.p.) with 50 mg/kg of **SMI-10B13** or vehicle (10% DMSO, 10% EtOH, 20% Cremophor EL, 60% propylene glycol; diluted in half with PBS). The following morning, MCF-7-Luc-flOSM cells (described above) were grown to 70–75% confluency, and the cells were concentrated to 4.0 × 10^7^ cells/mL in PBS containing serum-free RPMI-1640. A 50 μL cell suspension was injected into the fourth mammary fat pad, resulting in 2.0 × 10^6^ cells/injection. Mice were then injected (via i.p.) 3x weekly with **SMI-10B13** or the vehicle control. Mice were also injected 1x weekly with d-luciferin (PerkinElmer; 122799–5) for bioluminescence detection for a period of 40–50 days, and 3x weekly for tumor growth via caliper measurement. Animals were sacrificed, and their serum and tumors were collected for analysis. Organs were harvested, and *ex vivo* imaging was performed to evaluate metastasis. All in vivo and ex vivo imaging was performed as previously described.^63^ All animal experiments were performed following the protocols evaluated and approved by the Boise State University Institutional Animal Care and Use Committee (IACUC) (Ethics Approval Number: AC23-043).

## Supplementary Material







## Notes

Inhibitory activity of **SMI-10** analogs against the OSM signaling cascade as analyzed via ELISA and immunoblot analysis; inhibitory activity of **SMI-10B** analogs against the OSM signaling cascade as analyzed via ELISA and immunoblot analysis; surface plasmon resonance competition binding assay between gp130 and **SMI-10B13** for immobilized OSM; SwissADME data for **SMI-10B13**; ^1^H and ^13^C NMR spectra and HPLC chromatograms for analogs of **SMI-10** and **SMI-10B** (PDF) (PDF)
molecular formula strings (CSV) (XLSX)
SMI10B13Complex.pdb (PDB)

## ∇

Lam Research, 6401 S Eisenman Road, Suite 109, Boise, ID 83716

## Abbreviations

ADMET: absorption, distribution, metabolism, excretion, and toxicity
AKT: protein kinase B
ANOVA: analysis of variance
CLCLF1: cardiotrophin-like cytokine factor 1
CNTF: ciliary neurotrophic factor
CSP: chemical shift perturbation
CT-1: cardiotrophin-1
CYP: cytochrome P450
ECM: extracellular matrix
ELISA: enzyme-linked immunosorbent assay
ER+: estrogen receptor positive
ERK: extracellular signal-regulated kinase
GI: gastrointestinal
gp130: glycoprotein 130
HIF1α: hypoxia-inducible factor 1 alpha
HPLC: high-performance liquid chromatography
hERG: human ether-à-go-go-related gene potassium channel
HSAB: hard and soft acids and bases
HSQC: heteronuclear single quantum coherence
HTVS: high-throughput virtual screening
IC50: half maximal inhibitory concentration
IL-11: interleukin-11
IL-27: interleukin-27
IL-6: interleukin-6
JAK: Janus kinase
JNK: Jun N-terminal kinase
KD: dissociation constant
LIF: leukemia inhibitory factor
LIFRβ: leukemia inhibitory factor receptor beta
LOXL2: lysyl oxidase-like 2
MAPK: mitogen-activated protein kinase
MMP: matrix metalloproteinase
NCI: National Cancer Institute
NMR: nuclear magnetic resonance
OD: optical density
OSM: oncostatin M
OSMR: oncostatin-M receptor
OSMRβ: oncostatin-M receptor beta
P-gp: P-glycoprotein
PAINS: pan-assay interference compounds
pAKT: phosphorylated AKT
pERK: phosphorylated ERK
pJNK: phosphorylated JNK
pSTAT3: phosphorylated STAT3
PI3K: phosphatidylinositol 3-kinase
SAR: structure–activity relationship
SD: standard deviation
SMI: small molecule inhibitor
SPR: surface plasmon resonance
STAT3: signal transducer and activator of transcription 3
TLC: Thin-layer chromatography
TPSA: topological polar surface area
VEGF: vascular endothelial growth factor
ZINC: ZINC is not commercial
