# Supplementary material for: Development of the First Small-Molecule Inhibitor Targeting Oncostatin M for Treatment of Breast Cancer
Source: J Med Chem. 2025 Aug 1;68(15):15422–45. doi: 10.1021/acs.jmedchem.4c03233 (PMC12362618; doi:10.1021/acs.jmedchem.4c03233)
Supplement: Supplementary file 1 [file jm4c03233_si_001.pdf]

Supporting Information

Development of the First Small Molecule Inhibitor Targeting Oncostatin M for Treatment of Breast Cancer

Cody L. Wolf,[1,2,‡] Andrea Feci,[3,‡] Joseph P. Tuccinardi,[3‡] Grace H. Coughlin,[3] Kelsey A. Holdaway,[3] Thaaer Muhammed,[3] Clyde Pruett,[2] Darren Lighter,[2] Cooper McGrath,[2] Terrell Engmann,[1,2] Maria Pou-Torres,[1,2] Brittany Rushing,[3] Luke Woodbury,[4] Sierra E. Haile,[2] Hannah Scott,[2] Ken Tawara,[1,2] Simion Dinca,[1,2] Dong Xu,[5] Matthew D. King,[3] Lisa Rose Warner,[3] Cheryl L. Jorcyk,[2,*] and Don L. Warner[3,*]

[1]Department of Biomolecular Sciences, Boise State University, Boise, ID 83725. [2]Department of Biological Sciences, Boise State University, Boise, ID 83725. [3]Department of Chemistry and Biochemistry, Boise State University, Boise, ID 83725. [4]Biomedical Research Institute, Boise State University, Boise, ID 83725. [5]Biomedical and Pharmaceutical Sciences, Idaho State University, Meridian, ID 83642

[‡]These authors contributed equally.

Corresponding authors
*Don Warner; Tel.: (208) 426-3030 ; E-mail:donwarner@boisestate.edu
*Cheryl Jorcyk; Tel.: (208) 426-4287 ; E-mail:cjorcyk@boisestate.edu

Table of Contents

Page S2	**Figure S1. SMI-10** analogs inhibit OSM-mediated signaling cascades.
Page S3	**Figure S2. SMI-10B** analogs inhibit OSM-mediated pathways.
Page S4	**Figure S3.** Dose-response curve of **SMI-10B13** to determine IC_{50}
Page S5	**Figure S4.** OSM-gp130 binding kinetics and surface competition binding assay between gp130 and **SMI-10B13** for immobilized OSM.
Page S6	**Figure S5. SMI-10B** and **SMI-10B13** show minimal toxicity *in vitro*.
Page S7	**Table S1.** SwissADME Data for **SMI-10B13**
Pages S8-S56	**Characterization Data.** ^{1}H, ^{13}C NMR spectra, and HPLC chromatograms for analogs of SMI-10 and SMI-10B.

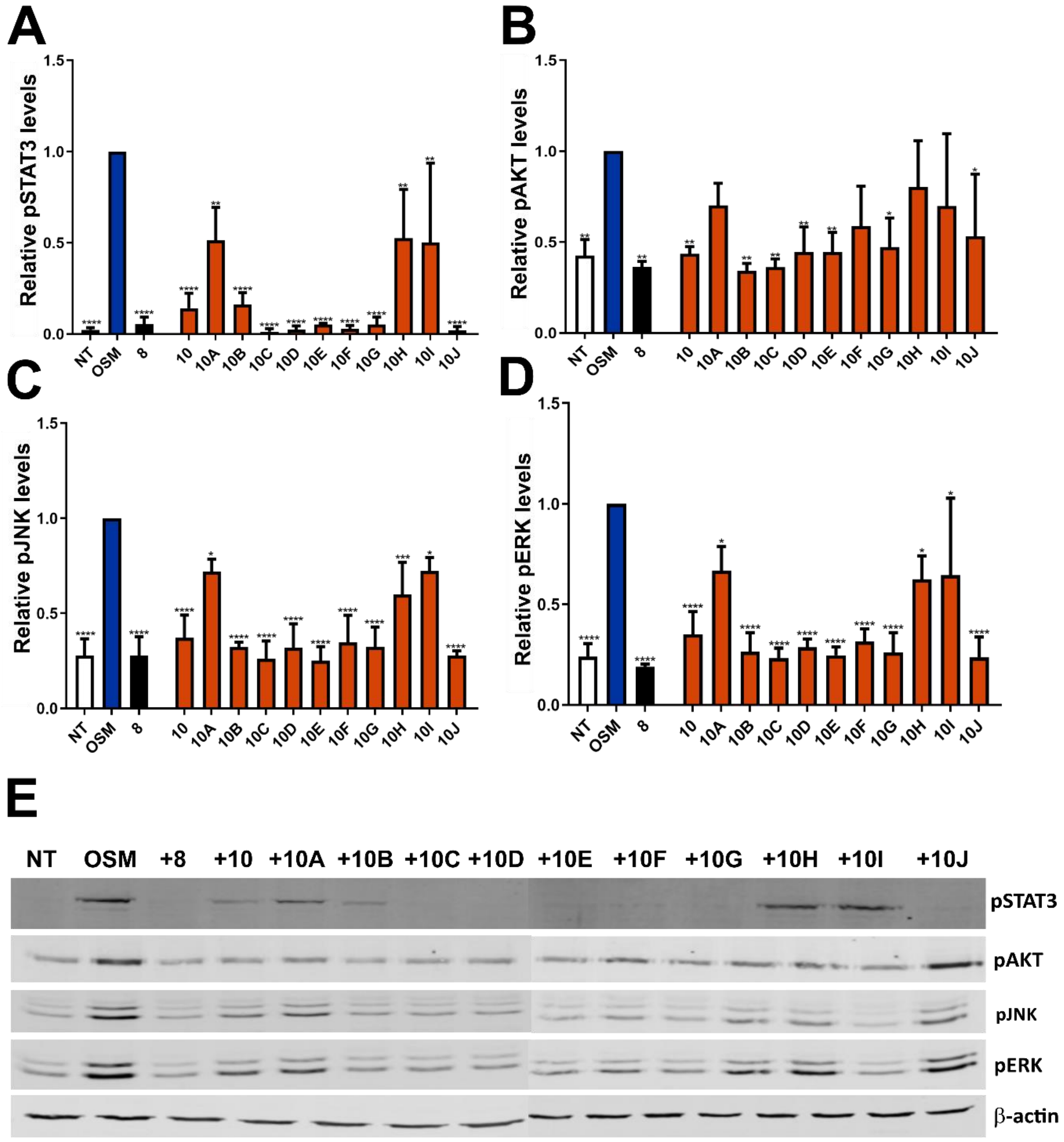

Figure S1. SMI-10 Analogs Inhibit OSM-mediated signaling cascade. Human T47D cells were treated with **SMI-10A** to **SMI-10J** SMI analogs (10 μM) and OSM (10 ng/mL) for a period of 30 minutes, and cell lysates were evaluated for repression of OSM-mediates signaling, particularly pSTAT3, pAKT, pJNK, and pERK. Immunoblot analysis identifies several compounds capable of inhibiting multiple cascades including **SMIs-10B**, **10C**, **10D**, **10E**, **10F**, and **10G**. **SMI-8** is used as a positive control. Protein concentration normalized to β-actin expression. Data expressed as mean ± SD and assessed relative to +OSM treatment by one-way ANOVA with Tukey's post-test *$p<0.05$, ** $p<0.01$, *** $p<0.001$, ****$p<0.0001$.

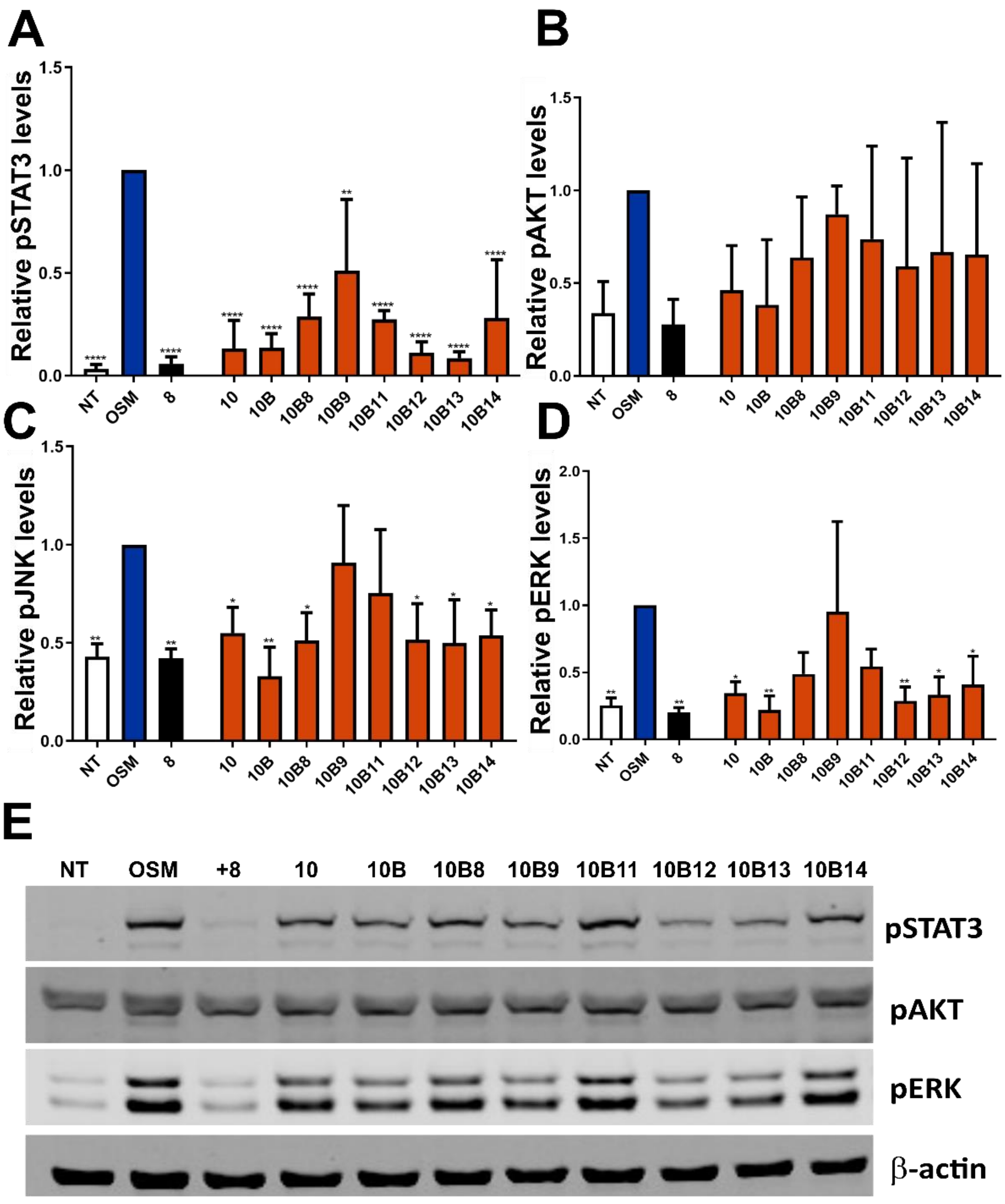

Figure S2. Second generation analogs of SMI-10B inhibit OSM-mediated signaling pathways. Human T47D cells were treated with select **SMI-10B** analogs (10 μM) and OSM (10 ng/mL) for 30 minutes, and cell lysates were evaluated for inhibition of OSM-mediated signaling pathways. Immunoblot analysis of **SMI-10B** analogs confirm effectiveness of SMIs to inhibit multiple signaling cascades. **SMI-10B8**, **10B12**, **10B13**, and **10B14** proved to be the most effective at inhibiting OSM-induced pSTAT3, pJNK, and pERK signaling cascades. No significant trends were noticed in repression of pAKT signaling. **SMI-8** was used as a positive control. Protein concentration was normalized to β-actin expression. Data expressed as mean ± SD and assessed relative to +OSM treatment by one-way ANOVA with Tukey's post-test *p<0.05, ** p<0.01.

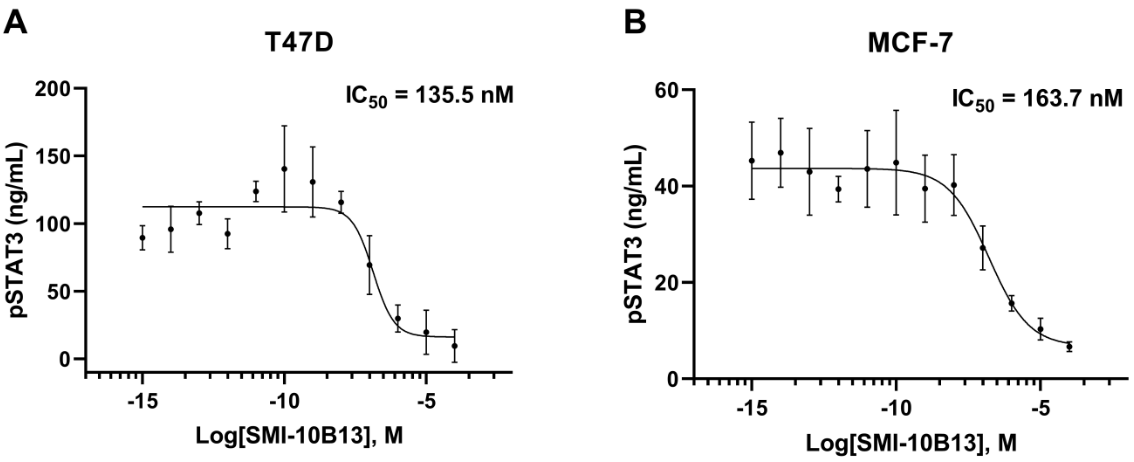

Figure S3. Dose response curve of SMI-10B13 to determine IC_{50}. Human breast cancer cell lines T47D and MCF-7 were treated with the specified concentration of **SMI-10B13** and the concentration of phosphorylated STAT3 (pSTAT3) was measured via ELISA. A four parameter nonlinear regression was fitted to the data to calculate an IC_{50} value for each cell line. The R squared values are 0.7952 for graph A and 0.8585 for graph B. The $LogIC_{50}$ for graph A is -6.868 ± 0.2204 ($IC_{50} = 1.355 \times 10^{-7}$), and -6.786 ± 0.2805 ($IC_{50} = 163.7 \times 10^{-7}$) for graph B. Data expressed as mean ± SD of three independent replicates.

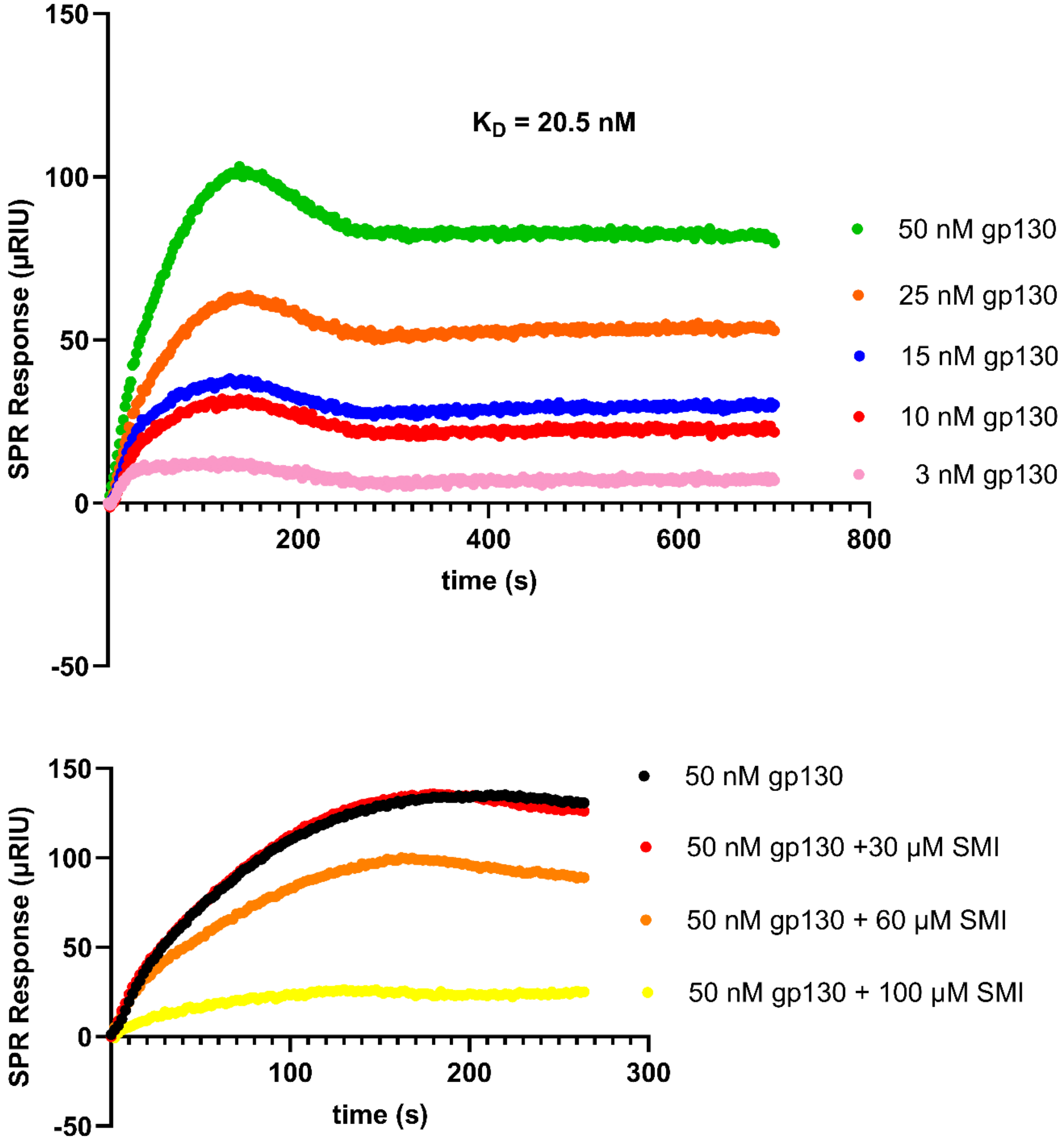

Figure S4. OSM-gp130 binding kinetics and surface competition binding assay between gp130 and SMI-10B13 for immobilized OSM. Top: Binding kinetics study of gp130 injections on immobilized OSM, revealing a K_D of 20.5 nM. To each injection, a 0 nM gp130 blank was subtracted. **Bottom:** Surface competition binding assay between gp130 and **SMI-10B13** for immobilized OSM. OSM is immobilized on the surface of the gold chip, injections of gp130 (50 nM) and **SMI-10B13** (black: 0 µM, red: 30 µM, orange: 60 µM, and yellow: 100 µM) are passed through the flow cell. The association phase is shown. All curves displayed are the result of three curves averaged to which the average of three blank curves, with a solution of **SMI-10B13** alone, was subtracted.

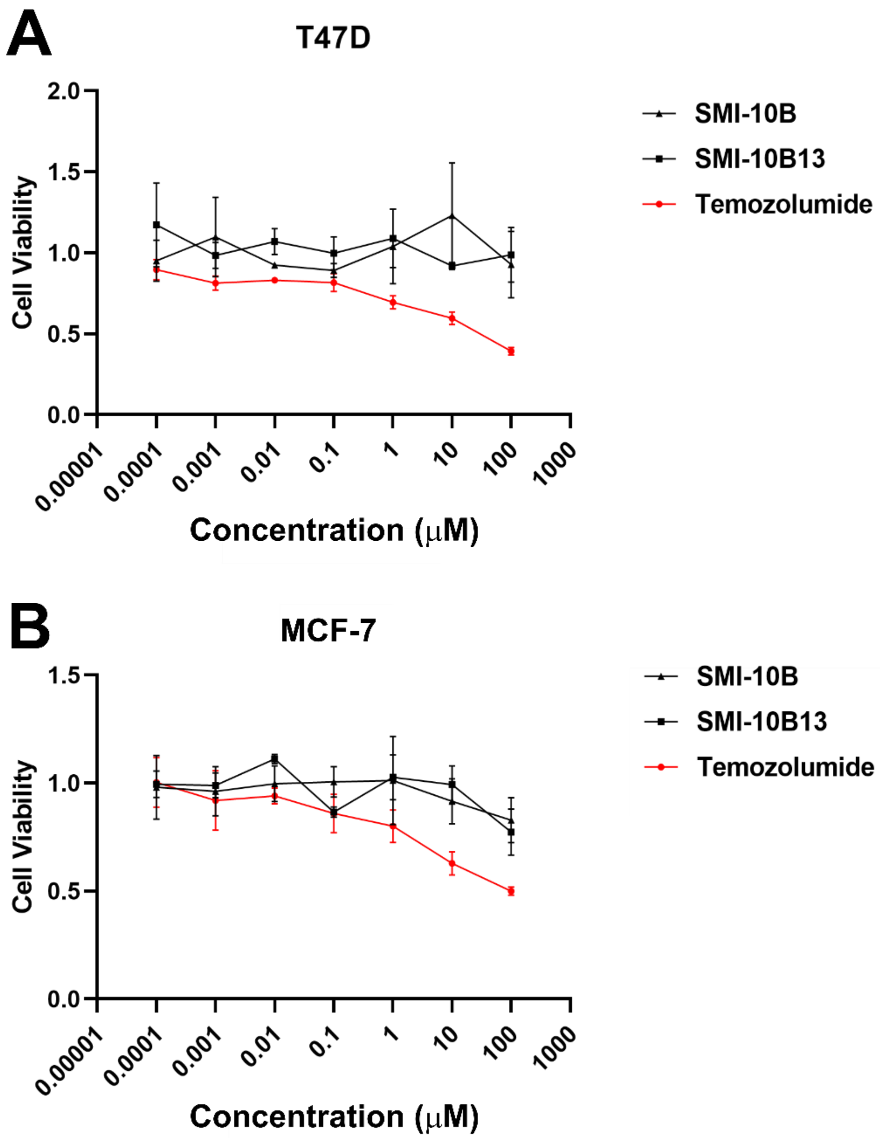

Figure S5. SMI-10B and SMI-10B13 show minimal toxicity *in vitro*. Human T47D and MCF7 human breast cancer cell lines were treated with varying concentrations of SMI-10B, SMI-10B13 and temozolomide (positive control) for a period of 24 hrs. Cell viability was analyzed via MTS assay. Minimal toxicity is observed for both SMI-10B and SMI-10B13, while temozolomide, a DNA-alkylating chemotherapeutic agent, shows moderate toxicity as concentration increases. Data expressed as mean ± SD over three independent replicates

Table S1: SwissADME[a] Data for SMI-10B13

Parameter		Value
**Physicochemical Properties**	Molecular Weight	368.38 g/mol
	TPSA	82.02 Å²
	Rotatable Bonds	4
**Lipophilicity**	Log $P_{o/w}$ (Consensus)	4.08
**Solubility**	Log S (ESOL)	-5.32
	Log S (Ali)	-5.96
	Log S (SILICOS-IT)	-7.83
**Absorption**	Gastrointestinal (GI) Absorption	High
	P-gp Substrate	No
**Distribution**	Blood-Brain Barrier (BBB) Permeability	No
	Skin Permeability (Log Kp)	-5.34 cm/s
**Metabolism**	CYP1A2 Inhibitor	Yes
	CYP2C19 Inhibitor	Yes
	CYP2C9 Inhibitor	No
	CYP2D6 Inhibitor	Yes
	CYP3A4 Inhibitor	No
**Excretion**	Renal Clearance	Not reported
**Toxicity**	hERG Inhibition	Not reported
	PAINS	0 alerts
	Brenk Alert	1 alert (Michael Acceptor)
**Drug-Likeness**	Lipinski's Rule	0 violations
	Veber, Ghose, Egan, Muegge	Yes
	Bioavailability Score	0.56

Supporting Table S1: SwissADME[a] Data for SMI-10B13. Lead compound **SMI-10B13** exhibits high gastrointestinal absorption, favorable lipophilicity (Log $P_{o/w}$ = 4.08), and satisfies Lipinski's rule with no PAINS alerts. However, it is predicted to have low water solubility (Log S < –5 across all models), which may limit oral bioavailability without further optimization. **SMI-10B13** is likely an inhibitor of some cytochrome P450 enzymes (CYP1A2, CYP2C19, and CYP2D6) and thus has the potential of causing drug-drug interactions. SwissADME identified a Brenk alert for **SMI-10B13** being a Michael acceptor, suggesting the possibility of nonspecific reactivity. Many of these concerns may be addressed by incorporating solubilizing polar functional groups (e.g., hydroxyl or carboxyl moieties) to improve solubility, and by modifying the electrophilic site to reduce off-target reactivity and CYP inhibition. Overall, these results indicate that **SMI-10B13** displays a promising drug-like profile but would benefit from further structural optimization.

[a]Daina, A., Michielin, O. & Zoete, V. SwissADME: a free web tool to evaluate pharmacokinetics, drug-likeness and medicinal chemistry friendliness of small molecules. Sci Rep **7**. 2017 March.

<u>Characterization Data</u>: ^{1}H and ^{13}C NMR spectra and HPLC chromatograms for all final SMIs.

SMI 10A

^{1}H NMR (600 MHz, $CDCl_3$ with 0.05% v/v TMS) δ 7.22 (s, 1H), 7.14 (dd, J = 8.2, 1.7 Hz, 1H), 7.05 (d, J = 1.8 Hz, 1H), 6.84 – 6.81 (m, 3H), 6.77 (d, J = 8.2 Hz, 1H), 6.00 (s, 2H), 5.97 (s, 2H), 3.91 (s, 3H).

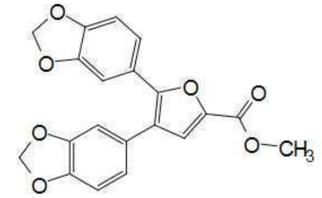

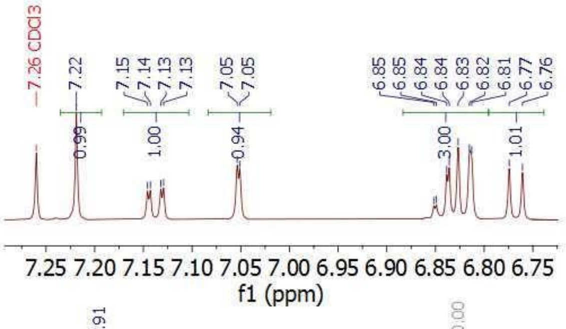

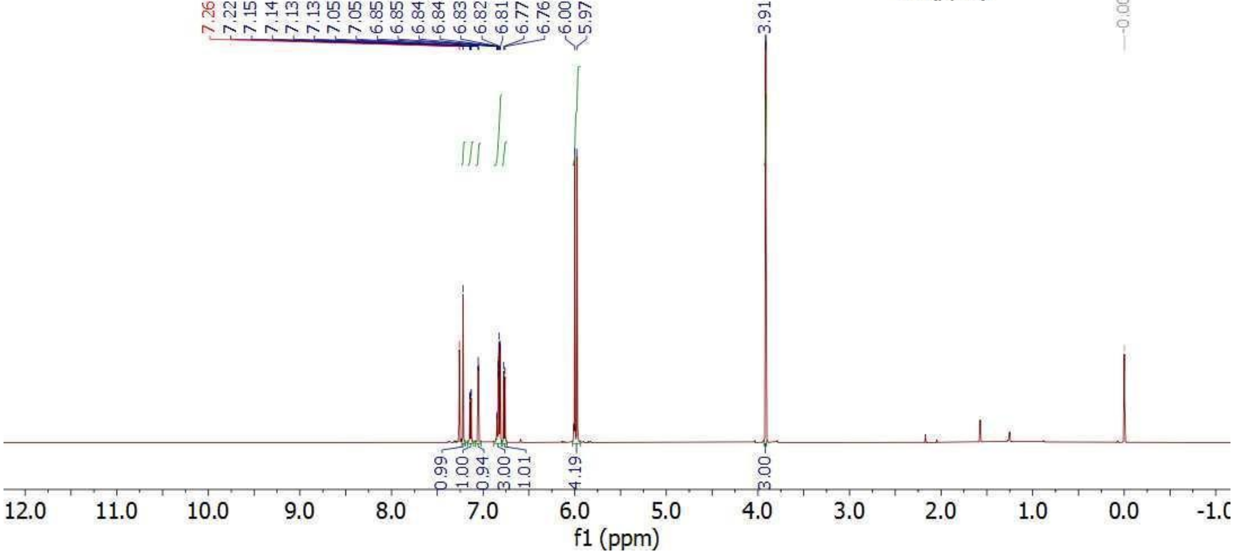

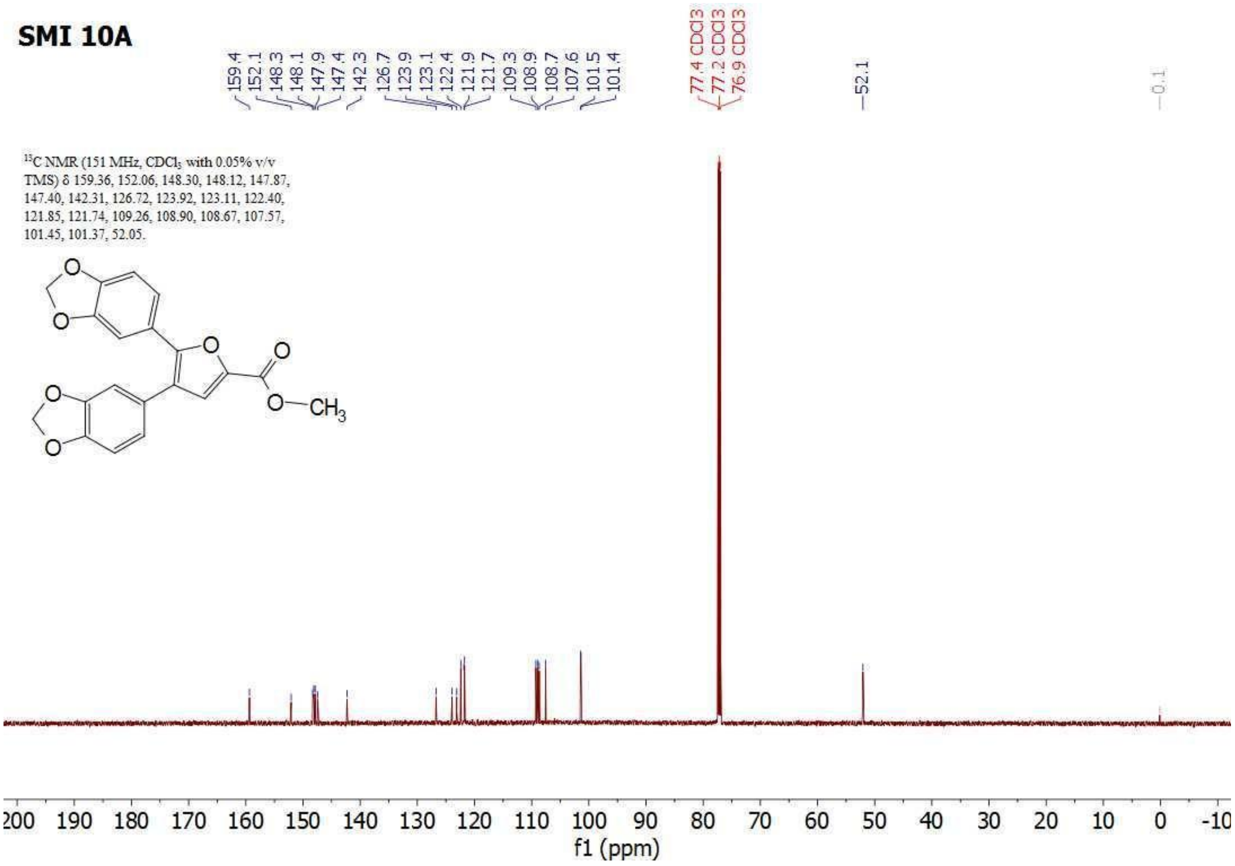

SMI-10A, solvent DMSO, UV abs. measured at 280 nm.

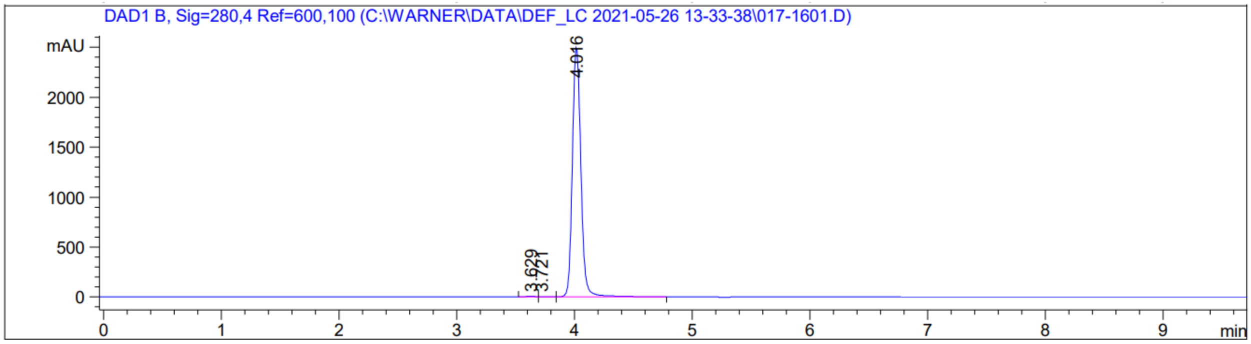

SMI 10B

^{1}H NMR (600 MHz, DMSO) δ 12.43 (s, 1H), 7.38 (d, J = 15.7 Hz, 1H), 7.06 (d, J = 9.8 Hz, 3H), 6.95 (dd, J = 15.4, 8.0 Hz, 2H), 6.91 (s, 1H), 6.85 (d, J = 8.0 Hz, 1H), 6.32 (d, J = 15.7 Hz, 1H), 6.06 (s, 2H), 6.06 (s, 2H).

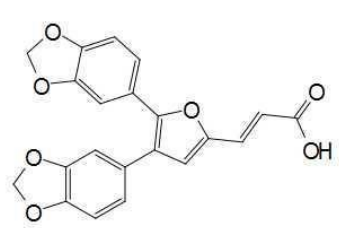

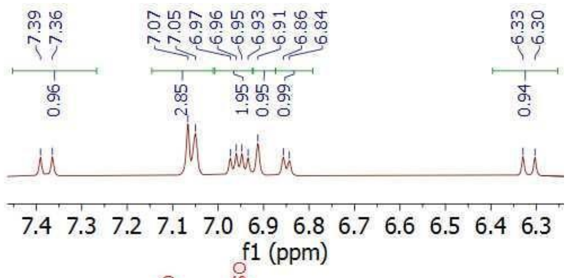

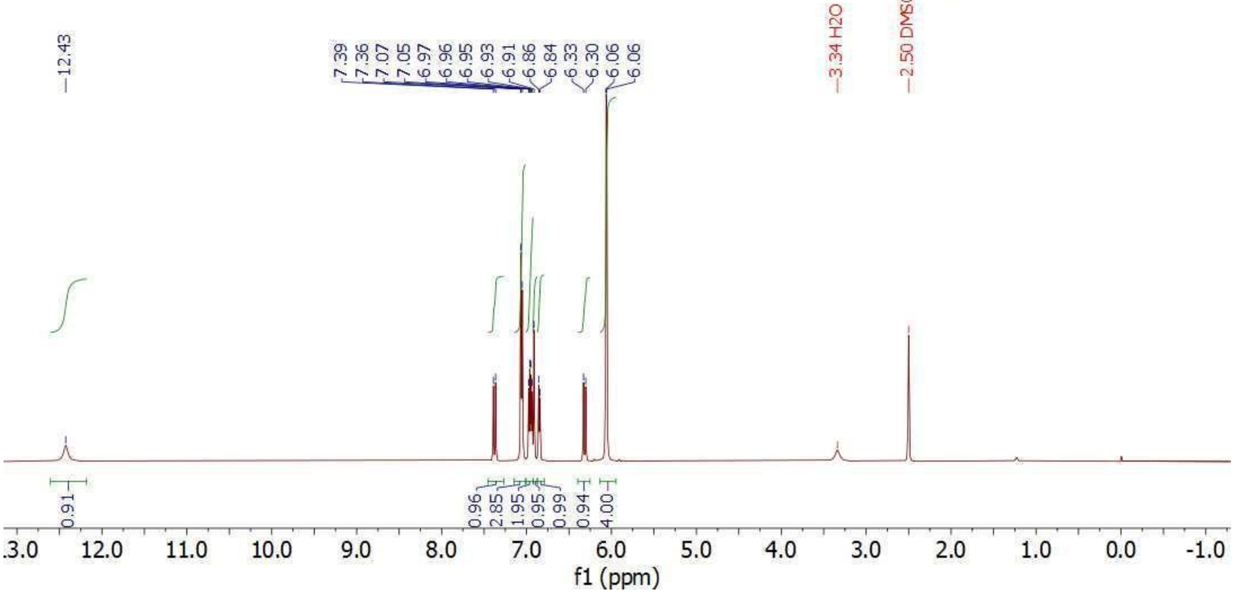

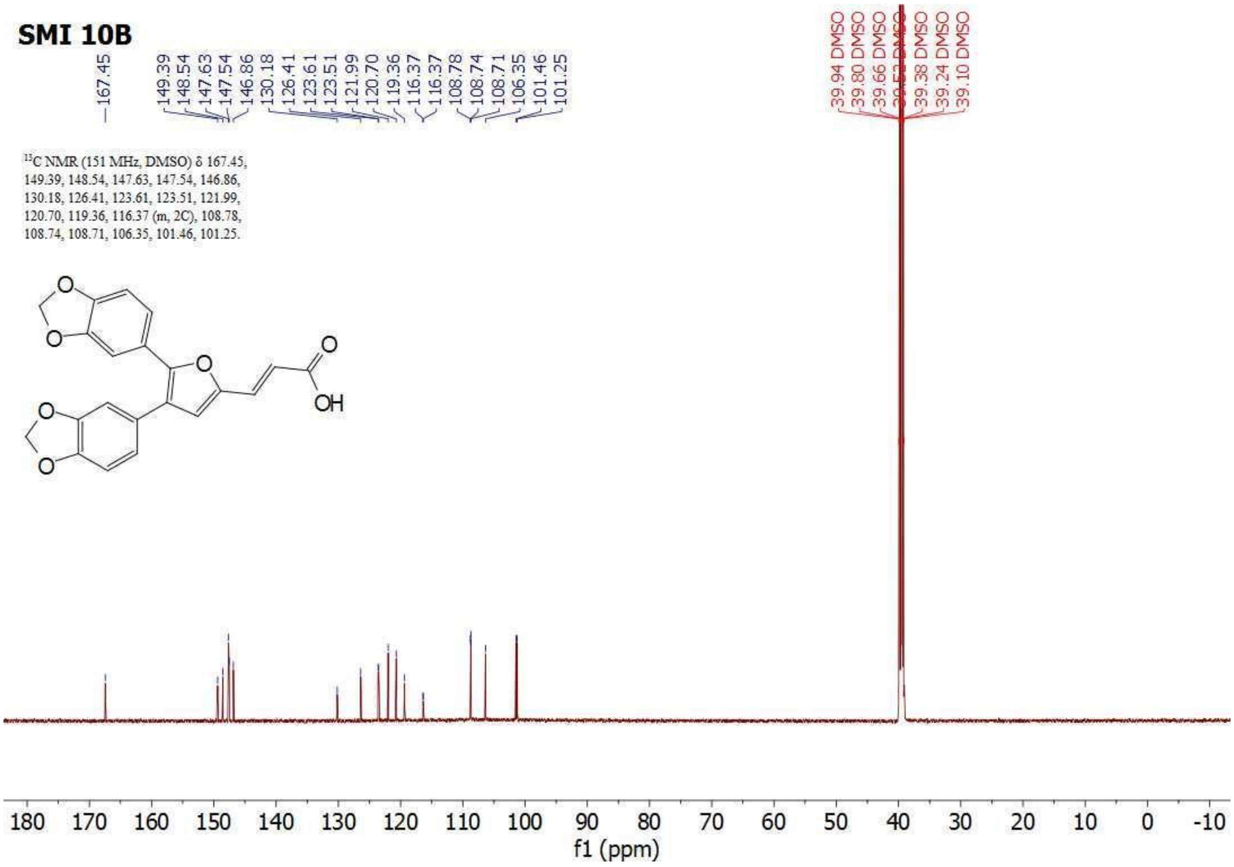

SMI-10B, solvent DMSO, UV abs. measured at 280 nm.

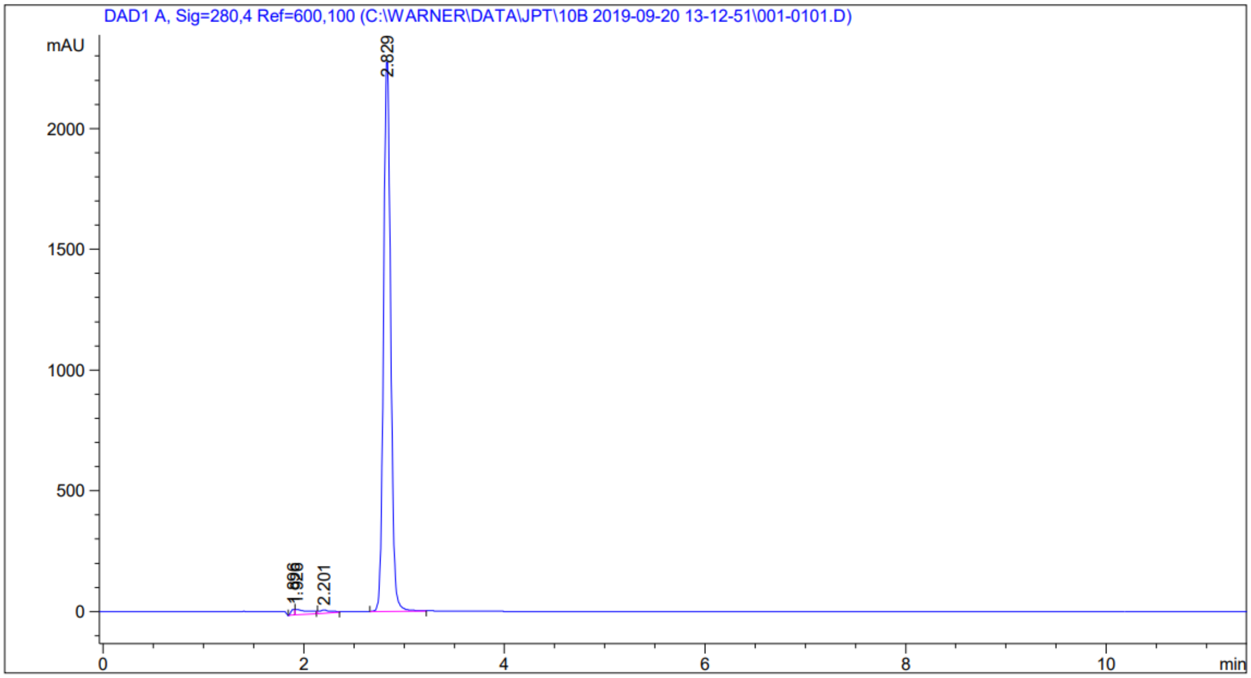

SMI 10C

^{1}H NMR (600 MHz, $CDCl_3$ with 0.05% v/v TMS) δ 8.08 – 8.04 (m, 2H), 7.63 – 7.56 (m, 2H), 7.55 – 7.47 (m, 3H), 7.15 (dd, J = 8.2, 1.8 Hz, 1H), 7.09 (d, J = 1.8 Hz, 1H), 6.91 – 6.81 (m, 3H), 6.80 (d, J = 8.7 Hz, 2H), 6.01 (s, 2H), 6.00 (s, 2H).

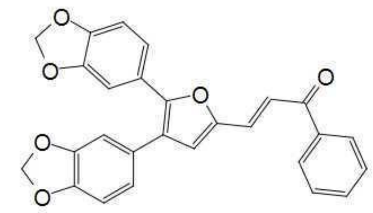

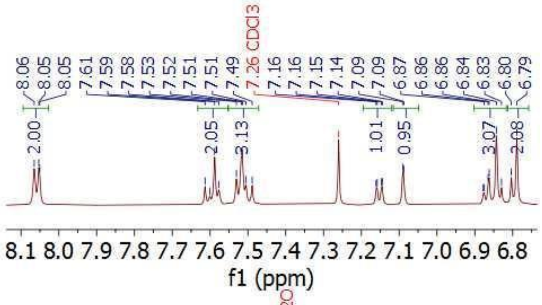

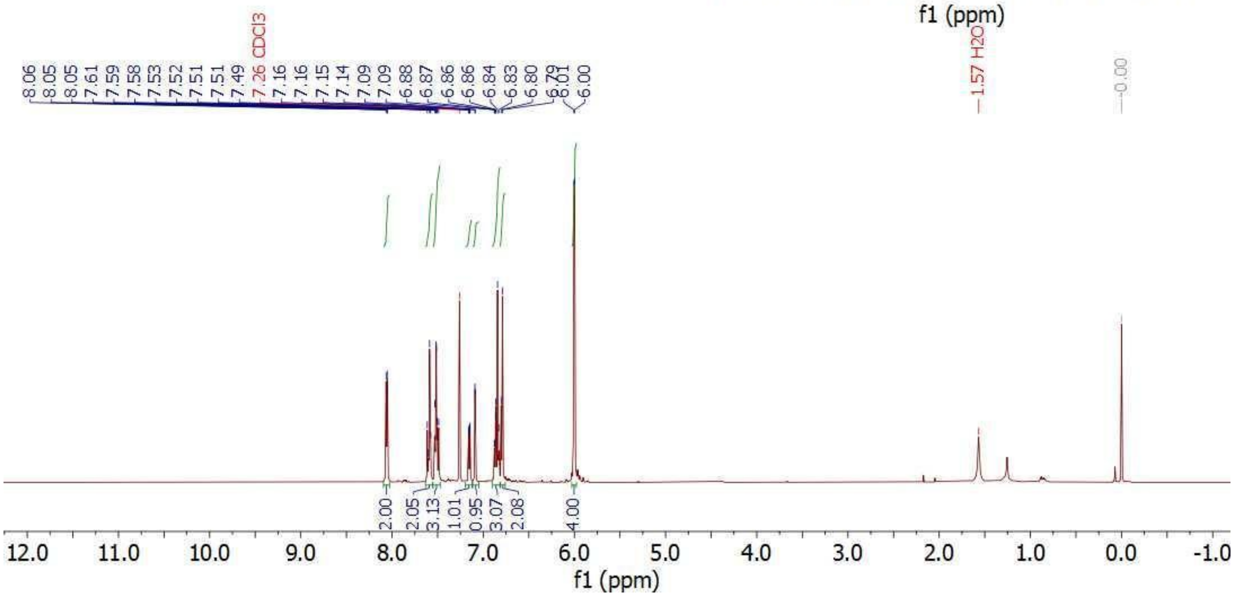

SMI 10C

^{13}C NMR (151 MHz, $CDCl_3$ with 0.05% v/v TMS) δ 189.86, 150.87, 149.77, 148.10, 147.90, 147.35, 138.50 – 138.38 (m, 2C), 132.88, 130.42, 128.77, 128.58, 127.01, 124.47, 124.32, 122.36, 121.37, 120.92, 118.87, 109.26, 108.91, 108.75, 107.23, 101.47, 101.37.

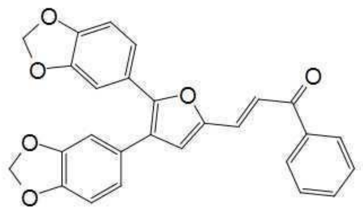

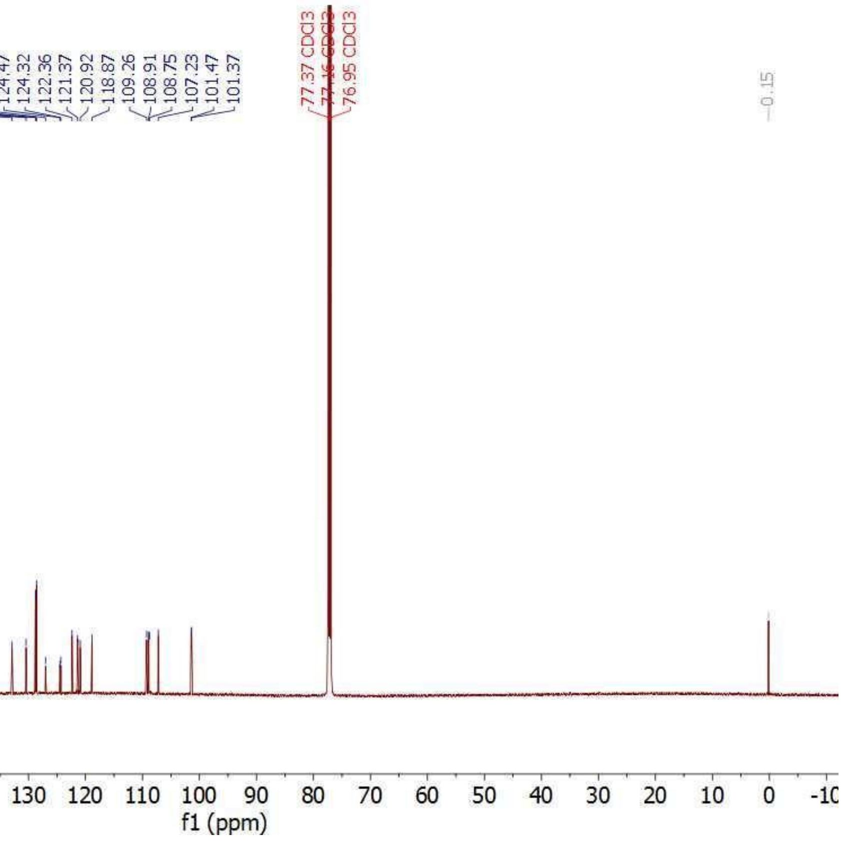

SMI-10C, solvent DMSO, UV abs. measured at 280 nm. Injection volume: 3.0 µL

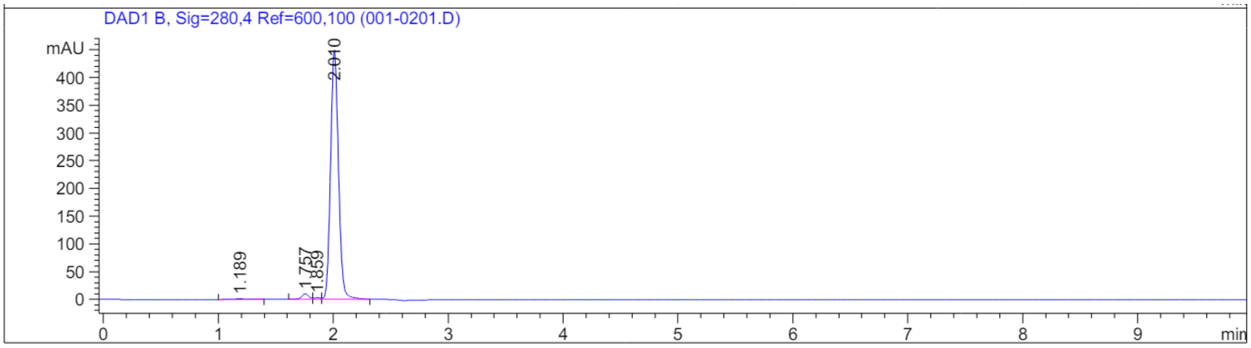

SMI 10D

^{1}H NMR (600 MHz, $CDCl_3$ with 0.05% v/v TMS) δ 7.42 (d, J = 15.6 Hz, 1H), 7.10 (dd, J = 8.2, 1.7 Hz, 1H), 7.02 (d, J = 1.7 Hz, 1H), 6.86 – 6.80 (m, 3H), 6.76 (d, J = 8.2 Hz, 1H), 6.65 (s, 1H), 6.38 (d, J = 15.6 Hz, 1H), 6.00 (s, 2H), 5.97 (s, 2H), 4.26 (q, J = 7.2 Hz, 2H), 1.34 (t, J = 7.1 Hz, 3H).

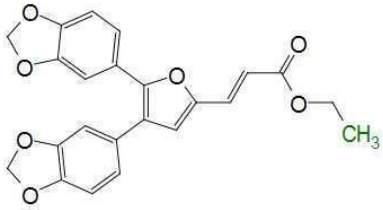

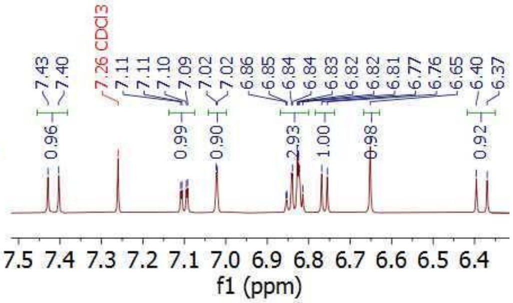

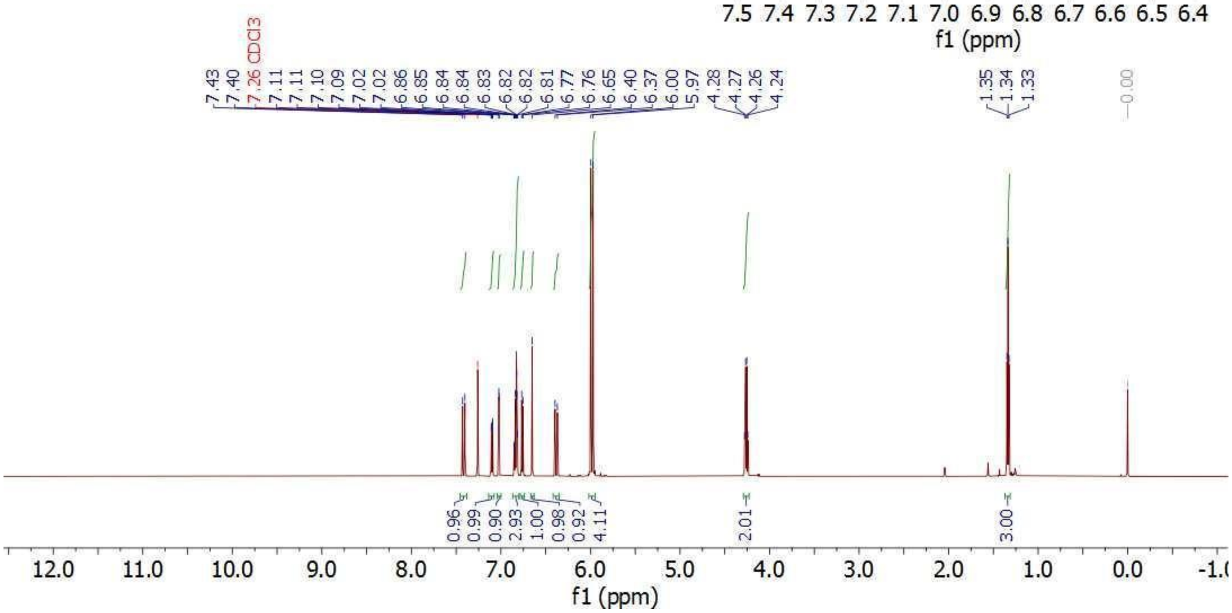

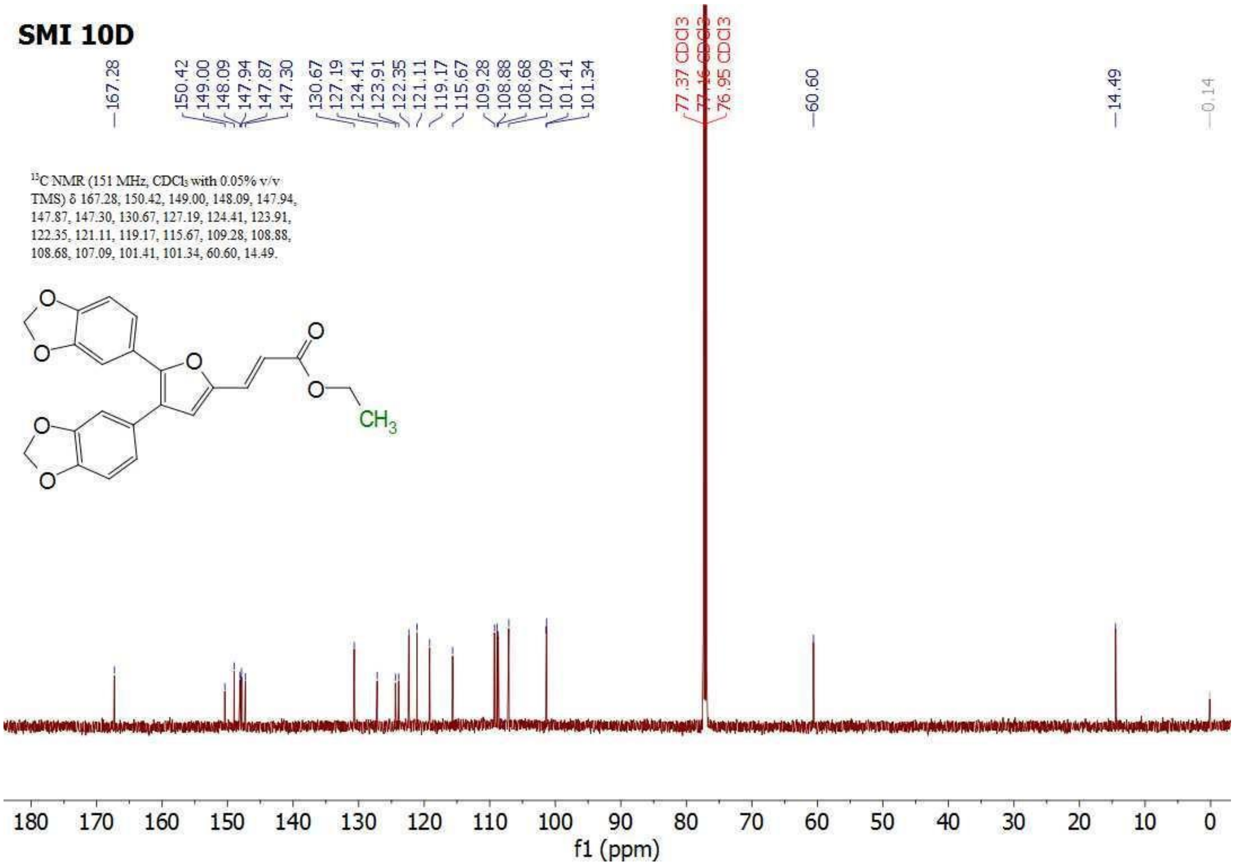

SMI-10D, solvent THF, UV abs. measured at 280 nm.

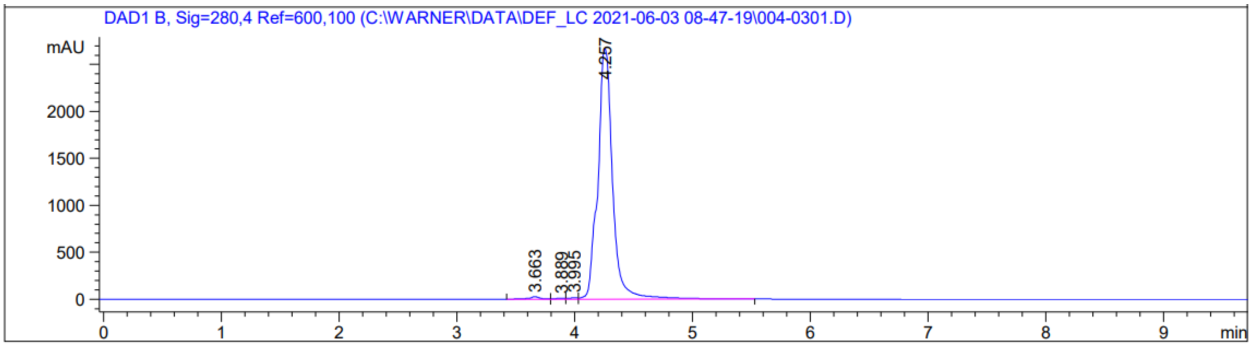

SMI 10E

^{1}H NMR (600 MHz, $CDCl_3$ with 0.05% v/v TMS) δ 7.63 (s, 1H), 7.57 (s, 1H), 7.34 – 7.22 (m, 2H), 7.11 (m, 3H), 7.06 (d, J = 1.7 Hz, 1H), 6.94 – 6.85 (m, 3H), 6.88 – 6.79 (m, 1H), 6.77 (d, J = 8.2 Hz, 1H), 6.65 (s, 1H), 6.00 (s, 2H), 5.97 (s, 2H).

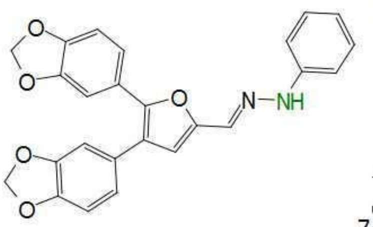

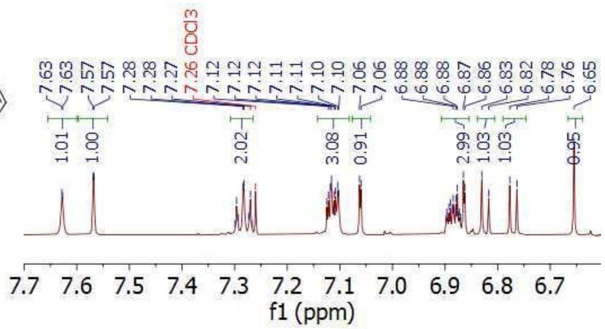

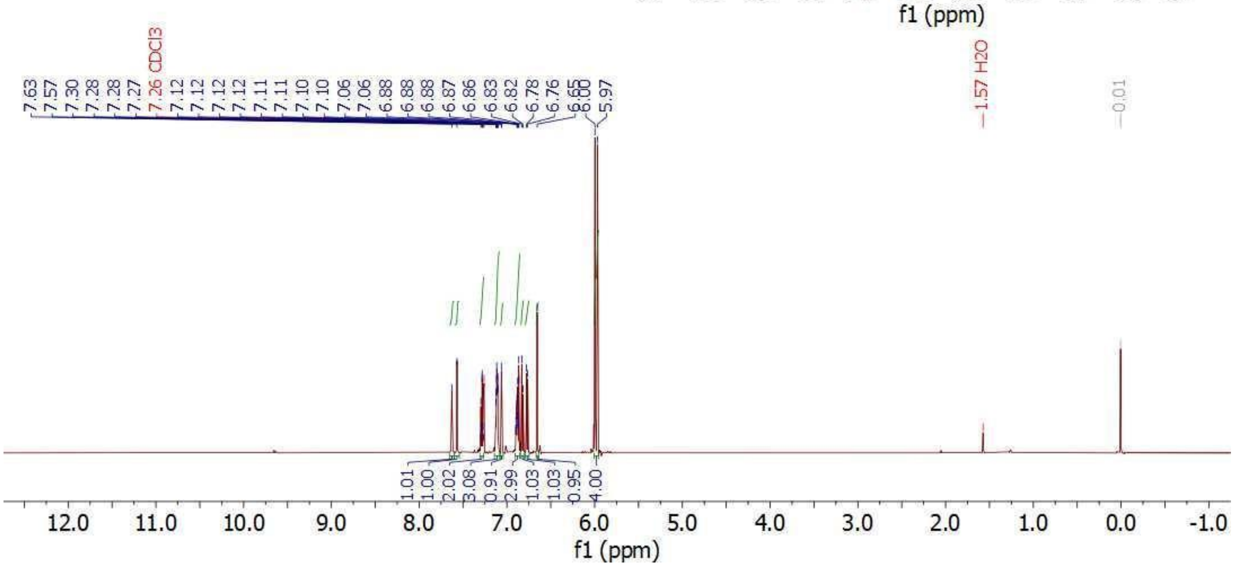

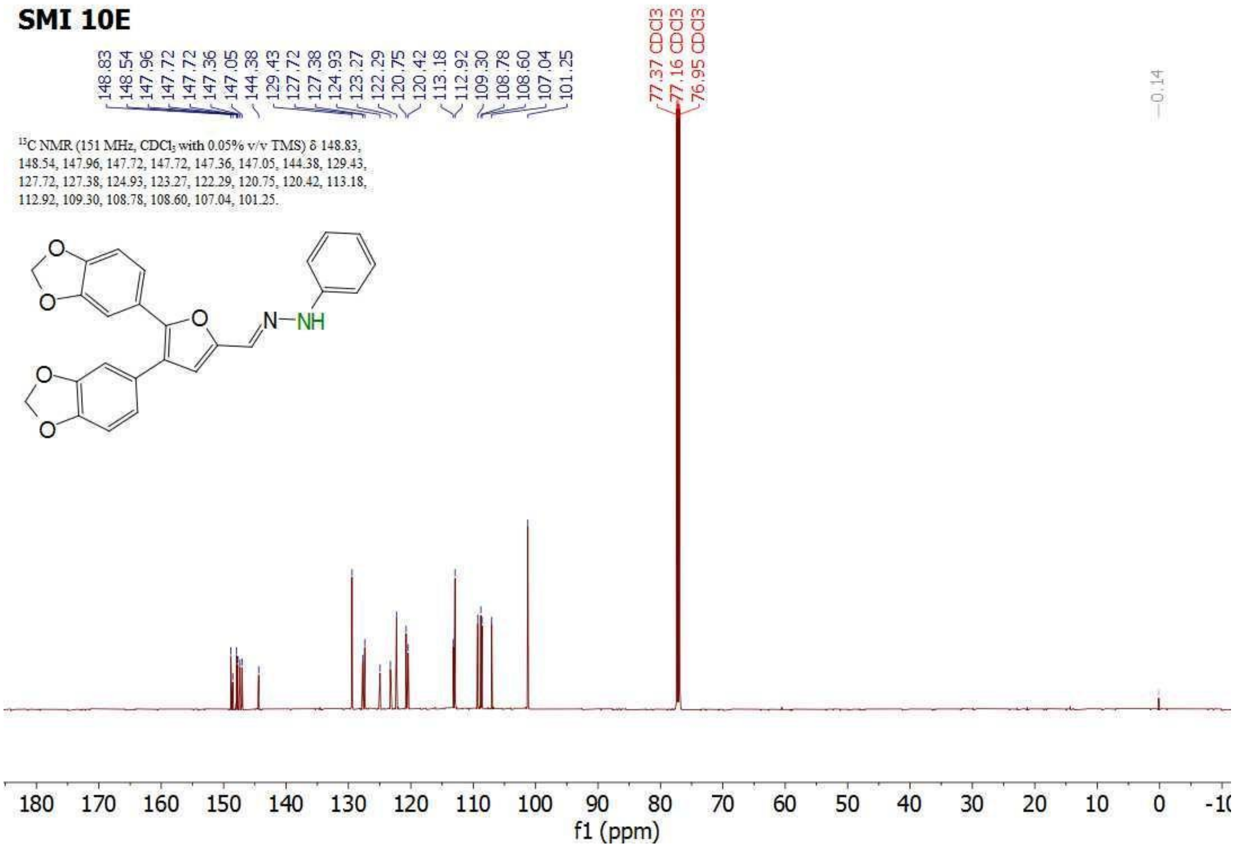

SMI-10E, solvent DMSO, UV abs. measured at 280 nm.

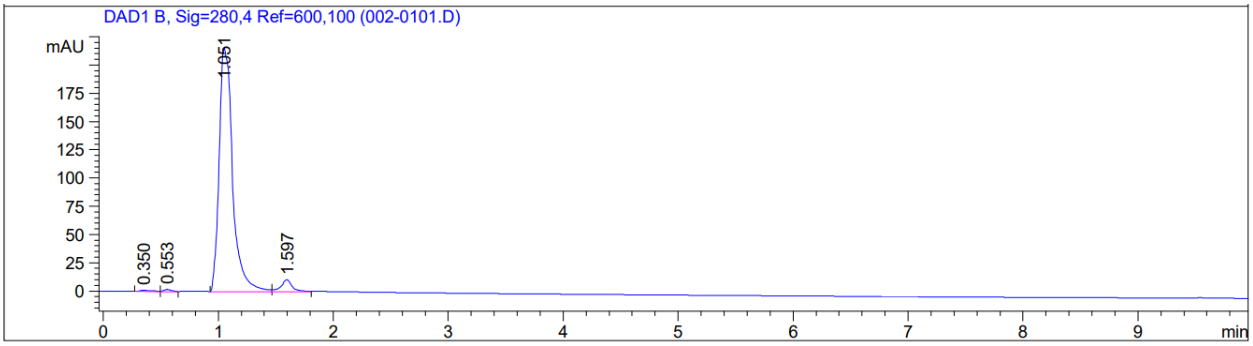

SMI 10F

^{1}H NMR (600 MHz, $CDCl_3$ with 0.05% v/v TMS) δ 7.37 – 7.29 (m, 4H), 7.27 – 7.21 (m, 1H), 7.02 (d, J = 8.2 Hz, 1H), 6.98 (s, 1H), 6.86 – 6.82 (m, 2H), 6.78 (d, J = 7.9 Hz, 1H), 6.72 (d, J = 8.2 Hz, 1H), 6.26 (s, 1H), 5.93 (s, 2H), 5.90 (s, 2H), 3.85 (s, 2H), 3.81 (s, 2H), 1.76 (s, 1H).

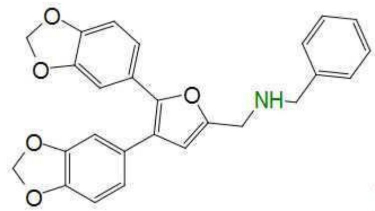

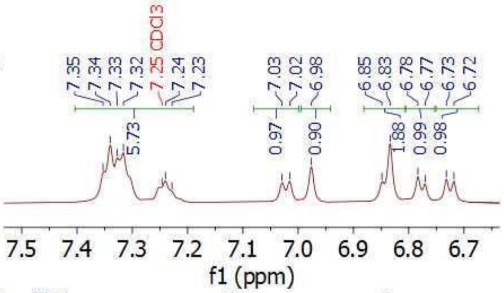

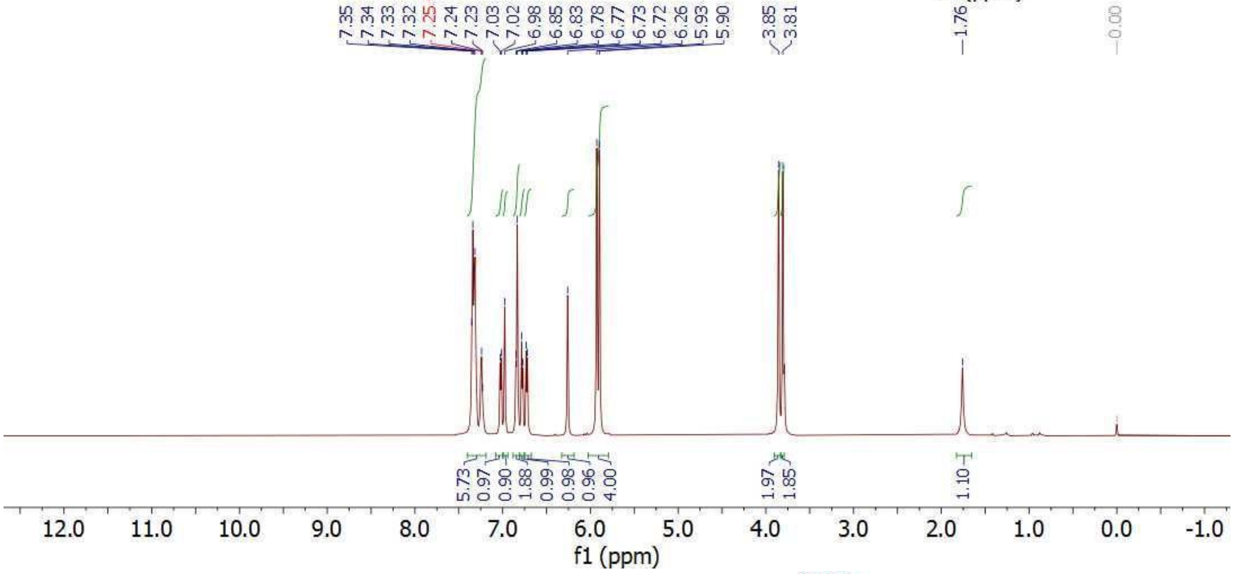

SMI 10F

^{13}C NMR (151 MHz, $CDCl_3$ with 0.05% v/v TMS) δ 128.47, 123.88, 123.67, 123.39, 123.02, 122.77, 116.04, 104.56, 104.40, 104.24, 103.18, 101.43, 98.11, 97.66, 96.44, 87.47, 85.20, 84.70, 84.51, 83.03, 77.16, 29.07, 21.56.

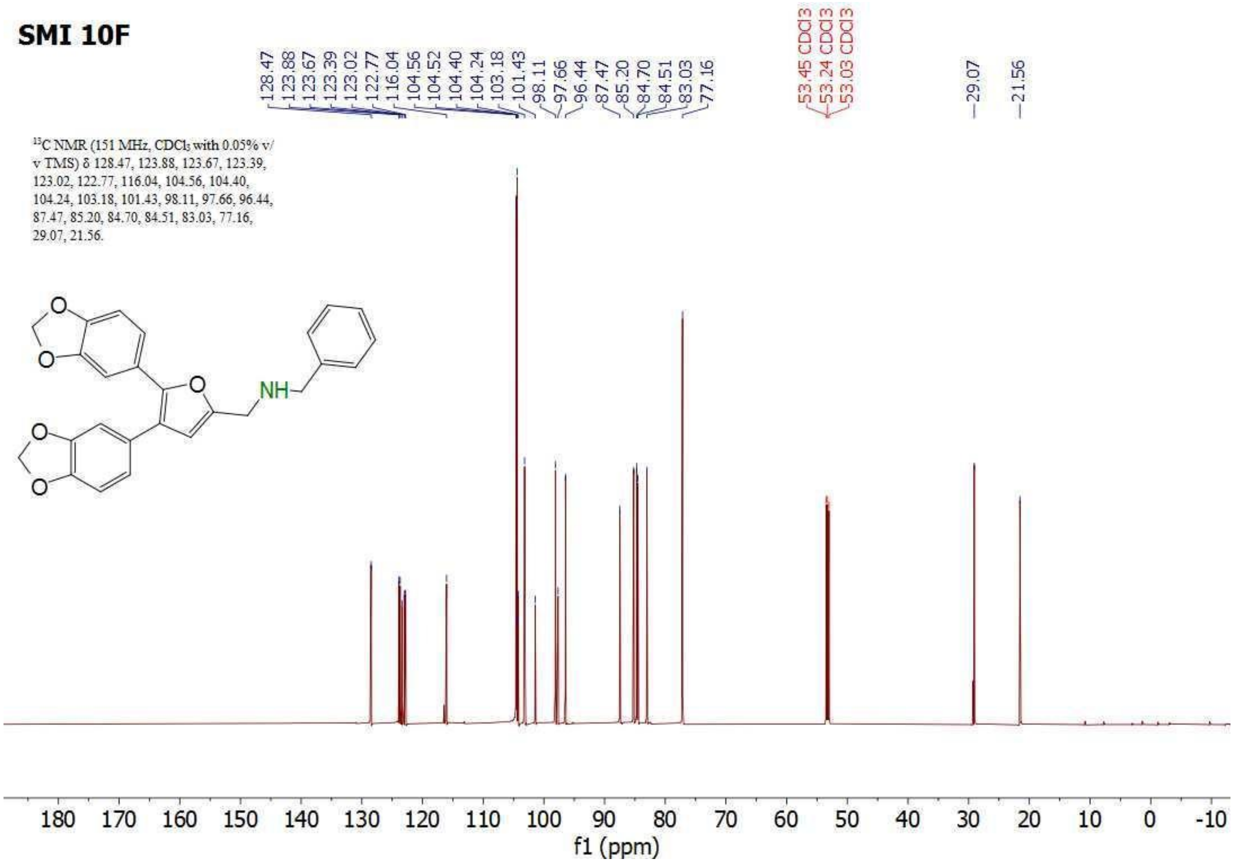

SMI-10F, solvent DMSO, UV abs. measured at 280 nm.

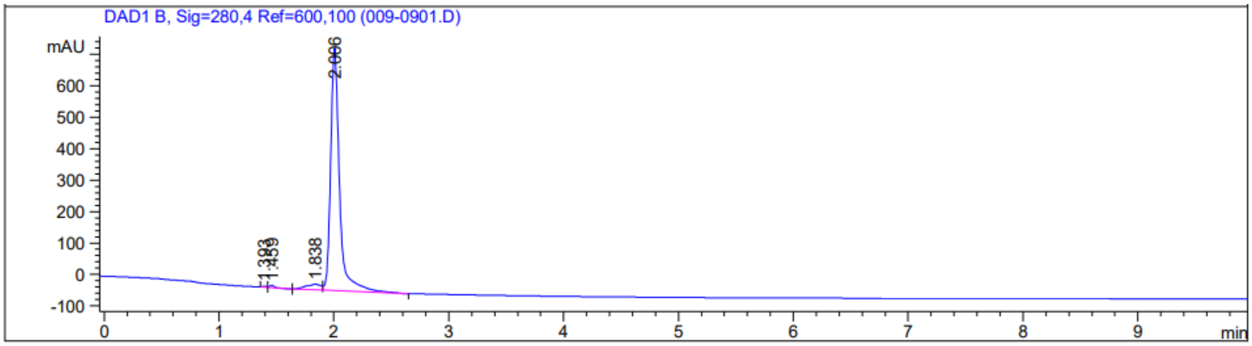

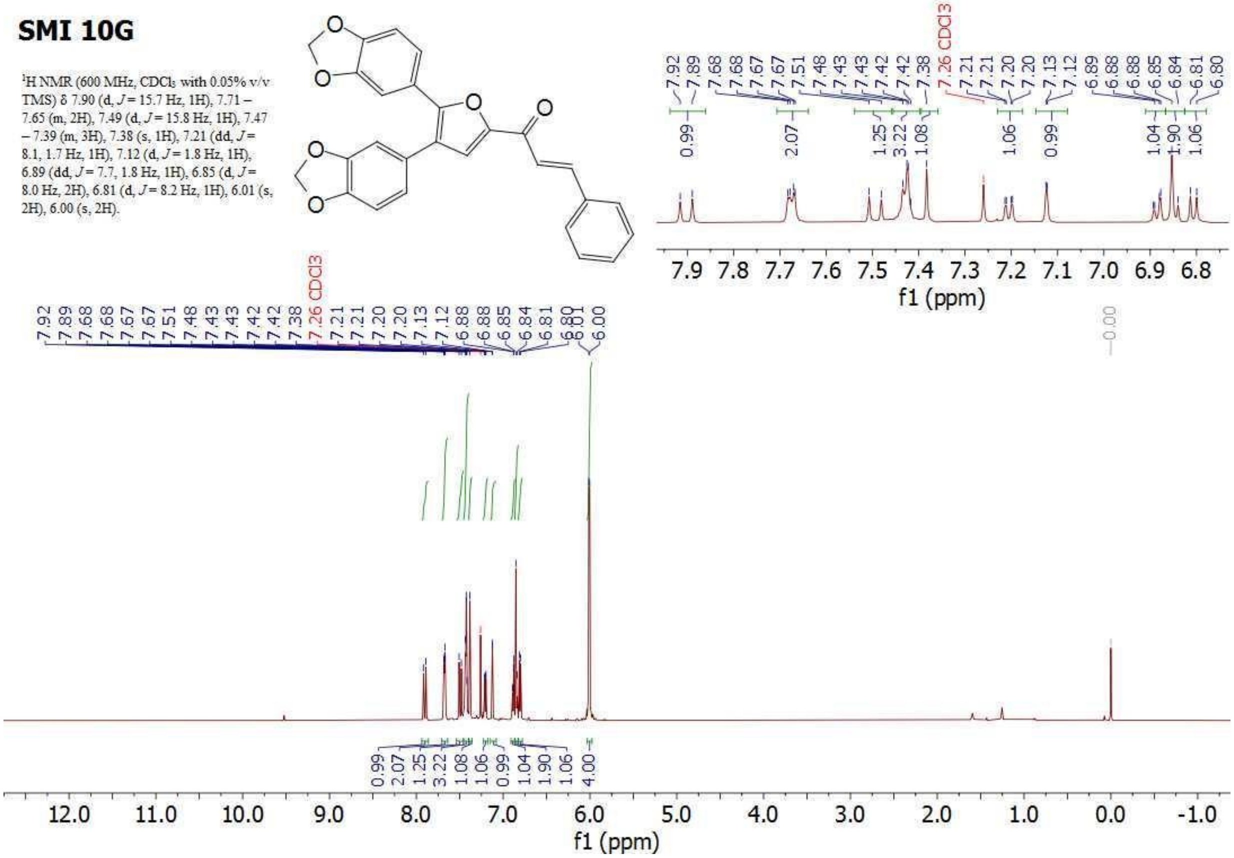

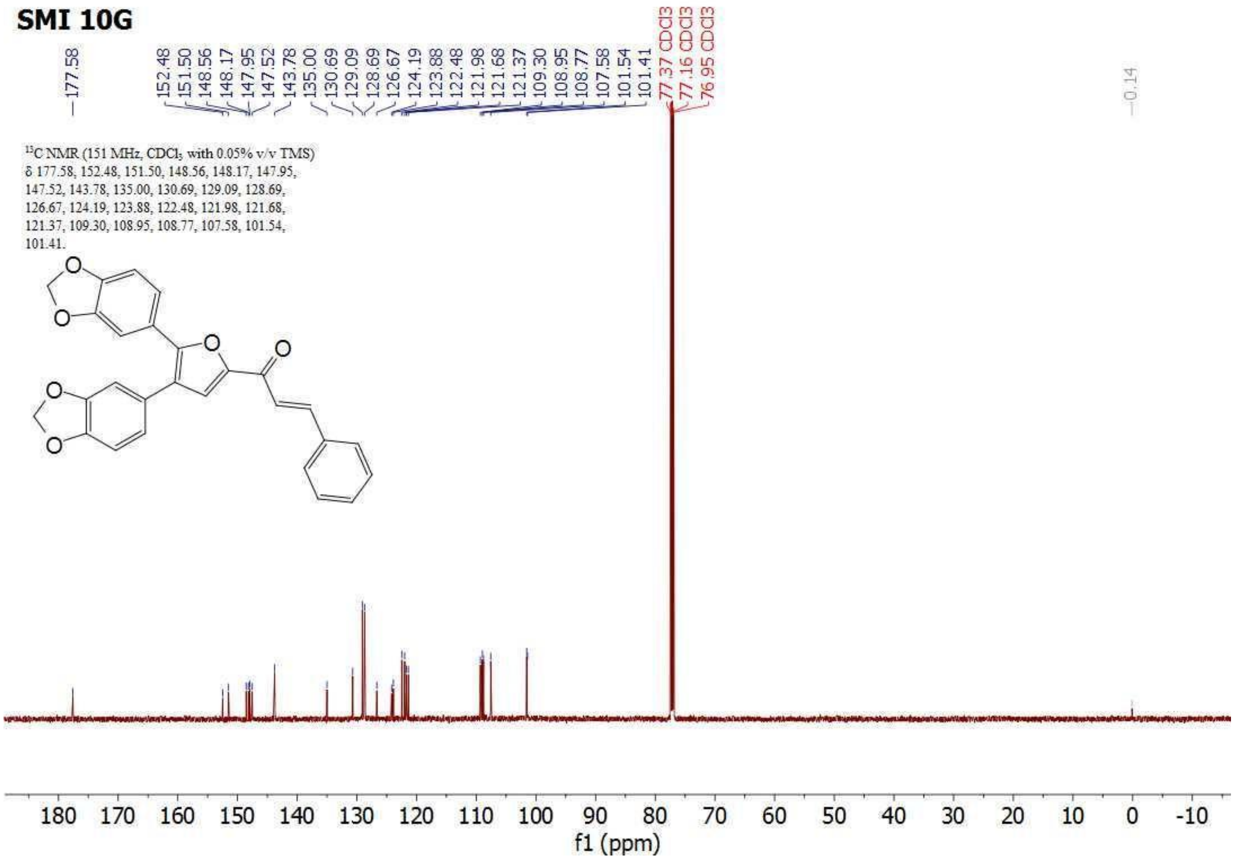

SMI-10G, solvent DMSO, UV abs. measured at 254 nm. Injection volume: 3.0 uL

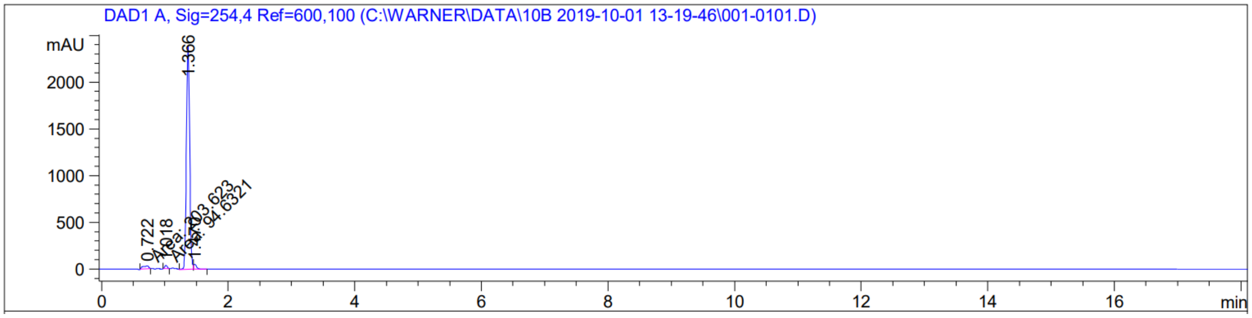

SMI 10H

^{1}H NMR (600 MHz, $CDCl_3$ with 0.05% v/v TMS) δ 7.04 (dd, J = 8.2, 1.8 Hz, 1H), 6.98 (d, J = 1.8 Hz, 1H), 6.86 – 6.80 (m, 2H), 6.80 (d, J = 7.9 Hz, 1H), 6.75 (d, J = 8.2 Hz, 1H), 6.38 (s, 1H), 5.98 (s, 2H), 5.95 (s, 2H), 4.65 (s, 2H), 1.90 (s, 1H).

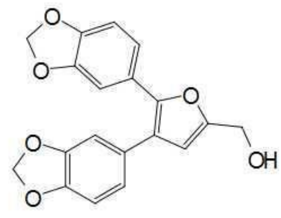

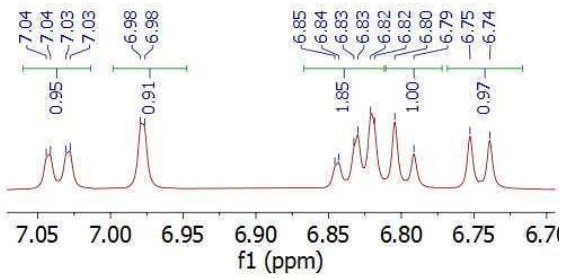

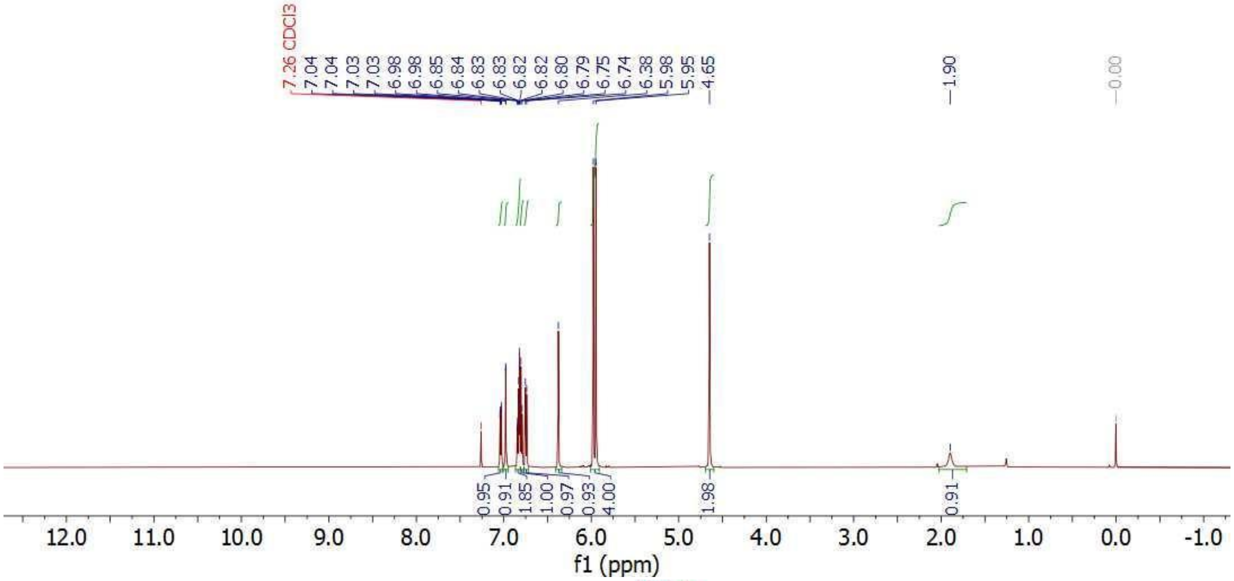

SMI 10H

^{13}C NMR (151 MHz, $CDCl_3$ with 0.05% v/v TMS) δ 152.36, 148.23, 147.93, 147.72, 147.27, 146.91, 127.97, 125.15, 122.19, 121.81, 120.66, 112.29, 109.22, 108.75, 108.56, 107.13, 101.22, 101.20, 57.72.

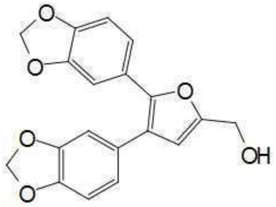

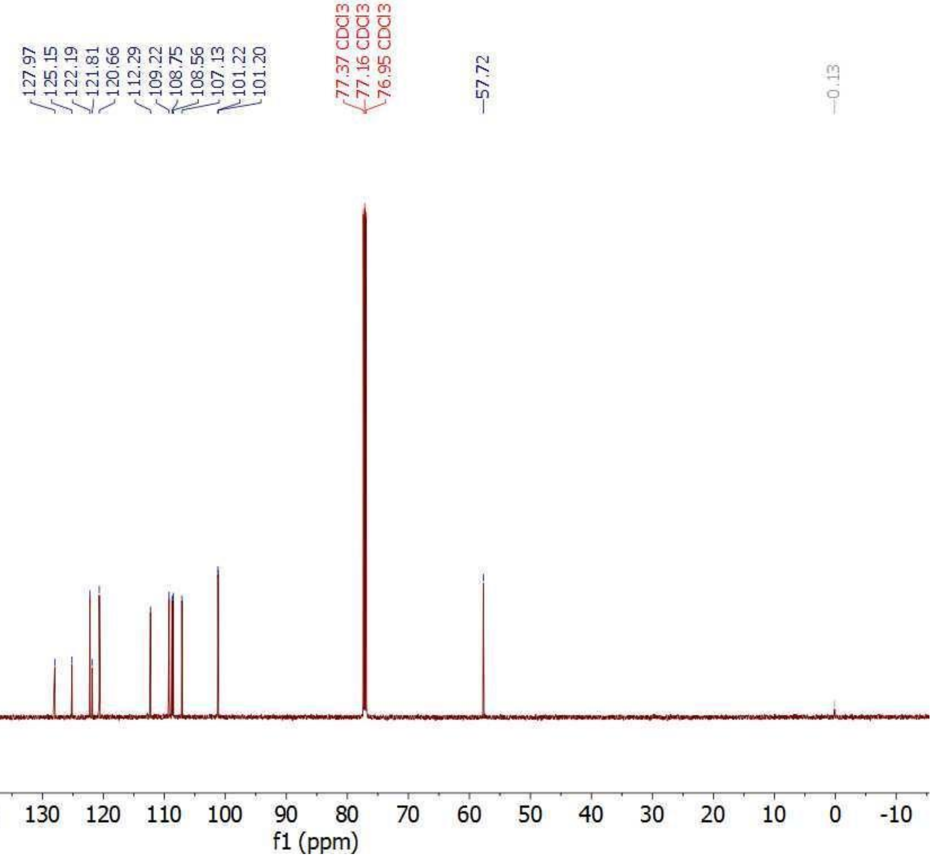

SMI-10H, solvent DMSO, UV abs. measured at 280 nm.

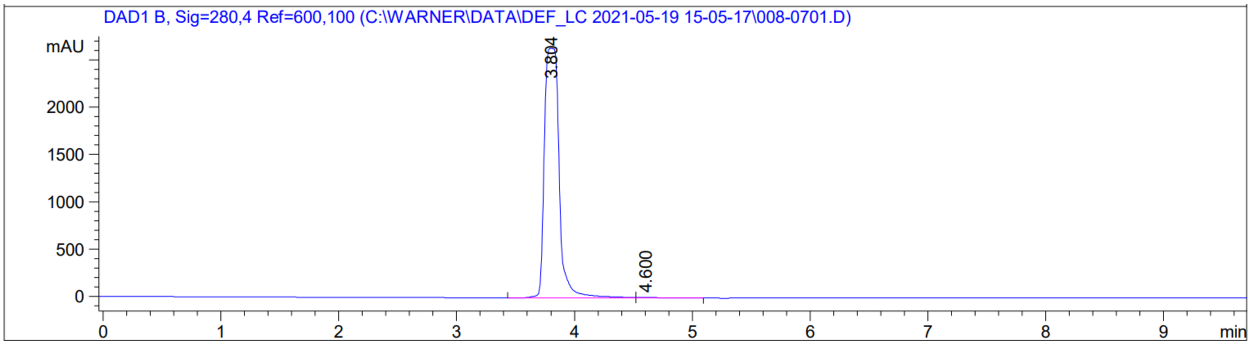

SMI 10I

^{1}H NMR (600 MHz, DMSO) δ 13.17 (s, 1H), 7.36 (d, J = 1.0 Hz, 1H), 7.03 (dd, J = 8.2, 1.7 Hz, 1H), 7.00 – 6.93 (m, 4H), 6.86 (dd, J = 8.1, 1.7 Hz, 1H), 6.07 (s, 2H), 6.06 (s, 2H).

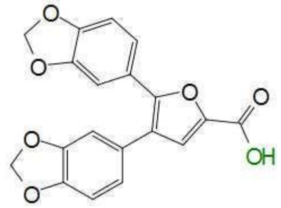

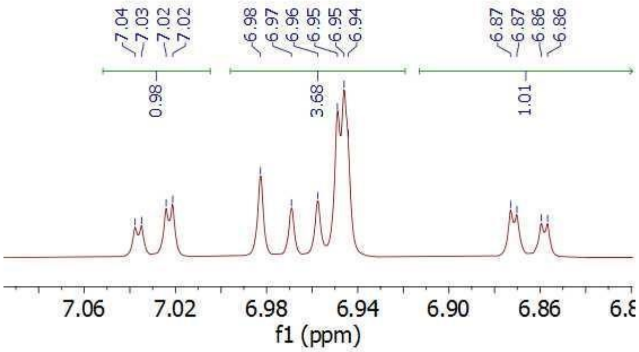

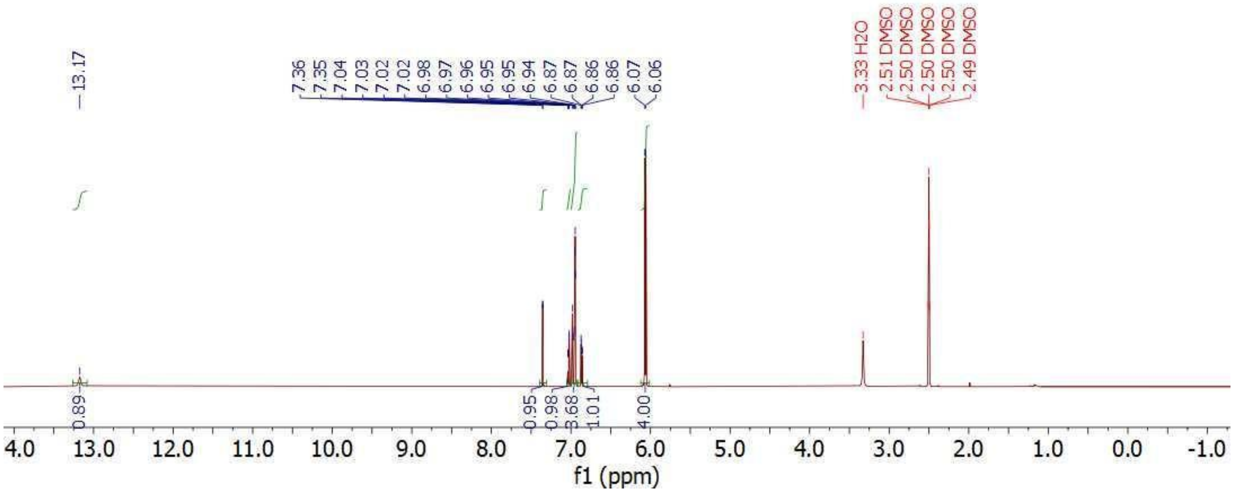

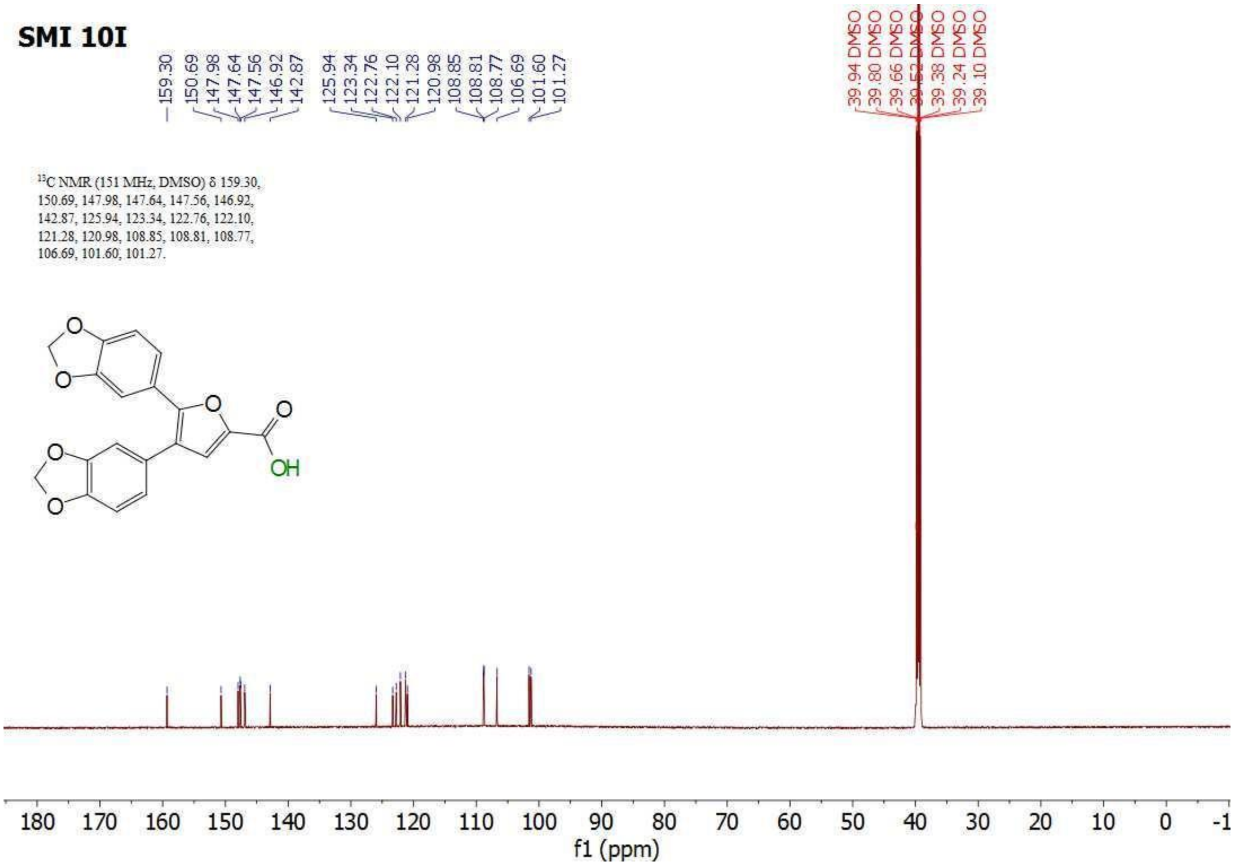

SMI-10I, solvent THF, UV abs. measured at 280 nm.

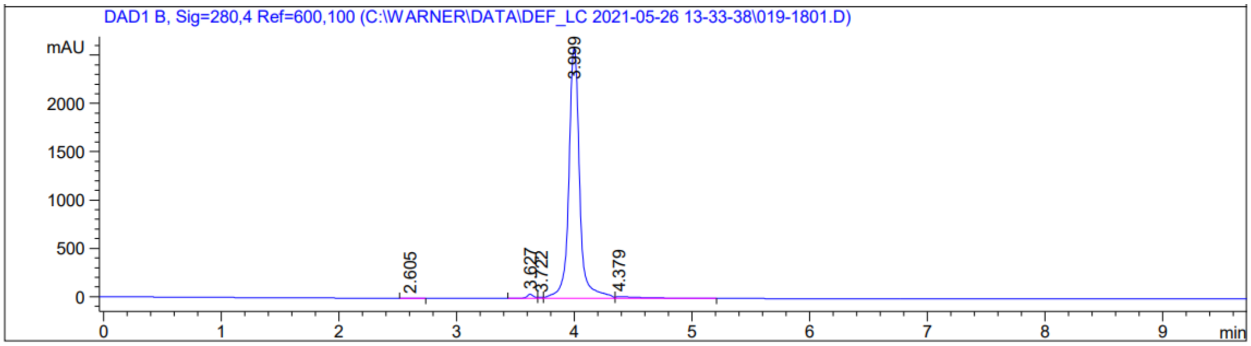

SMI 10J

^{1}H NMR (600 MHz, $CDCl_3$ with 0.05% v/v TMS) δ 7.41 – 7.34 (m, 4H), 7.34 – 7.28 (m, 1H), 7.25 (s, 1H), 7.22 (s, *J* = 0.7 Hz, 1H), 7.03 (dd, *J* = 8.3, 1.7 Hz, 1H), 6.95 (d, *J* = 1.7 Hz, 1H), 6.87 – 6.79 (m, 3H), 6.76 (d, *J* = 8.2 Hz, 1H), 6.67 (t, *J* = 5.9 Hz, 1H), 5.99 (s, 2H), 5.97 (s, 2H), 4.66 (d, *J* = 5.9 Hz, 2H).

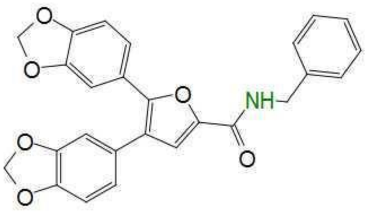

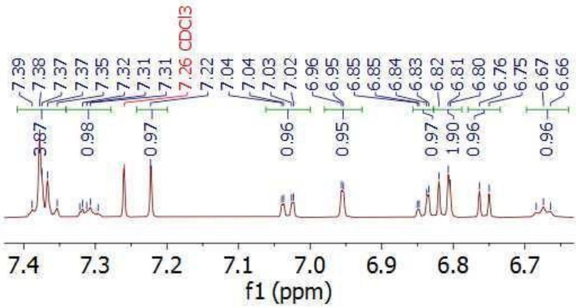

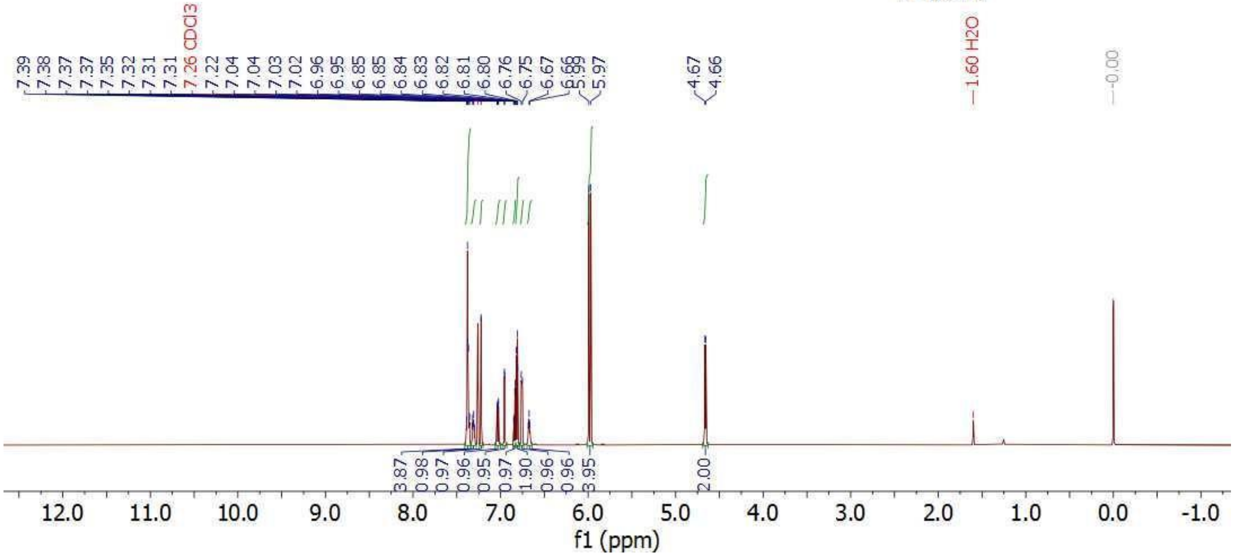

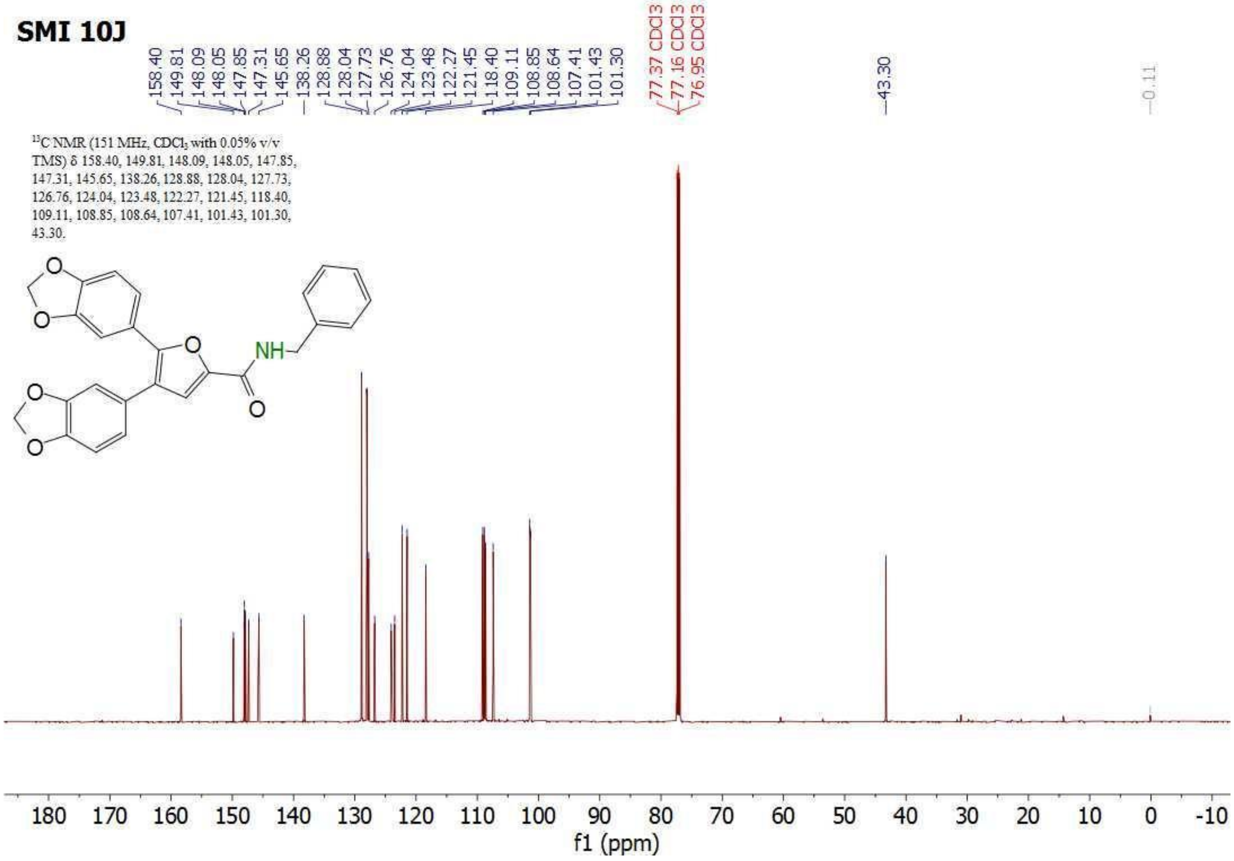

SMI-10J, solvent DMSO, UV abs. measured at 280 nm.

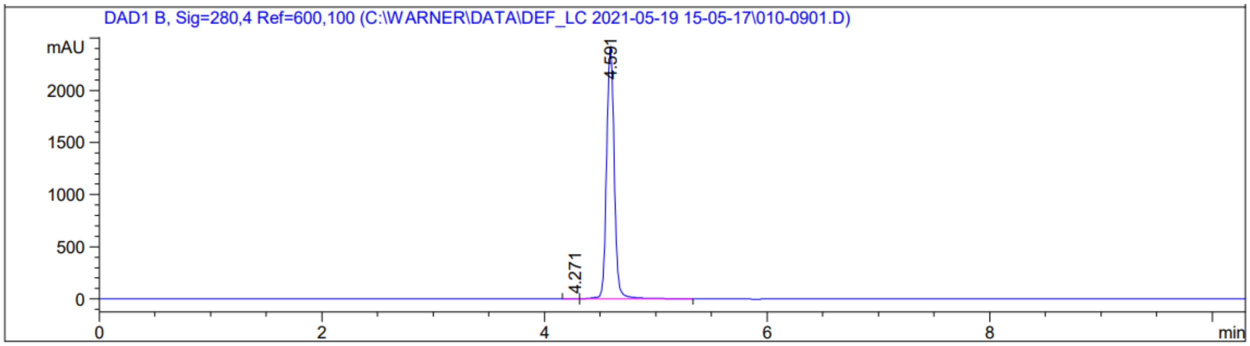

SMI 10B1

^{1}H NMR (600 MHz, $CDCl_3$) δ 7.39 (s, 0H), 7.15 – 7.09 (m, 3H), 7.00 (d, J = 8.2 Hz, 1H), 6.97 (dd, J = 5.3, 3.3 Hz, 2H), 6.94 (dd, J = 8.2, 2.0 Hz, 1H), 6.32 (d, J = 15.7 Hz, 1H), 3.77 (d, J = 5.5 Hz, 6H), 3.69 (s, 3H), 3.63 (s, 3H).

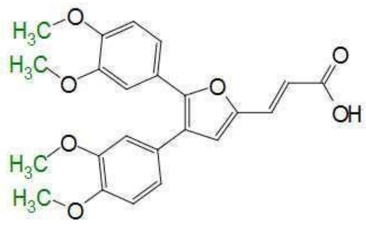

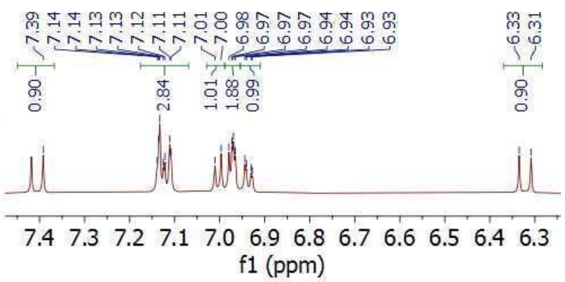

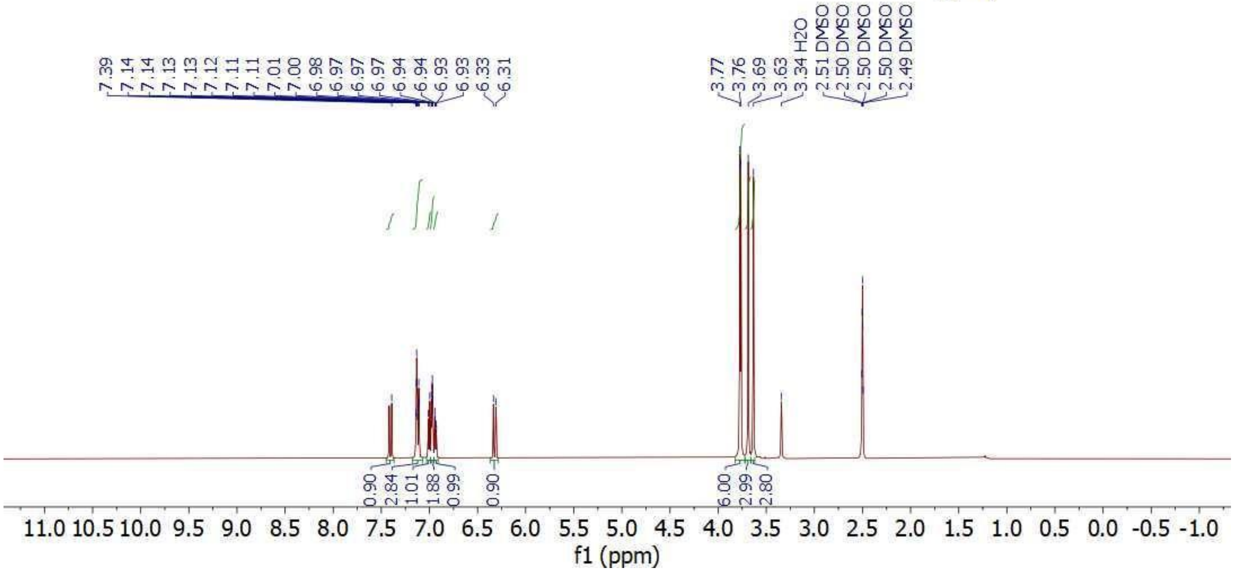

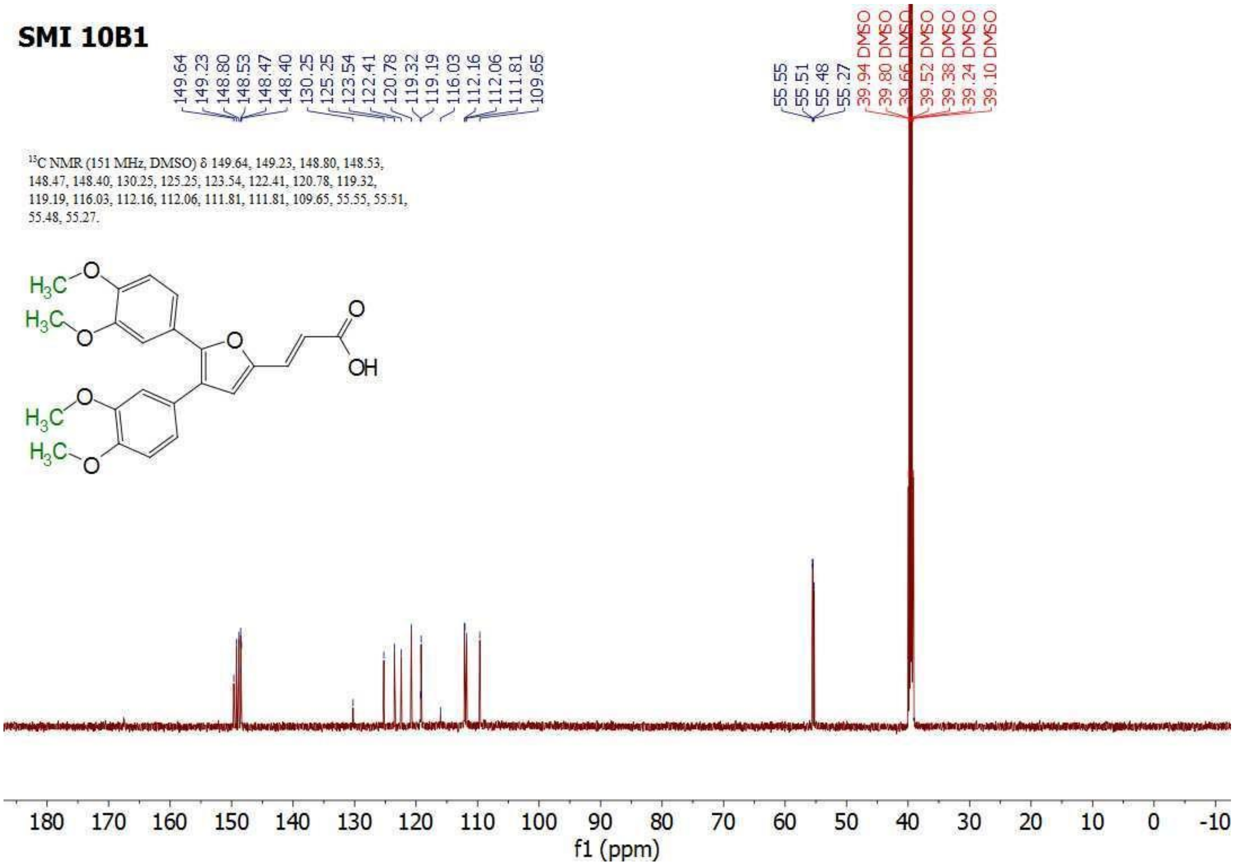

SMI-10B1, solvent DMSO, UV abs. measured at 280 nm.

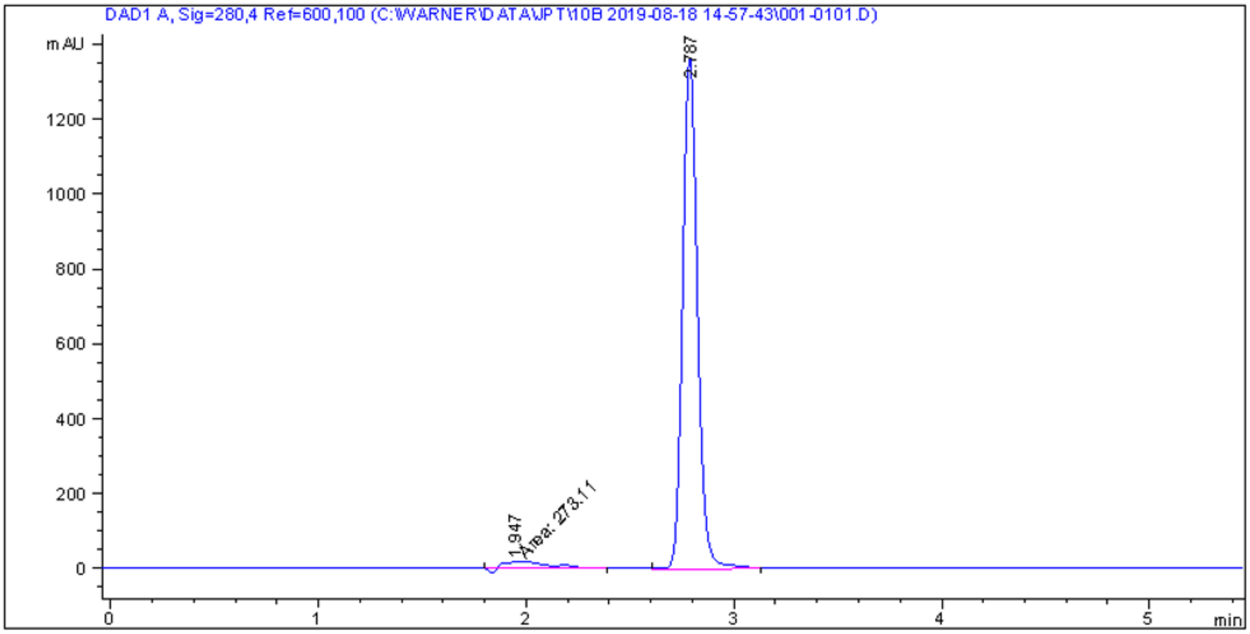

SMI 10B2

^1H NMR (300 MHz, DMSO) δ 12.35 (s, 1H), 9.14 (d, J = 21.4 Hz, 2H), 7.39 (d, J = 15.7 Hz, 1H), 7.05 – 6.87 (m, 5H), 6.81 – 6.72 (m, 2H), 6.23 (d, J = 15.7 Hz, 1H), 3.79 (s, 3H), 3.77 (s, 3H).

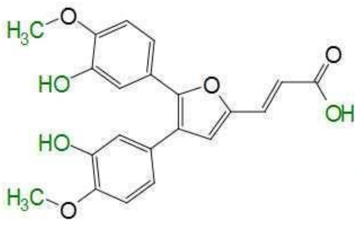

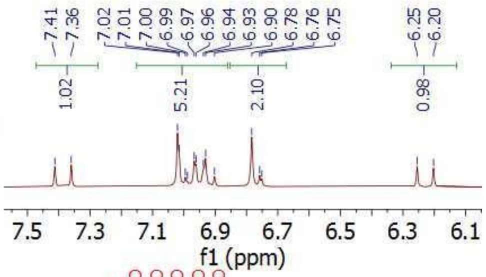

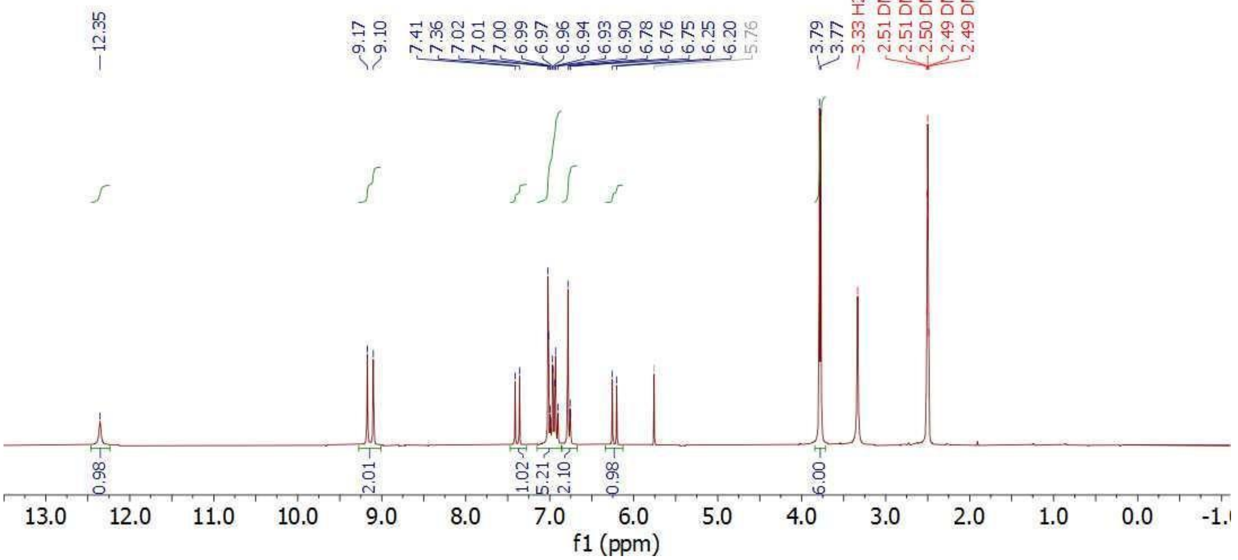

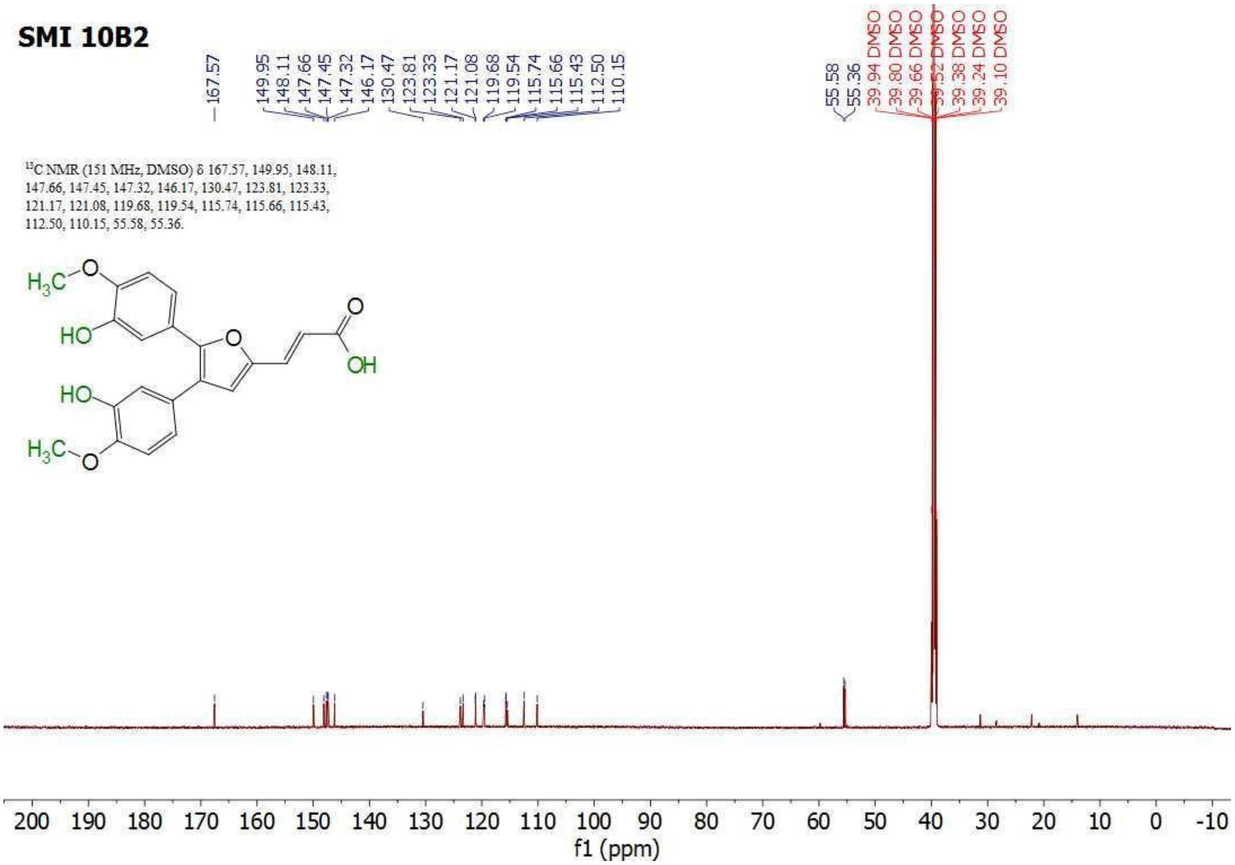

SMI-10B2, solvent DMSO, UV abs. measured at 280 nm.

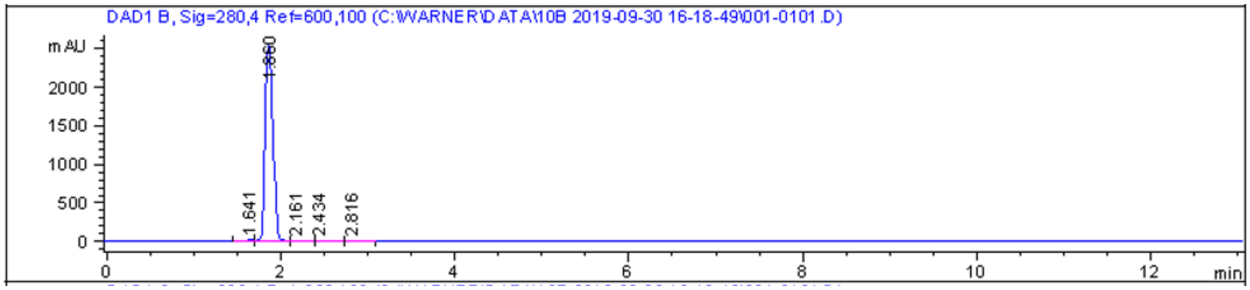

SMI 10B3

^{1}H NMR (600 MHz, DMSO) δ 12.43 (s, 1H), 9.45 (s, 1H), 9.18 (s, 1H), 7.37 (d, J = 15.7 Hz, 1H), 7.08 (d, J = 7.7 Hz, 2H), 7.06 – 7.01 (m, 1H), 6.93 (s, 1H), 6.81 (s, 2H), 6.77 (d, J = 8.4 Hz, 1H), 6.28 (d, J = 15.7 Hz, 1H), 3.70 (s, 3H), 3.64 (s, 3H).

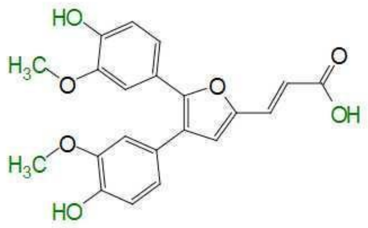

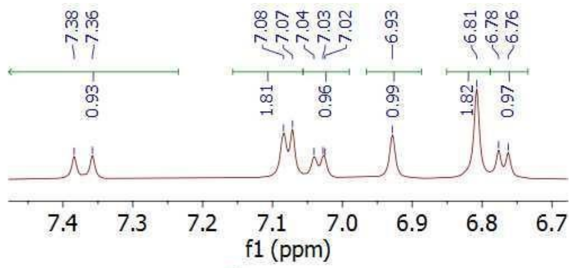

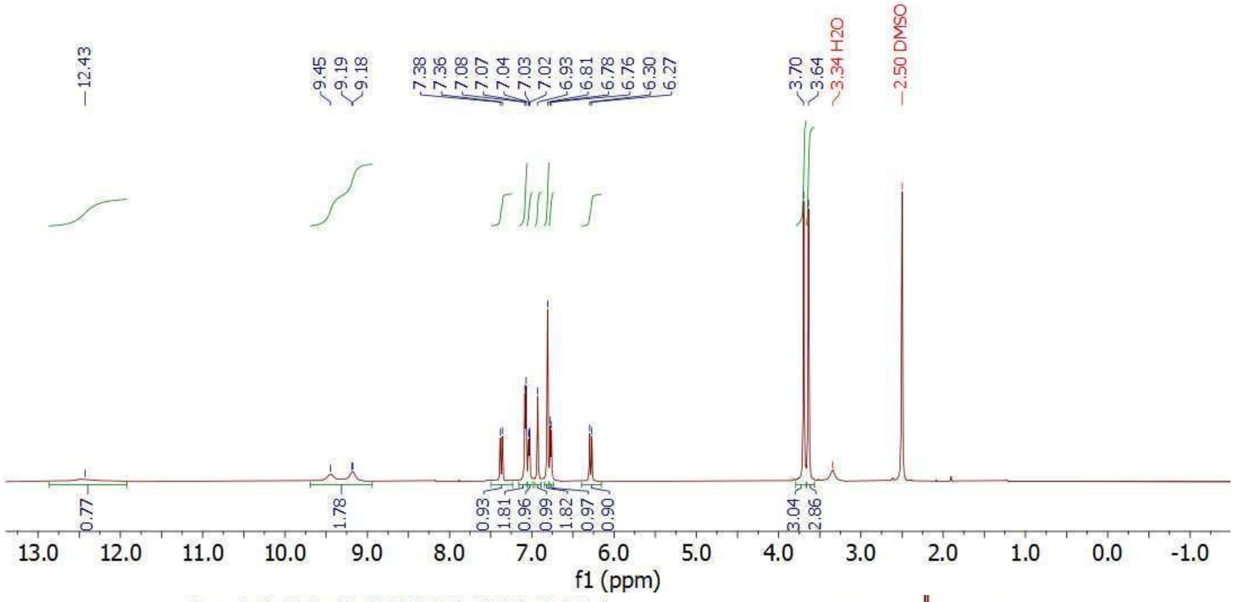

SMI 10B3

^{13}C NMR (151 MHz, DMSO) δ 167.63, 149.87, 148.17, 147.65, 147.44, 147.31, 146.17, 130.23, 123.82, 123.30, 121.19, 121.08, 119.67 – 119.36 (m, 2C), 115.83 – 115.60 (m, 3C), 112.50, 110.14, 55.58, 55.36.

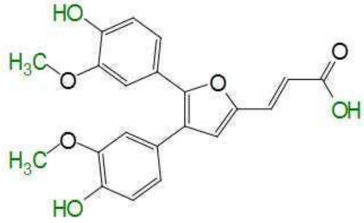

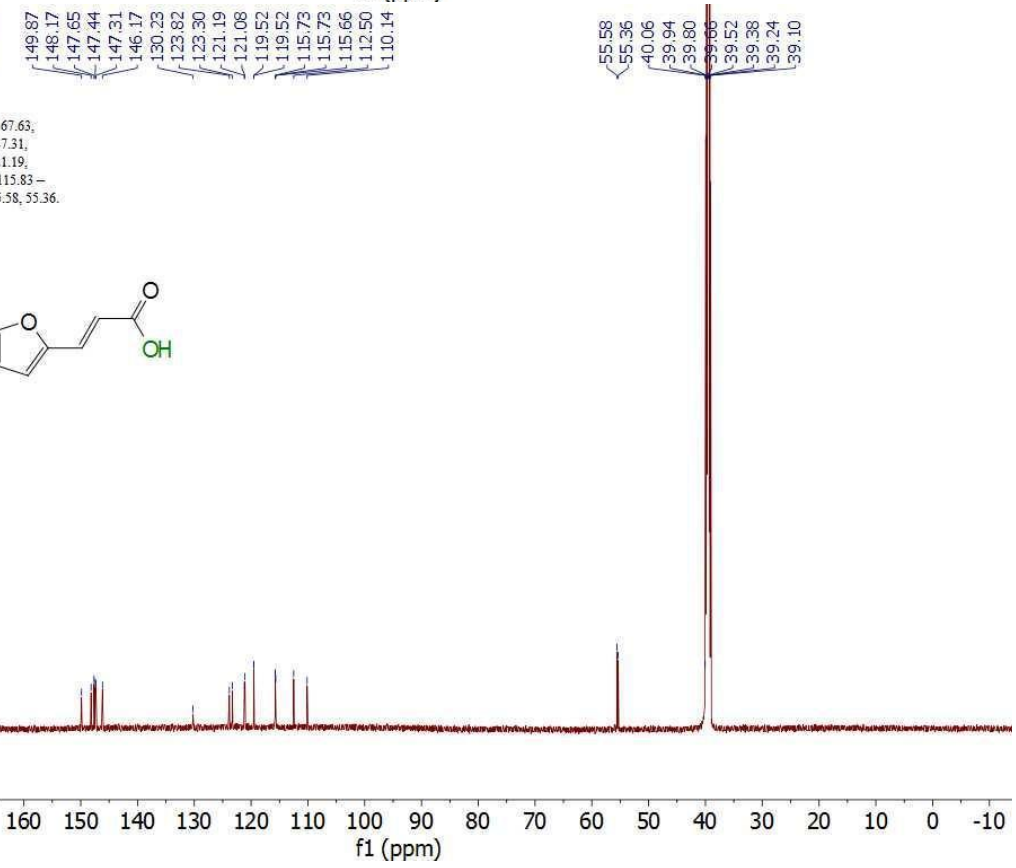

SMI-10B3, solvent DMSO, UV abs. measured at 280 nm.

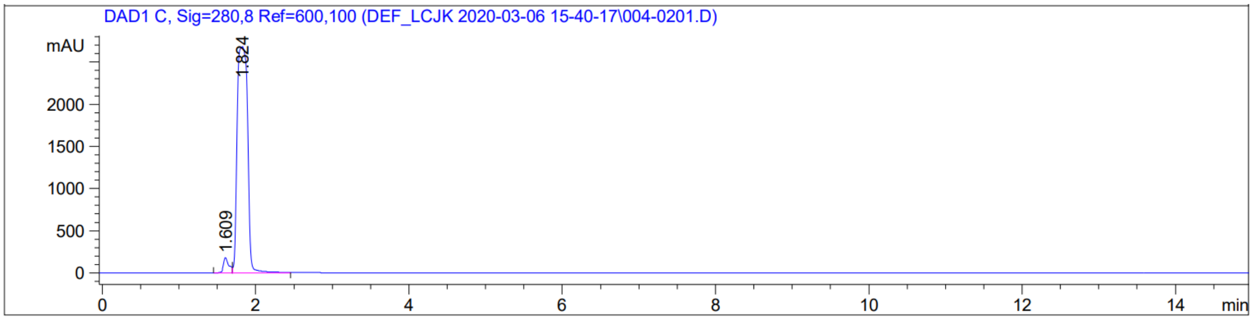

SMI 10B4

^{1}H NMR (300 MHz, DMSO) δ 7.67 (d, J = 15.7 Hz, 1H), 7.54 (s, 1H), 6.91 (t, J = 8.1 Hz, 2H), 6.86 – 6.64 (m, 4H), 6.19 (d, J = 15.7 Hz, 1H), 6.05 (s, 2H), 6.03 (s, 2H).

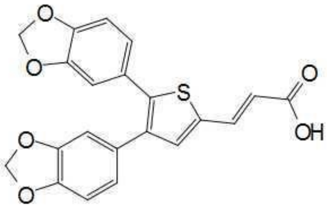

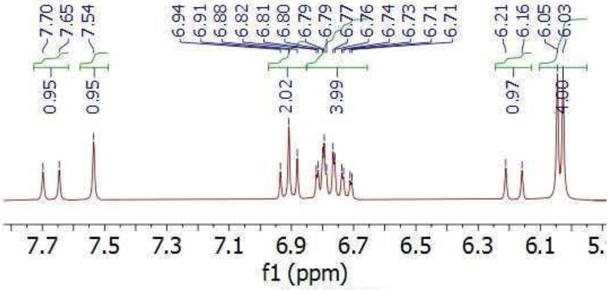

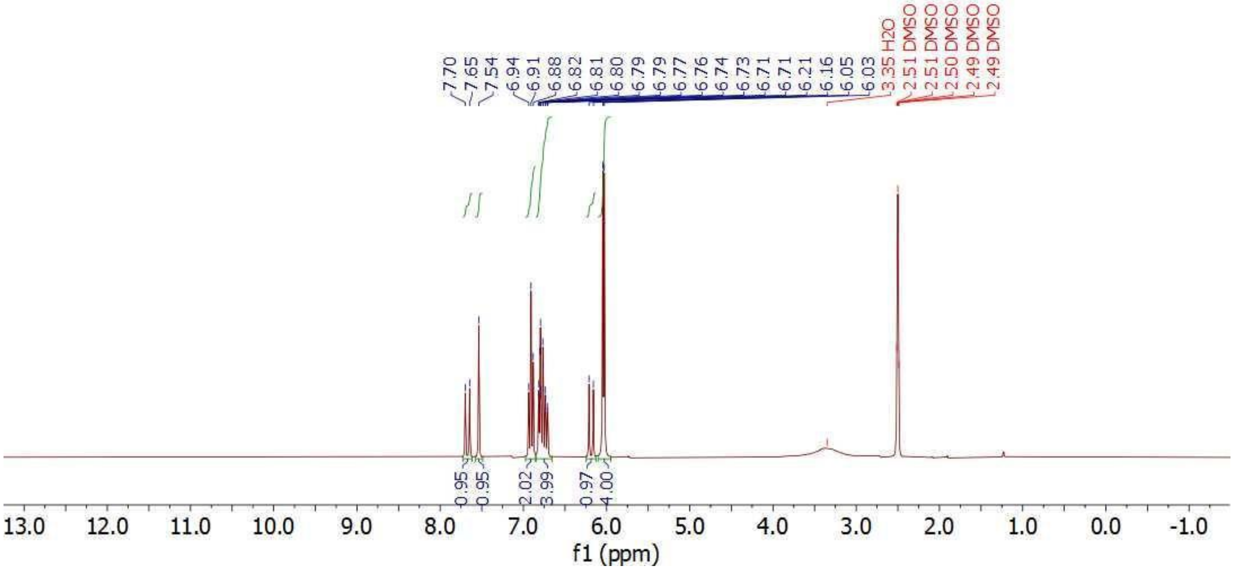

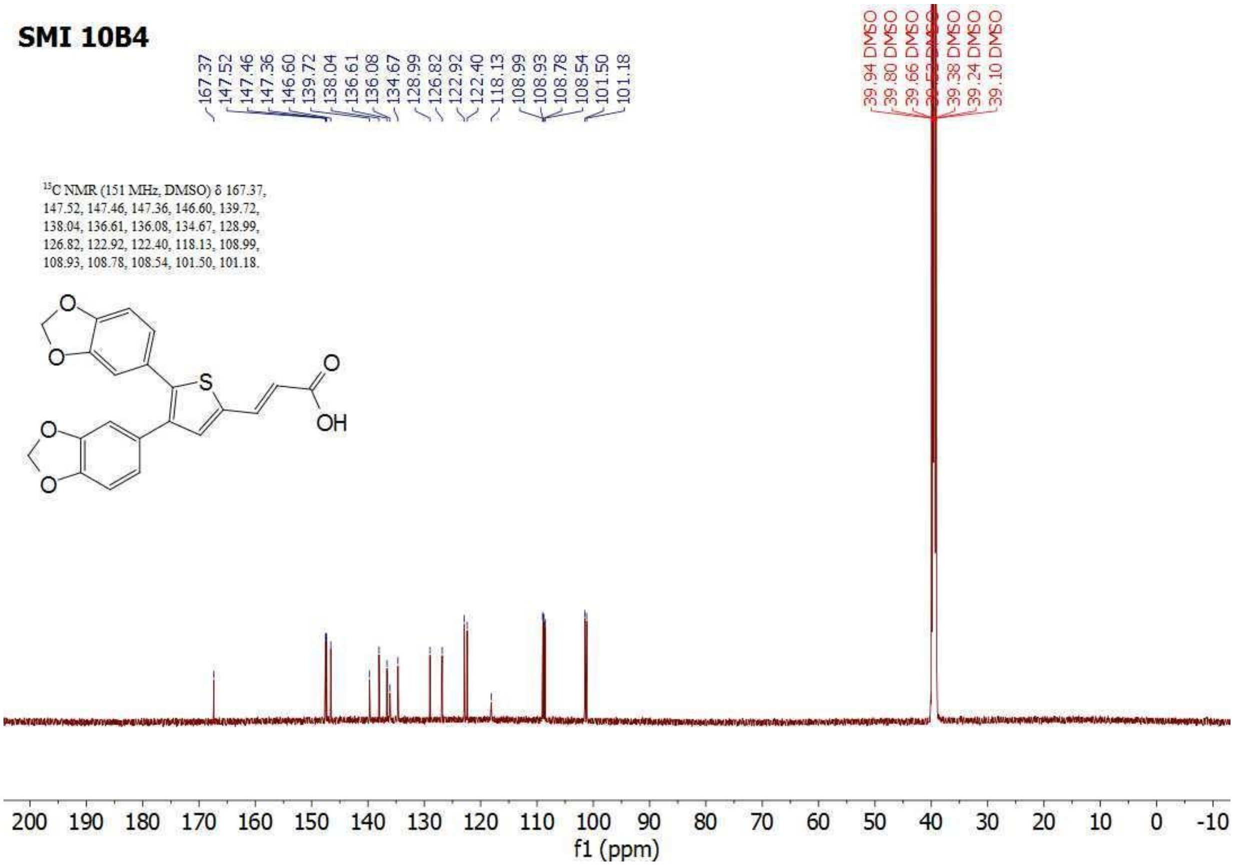

SMI-10B4, solvent DMSO, UV abs. measured at 280 nm.

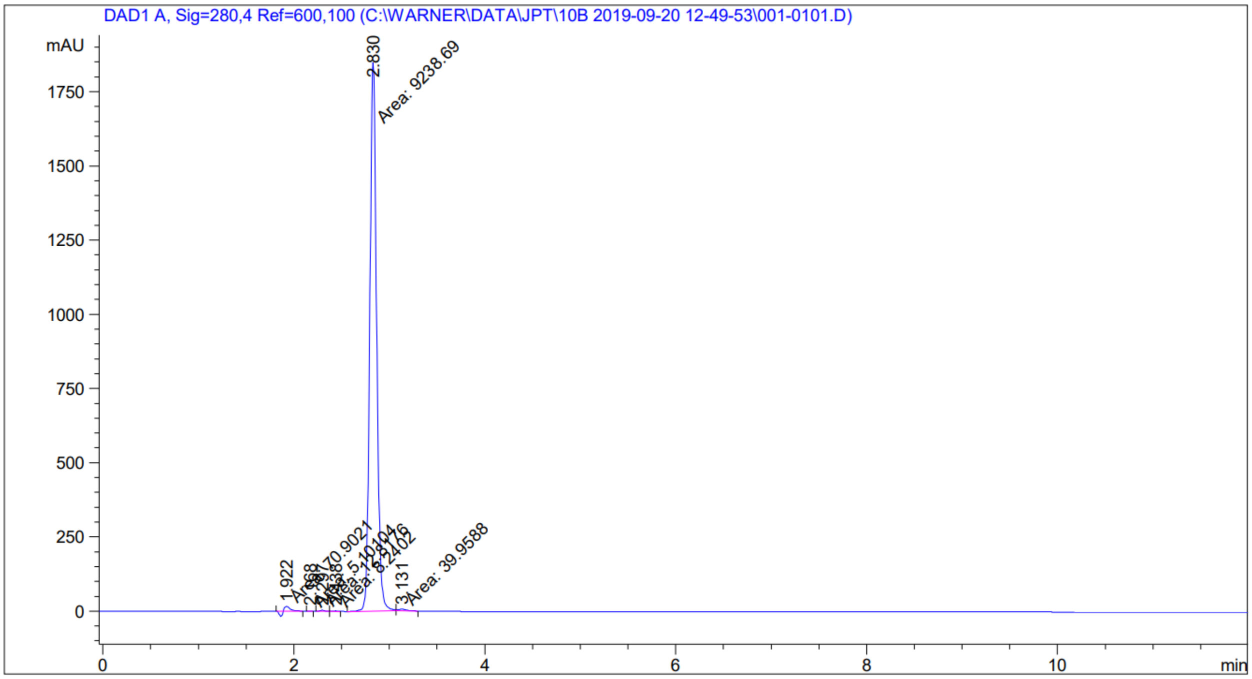

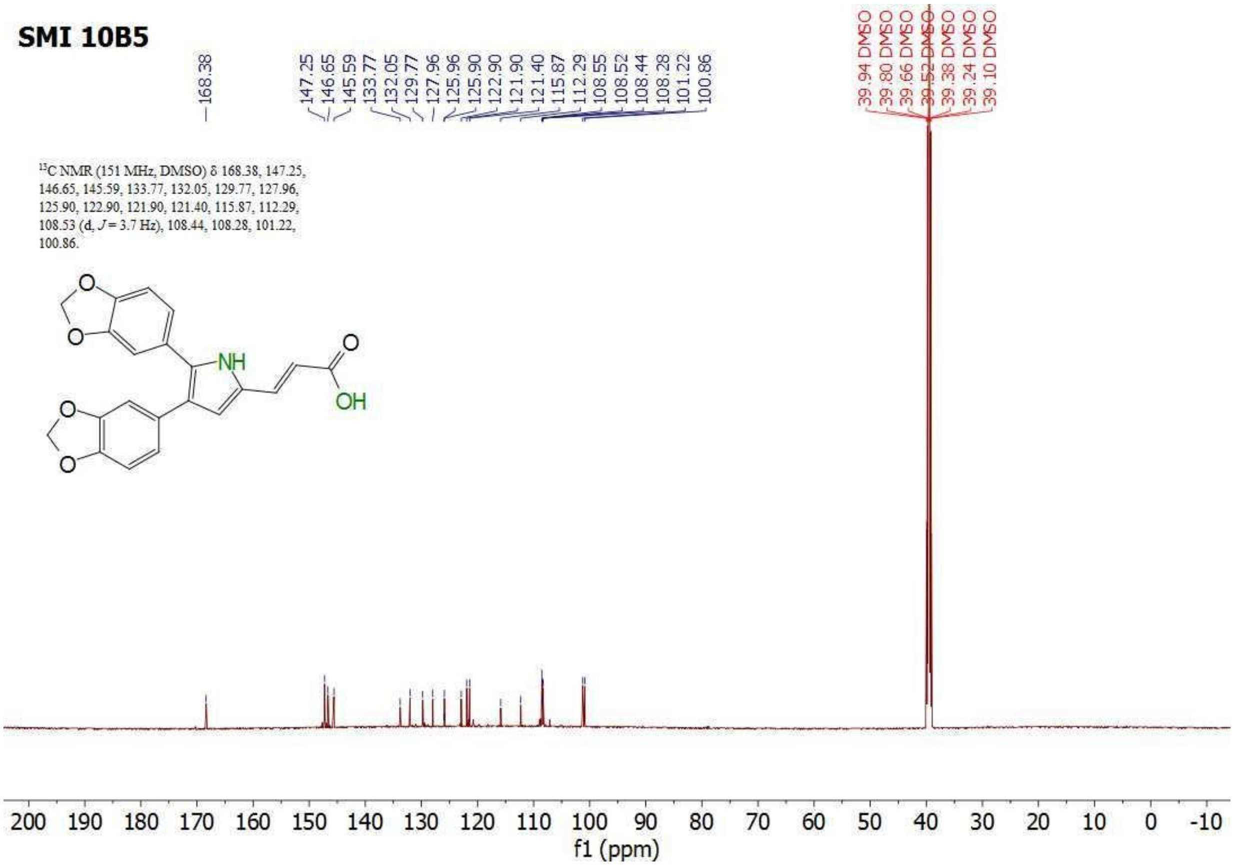
SMI 10B5
168.38
147.25
146.65
145.59
133.77
132.05
129.77
127.96
125.96
125.90
122.90
121.90
121.40
115.87
112.29
108.55
108.52
108.44
108.28
101.22
100.86
39.94 DMSO
39.80 DMSO
39.66 DMSO
39.52 DMSO
39.38 DMSO
39.24 DMSO
39.10 DMSO
^{13}C NMR (151 MHz, DMSO) δ 168.38, 147.25, 146.65, 145.59, 133.77, 132.05, 129.77, 127.96, 125.90, 122.90, 121.90, 121.40, 115.87, 112.29, 108.53 (d, J = 3.7 Hz), 108.44, 108.28, 101.22, 100.86.
NH
O
OH
200 190 180 170 160 150 140 130 120 110 100 90 80 70 60 50 40 30 20 10 0 -10
f1 (ppm)

SMI 10B5

^{1}H NMR (600 MHz, DMSO) δ 11.99 (s, 1H), 11.50 (d, J = 2.6 Hz, 1H), 7.37 (d, J = 15.8 Hz, 1H), 6.93 (d, J = 8.1 Hz, 1H), 6.89 (d, J = 1.7 Hz, 1H), 6.89 – 6.81 (m, 2H), 6.74 (d, J = 1.7 Hz, 1H), 6.70 (dd, J = 8.0, 1.7 Hz, 1H), 6.67 (d, J = 2.6 Hz, 1H), 6.29 (d, J = 15.8 Hz, 1H), 6.04 (s, 2H), 5.99 (s, 2H).

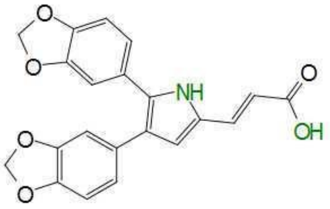

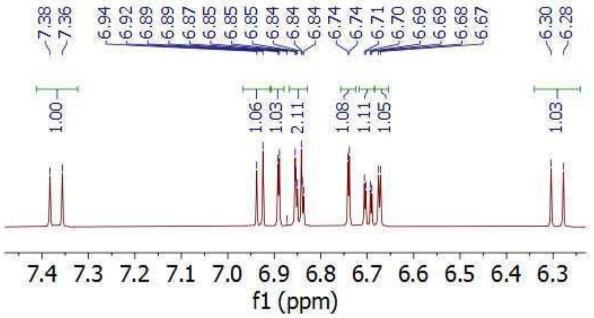

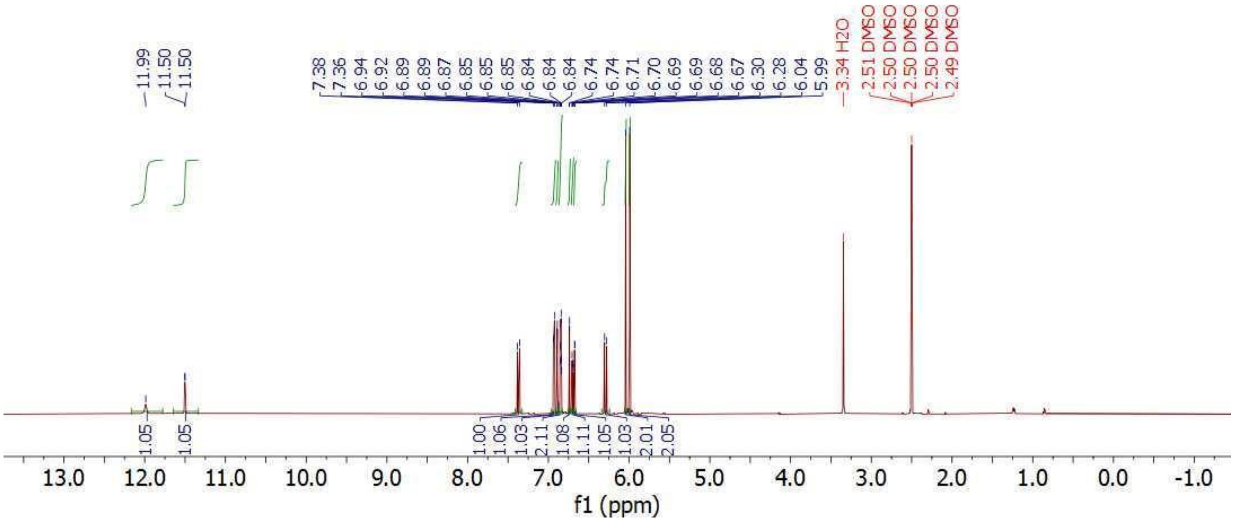

SMI-10B5, solvent DMSO, UV abs. measured at 280 nm.

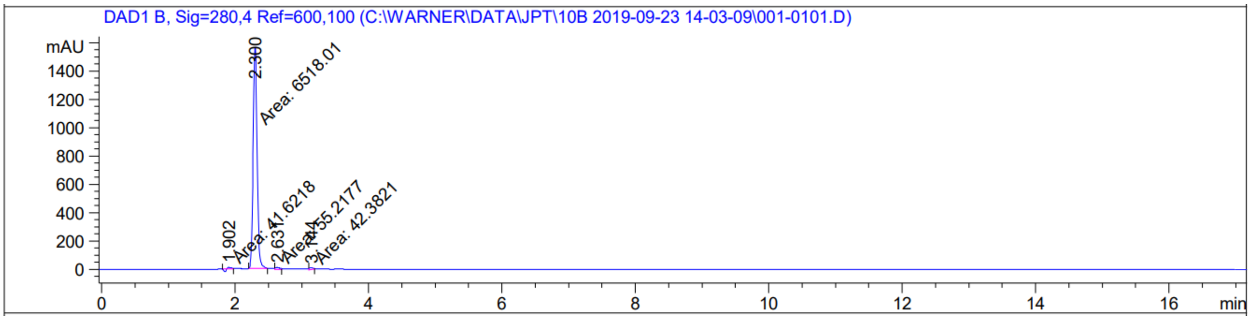

SMI 10B6

^{1}H NMR (600 MHz, DMSO) δ 12.10 (s, 1H), 7.54 (d, J = 15.6 Hz, 1H), 7.04 (s, 1H), 6.99 (d, J = 8.0 Hz, 1H), 6.86 (d, J = 1.6 Hz, 1H), 6.77 (d, J = 8.1 Hz, 1H), 6.73 (dd, J = 7.9, 1.7 Hz, 1H), 6.63 (d, J = 1.7 Hz, 1H), 6.60 (dd, J = 8.1, 1.8 Hz, 1H), 6.24 (d, J = 15.6 Hz, 1H), 6.09 (s, 2H), 5.94 (s, 2H), 3.46 (s, 3H).

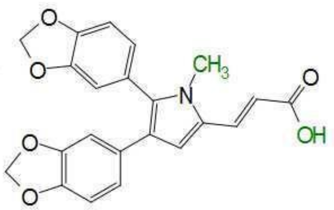

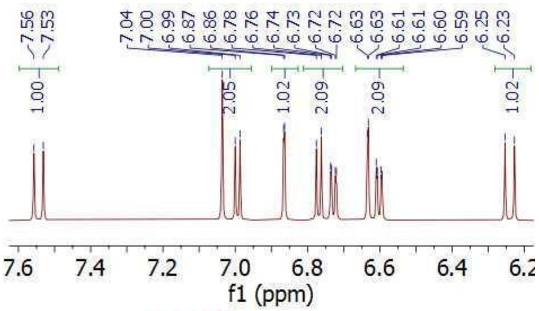

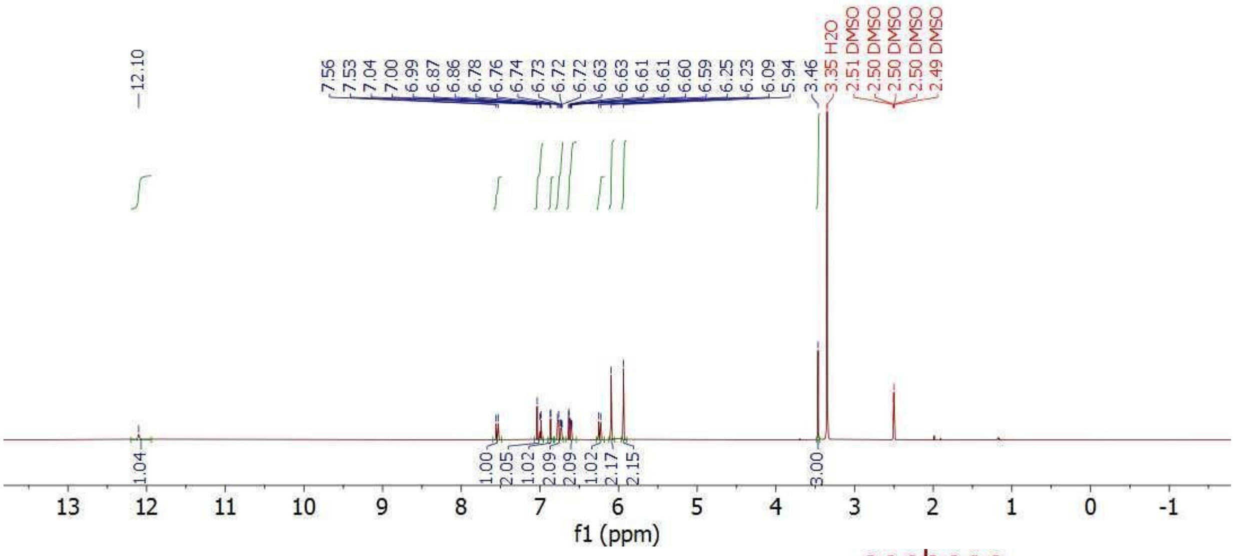

SMI 10B6

^{13}C NMR (151 MHz, DMSO) δ 168.18, 147.47, 147.34, 147.10, 145.22, 134.23, 132.31, 129.42, 128.71, 125.06, 124.82, 123.16, 120.57, 113.64, 110.93, 110.76, 108.70, 108.25, 107.74, 101.41, 100.73, 31.59.

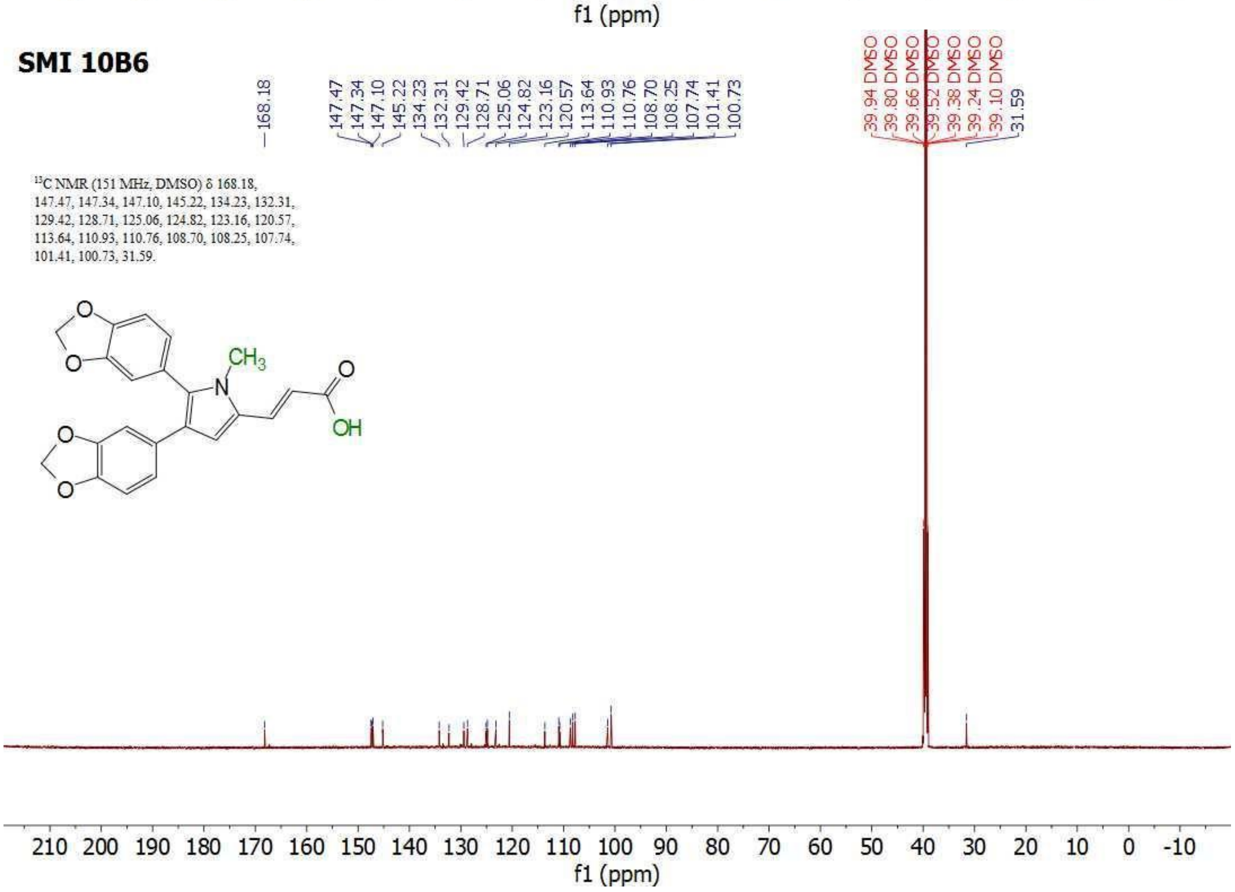

SMI-10B6, solvent DMSO, UV abs. measured at 280 nm.

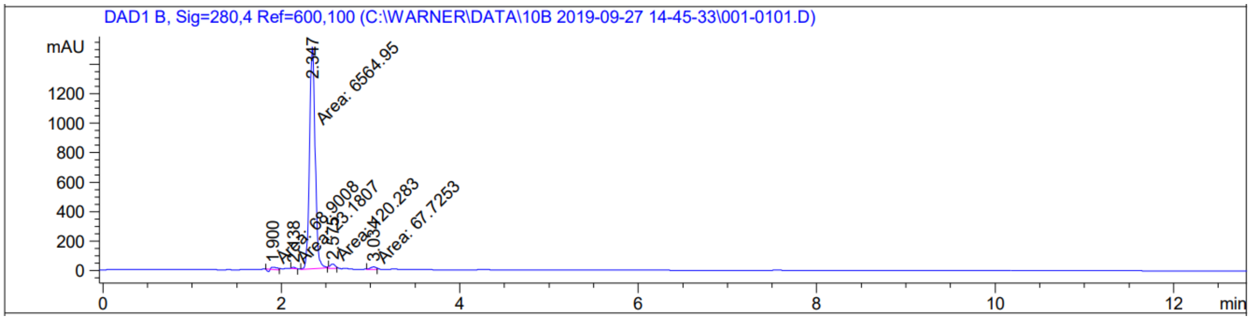

SMI 10B7

^{1}H NMR (300 MHz, DMSO) δ 12.43 (s, 1H), 7.37 (d, J = 15.7 Hz, 1H), 7.08 – 6.99 (m, 3H), 6.94 – 6.76 (m, 4H), 6.28 (d, J = 15.7 Hz, 1H), 4.31 – 4.19 (m, 8H).

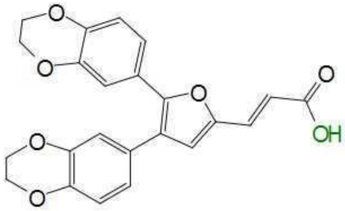

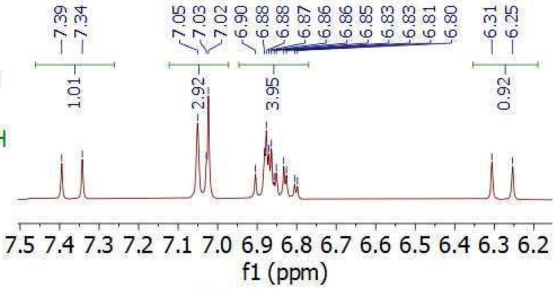

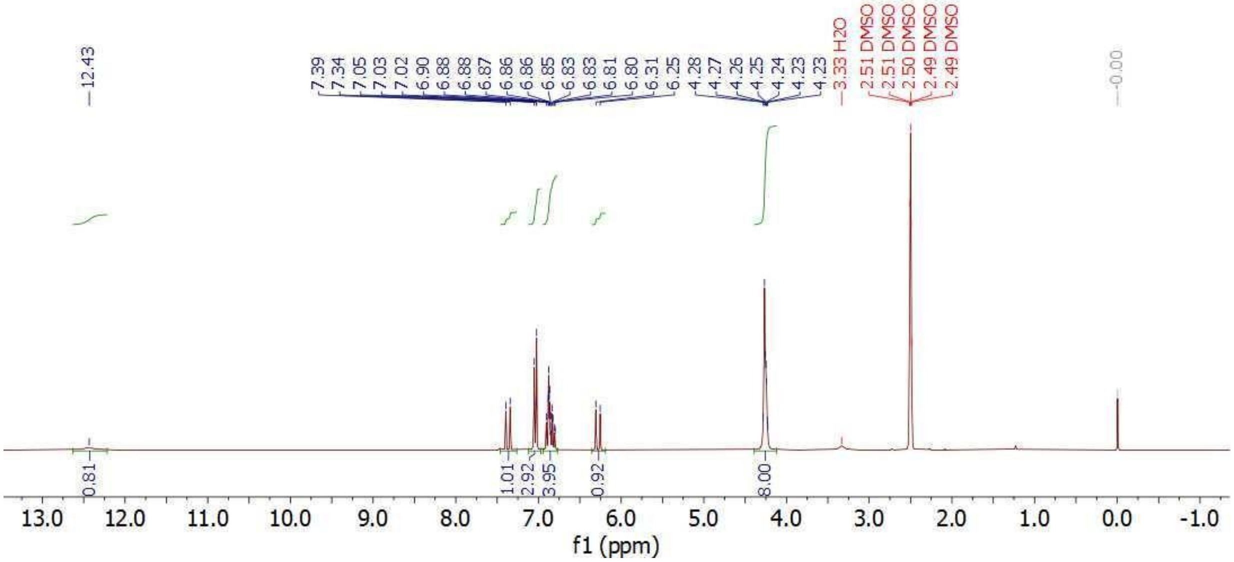

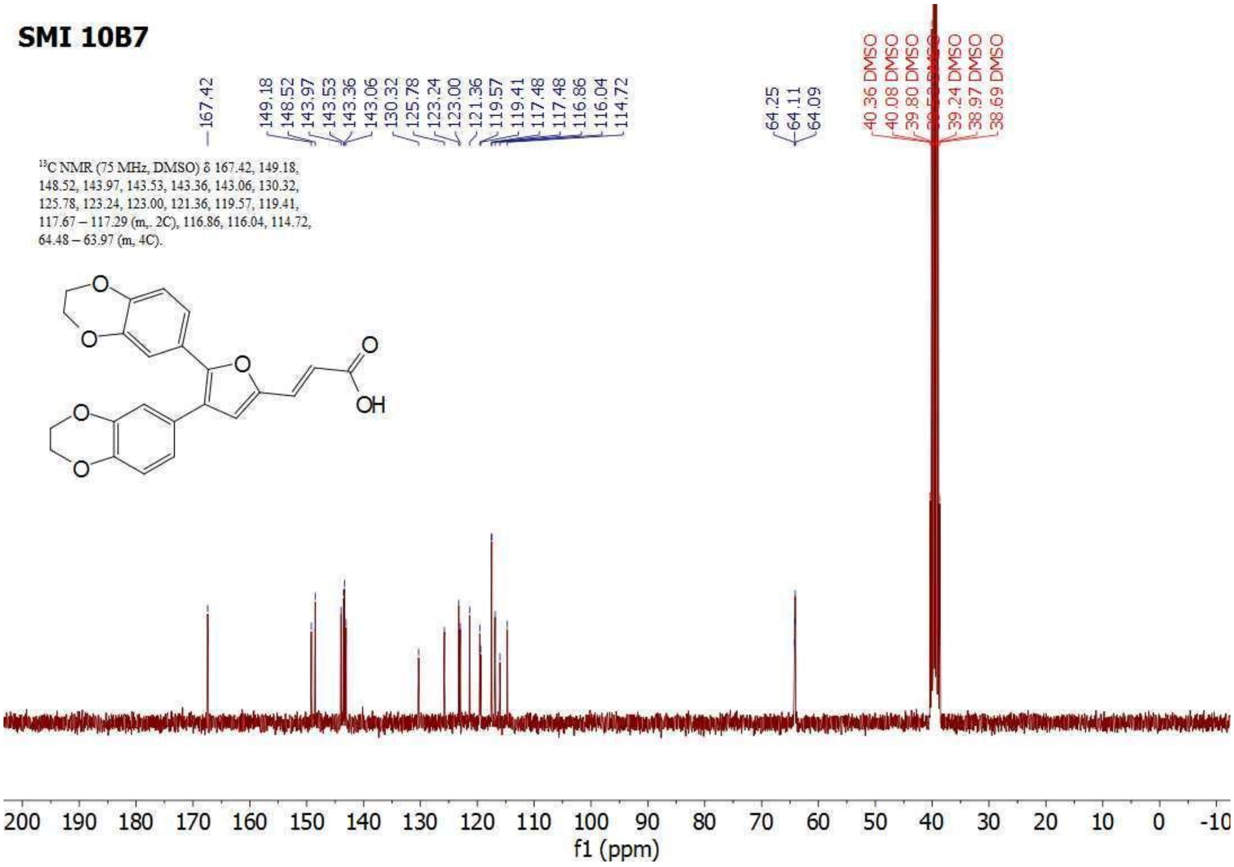

SMI-10B7, solvent DMSO, UV abs. measured at 280 nm.

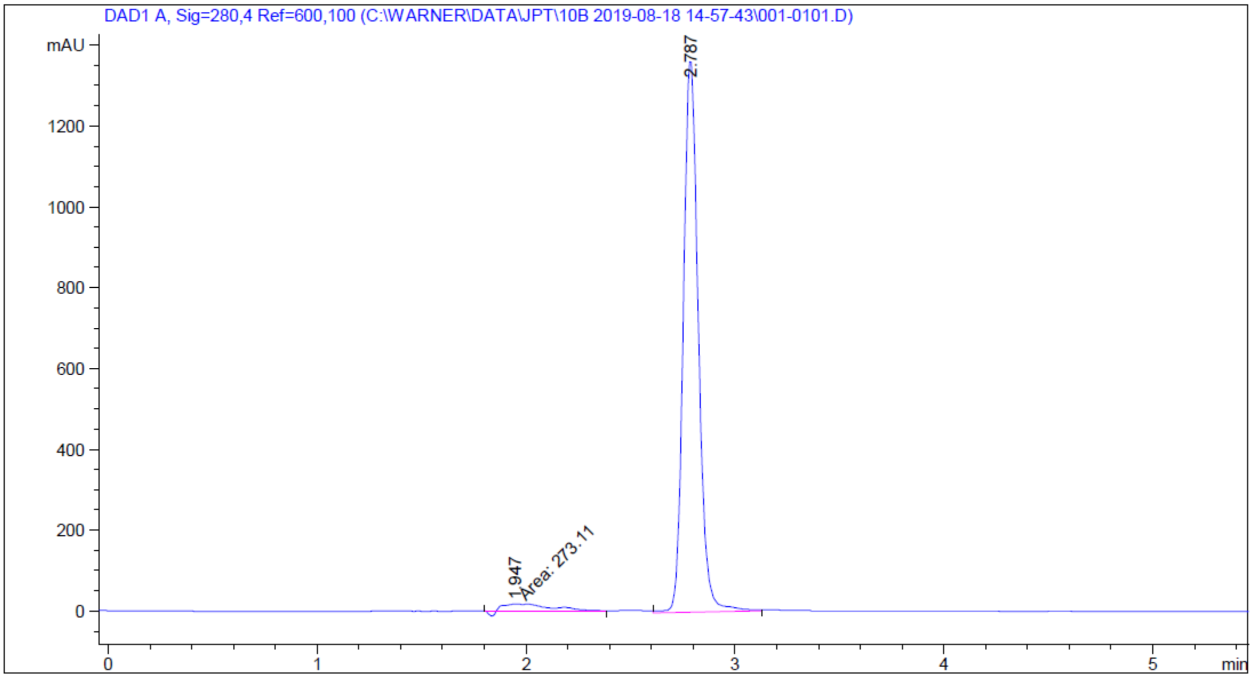

SMI 10B8

^{1}H NMR (300 MHz, DMSO) δ 12.41 (s, 1H), 7.50 – 7.31 (m, 6H), 7.13 (s, 1H), 7.09 – 6.96 (m, 2H), 6.92 (d, *J* = 8.1 Hz, 1H), 6.34 (d, *J* = 15.7 Hz, 1H), 6.05 (s, 2H).

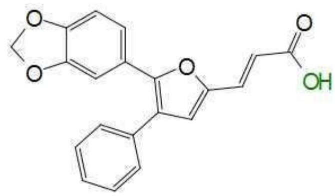

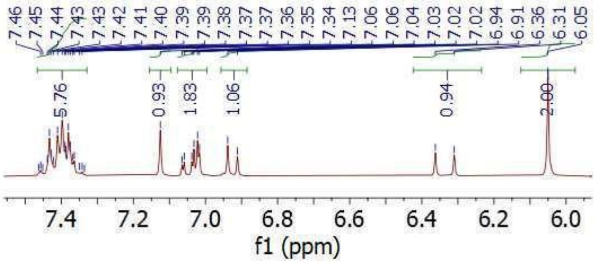

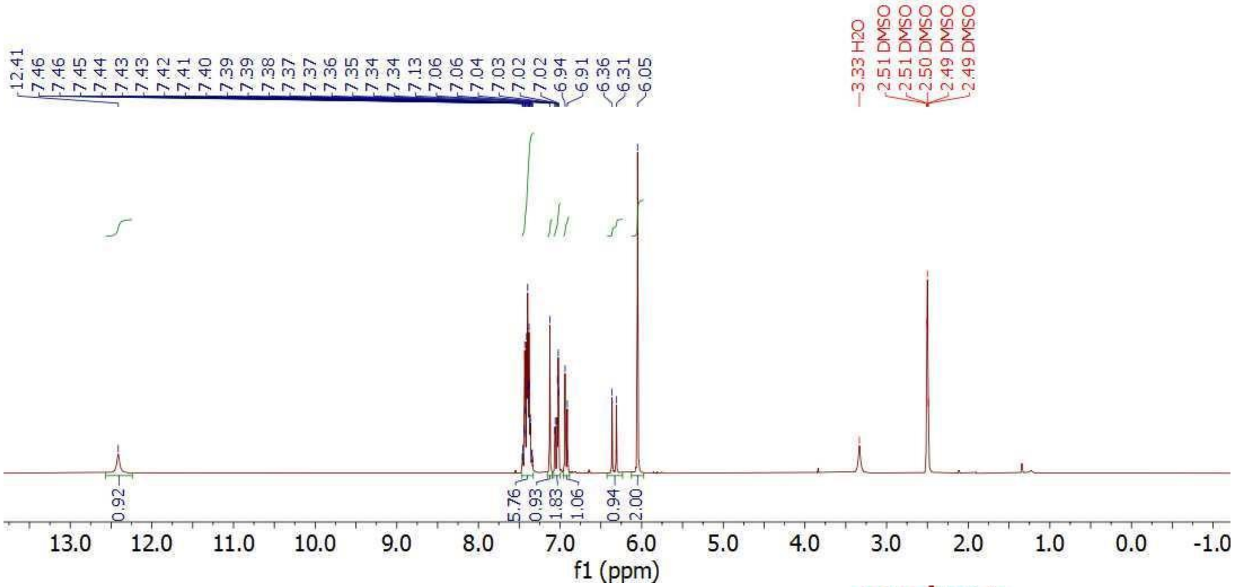

SMI 10B8

^{13}C NMR (75 MHz, DMSO) δ 167.42, 149.61, 148.76, 147.70, 147.55, 132.85, 130.24, 128.93, 128.38, 127.77, 123.73, 123.57, 120.71, 119.22, 116.35, 108.74, 106.35, 101.47.

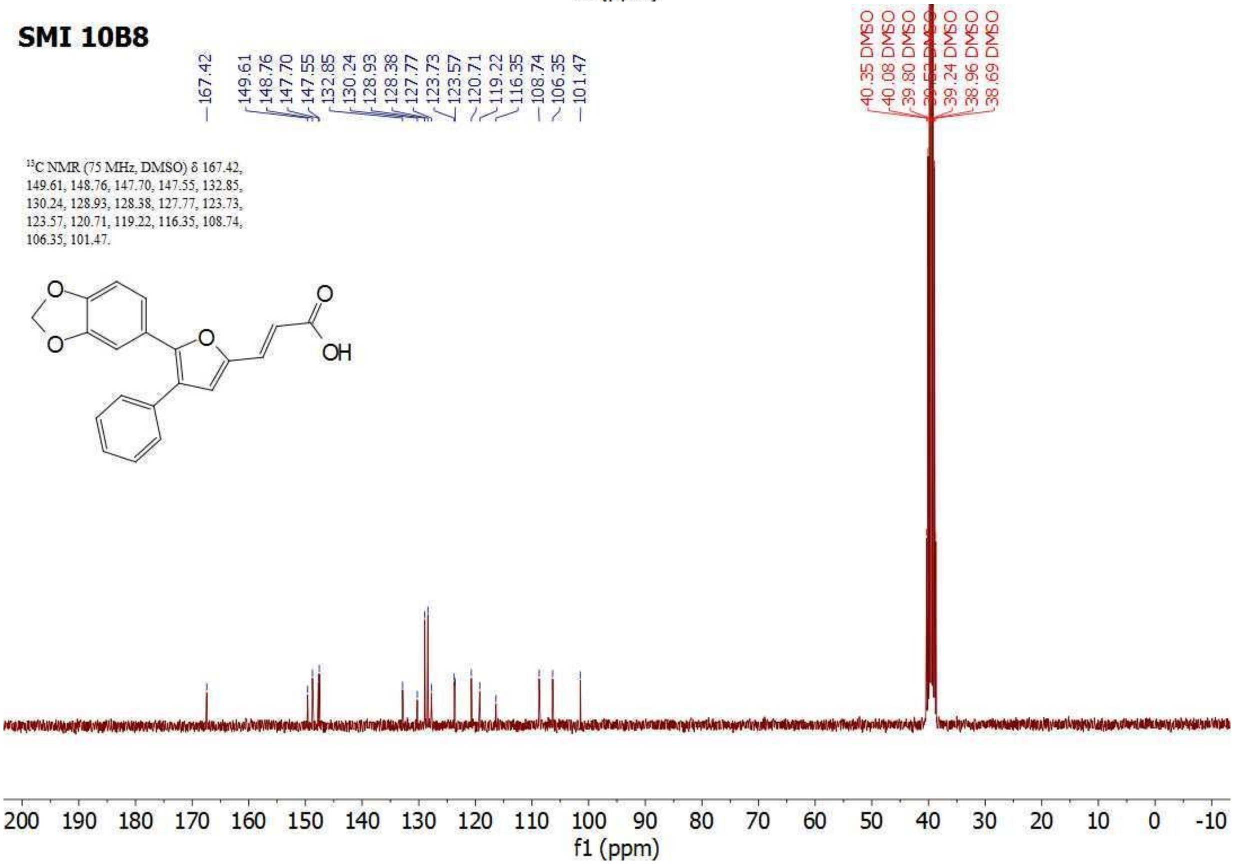

SMI-10B8, solvent DMSO, UV abs. measured at 280 nm.

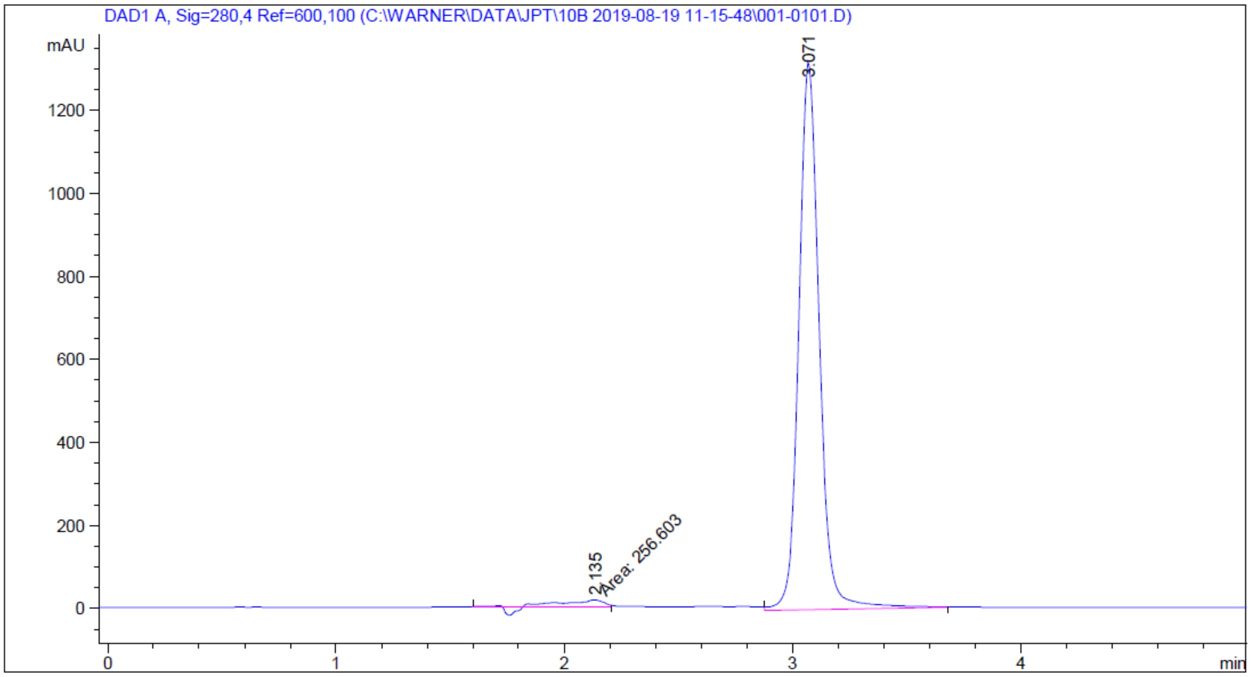

SMI 10B9

^{1}H NMR (600 MHz, DMSO) δ 12.46 (s, 1H), 7.58 – 7.52 (m, 2H), 7.44 – 7.31 (m, 4H), 7.12 (s, 1H), 6.97 (d, J = 8.0 Hz, 1H), 6.92 (d, J = 1.7 Hz, 1H), 6.86 (dd, J = 7.9, 1.7 Hz, 1H), 6.33 (d, J = 15.7 Hz, 1H), 6.06 (s, 2H).

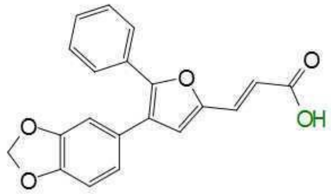

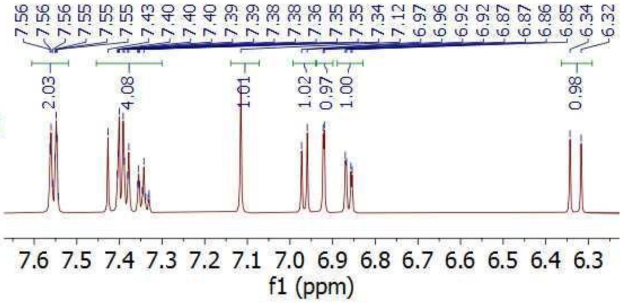

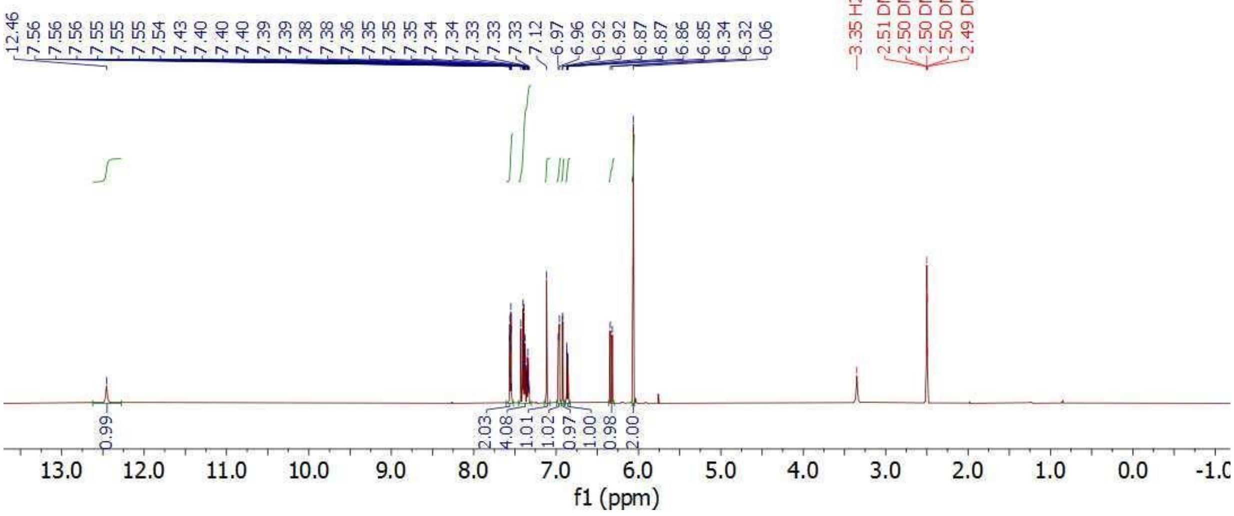

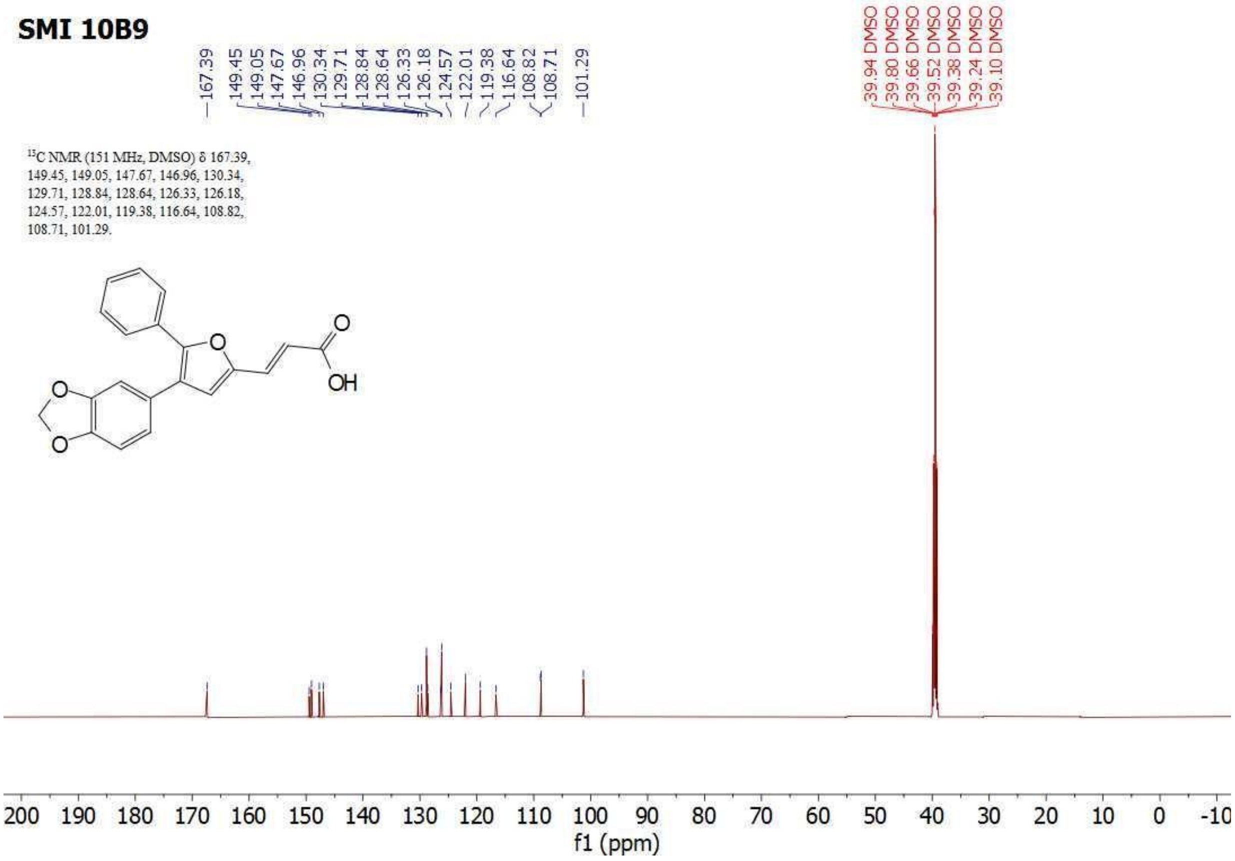

SMI-10B9, solvent DMSO, UV abs. measured at 254 nm. Injection volume: 5.0 µL

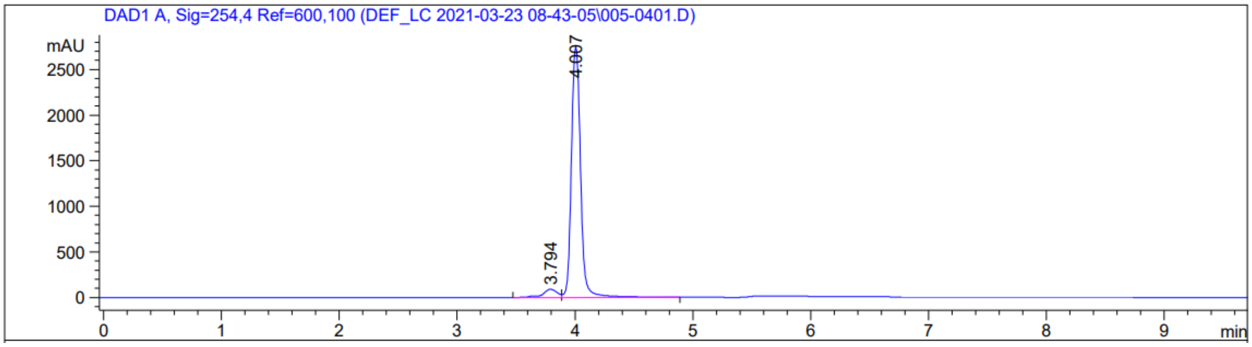

SMI 10B10

^{1}H NMR (300 MHz, DMSO) δ 12.45 (s, 1H), 7.58 – 7.48 (m, 2H), 7.50 – 7.29 (m, 9H), 7.17 (s, 1H), 6.35 (d, *J* = 15.8 Hz, 1H).

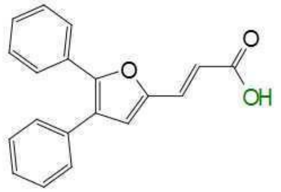

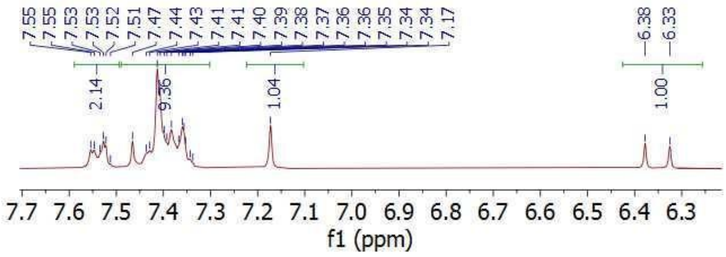

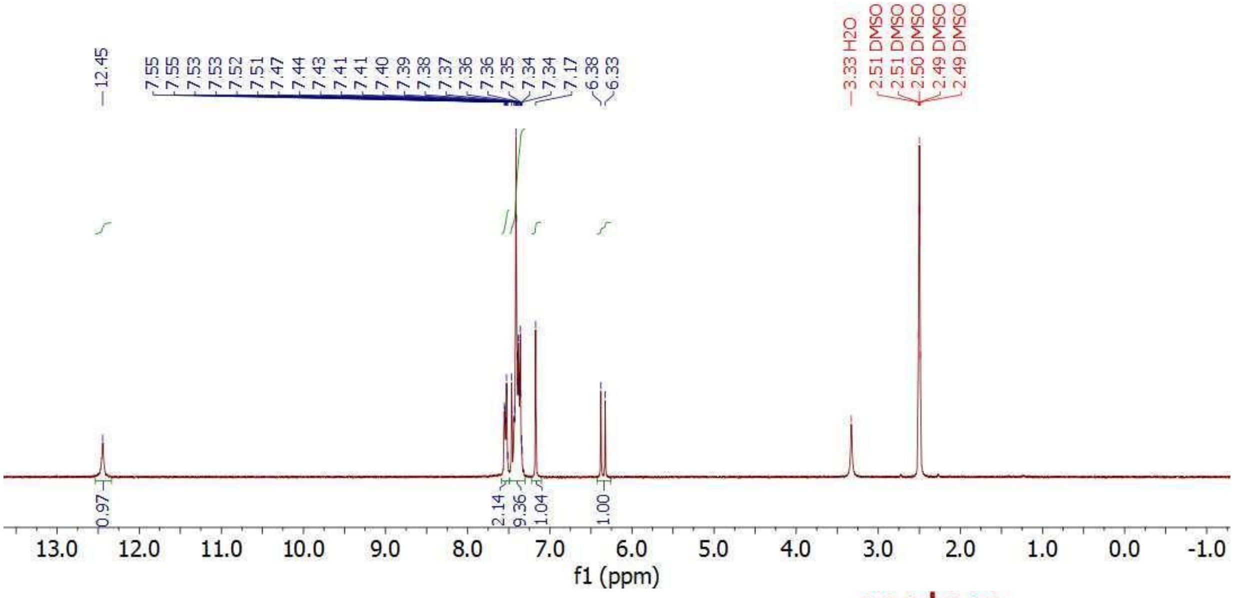

SMI 10B10

^{13}C NMR (75 MHz, DMSO) δ 167.33, 149.63, 149.27, 132.75, 130.29, 129.67, 128.93, 128.80, 128.71, 128.35, 127.86, 126.16, 124.76, 119.15, 116.73.

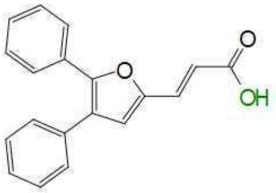

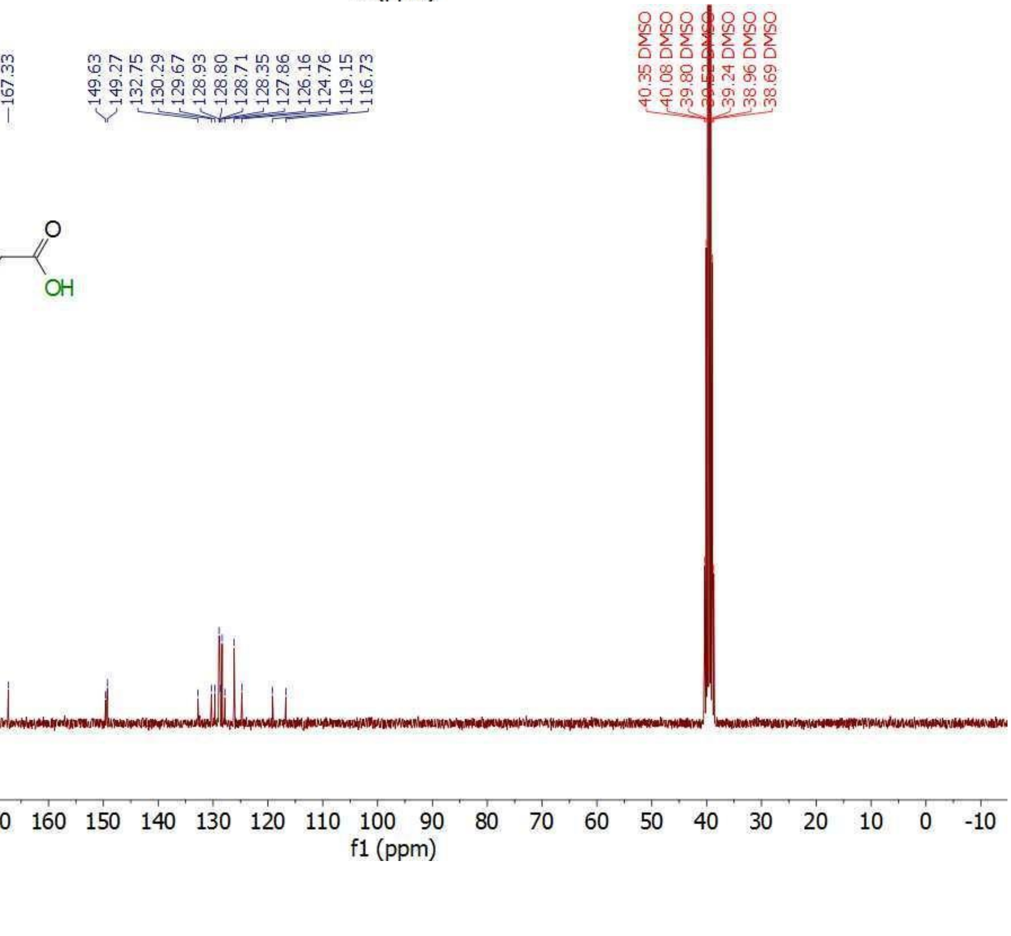

SMI-10B10, solvent DMSO, UV abs. measured at 254 nm. Injection volume: 10.0 uL

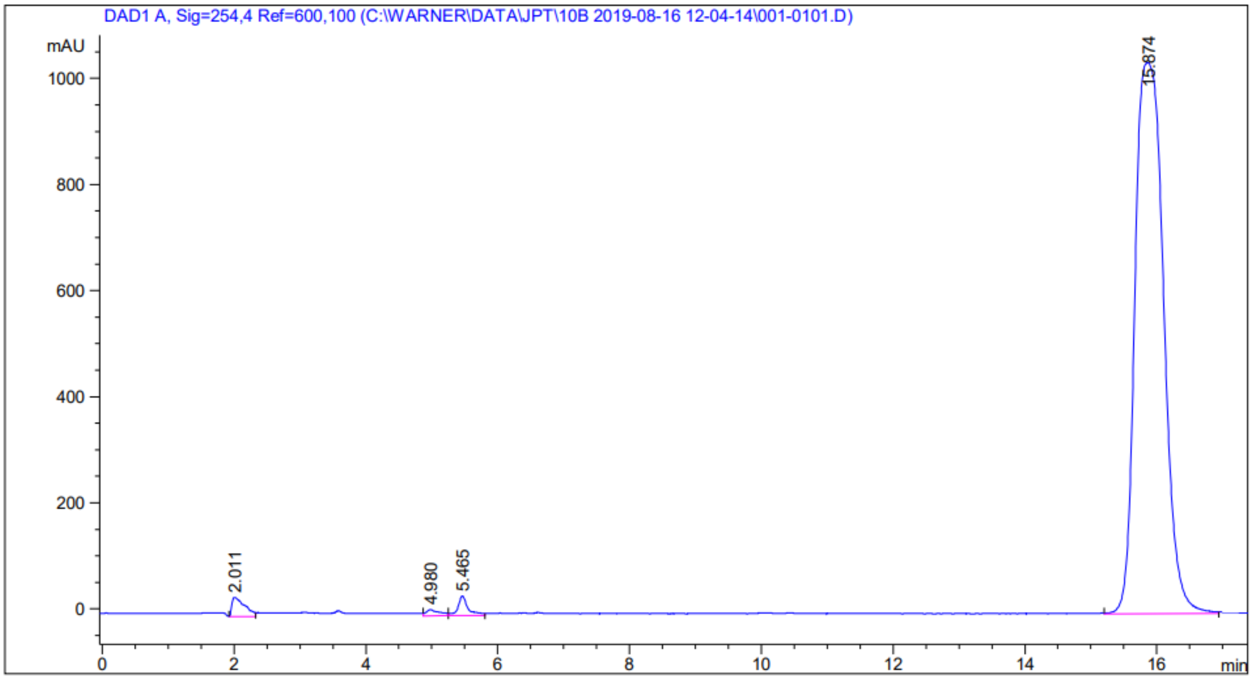

SMI 10B11

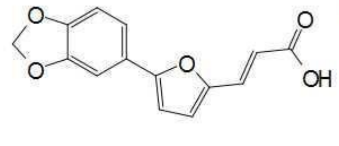

^{1}H NMR (600 MHz, DMSO) δ 12.33 (s, 1H), 7.42 (d, J = 1.7 Hz, 1H), 7.39 – 7.34 (m, 2H), 7.03 – 6.96 (m, 3H), 6.30 (d, J = 15.7 Hz, 1H), 6.08 (s, 2H).

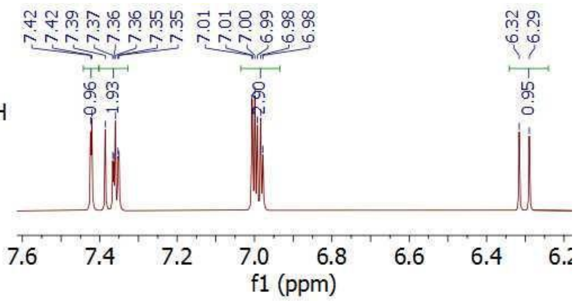

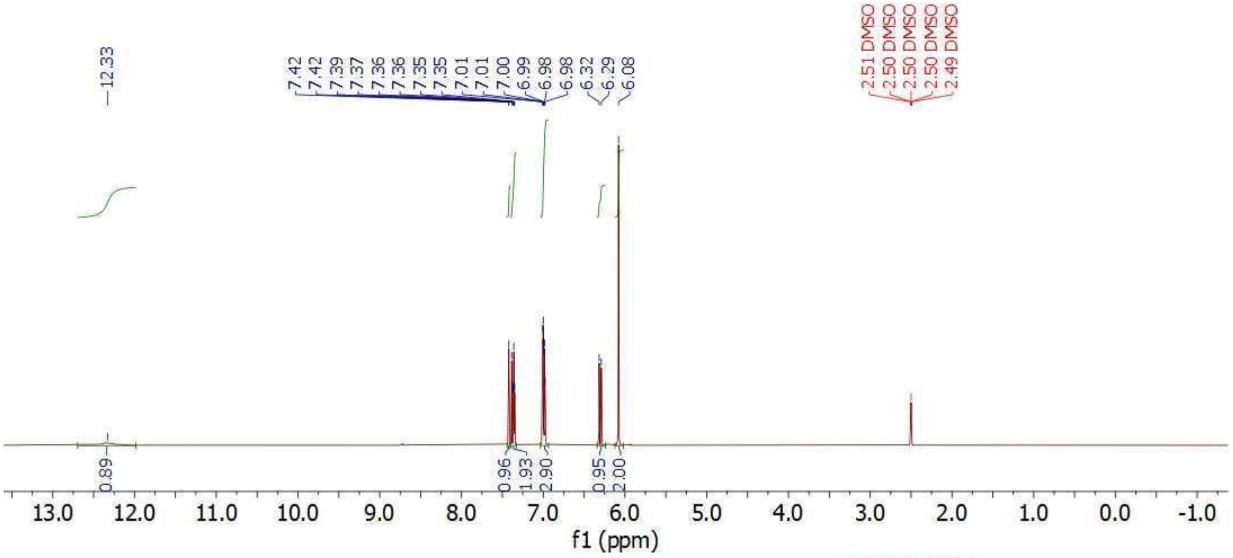

SMI 10B11

^{13}C NMR (151 MHz, DMSO) δ 167.62, 155.23, 149.43, 148.00, 147.68, 130.47, 123.72, 118.47, 118.20, 115.36, 108.90, 107.69, 104.68, 101.47.

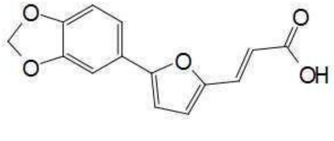

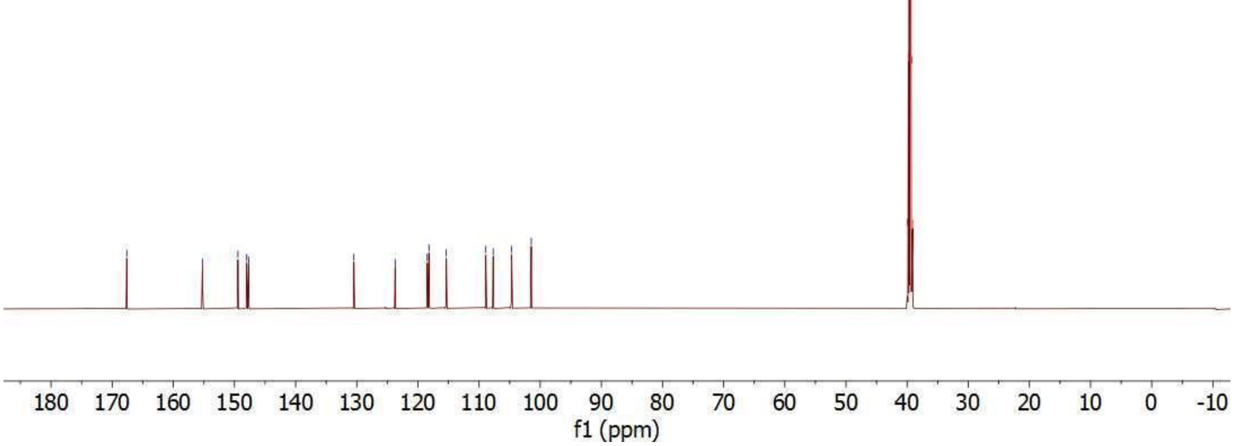

SMI-10B11, solvent DMSO, UV abs. measured at 280 nm. Injection volume: 3.0 µL

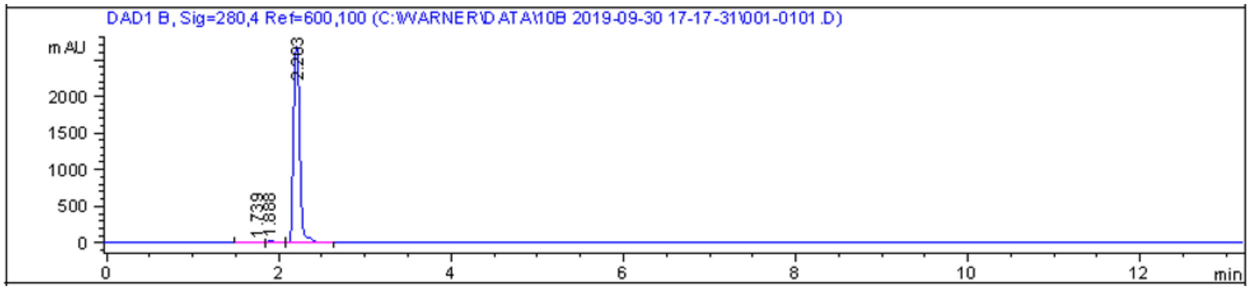

SMI 10B12

^{1}H NMR (300 MHz, DMSO) δ 12.44 (s, 1H), 8.26 (s, 1H), 7.44 – 7.31 (m, 2H), 7.24 (d, J = 1.7 Hz, 1H), 7.11 (dd, J = 8.0, 1.8 Hz, 1H), 6.95 (d, J = 8.1 Hz, 1H), 6.20 (d, J = 15.8 Hz, 1H), 6.04 (s, 2H).

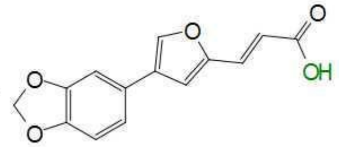

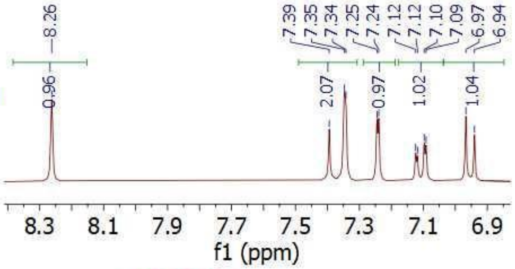

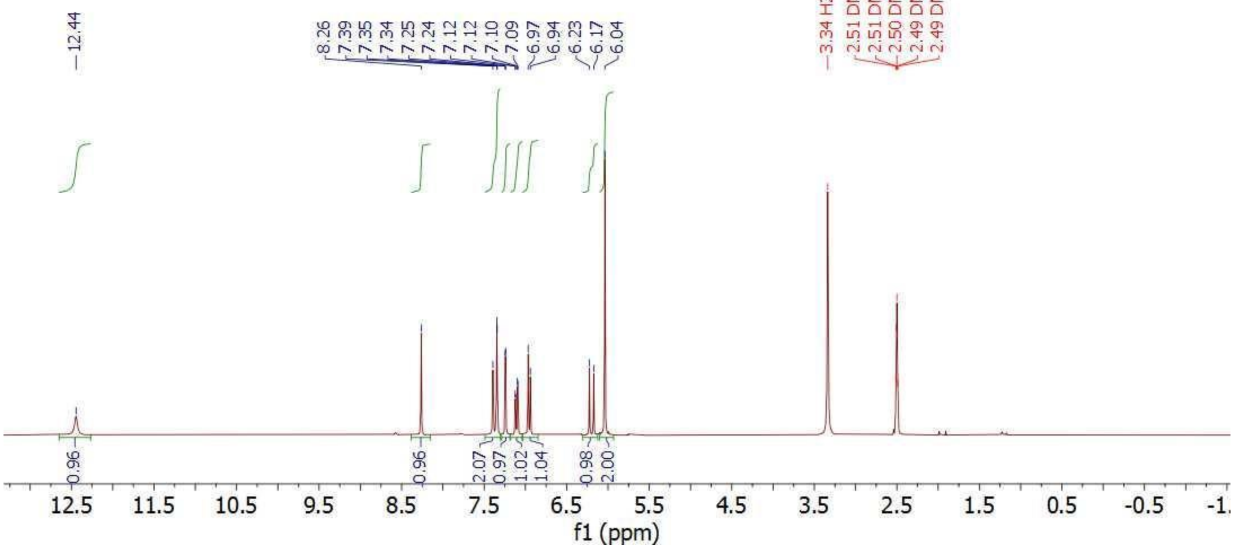

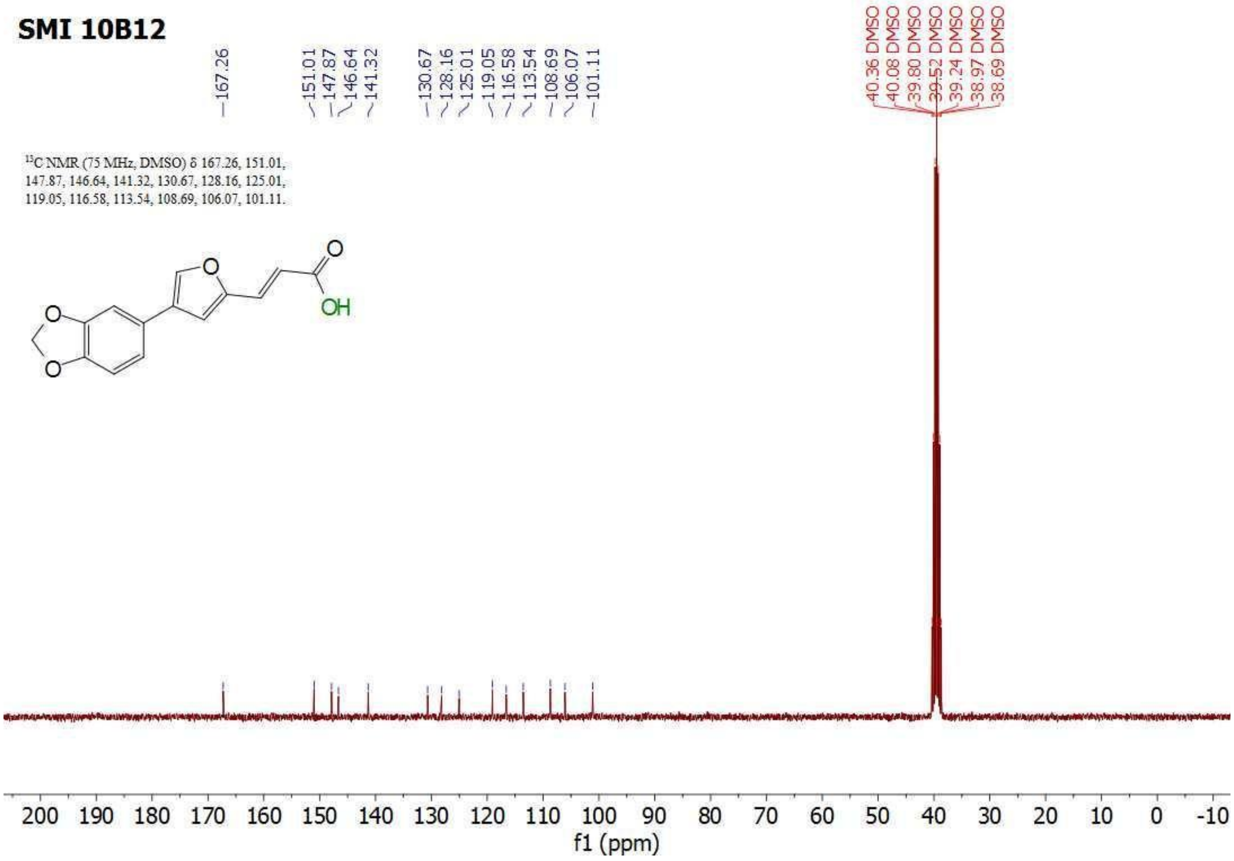

SMI-10B12, solvent DMSO, UV abs. measured at 280 nm. Injection volume: 3.0 uL

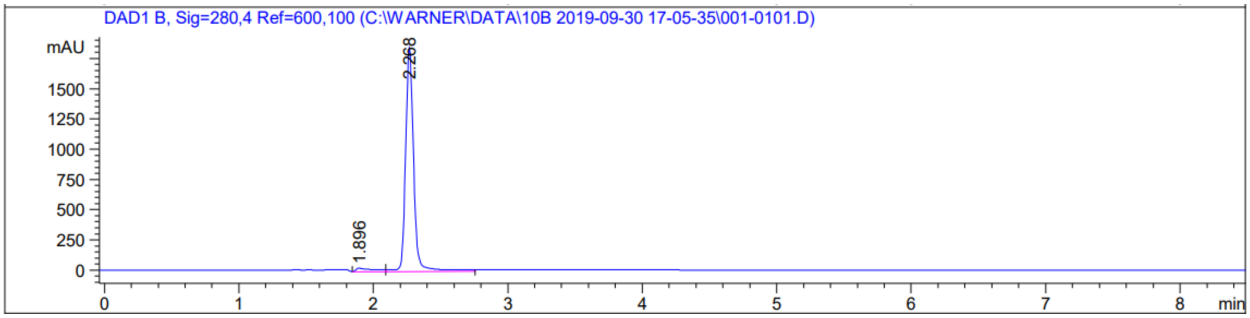

SMI 10B13

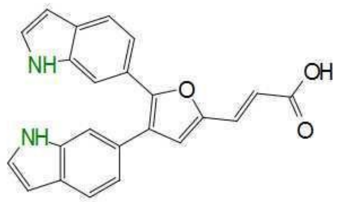

^{1}H NMR (300 MHz, DMSO) δ 11.18 (d, J = 11.4 Hz, 2H), 7.64 (s, 1H), 7.56 (d, J = 8.1 Hz, 1H), 7.54 – 7.42 (m, 2H), 7.40 – 7.29 (m, 3H), 7.25 (d, J = 7.6 Hz, 1H), 7.11 – 7.01 (m, 2H), 6.48 – 6.37 (m, 2H), 6.33 (d, J = 15.7 Hz, 1H).

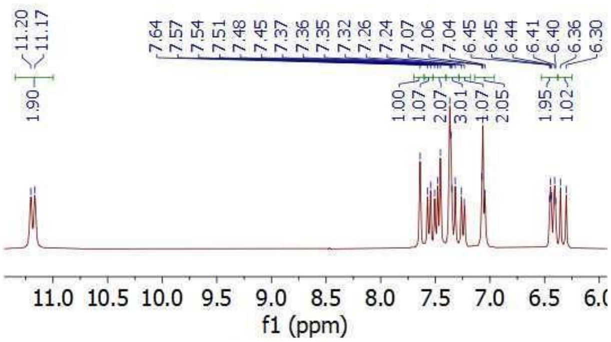

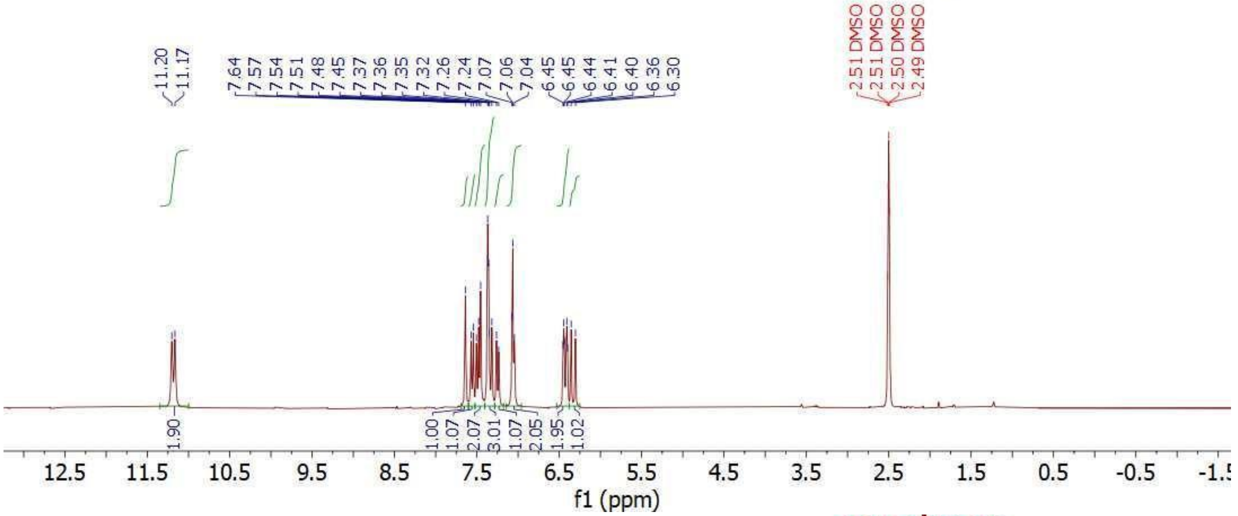

SMI 10B13

^{13}C NMR (75 MHz, DMSO) δ 168.52, 150.43, 149.00, 136.15, 135.69, 128.57 (m, 2C), 127.79, 127.05, 126.05, 125.93, 124.36, 123.07, 120.33, 120.18, 119.74, 118.73 (m, 2C), 117.66, 111.07, 109.45, 101.31, 101.11.

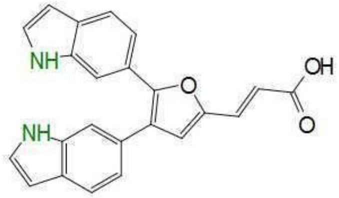

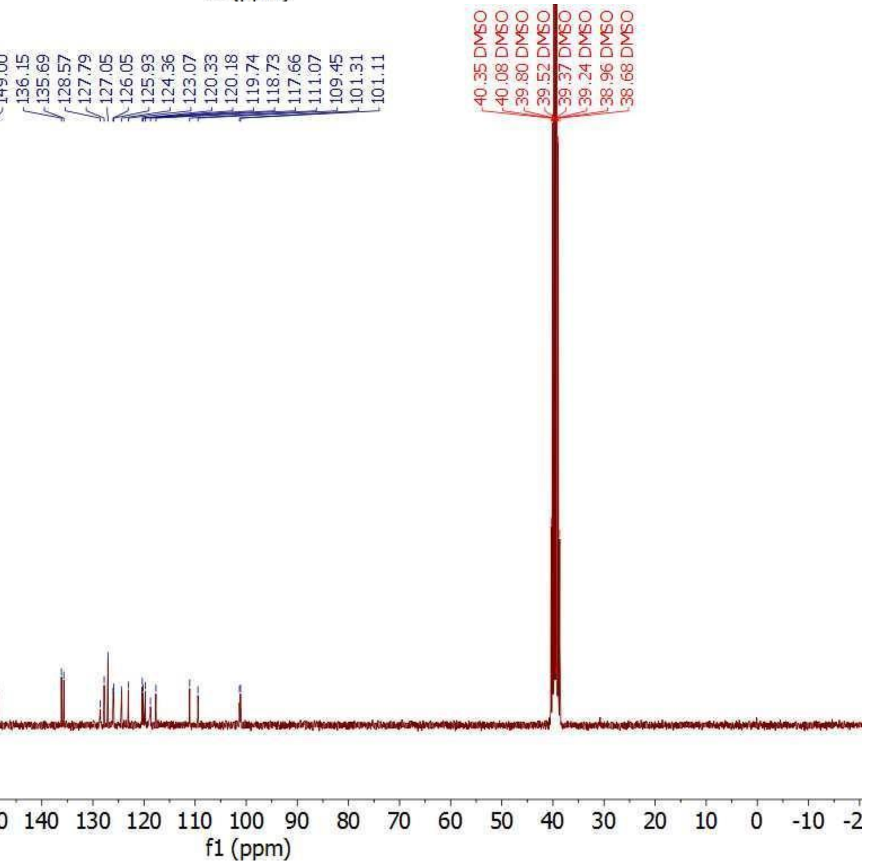

SMI-10B13, solvent DMSO, UV abs. measured at 280 nm.

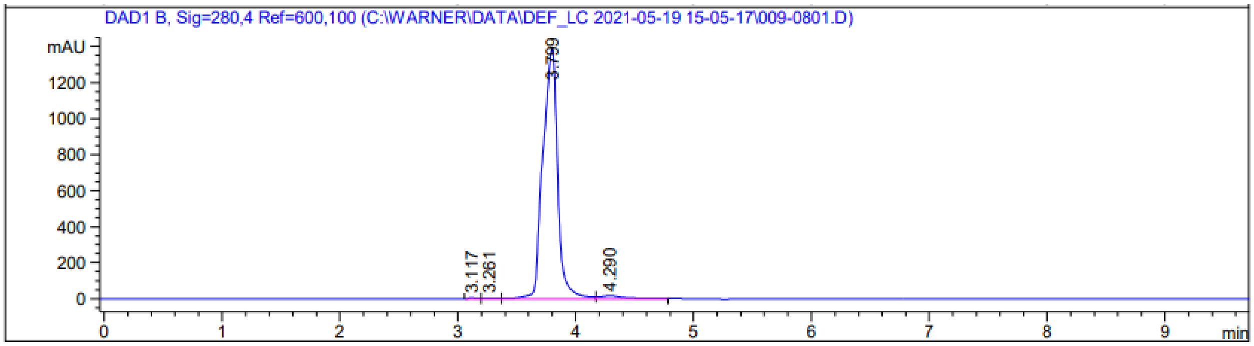

SMI 10B14

^{1}H NMR (300 MHz, $CDCl_3$ with 0.05% v/v TMS) δ 7.00 (dd, J = 8.1, 1.7 Hz, 1H), 6.95 (d, J = 1.7 Hz, 1H), 6.89 – 6.75 (m, 3H), 6.74 (d, J = 8.1 Hz, 1H), 6.17 – 6.10 (m, 1H), 5.97 (s, 2H), 5.94 (s, 2H), 3.03 (t, J = 7.5 Hz, 2H), 2.79 (t, J = 7.5 Hz, 2H).

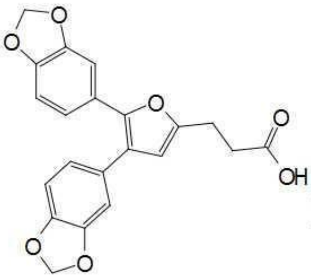

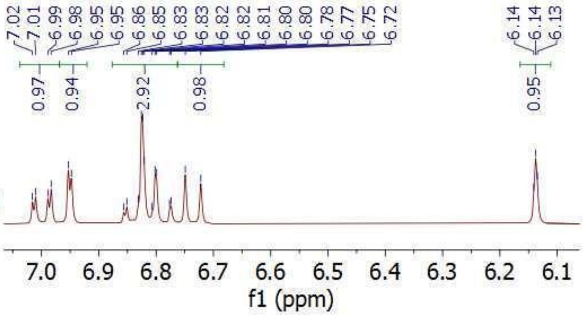

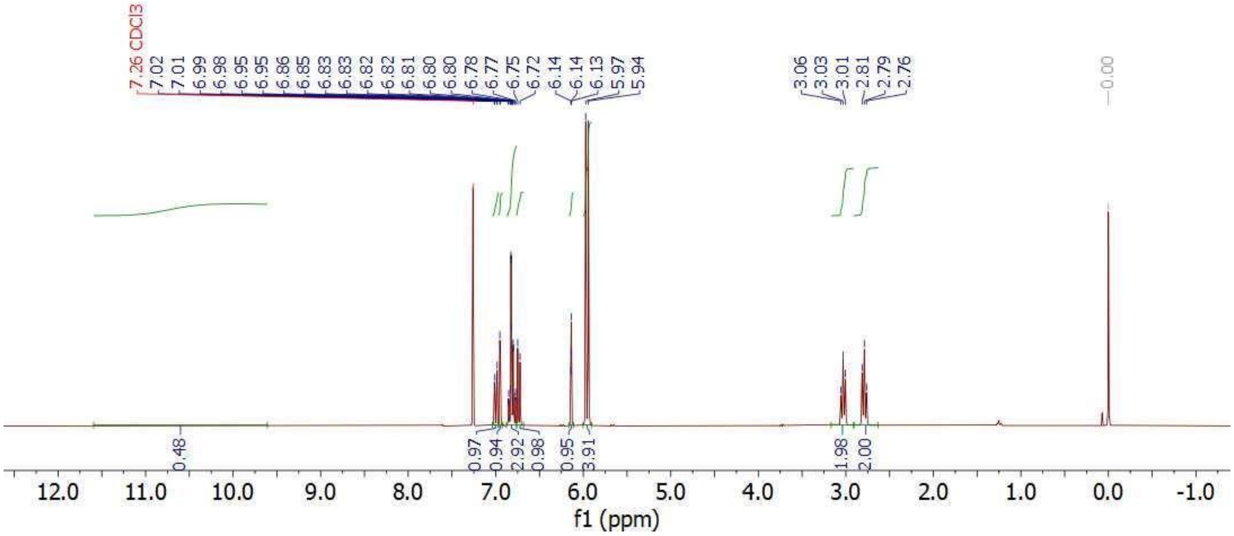

SMI 10B14

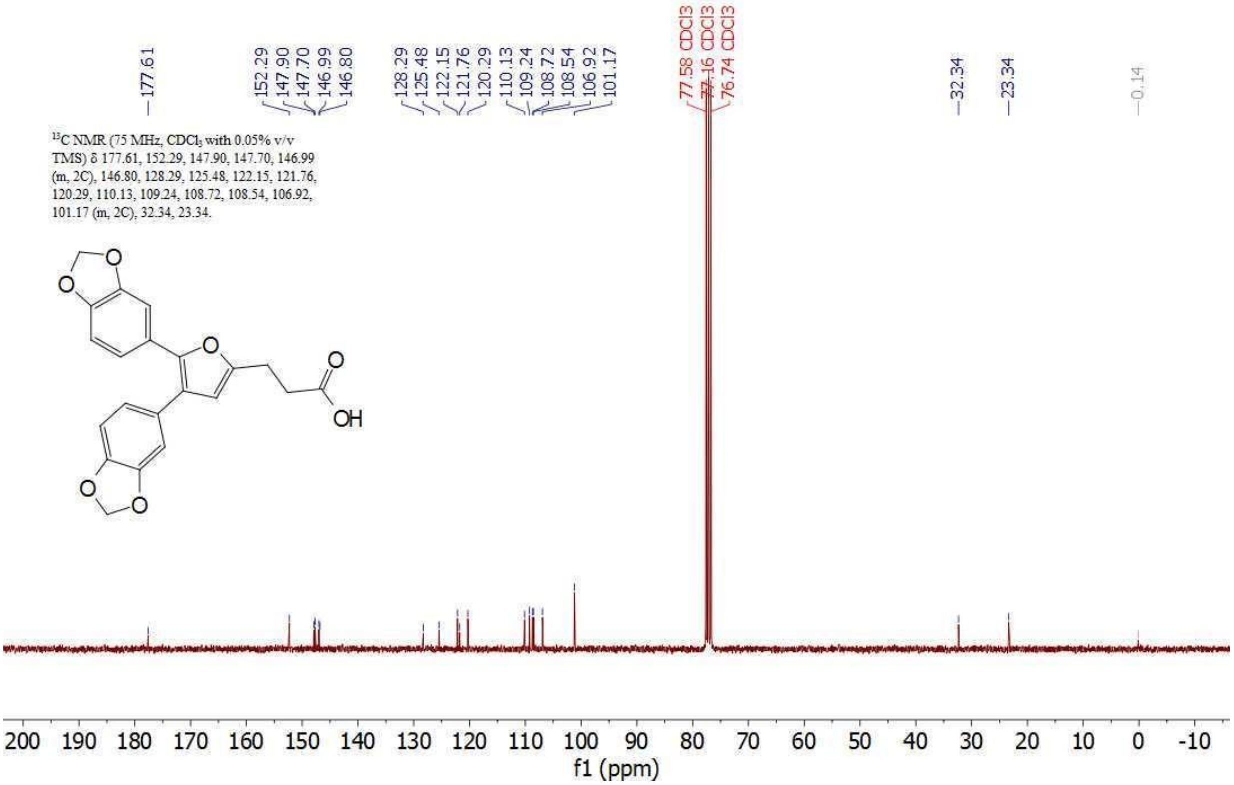

SMI-10B14, solvent DMSO, UV abs. measured at 280 nm.

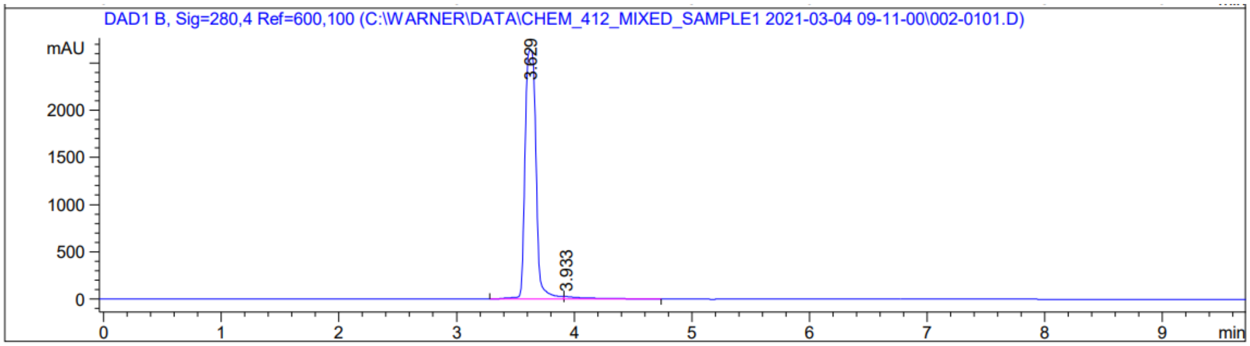

SMI 10B15

^{1}H NMR (600 MHz, $CDCl_3$ with 0.05% v/v TMS) δ 10.68 (s, 1H), 7.19 – 7.13 (m, 1H), 7.13 – 7.03 (m, 2H), 6.94 (dd, J = 8.1, 1.7 Hz, 1H), 6.90 (d, J = 1.7 Hz, 1H), 6.75 (d, J = 8.1 Hz, 1H), 6.16 (t, J = 0.9 Hz, 1H), 5.96 (s, 2H), 3.04 (t, J = 7.4 Hz 1H), 2.79 (t, J = 7.5 Hz, 2H).

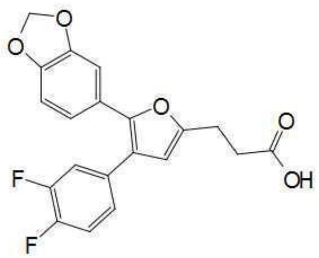

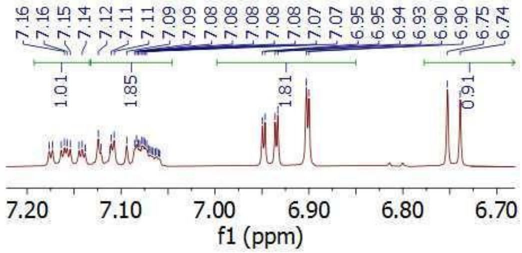

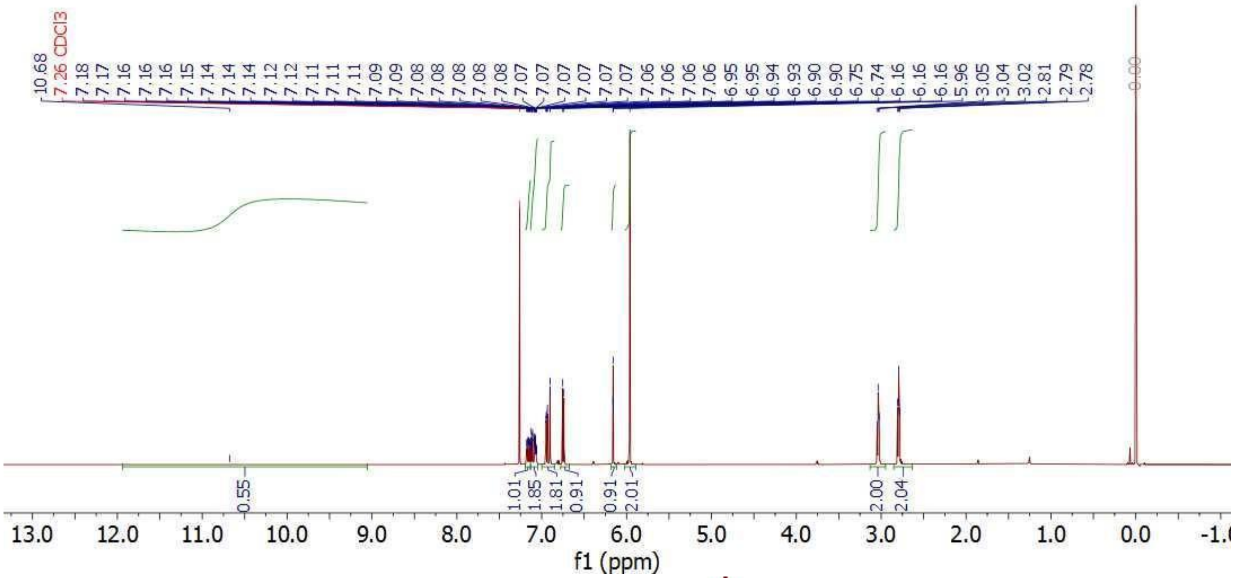

SMI 10B15

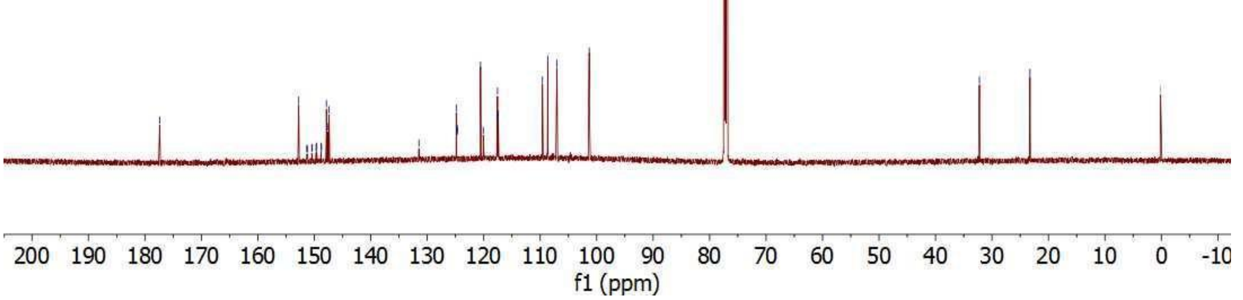

^{13}C NMR (151 MHz, $CDCl_3$ with 0.05% v/v TMS) δ 177.38, 152.77, 151.28 (d, J = 12.9 Hz, 0.5C), 150.41 (d, J = 12.6 Hz, 0.5C), 149.64 (d, J = 12.6 Hz, 0.5C), 148.77 (d, J = 12.5 Hz, 0.5C), 147.86, 147.67, 147.39, 131.55 – 131.40 (m, 1C), 124.86, 124.74 (dd, J = 6.2, 3.4 Hz, 1C), 120.57, 120.08 (m, 2C), 117.56 (t, J = 16.8 Hz, 1C), 109.61, 108.66, 107.03, 101.32, 32.22, 23.27.

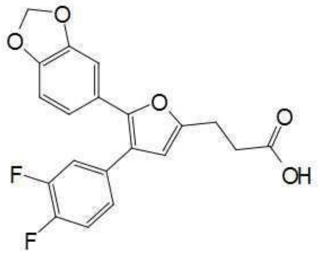

SMI-10B15, solvent DMSO, UV abs. measured at 280 nm. Injection volume: 3.0 µL

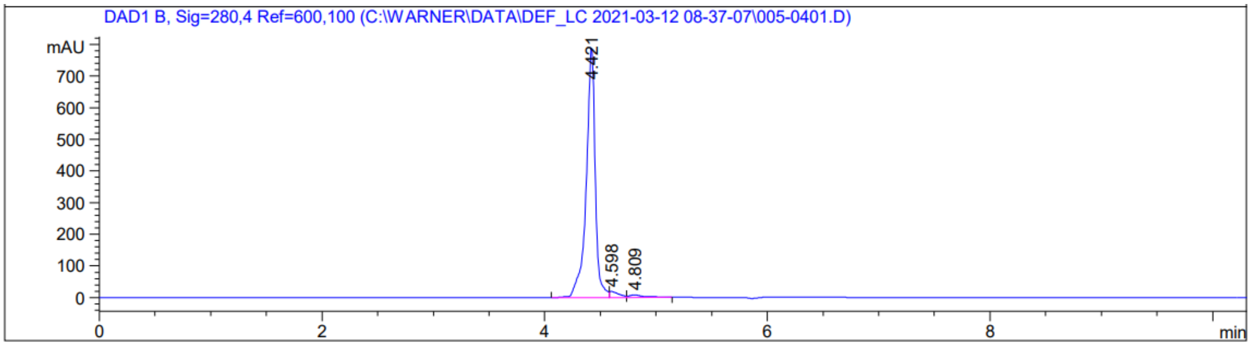

SMI 10B16

^{1}H NMR (600 MHz, DMSO) δ 9.11 (s, 1H), 8.78 (s, 2H), 6.96 – 6.92 (m, 2H), 6.90 (dd, *J* = 8.1, 1.7 Hz, 1H), 6.57 (t, *J* = 1.0 Hz, 1H), 6.06 (s, 2H), 2.92 (t, *J* = 7.9 Hz, 1H), 2.64 (t, *J* = 7.4 Hz, 2H).

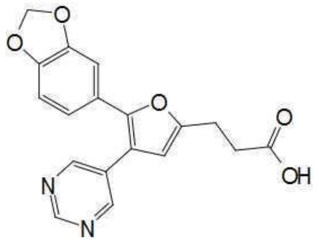

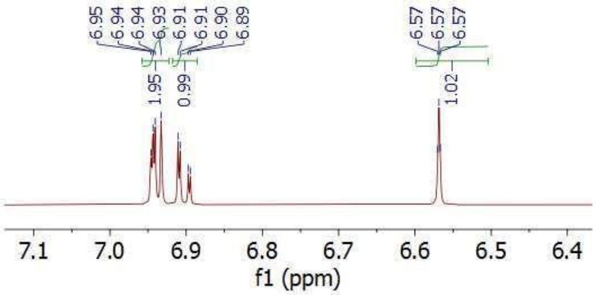

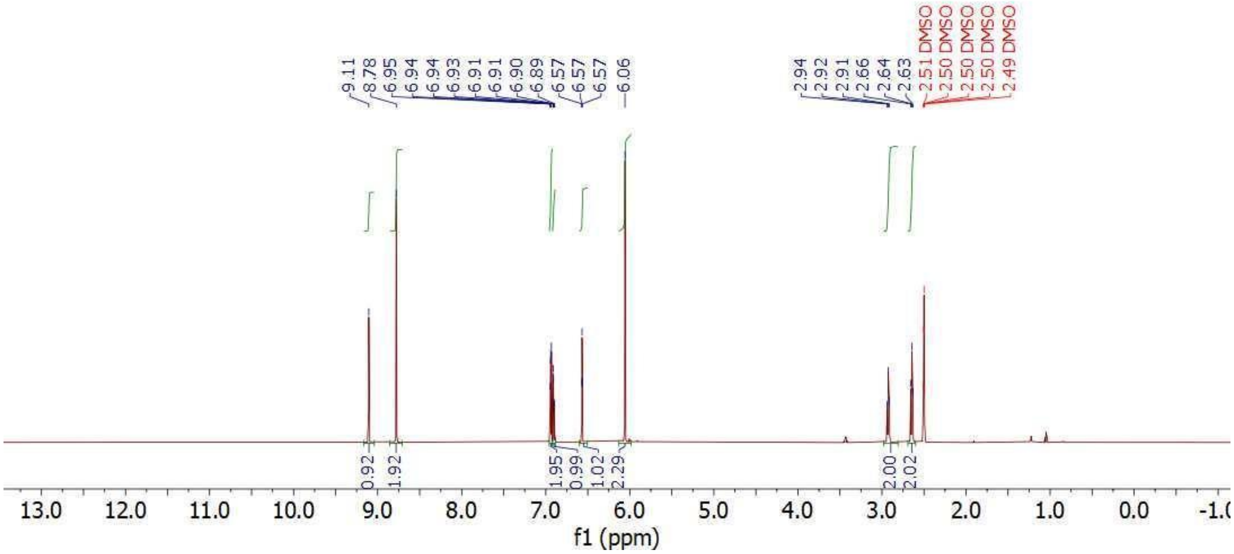

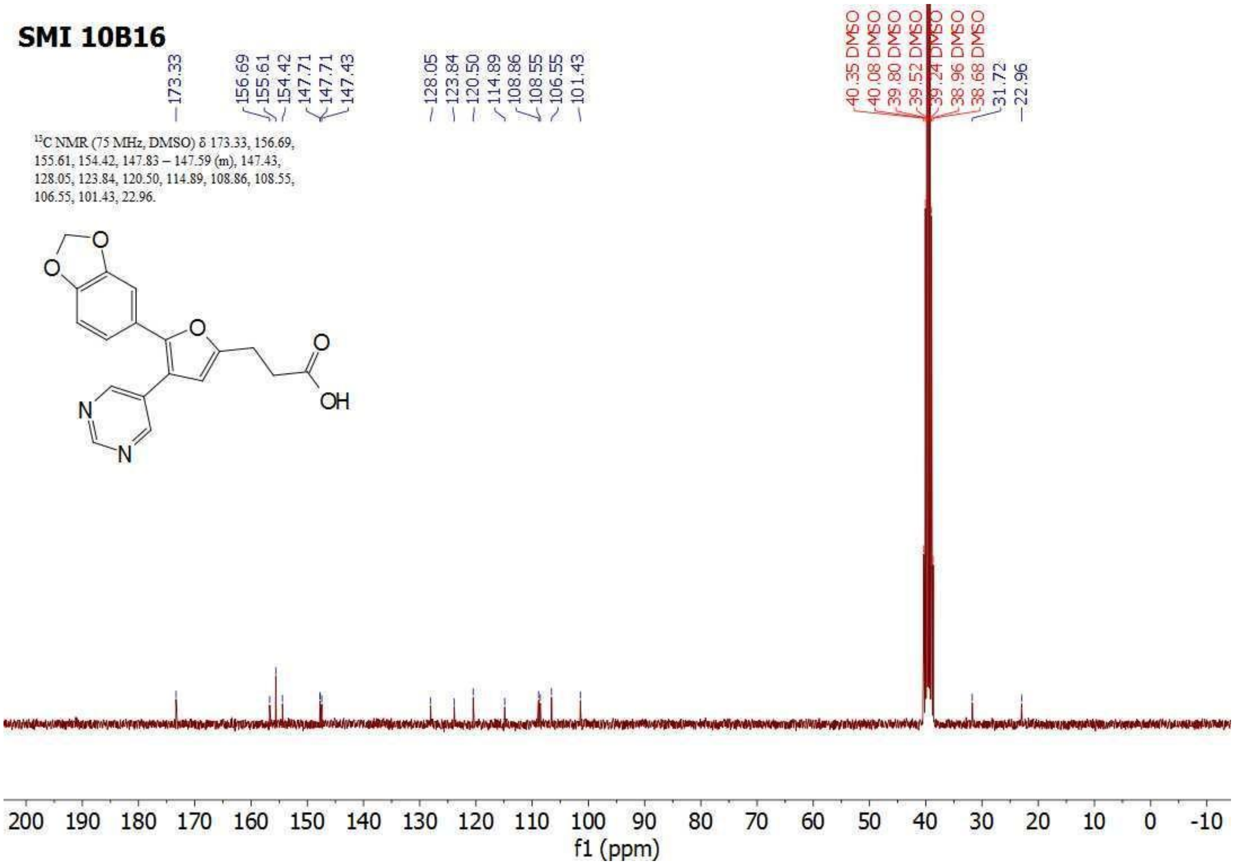

SMI-10B16, solvent DMSO, UV abs. measured at 280 nm.

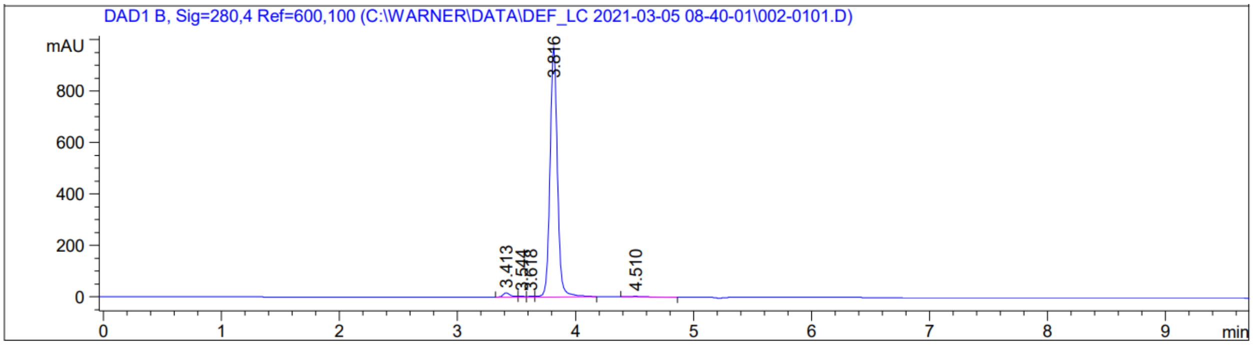

SMI 10B17

^{1}H NMR (600 MHz, $CDCl_3$ with 0.05% v/v TMS) δ 7.18 – 7.13 (m, 2H), 6.90 (dd, J = 8.1, 1.7 Hz, 2H), 6.87 (d, J = 1.8 Hz, 1H), 6.79 – 6.71 (m, 2H), 6.60 (d, J = 8.1 Hz, 1H), 6.01 (s, 1H), 5.81 (s, 2H), 3.74 (s, 3H), 2.93 (t, J = 7.8 Hz, 2H), 2.67 (t, J = 7.8 Hz, 2H).

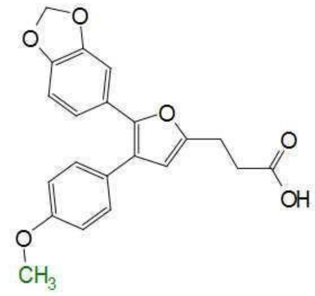

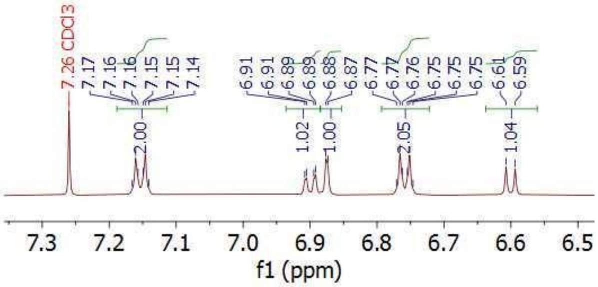

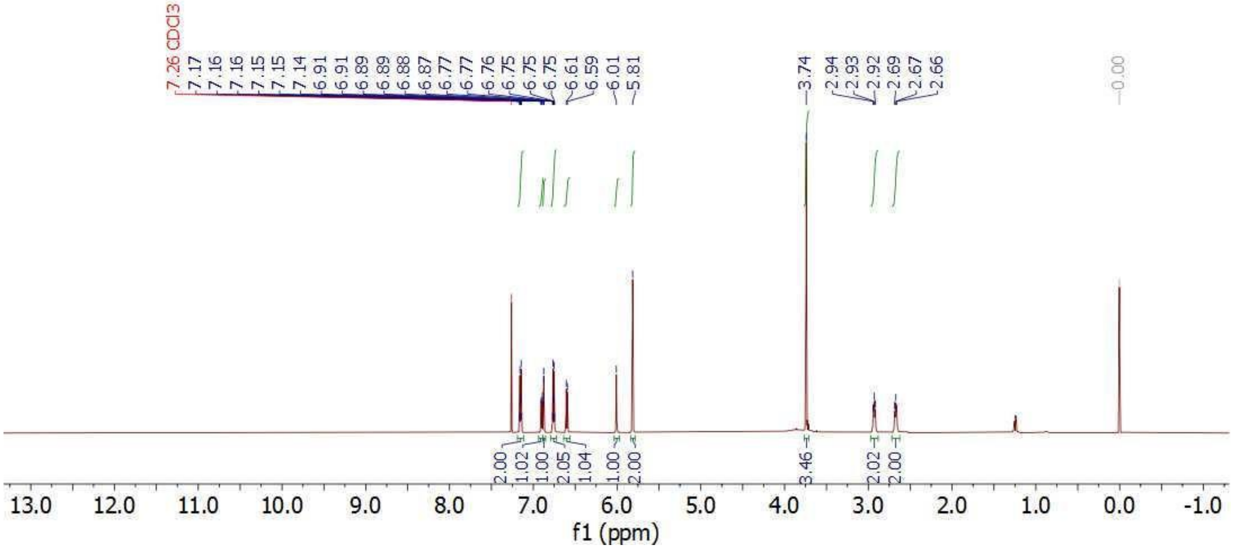

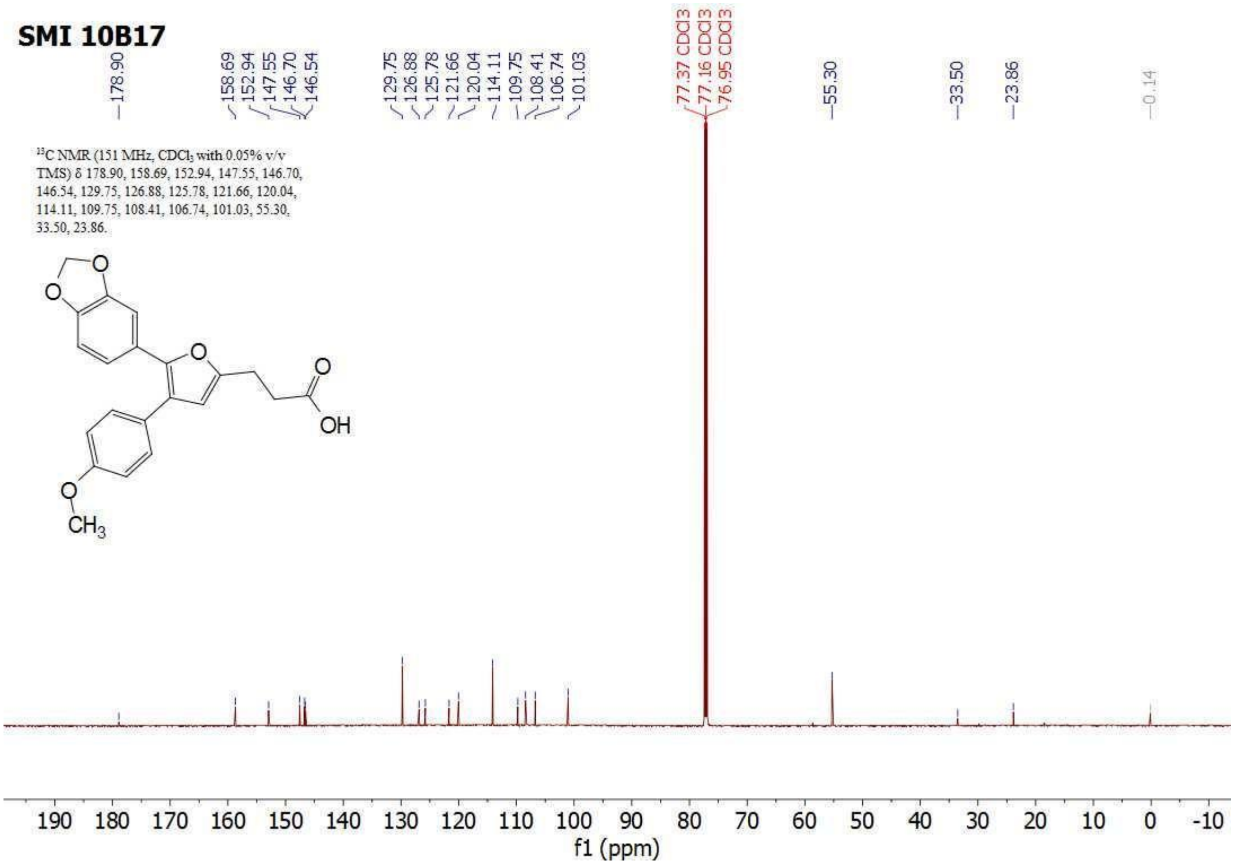

SMI-10B17, solvent DMSO, UV abs. measured at 280 nm.

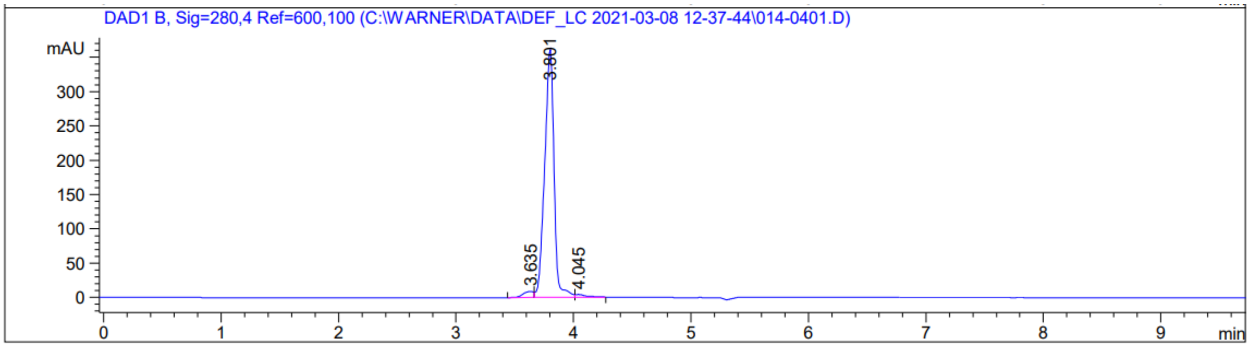

SMI 10B18

^{1}H NMR (600 MHz, $CDCl_3$) δ 10.82 (s, 1H), 7.46 (d, J = 2.1 Hz, 1H), 7.38 (d, J = 8.3 Hz, 1H), 7.19 (dd, J = 8.3, 2.0 Hz, 1H), 6.94 (dd, J = 8.2, 1.7 Hz, 1H), 6.92 (d, J = 1.7 Hz, 1H), 6.75 (d, J = 8.1 Hz, 1H), 6.17 (s, 1H), 5.97 (s, 2H), 3.04 (t, J = 7.5 Hz, 2H), 2.79 (t, J = 7.5 Hz, 2H).

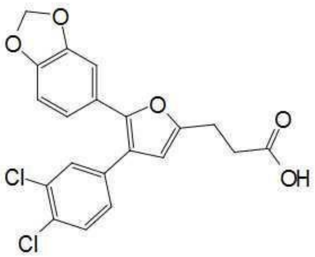

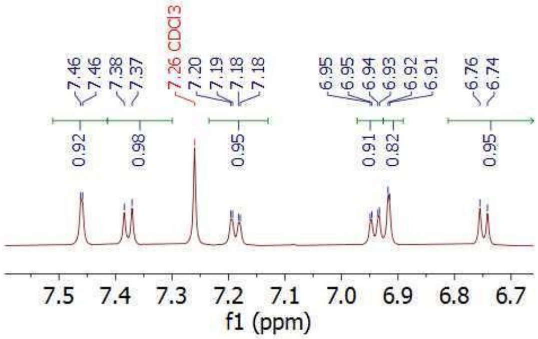

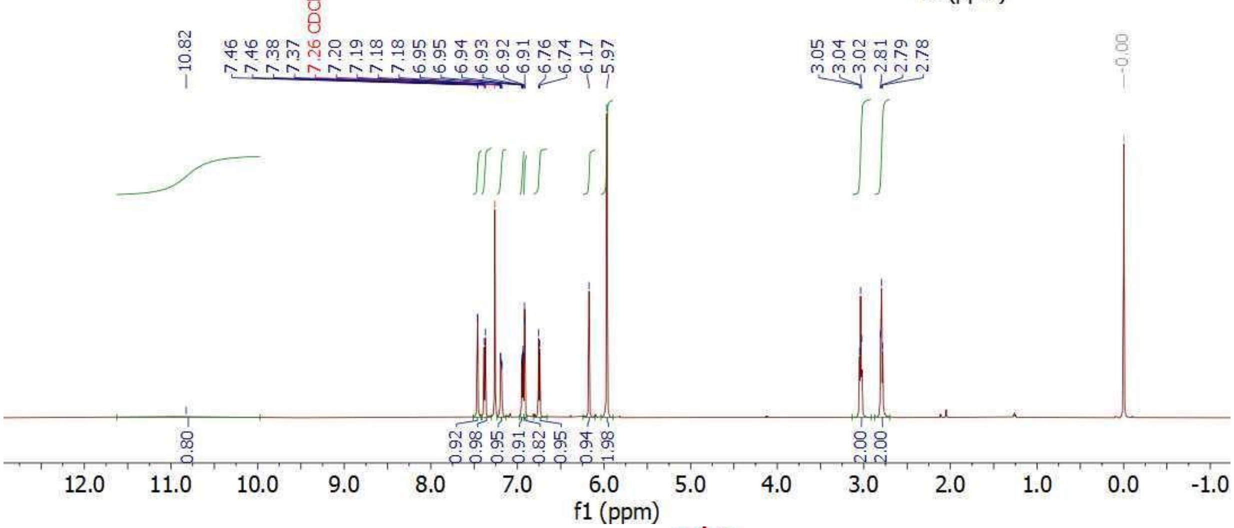

SMI 10B18

^{13}C NMR (151 MHz, $CDCl_3$ with 0.05% v/v TMS) δ 177.55, 152.94, 147.95, 147.91, 147.49, 134.61, 132.80, 131.00, 130.69, 130.30, 127.97, 124.77, 120.65, 119.72, 109.42, 108.68, 107.05, 101.35, 32.24, 23.26.

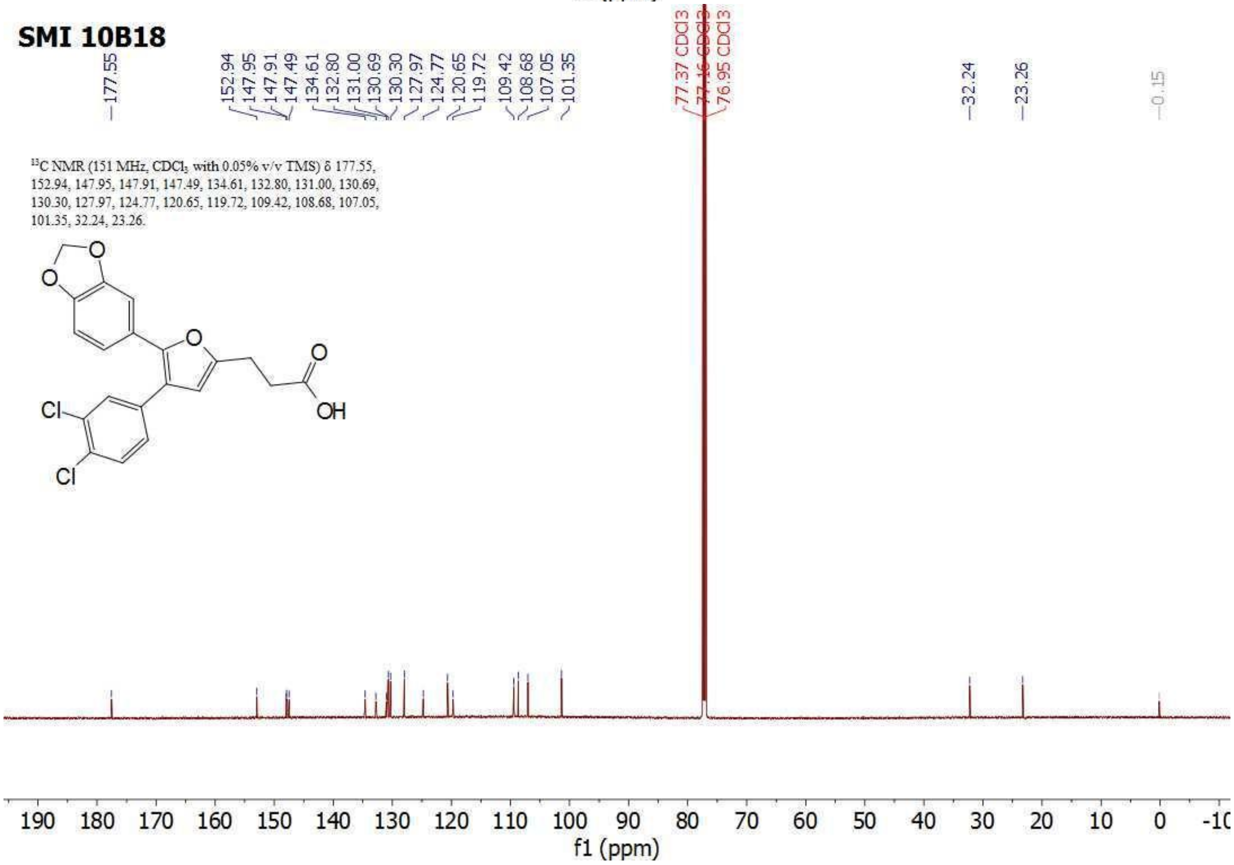

SMI-10B18, solvent DMSO, UV abs. measured at 254 nm.

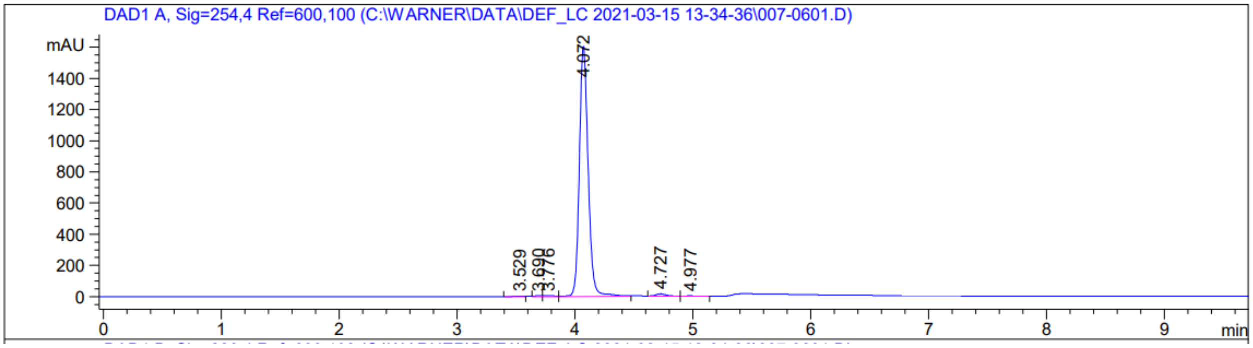

DMSO blank

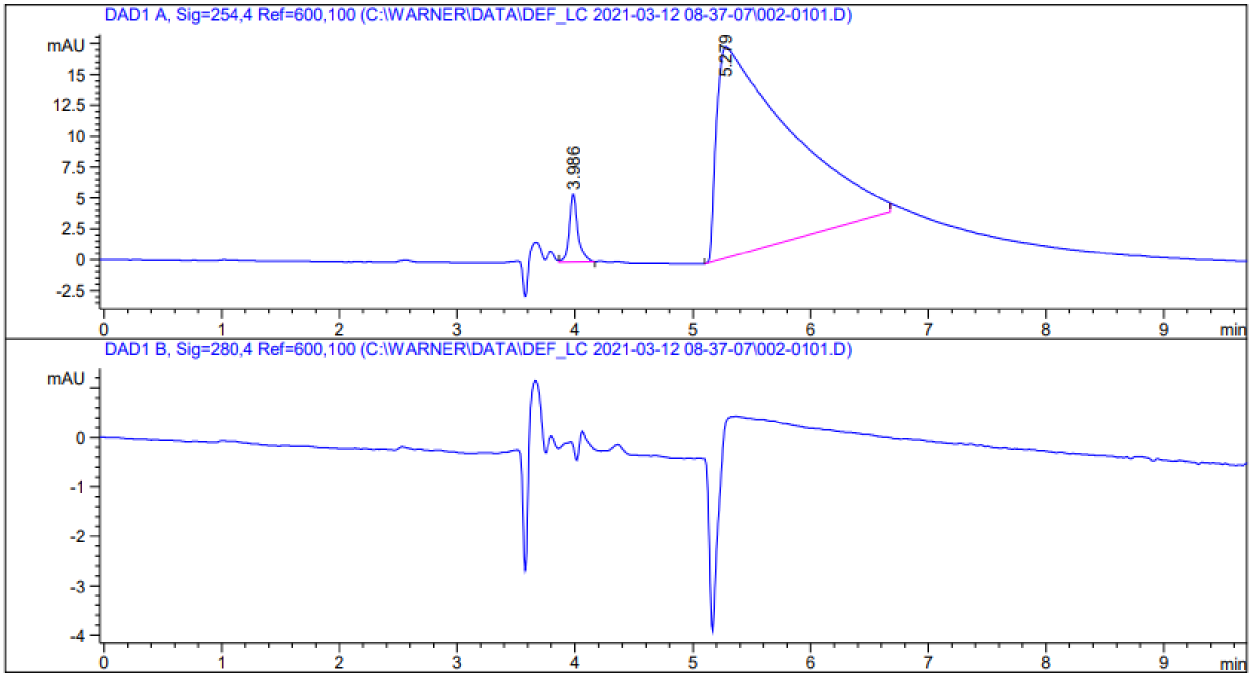

THF blank

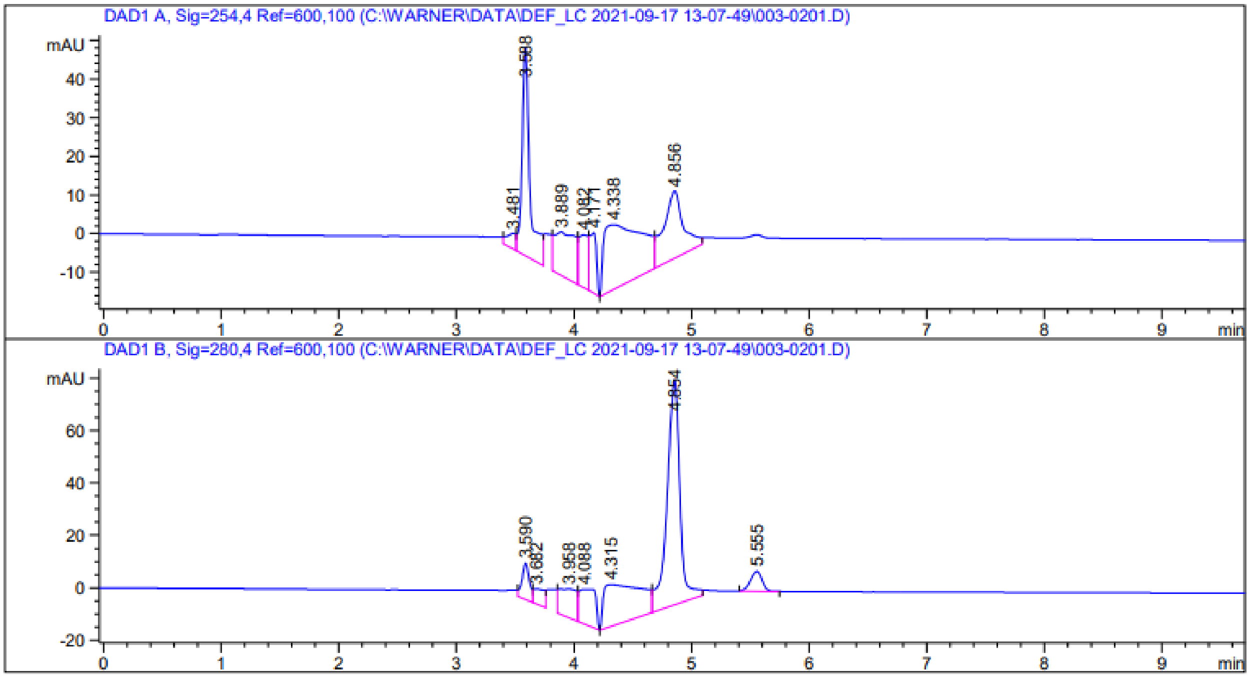

DAD1 A, Sig=254,4 Ref=600,100 (C:\WARNER\DATA\DEF_LC 2021-09-17 13-07-49\003-0201.D)
mAU
3.588
4.856
3.481
3.889
4.082
4.171
4.338
min
DAD1 B, Sig=280,4 Ref=600,100 (C:\WARNER\DATA\DEF_LC 2021-09-17 13-07-49\003-0201.D)
mAU
4.854
3.590
3.682
3.958
4.088
4.315
5.555
min
